# L-Histidine alleviates tacrolimus-induced islet dysfunction via the gut-pancreas axis

**DOI:** 10.1016/j.isci.2026.117802

**Published:** 2026-10-09

**Authors:** Zhen Geng, Qiang Li, Duo Wang, Hongji Yang, Hailian Wang, Jinku Bao, Yi Wang, Liang Wei

**Affiliations:** 1 Key Laboratory of BioResource and Eco-Environment of Ministry of Education, College of Life Science, Sichuan University, Sichuan Provincial People’s Hospital, University of Electronic Science and Technology of China, Chengdu, China; 2 Department of Gastrointestinal Surgery I Section, Renmin Hospital of Wuhan University, Wuhan 430060, China; 3 Translational Clinical Immunology Medicine Key Laboratory of Sichuan Province, Sichuan Provincial People’s Hospital, School of Medicine, University of Electronic Science and Technology of China, Chengdu, China; 4 Transplantation Center, Sichuan Provincial People’s Hospital, School of Medicine, University of Electronic Science and Technology of China, Chengdu, China; 5 Transplantation Health Management Center, Sichuan Taikang Hospital, Chengdu, China; 6 Center for Critical Care Medicine, Sichuan Provincial People’s Hospital, School of Medicine, University of Electronic Science and Technology of China, Chengdu, China; 7 Department of Dermatology, Sichuan Provincial People’s Hospital, School of Medicine, University of Electronic Science and Technology of China, Chengdu, China

**Keywords:** tacrolimus-induced diabetes, gut microbiota, L-histidine, PI3K/AKT/mTOR signaling pathway, PDX-1/ NeuroD1/MafA

## Abstract

Tacrolimus is widely used after organ transplantation but can impair pancreatic β-cell function and glucose homeostasis. Using rodent models, antibiotic-mediated microbiota depletion, fecal microbiota transplantation, 16S rRNA sequencing, metabolomic profiling, and MIN-6 cells, we examined whether microbiota-associated L-histidine contributes to β-cell protection. Tacrolimus altered gut microbial composition and increased histidine-associated microbial and metabolic signals. L-histidine supplementation improved glucose tolerance and insulin secretion, reduced β-cell apoptosis, and increased PI3K/Akt/mTOR/p70S6K signaling and PDX-1, NeuroD1, and MafA expression. Microbiota depletion attenuated several protective effects of L-histidine, supporting a contribution of the gut microbiota to this response. These findings connect a microbiota-associated amino acid with tacrolimus-related β-cell dysfunction and provide a framework for investigating microbiota-metabolite contributions to metabolic complications of immunosuppression.

## Highlights

• Tacrolimus alters gut microbiota and histidine-associated metabolism in rodents
• L-Histidine reduces tacrolimus-associated β-cell injury and apoptosis
• PI3K/AKT/mTOR signaling contributes to L-Histidine-associated protection
• L-Histidine supports PDX-1, NeuroD1, MafA expression and insulin secretion

## Teaser

Health sciences; Medicine; Medical specialty; Internal medicine; Endocrinology

## Introduction

Tacrolimus (TAC, also named FK506) is one of the immune suppressants widely applied for patients post-transplantation; however, it has been documented to induce islet dysfunction.^1^^,^^2^ Researchers found that TAC mediated islet dysfunction via the inhibition of voltage-gated Ca_2_^+^ entry,^2^ suppression of glucose-induced insulin release via reducing glucokinase activity,^3^ and the increase of sodium glucose co-transporter type 2 (SGLT-2) to impair β-cell function.^4^ By binding with FK506-binding protein 12 (FKBP12), TAC inhibits the activation of mTOR and lead to reduced insulin secretion.^5^^,^^6^ Because TAC induced islet dysfunction is reversible, it is of crucial importance to find an agent to counteract the cytotoxicity of TAC and to rescue the function if islet β-cell.

In recent years, gut-microbiota has drawn attention to TAC induced hyperglycemia via the gut-pancreas axis. These studies focused on glucagon-like peptide-1 (GLP-1) related signaling pathways,^7^ including butyrate, bile acid-glucuronic and farnesoid X receptor (FXR).^8^ However, as the majority of the studies focused on the gut microbiota and dysbiosis,^9^^,^^10^ no specific metabolite, except butyrate, was found and further validated.

Therefore, in the current study, we performed the bioinformatic analysis of the gut microbiota and metabolites profile to mining the potential metabolites, which are increased in response to TAC treatment. Then, we investigated whether the increased metabolite possess the function of islet protection by both *in vitro* and *in vivo* studies. Firstly, we found that L-histidine (L-His) was increased among the gut microbiota metabolites, which could counteract the cytotoxicity of TAC. Further studies revealed that L-His possess dual role of islet protective in both maintaining the cell viability via PI3K/AKT/mTOR pathway and stimulating the viable cell function via the upregulation of insulin upstream genes. Taken together, our study suggested that the TAC induced islet dysfunction could be reversed by L-His.

## Results

### TAC induces hyperglycemia with aberrant intestinal dysbiosis

In this study, we established the TAC induced islet dysfunction model in rats, so that we could have enough serum for further analysis. As demonstrated by Figure 1A, during the whole experimental period, the weight of the rats in the TAC group was lower than that of the controls. In contrast to body weight, random blood glucose (BG) levels gradually increased in the TAC group while the BG levels of the control group showed normal glycemia (Figure 1B, *p* < 0.001). Glucose tolerance test also demonstrated that the TAC-treated mice had impaired islet function compared with that of the controls (Figure 1C).

**Figure 1:**
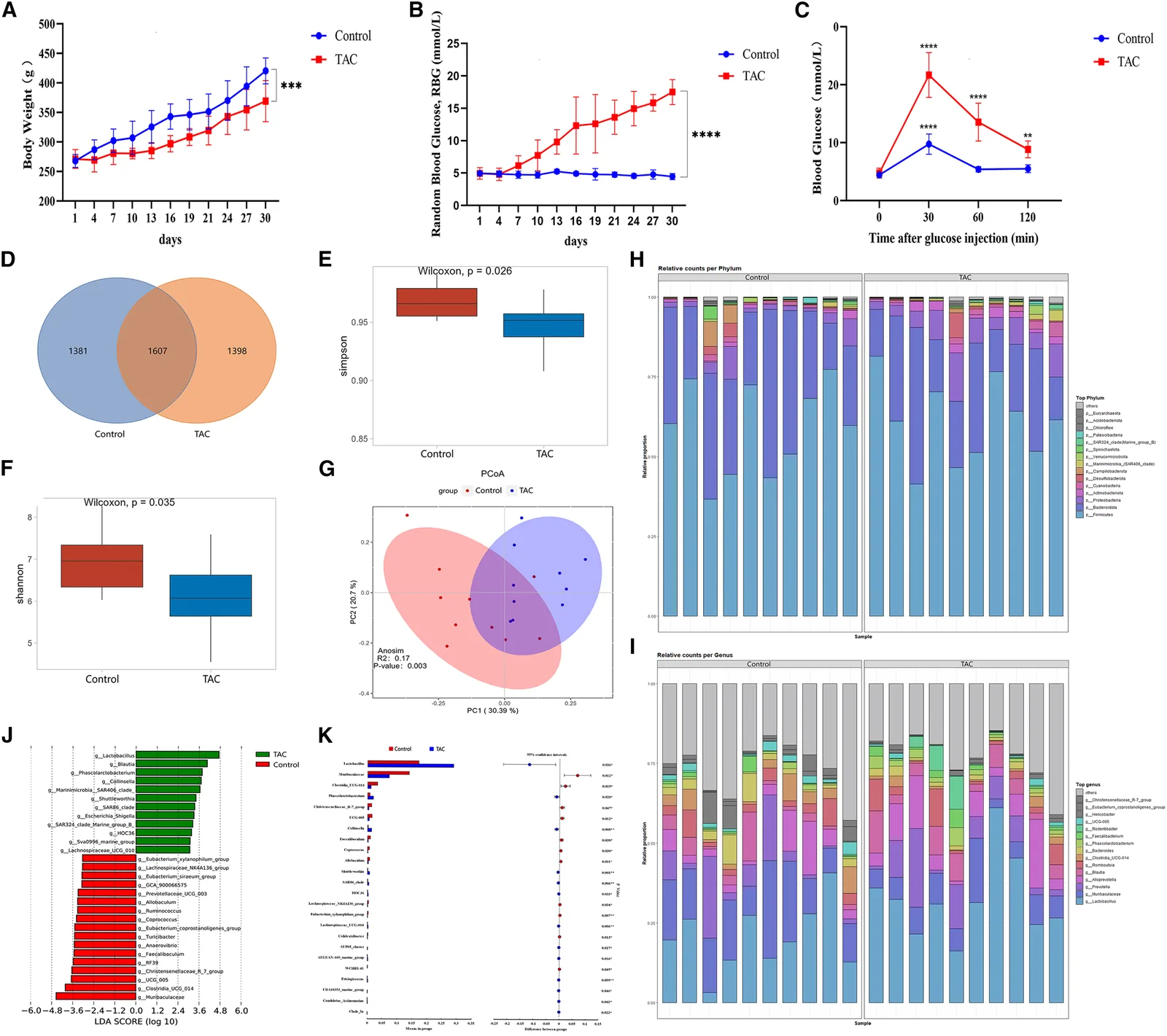
Glucose metabolism and intestinal dysbiosis analysis in animal models (A) Body weights of different groups. (B) Random blood glucose levels of different groups. (C) Intraperitoneal glucose tolerance test (IPGTT). (D) Venn diagram showing ASVs (amplicon sequence variants) between the control group and the TAC group. (E and F) Simpson index (E) and Shannon index (F) showed higher flora richness in the control group, but lower flora richness in the TAC group (*p* < 0.05). (G) PCoA analysis diagram of gut microbiota in the control group and TAC group (*p* < 0.05). (H and I) Different composition between the control and TAC groups at the phylum level (H) and the genus level (I). (J) The gut microbiota of animal models was analyzed by LEfSe. (K) The relative abundances, *p* value, and confidence intervals of the gut microbial community in the feces from the control group and the TAC group on the 30th day. Data are expressed as mean ± SD. The control group was shown in blue (●) and the tacrolimus group (TAC) was shown in red (■).

Subsequently, we collected the fecal of these rats to investigate whether TAC has any impact on gut microbiota. The abundance features of amplicon sequence variants (ASVs) in the samples are primarily reflected in the detection results. A total of 4,386 ASVs were found, of which 1,398 were unique to the TAC group, 1,381 were distinct to the control group, and 1,607 were identical to both groups (Figure 1D). Further analysis demonstrates that there was a substantial difference (*p* <0.05) between the two groups, with the control group having a higher species richness and the TAC group having a lower species richness by using Simpson index (Figure 1E) and Shannon index (Figure 1F). Additionally, the Principal Coordinate Analysis (PCoA) analysis demonstrated that the gut microbiota composition of the TAC group differed from that of the control group (Figure 1G).

We then analyzed the abundance of gut microbiota at the phylum and genus level. At phylum level, *Firmicutes* (*p* <0.05), *Proteobacteria* (*p* <0.05) and other taxa were more abundant in the TAC group, whereas *Bacteroidota* was less abundant (Figure 1H). At genus level, *Lactobacillus* (*p* <0.05), *Phascolarctobacterium* (*p* <0.05), and other genera were increased in the TAC group, whereas *Muribaculacea* (*p* <0.05), *Clostridia_UCG-014* (*p* <0.05), and *Eubacterium_coprostanoligenes_group* (*p* <0.05) were decreased (Figure 1I).

The gut microbiota of animal models was further analyzed by Linear discriminant analysis Effect Size (LEfSe) method. As shown in Figure 1J, the relative abundance of 12 bacterial genera, including *Lactobacillus*, *Blautia*, *Phascolarctobacterium*, and *Collinsella*, increased significantly in the TAC group (LDA score>3, *p* <0.05). Conversely, 17 species from the bacterial genera with considerably higher abundance were found in the control group (Figure 1J). We next used Welch’s *t*-test to excluded the impact of low-abundance bacterial communities. The results showed that 11 bacterial genera, including *Muribaculaceae*, were significantly depleted in the TAC group, whereas 13 bacterial genera, including *Lactobacillus*, were significantly enriched (Figure 1K). Collectively, these findings suggest that TAC-induced metabolic disorder may closely associated with alterations in gut microbial diversity and composition.

### Metabolite changes in feces and serum of TAC-induced diabetic rats

Furthermore, we analyzed the metabolites to determine whether TAC-induced diabetes is caused by Short-Chain Fatty Acid (SCFA) or other metabolites. The fecal (Figure 2A) and serum metabolites (Figure 2B) differed significantly, according to the principal component analysis (PCA) results. Additionally, the partial least squares discrimination analysis (PLS-DA) results (Figures 2C and 2D) demonstrated that the two groups’ serum and fecal metabolites differed.

**Figure 2:**
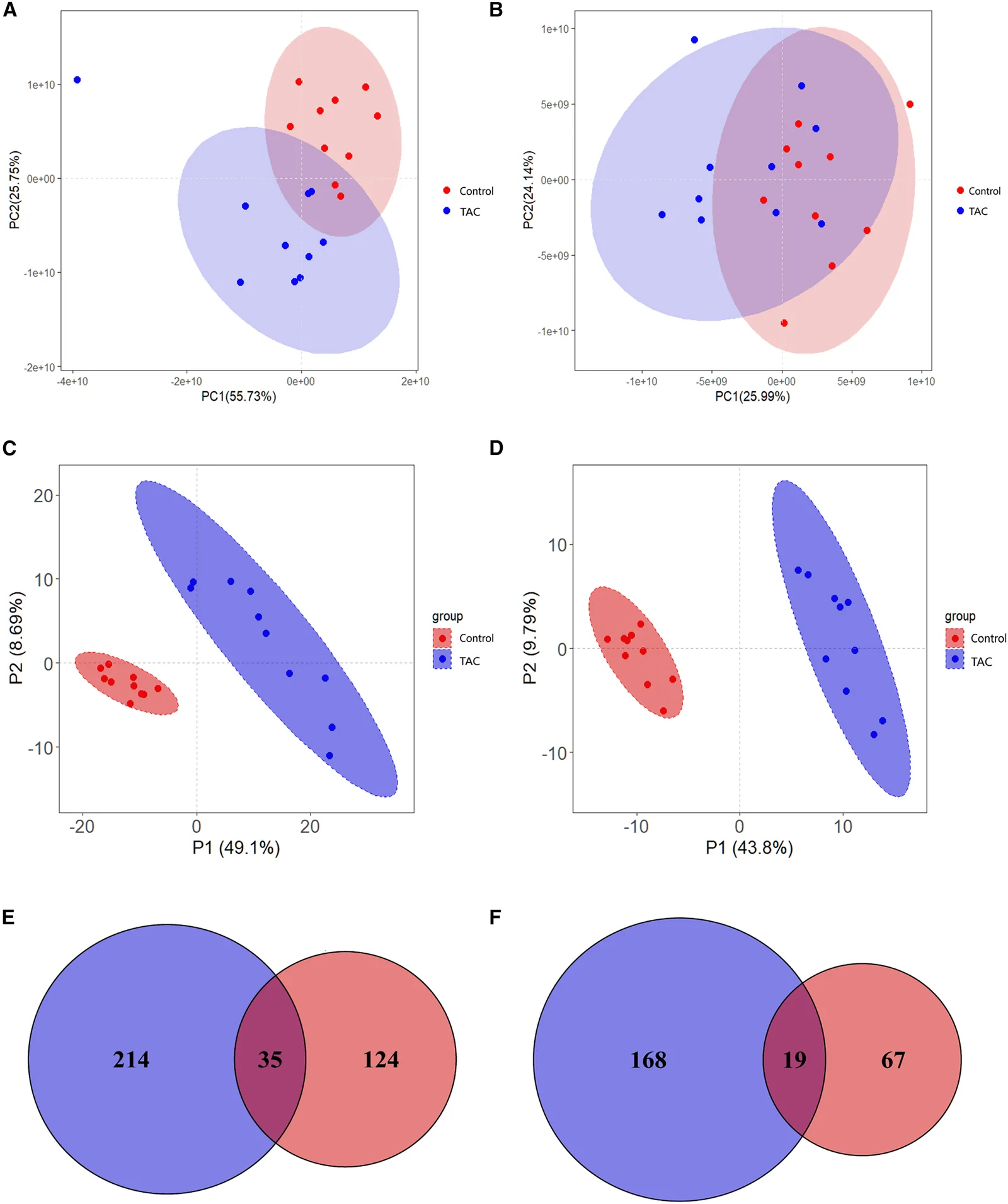
Tacrolimus changed the composition of the metabolites (A) Principal component analysis (PCA) diagram of fecal sample. (B) PCA diagram of serum samples. (C) Partial least squares discrimination analysis (PLS-DA) diagram of fecal samples. (D) PLS-DA diagram of serum samples. (E) 35 metabolites showed an increasing trend in fecal and serum samples. (F) 19 metabolites showed a decreasing trend in fecal and serum samples. The blue circles represent metabolites in the stool, the red circles represent metabolites in the serum, and the overlaps represent metabolites in the stool and serum that change in the same trend. *p* < 0.05, VIP (variable importance in projection) ≥ 1 for data.

To investigate the specific metabolites, we performed further analysis. The trend of the metabolites with notable variations between the two groups is displayed in Figures 2E and 2F. The metabolites of the fecal and serum samples are represented by the blue and red circles, respectively, and the overlapping portions indicate the metabolites that share a common trend between the two samples. The findings indicate that 35 metabolites, such as D-(-)-aspartic acid, D-(-)-mannitol, 4-(hydroxymethyl)benzoic acid, LPG 16:0, 3-(4-hydroxyphenyl)propionic acid, 4-pyridoxic acid, D-xylonic acid, 2-hydroxy-3-methylbutanoic acid, and other metabolites—have significantly increased in both feces and serum under TAC induction (Figure 2E), while 19 metabolites have significantly decreased (Figure 2F). Table 1 displayed the 35 increased metabolites detected in serum and feces, whereas Table 2 demonstrated the 19 decreased metabolites in the TAC group. For example, D-xylonic acid levels increased significantly in both serum and feces, whereas glycodeoxycholic acid (hydrate) and deoxycholic acid levels were greatly reduced in the TAC group. Meanwhile, the concentrations of metabolites like allantoate acid, N1, N2-dicyclohexylethanedithioamide, [3,4-b] 4,6-dimethyl-2,7-diphenyl-3,7-dihydro-2H-pyrazolo 3-one pyridin, 1-(4-methoxyphenyl) 5-[(10Z)-14-(3,5-dihydroxyphenyl)tetradec-10-en-1-yl] propane-1,2-diol 3-phenyl-5-(trifluoromethyl)-4,5-dihydro-1H-pyrazol-5-ol and benzene-1,3-diol were also reduced (Table 2).

**Table 1:** Table of 35 metabolites with increased expression of serum and feces

Name	Feces	Feces	Feces	Serum	Serum	Serum
Name	control	TAC	log2FC	control	TAC	log2FC
D-Xylonic Acid	1.35E+07	2.22E+08	4	2.31E+07	4.82E+07	1.1
D-(-)-Mannitol	6.15E+06	5.51E+07	3.2	8.36E+06	2.51E+07	1.6
Ranitidine N-oxide	3.22E+06	2.45E+07	2.9	2.42E+06	4.12E+06	0.8
L-Histidine	6.07E+07	3.11E+08	2.4	8.28E+07	1.07E+08	0.4
LPG 16:0	2.46E+06	1.22E+07	2.3	1.98E+06	5.02E+06	1.3
gamma-Glutamylleucine	7.51E+07	3.55E+08	2.2	8.16E+07	1.28E+08	0.6
HRH	3.84E+06	1.75E+07	2.2	2.12E+06	2.98E+06	0.5
3-[(methoxycarbonyl)amino]-2,2,3-trimethylbutanoic acid	9.24E+06	3.96E+07	2.1	5.04E+06	9.40E+06	0.9
Orotic acid	4.76E+06	2.04E+07	2.1	1.67E+06	3.21E+06	0.9
Creatine phosphate	1.13E+06	4.51E+06	2	9.13E+05	1.28E+06	0.5
4-Pyridoxic acid	1.69E+07	6.21E+07	1.9	5.73E+06	1.22E+07	1.1
2-Hydroxybutyric acid	3.67E+07	1.24E+08	1.8	1.24E+07	2.15E+07	0.8
Choline	1.18E+08	3.78E+08	1.7	4.37E+08	5.92E+08	0.4
N-Acetylglucosamine 1-phosphate	1.89E+05	6.05E+05	1.7	4.89E+05	6.94E+05	0.5
DNK	1.98E+06	6.14E+06	1.6	1.76E+06	2.49E+06	0.5
Eugenol	1.79E+06	5.46E+06	1.6	1.44E+06	2.04E+06	0.5
2-Hydroxy-1-(4-methoxyphenyl)propyl hexopyranoside	8.37E+05	2.38E+06	1.5	3.32E+05	6.12E+05	0.9
2-Hydroxy-3-methylbutanoic acid	4.36E+07	1.17E+08	1.4	2.71E+07	5.27E+07	1
D-(+)-Proline	5.12E+08	1.32E+09	1.4	4.00E+08	6.41E+08	0.7
(+/-)12(13)-DiHOME	3.03E+08	7.60E+08	1.3	1.05E+08	1.96E+08	0.9
2-Aminobenzenesulfonic acid	2.70E+06	6.53E+06	1.3	1.49E+06	1.98E+06	0.4
Sphinganine	5.52E+05	1.33E+06	1.3	1.36E+06	1.72E+06	0.3
D-(-)-Aspartic acid	5.75E+07	1.33E+08	1.2	4.02E+07	1.37E+08	1.8
Lysope 16:0	7.99E+07	1.83E+08	1.2	8.80E+06	1.37E+07	0.6
3-(4-Hydroxyphenyl)propionic acid	1.42E+06	3.08E+06	1.1	3.29E+06	6.82E+06	1.1
5-(*tert*-butyl)-2-methyl-N-(5-methyl-3-isoxazolyl)-3-furamide	1.18E+07	2.46E+07	1.1	7.19E+06	1.08E+07	0.6
Linderalactone	2.59E+06	5.62E+06	1.1	5.49E+06	9.71E+06	0.8
2-(acetylamino)-3-[4-(acetylamino)phenyl]acrylic acid	9.78E+06	1.89E+07	1	3.98E+06	5.74E+06	0.5
Methyl nicotinate	9.91E+07	2.02E+08	1	8.01E+07	1.39E+08	0.8
N3,N4-Dimethyl-L-arginine	1.32E+08	2.49E+08	0.9	4.22E+07	6.80E+07	0.7
4-(hydroxymethyl)benzoic acid	1.17E+06	2.00E+06	0.8	2.03E+06	5.62E+06	1.5
Dehydrotumulosic acid	8.71E+05	1.46E+06	0.7	1.83E+05	2.56E+05	0.5
N-Methylthreonine	3.46E+06	5.54E+06	0.7	2.11E+06	2.76E+06	0.4
3-amino-4-(propylamino)cyclobut-3-ene-1,2-dione	1.62E+08	2.42E+08	0.6	1.20E+08	1.66E+08	0.5
N-Acetylornithine	9.07E+07	1.39E+08	0.6	4.13E+07	6.67E+07	0.7

Data are expressed as mean ± SD, *N* = 6 per group. *p* < 0.05. | log2FC |= 1, representing a two-fold difference in metabolites between the two groups.

**Table 2:** Table of 19 metabolites with decreased expression of serum and feces

Name	Feces	Feces	Feces	Serum	Serum	Serum
Name	control	TAC	log2FC	control	TAC	log2FC
4,6-dimethyl-2,7-diphenyl-3,7-dihydro-2H-pyrazolo[3,4-*b*]pyridin-3-one	4.01E+07	9.55E+05	-5.4	4.33E+06	1.28E+06	-1.8
N1,N2-dicyclohexylethanedithioamide	8.97E+06	9.02E+05	-3.3	2.58E+06	4.62E+05	-2.5
Deoxycholic Acid	1.42E+10	1.67E+09	-3.1	9.11E+08	7.59E+07	-3.6
Glycodeoxycholic Acid (hydrate)	1.33E+07	1.96E+06	-2.8	1.04E+08	2.24E+06	-5.5
Skatole	2.09E+07	3.59E+06	-2.5	5.64E+06	2.99E+06	-0.9
1-(3,4-dimethoxyphenyl)ethan-1-one oxime	1.24E+07	2.33E+06	-2.4	2.39E+06	1.55E+06	-0.6
5-[(10Z)-14-(3,5-dihydroxyphenyl)tetradec-10-en-1-yl]benzene-1,3-diol	4.47E+08	1.14E+08	-2	1.86E+07	6.46E+06	-1.5
Androsterone	1.35E+07	3.67E+06	-1.9	8.10E+05	5.10E+05	-0.7
Ergosta-5,7,9(11),22-Tetraen-3-beta-Ol	4.64E+07	1.27E+07	-1.9	1.86E+06	1.18E+06	-0.7
1-(4-methoxyphenyl)propane-1,2-diol	5.54E+07	1.55E+07	-1.8	3.64E+07	1.18E+07	-1.6
N-Desmethylvenlafaxine	8.18E+06	2.60E+06	-1.7	1.27E+06	8.56E+05	-0.6
Delta-Tridecalactone	2.03E+07	7.32E+06	-1.5	3.70E+06	2.49E+06	-0.6
ALK	1.29E+06	5.10E+05	-1.3	4.94E+05	3.75E+05	-0.4
6 β-Hydroxycortisol	1.21E+06	2.31E+06	-1.08	5.80E+05	8.76E+05	-1.68
2-Furoylglycine	5.54E+06	2.82E+06	-1	1.26E+06	8.41E+05	-0.6
4-[1-(dimethylamino)ethylidene]-2-phenyl-1,3-oxazol-5(4H)-one	1.92E+06	1.04E+06	-0.9	9.05E+05	6.54E+05	-0.5
allantoate	1.16E+08	6.38E+07	-0.9	1.26E+08	3.39E+07	-1.9
Cinchonine	4.80E+06	2.83E+06	-0.8	1.80E+06	1.26E+06	-0.5
3-phenyl-5-(trifluoromethyl)-4,5-dihydro-1H-pyrazol-5-ol	2.40E+06	1.74E+06	-0.5	3.09E+06	1.26E+06	-1.3

Data are expressed as mean ± SD, *N*=6 per group. *p* < 0.05. | log2FC |= 1, representing a two-fold difference in metabolites between the two groups.

### TAC-associated gut microbiota promote L-histidine production and imidazole propionate accumulation

Our previous study demonstrated that transplant recipients with diabetes had significantly different and characteristic gut microbiota and metabolites compared with the healthy controls.^11^ To identify microbial taxa associated with the TAC-responsive metabolites, we performed correlation analyses between the differentially abundant gut microbes and fecal or serum L-histidine and D-(-)-mannitol. In fecal samples, both metabolites were positively correlated with HOC36, SAR86_clade, *Collinsella*, and *Lactobacillus*, and negatively correlated with *Allobaculum* and *Muribaculaceae* (Figure 3A). A similar pattern was observed in serum, where L-histidine and D-(-)-mannitol were positively associated with Lactobacillus, SAR86_clade, Collinsella, and related taxa but negatively associated with *Allobaculum*, *Faecalibaculum*, and *Muribaculaceae* (Figure 3B). These results linked the TAC-associated microbial shift to altered histidine metabolism.

**Figure 3:**
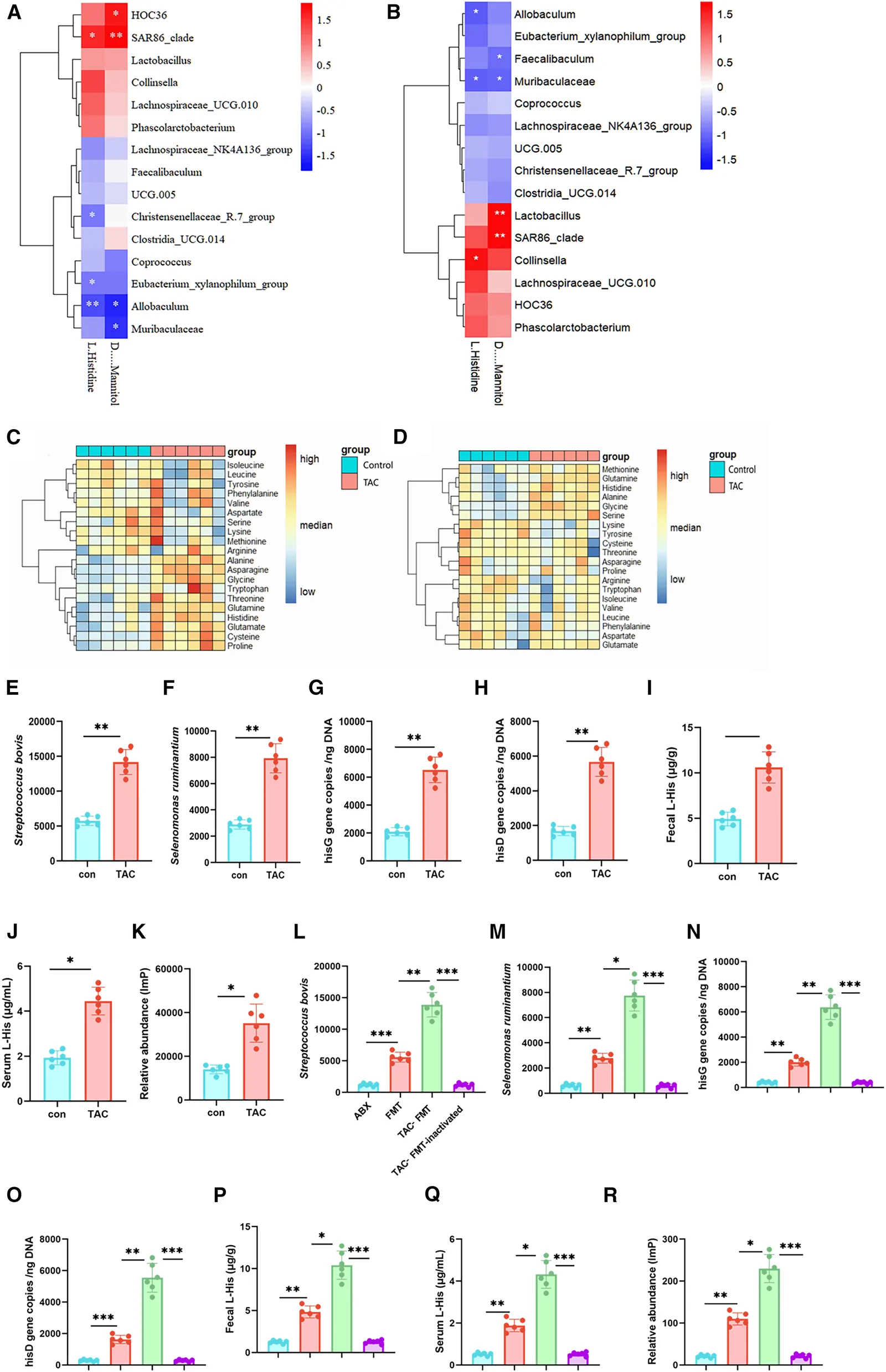
TAC-associated gut microbiota increases microbial L-histidine biosynthesis (A and B) Spearman correlation heatmaps showing associations between differentially abundant gut microbes and fecal (A) or serum (B) L-histidine and D-(-)-mannitol. (C and D) Heatmaps of targeted amino acid profiles in fecal (C) and serum (D) samples from control and TAC-treated animals. (E and F) Quantification of *Streptococcus bovis* (E) and *Selenomonas ruminantium* (F) in fecal samples. (G and H) Copy numbers of the microbial histidine-biosynthesis genes hisG (G) and hisD (H), normalized to fecal DNA. (I and J) L-Histidine concentrations in feces (I) and serum (J). (K) Relative abundance of imidazole propionate (ImP). (L–R) Effects of fecal microbiota transplantation on *S. bovis* (L), *S. ruminantium* (M), hisG (N), hisD (O), fecal L-histidine (P), serum L-histidine (Q), and ImP (R). ABX, antibiotic-treated group; FMT, recipients of fecal microbiota from control donors; TAC-FMT, recipients of fecal microbiota from TAC-treated donors; TAC-FMT-inactivated, recipients of inactivated fecal microbiota from TAC-treated donors. Data are presented as mean ± SD. Statistical tests: Spearman’s correlation was used for (A) and (B); the Wilcoxon rank-sum test was used for the two-group comparisons in (E)–(K); and one-way ANOVA was used for the multi-group comparisons in (L)–(R). Asterisks denote ∗*p* < 0.05, ∗∗*p* < 0.01, and ∗∗∗*p* < 0.001. For the animal experiments in this figure, *n* = 6 per group.

**Figure 4:**
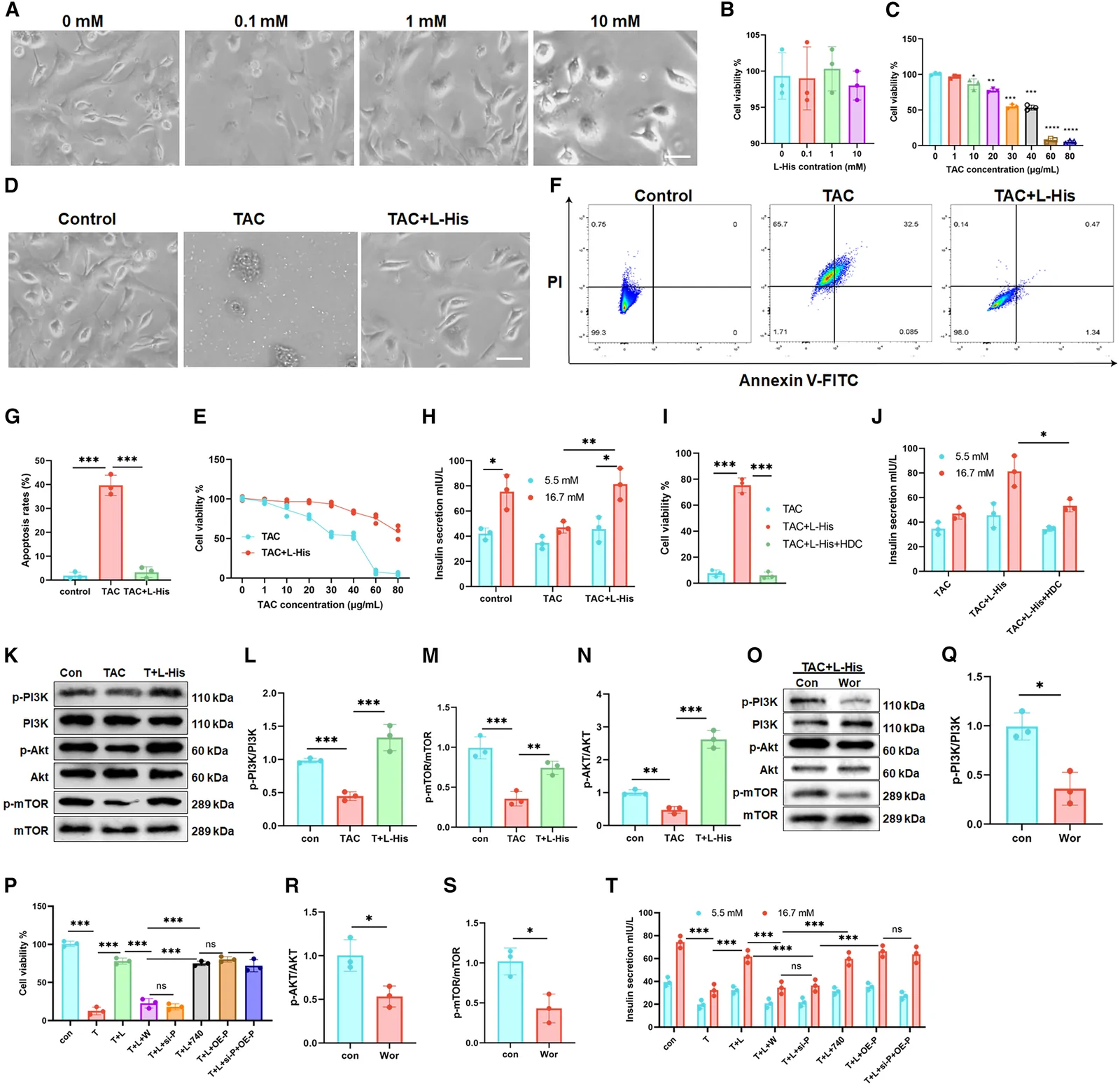
L-His counteracts TAC damage in islets (A) MIN-6 cells cultured with different dose of L-His (0, 0.1, 1, and 10 mM). The scale bars (10 μm) are labeled in the lower right corner, with a magnification of 400×. (B) Cell viability assay of MIN-6 cells with different doses of L-His. (C) Cell viability of MIN-6 cells with different doses of TAC. (D) Cell morphology of the control cells, TAC-treated cells, and TAC plus L-His cells. (E) Representative picture of flow cytometry of the control cells, TAC-treated cells, and TAC plus L-His cells. (F) Statistics of apoptosis rates. (G) Cell viability of different dose of TAC and TAC plus L-His. (H) Glucose stimulated insulin secretion of the three groups. (I) Cell viability data of L-His and its inhibitor. (J) Glucose stimulated insulin secretion of L-His and its inhibitor. (K) Immunoblotting of control cells, TAC-treated cells, and TAC plus L-His-treated cells. (L–N) Relative protein levels of activated PI3K, Akt, mTOR versus total protein. (O) Immunoblotting of PI3K inhibitor and the control solvent. (P) Cell viability of MIN-6 cell with control solvent, TAC, TAC+L-His, and TAC+L-His+wortmannin. (Q–S) Relative protein levels of activated PI3K, Akt, mTOR versus total protein. (T) Glucose stimulated insulin secretion. Statistical test for quantitative comparisons denoted by asterisks: one-way ANOVA. Asterisks denote ∗*p* < 0.05, ∗∗*p* < 0.01, and ∗∗∗*p* < 0.001. For MIN-6 experiments, *n* = 3 independent experiments.

**Figure 5:**
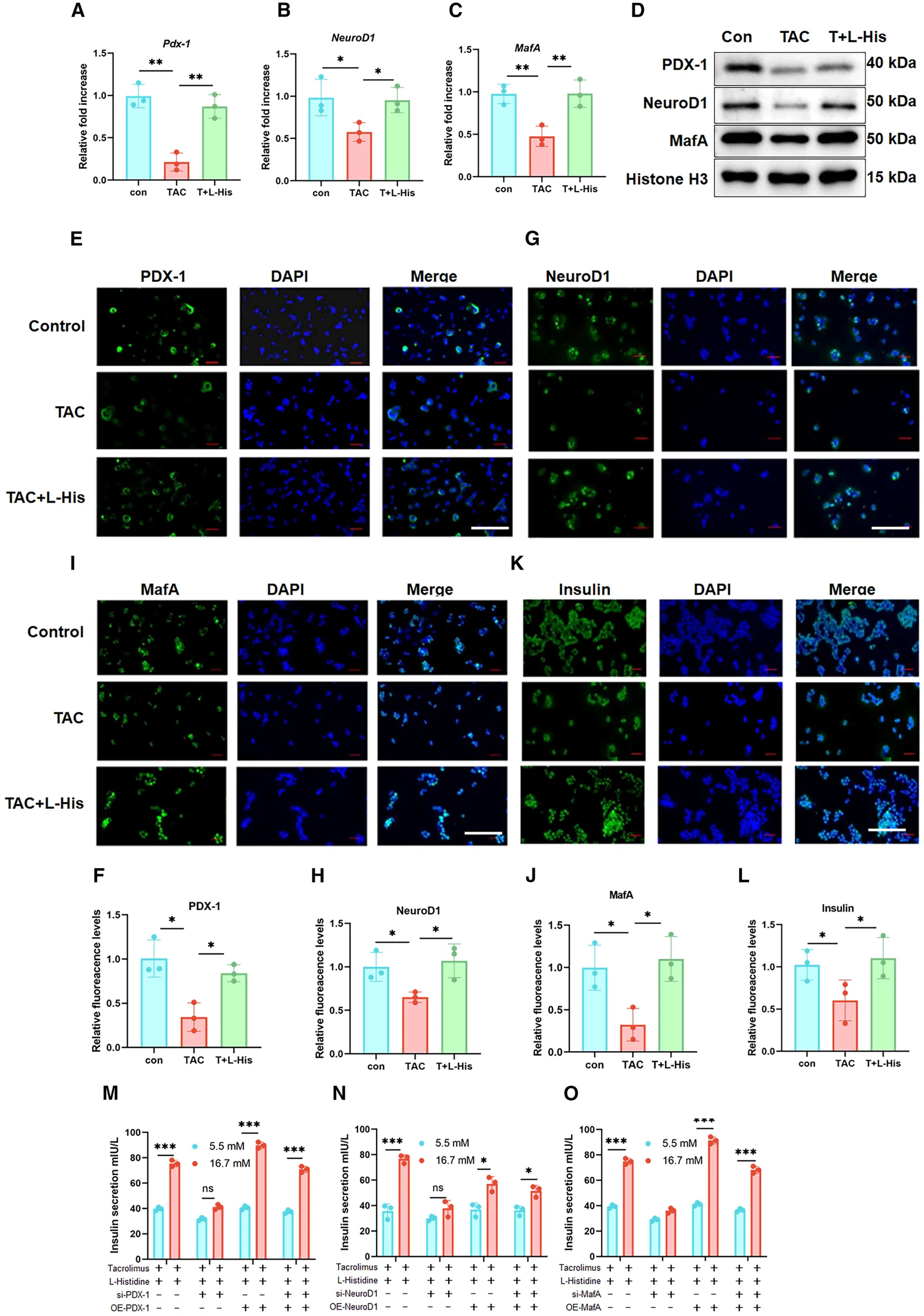
L-His stimulate the islet function via the upregulation of PDX-1/NeuroD1/MafA Real-time PCR of Pdx-1 (A), NeuroD1 (B) and MafA (C). Western blotting of PDX-1, NeuroD1, and MafA (D). Immunofluorescent staining of PDX-1 (E), Neuro D1 (G), MafA (I) and Insulin (K) in MIN-6 cells of the control, TAC, and TAC+L-His treated groups. Statistics of fluorescent intensity of PDX-1 (F), Neuro D1 (H), MafA (J), and insulin (L). Scale bars represent 50 μm with a magnification of 200×. GISI of silencing and overexpression of PDX-1 (M), NeuroD1 (N) and MafA (O) at the presence of L-Histidine and TAC. All data are presented as mean ± SD. Statistical test for quantitative comparisons denoted by asterisks: one-way ANOVA. Asterisks denote ∗*p* < 0.05, ∗∗*p* < 0.01, and ∗∗∗*p* < 0.001. For MIN-6 experiments, *n* = 3 independent experiments.

Judged from Table 1, D-Mannitol is also increased as well as L-His, and mannitol has been widely investigated to maintain glucose homeostasis,^12^^,^^13^^,^^14^ but its adverse effects is also well studied.^15^ So, we performed cell viability assay, apoptosis assay and function to see whether L-His has similar glucose regulation and islet β-cell protective function as mannitol. We found that L-His could increase cell viability (Figure S1A), decrease apoptosis (Figures S1B and S1C) and improve glucose response (Figure S1D) to the extent of mannitol. Further, the imidazole propionate (ImP) challenge experiment provides additional mechanism about L-His and ImP. L-His co-treatment preserved viability (Figure S2A) and Glucose-Stimulated Insulin Secretion (GSIS) in the presence of ImP (Figure S2B) and restored p-Akt, p-mTOR, PDX-1, NeuroD1, and MafA relative to the TAC+ImP condition (Figure S2C). These results indicate that, within β-cells, the protective L-His response can predominate over the ImP-associated inhibitory phenotype via Akt/mTOR signaling pathway and the stimulating the insulin regulatory proteins.

Previous studies have linked L-His to islet function.^16^^,^^17^^,^^18^ Targeted amino acid profiling further showed distinct fecal and serum amino acid signatures after TAC exposure, with histidine increased in both compartments (Figures 3C and 3D). To determine whether this increase was associated with microbial histidine biosynthesis, we quantified two histidine-producing bacteria and key biosynthetic genes. TAC-treated animals exhibited higher abundances of *Streptococcus bovis* and *Selenomonas ruminantium* (Figures 3E and 3F), together with increased copy numbers of *hisG* and *hisD* (Figures 3G and 3H). Targeted measurements confirmed elevated fecal and serum L-His concentrations and increased ImP abundance in the TAC group (Figures 3I–3K). To test whether the altered microbiota was sufficient to transmit this metabolic phenotype, antibiotic-treated recipients underwent Fecal Microbiota Transplantation (FMT). Compared with the ABX and FMT groups, recipients of microbiota from TAC-treated donors (TAC-FMT) showed the greatest increases in *S. bovis*, *S. ruminantium*, *hisG*, *hisD*, fecal L-His, serum L-His, and ImP (Figure 3L–3R). These effects were lost when the TAC-associated fecal microbiota was inactivated before transplantation. Together, these findings indicate that viable TAC-associated gut microbiota contribute directly to enhanced microbial histidine biosynthesis. Longitudinal profiling further defined the timing of this response. Fecal L-His remained similar between groups at the beginning of TAC exposure but became significantly elevated in TAC-treated animals from day 14 (Figure S3A). Serum L-His showed a later increase, reaching a significant increase at day 30 (Figure S3B). This could prove that L-His is a response to damage rather than a side effect of TAC exposure. Figure 6B shows interim blood glucose and L-His levels (Figure S3) at day 7, 14, 21, and 30. Meanwhile, in the microbiota-depletion experiment, fecal 16S rRNA copy number was markedly reduced in all ABX-treated groups, confirming effective depletion (Figure S4A). ABX treatment was accompanied by lower relative L-His abundance in serum, intestinal tissue, and pancreatic tissue. TAC+ABX further maintained low L-His levels, whereas exogenous L-His supplementation restored L-His abundance across these compartments despite the persistently depleted bacterial load (Figures S4B–S4D). These data also indicate that L-His levels begin to rise after the onset of significant hyperglycemia, and it is the temporal dynamics of gut-pancreas feedback loop.

**Figure 6:**
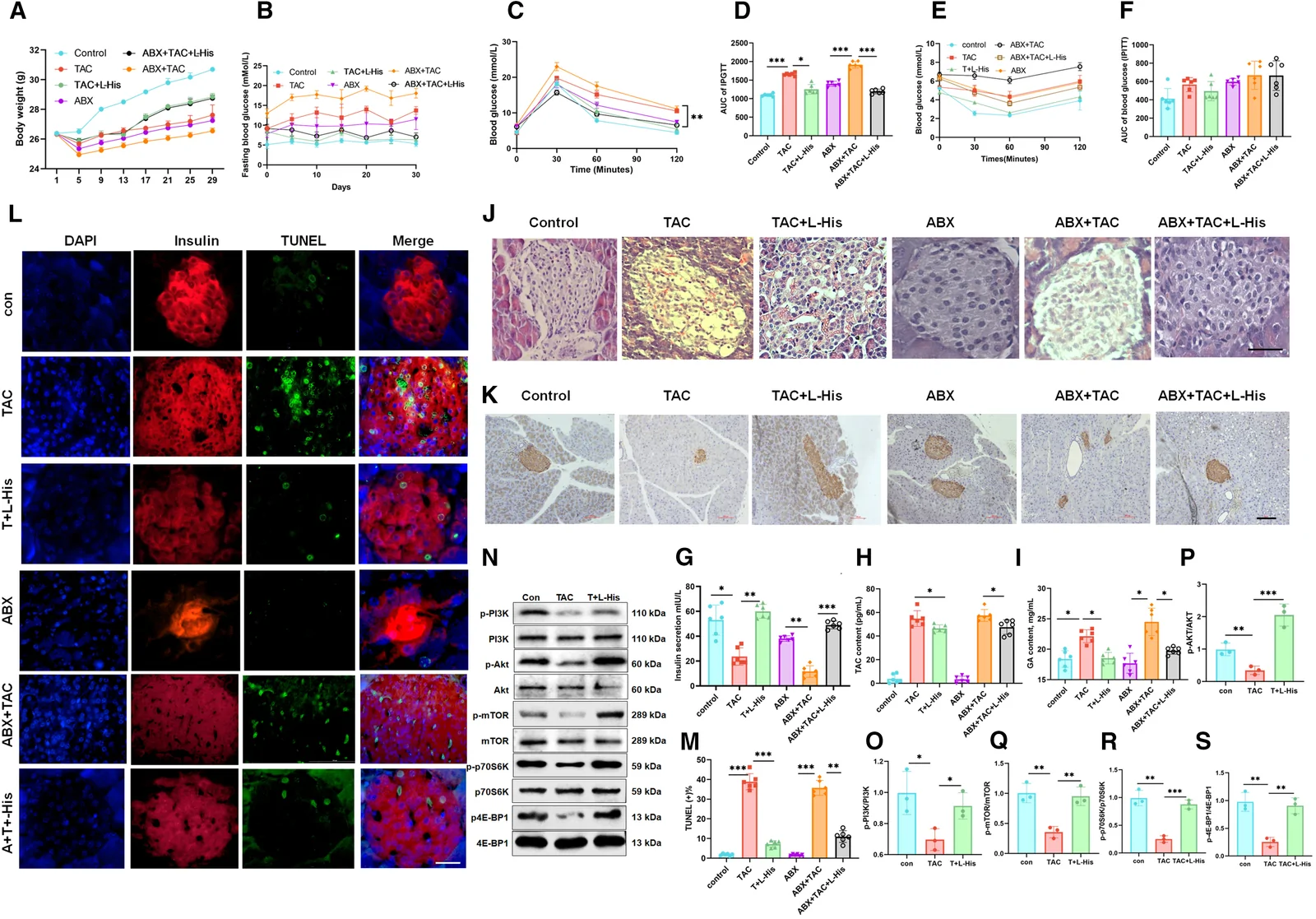
L-His alleviates TAC-induced islet injury *in vivo* (A and B) (A) Body weight and (B) fasting blood glucose in control, TAC, TAC+L-His, ABX, ABX+TAC, and ABX+TAC+L-His groups. (C and D) (C) Intraperitoneal glucose tolerance test (IPGTT) and (D) area under the curve (AUC) of IPGTT. (E and F) (E) Intraperitoneal insulin tolerance test (IPITT) and (F) AUC of IPITT. (G) Serum insulin concentration. (H) Serum TAC concentration. (I) Glycated albumin (GA) concentration. (J) Representative hematoxylin and eosin (H&E) staining of pancreatic islets. (K) Representative insulin immunohistochemistry (IHC). (L) Representative immunofluorescence for DAPI (blue), insulin (red), TUNEL (green), and merged images. (M) Quantification of TUNEL-positive cells. (N) Immunoblotting of phosphorylated and total PI3K, Akt, mTOR, p70S6K, and 4E-BP1 in islets from control, TAC, and TAC+L-His mice. (O–S) Densitometric ratios of p-PI3K/PI3K (O), p-Akt/Akt (P), p-mTOR/mTOR (Q), p-p70S6K/p70S6K (R), and p-4E-BP1/4E-BP1 (S). Scale bars represent 50 μm. ABX, antibiotic-mediated gut microbiota depletion. Data are presented as mean ± SD. Statistical tests for quantitative comparisons denoted by asterisks: two-way ANOVA was used for (A), (B), (C), and (E); one-way ANOVA was used for the remaining quantitative comparisons. Asterisks denote ∗*p* < 0.05, ∗∗*p* < 0.01, and ∗∗∗*p* < 0.001. *n* = 6 mice per group.

### L-His protects islet β-cells from TAC-mediated dysfunction *in vitro*

To further validate our hypothesis, we performed *in vitro* studies using MIN-6 cells. First, we observed whether L-His have any toxicity on MIN-6 cells, and found that there are no morphology changes with different dose of L-His, ranging from 0–10 mM (Figure 4A), and statistics of viable cells also reached to almost 100% with the higher concentration of L-His (10 mM, Figure 4B). As expected, TAC could induce the MIN-6 cell death in a dose-dependent manner (Figure 4C). However, the TAC induced cell death could be reversed by the addition of L-His (Figure 4D). Further the supplement of L-His could rescue the cells from TAC-induced death in a dose-dependent manner (Figure 4E). Subsequent flow cytometry analysis demonstrated that TAC-treated cells undergo apoptosis, whereas the TAC cytotoxicity could be abolished by L-His treatment (Figures 4F and 4G). Next, we questioned whether the survived cells were functionable. Glucose stimulated insulin secretion was analyzed. As shown in Figure 4H, the TAC treatment lead to impaired insulin secretion in response to glucose stimulation, and it could be reversed by the supplement of L-His. To support that L-His contribute the islet protection, we supplemented HDC (histidine decarboxylase), to abolish the function of L-His. It is obvious that with the triple therapy (HDC +TAC+L-His), the cell viability was decreased (Figure 4I), and the glucose response was impaired (Figure 4J). Although it is known that TAC impairs islet function via the phosphoinositide 3-kinase (PI3K)/protein kinase B (Akt)/mechanistic target of rapamycin (mTOR) pathway,^5^^,^^6^ there is no study whether the gut microbiota metabolite, L-His, could protect the islet cell with this signaling pathway. Then we performed the immunoblotting of phosphor-PI3K, total PI3K, phosphor-Akt, total Akt, phosphor-mTOR, and total mTOR. As seen in Figure 4I, L-His supplement could activate the PI3K/Akt/mTOR pathway by phosphorylation of PI3K, Akt, and mTOR. This could be further validated by the gray scale quantification of p-PI3K versus total PI3K (Figure 4J), p-Akt versus total Akt (Figure 4K), p-mTOR versus total mTOR (Figure 4L). To further substantiate that PI3K/Akt/mTOR pathway plays a crucial role in L-His induced islet protective function, the PI3K inhibitor, wortmannin, was supplemented, and the activation of this pathway was inhibited (Figure 4O), accompanied with decreased cell viability (Figure 4P). Quantification of gray scales of activated PI3K versus total PI3K (Figure 4Q), activated Akt versus total Akt (Figure 4R), activated mTOR versus total mTOR (Figure 4S) validated our observation in Figure 4O. Lastly, the L-His stimulated insulin response was abolished by wortmannin as well (Figure 4T), suggesting that L-His restores islet function via the PI3K/Akt/mTOR pathway.

Next, we questioned what caused the increased insulin response of L-His-treated cells. From transcription levels, we performed semi-quantitative PCR of “pancreatic duodenal homeobox-1 (PDX-1), neuronal differentiation 1 (NueroD1), and MAF bZIP transcription factor A (MafA)”, which are the upstream genes of insulin. As documented that TAC leads to the reduction of nuclear MafA(5, 24), we observed there were decreased transcription of Pdx-1 (Figure 5A), NeuroD1 (Figure 5B), and MafA (Figure 5C) with the treatment of TAC. However, the transcription of these insulin upstream genes could be upregulated with the L-His treatment. At the meantime, the protein levels of these three proteins were also increased with the addition of L-His (Figure 5D). Furthermore, we performed cell staining of PDX-1 (Figure 5E), NeuroD1 (Figure 5G), MafA (Figure 5I), and insulin (Figure 5K). It is obvious, that TAC-treated cells had less PDX-1 (Figure 5F), NeuroD1 (Figure 5H), MafA (Figure 5J), and insulin (Figure 5L) fluorescent intensity, whereas L-His could increase the number of positive cells.

### L-his protects islet β-cells from TAC-mediated dysfunction *in vivo*

To validate our *in vitro* findings and to determine whether an intact gut microbiota was required for the protective action of L-His, we expanded the *in vivo* experiment to six groups: control, TAC, TAC+L-His, ABX, ABX+TAC, and ABX+TAC+L-His. TAC reduced bodyweight gain and increased fasting blood glucose, whereas L-His increase the body weight and maintain the mice in normal glycemia. After microbiota depletion, ABX+TAC mice showed hyperglycemia and reduced weight gain, while L-His supplementation in the ABX+TAC group partially restored body weight and markedly lowered fasting glucose (Figures 6A and 6B). Intraperitoneal glucose tolerance test (IPGTT) showed impaired glucose tolerance after TAC exposure and a further adverse trend in ABX+TAC mice. L-His reduced the glucose excursion and AUC in both TAC-treated settings (Figures 6C and 6D). Intraperitoneal insulin tolerance test (IPITT) was also performed across all six groups (Figures 6E and 6F). To determine the temporal relationship between TAC-induced hyperglycemia and L-His elevation, we performed analysis of fecal and serum L-His levels during TAC exposure. Control animals maintained relatively constant L-His concentrations throughout the experimental period. In contrast, TAC-treated animals showed a gradual increase in fecal L-His abundance (Figure S3A), and serum L-His levels exhibited a delayed but consistent elevation, indicating increased systemic availability of microbiota-derived L-His (Figure S3B). At the end of the experiment, TAC reduced serum insulin and increased glycated albumin, and these abnormalities tended to be exacerbated in the ABX+TAC group. L-His restored insulin concentrations and lowered glycated albumin in both TAC and ABX+TAC mice (Figures 6G and 6I). Serum TAC concentrations remained detectable in the TAC-containing groups and were modestly lower after L-His supplementation (Figure 6H). Histological examination showed disrupted islet architecture after TAC treatment, with prominent injury in ABX+TAC mice, whereas L-His improved islet morphology in both microbiota-intact and microbiota-depleted animals (Figure 6J). Insulin immunohistochemistry showed reduced islet insulin staining with smaller islet morphology after TAC and ABX+TAC treatment, which was restored by L-His (Figure 6K). Consistently, insulin/TUNEL immunofluorescence demonstrated a marked increase in TUNEL-positive islet cells after TAC or ABX+TAC treatment and a substantial reduction of dead cell after L-His supplementation (Figures 6L and 6M). These findings indicate that L-His can restore β-cell viability and function. In isolated islets from these mice, TAC suppressed phosphorylation of PI3K, Akt, mTOR, and the mTOR downstream effectors p70S6K and 4E-BP1, whereas L-His restored activation of PI3K/Akt/mTOR/p70S6K signaling pathway (Figures 6N–6S).

Subsequently, we determined whether the insulin upstream genes were upregulated *in vivo*. Firstly, we performed real-time PCR of *PDX-1*, *NueroD1*, and *MafA*. As expected, we observed there were decreased transcription of *Pdx-1* (Figure 7A), *NeuroD1* (Figure 7B) and *MafA* (Figure 7C) with the treatment of TAC, and it could be reversed by the L-His treatmenbt. Secondly, followed by islet isolation of the mice from the three groups, the protein levels of these three proteins were also increased with the addition of L-His (Figure 7D). Thirdly, we performed immunofluorescent staining of the pancreas for PDX-1 (Figure 7E), NeuroD1 (Figure 7G), MafA (Figure 7I), and insulin (Figure 7K). These series of pictures demonstrated that L-His treatment could increase the PDX-1, NeuroD1, and MafA expression in the nucleus of the islet cells, and thereby increase insulin expression in the cytoplasm of these islets. Lastly, the fluorescent intensity comparing the three groups demonstrated that L-His treatment increases the fluorescent intensity of PDX-1 (Figure 7F), NeuroD1 (Figure 7H), MafA (Figure 7J), and insulin (Figure 7L). Taken together, L-His not only maintenance the morphology and viability of the islet β-cells via the PI3K/Akt/mTOR pathway but also stimulate its function by regulating the upstream genes of insulin.

**Figure 7:**
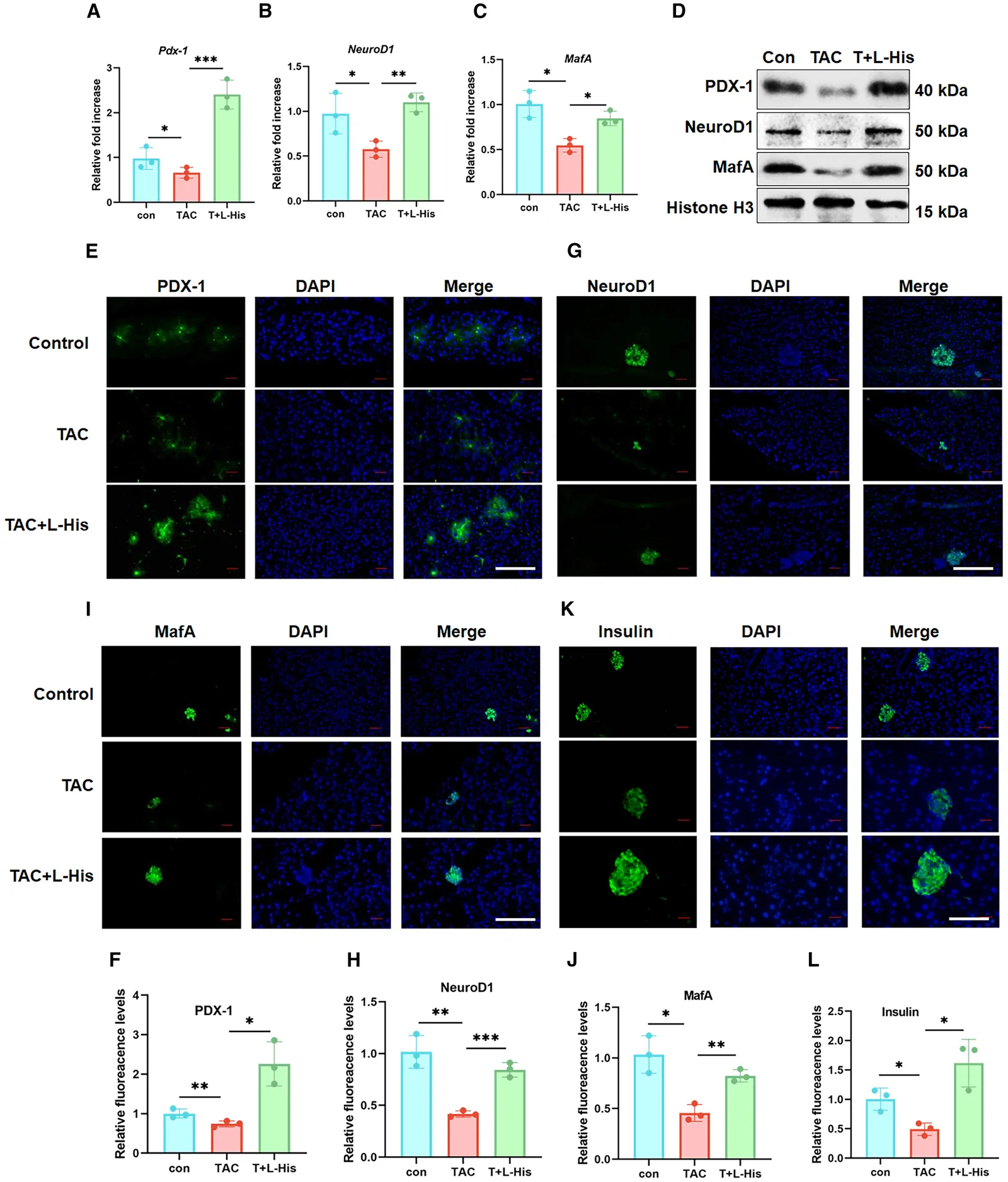
L-His stimulate the islet function of TAC treated mice Real-time PCR of Pdx-1 (A), NeuroD1 (B) and MafA (C). Western blotting of PDX-1, NeuroD1, and MafA (D) from islets isolated from the control mice, TAC-treated mice and TAC+L-His treated mice. Immunofluorescent staining of PDX-1 (E), Neuro D1 (G), MafA (I), and insulin (K) of the pancreas harvested from the control mice, TAC-treated mice and TAC+L-His treated mice. Statistics of fluorescent intensity of PDX-1 (F), Neuro D1 (H), MafA (J), and insulin (L). Scale bars represent 100 μm. All data are presented as mean ± SD. Statistical test for quantitative comparisons denoted by asterisks: one-way ANOVA. Asterisks denote ∗*p* < 0.05, ∗∗*p* < 0.01, and ∗∗∗*p* < 0.001. *n* = 6 mice per group.

## Discussion

Solid organ transplant recipients require lifelong immunosuppressive therapy to maintain graft function and prevent rejection.^16^ However, long-term exposure to TAC is associated with multiple complications, among which diabetes and islet dysfunction is one of the major risk factors affecting prognosis and survival.^17^ TAC is a key contributor to islet dysfunction, and previous studies have shown that it can impair insulin secretion^6^^,^^19^ and reduce insulin sensitivity.^3^^,^^20^ Therefore, it is of crucial importance to find a solution to alleviate the TAC mediated islet damage.

Most L-His is obtained from dietary protein digestion,^21^ although intestinal microorganisms can also synthesize it.^22^ TAC caused broad microbial remodeling, with reduced Simpson and Shannon diversity, separation from controls in PCoA, and changes at the phylum and genus levels. These alterations were accompanied by distinct fecal and serum metabolomic profiles, while L-His and D-(-)-mannitol correlated with TAC-responsive taxa. Consistent with our previous findings in transplant recipients with diabetes,^11^ the data support TAC-induced dysbiosis as an important component of the gut-pancreas axis. Figure 3 provides functional evidence that viable TAC-associated microbiota contribute to histidine metabolism. TAC increased *S. bovis*, *S. ruminantium*, hisG, and hisD, together with fecal and serum L-His and ImP. Microbiota from TAC-treated donors reproduced these changes, whereas inactivated donor microbiota did not, moving the interpretation beyond correlation. The increased L-His may represent an adaptive response because it protected β-cells through PI3K/Akt/mTOR signaling and the PDX-1/NeuroD1/MafA network. However, the parallel rise in ImP suggests potentially different metabolic effects. Longitudinal sampling, isotope tracing, and defined microbial consortia are needed to clarify causality and species-related differences.^23^

In the metabolites table, the D-Mannitol, and (+/-)12(13)-DiHOME were increased much more than L-His. For the latter one, there is no commercial product that we can purchase, so it cannot be investigated at current stage. We purchased D-Mannitol and performed cell viability assay by CCK-8 assay (Figure S1A), cell apoptosis quantification by flow cytometry of Annexin V-PI (Figures S1B and S1C), and GSIS studies (Figure S1D). As expected that D-Mannitol had similar function as L-histidine. However, because mannitol has been well documented to alleviate diabetes in multiple literatures, from glucose uptake, intestinal glucose absorption, intestinal permeability, specific gut microbiota, and diabetes patients since 1950’s,^12^^,^^13^^,^^14^^,^^24^ the side effects of mannitol are also well investigated.^15^ However, it is not clinically applied. Even more, mannitol is contraindicated in diabetic patients with renal insufficiency. Because mannitol is excreted via the kidneys, it can readily lead to cumulative hyperosmolarity, electrolyte disturbances, and acute kidney injury.^15^ For diabetic patients with concurrent dehydration, care must be taken to avoid exacerbating the hyperosmolar state. Its use is specifically contraindicated in cases of hyperosmolar dehydration. Therefore, we only compared the basic cell survival and function of L-His and mannitol in the Table S1 and choose L-His for subsequent studies. Moreover, the temporal separation between hyperglycemia and L-His elevation suggests that L-His is not a primary mediator of TAC toxicity but rather an inducible protective metabolite. The intestine may function as a metabolic sensor that responds to β-cell injury by increasing microbial histidine production, thereby activating a gut-pancreas compensatory axis. The supplementary D-(-)-mannitol comparison provides an important specificity control. Although D-(-)-mannitol, like L-His, improved viability and limited apoptosis in TAC-exposed MIN-6 cells, it failed to restore the glucose-stimulated insulin response. This suggests that simple preservation of cell survival is not sufficient to recover β-cell function and supports the conclusion that L-His might be better than D-mannitol.

TAC, a first-line calcineurin inhibitor for clinical organ transplantation, frequently induces post-transplantation diabetes mellitus (PTDM) as a dose-limiting adverse outcome, predominantly originated from direct toxic damage to pancreatic β-cells. Consistent with prior clinical and cellular observations, our data robustly recapitulated TAC’s diabetogenic toxicity. TAC disrupted β-cell morphology, suppressed cell viability in a concentration-dependent fashion, triggered extensive apoptotic cell death, and severely damaged GSIS under both basal and stimulatory glucose concentrations. These functional and phenotypic defects collectively explain the impaired glycemic homeostasis seen in transplant recipients receiving long-term TAC therapy, highlighting an urgent unmet need for adjuvant agents that can mitigate TAC-induced β-cell injury without compromising immunosuppressive efficacy. In our study, we found that L-His, an essential proteinogenic amino acid with documented antioxidant and cytoprotective properties across multiple somatic cell types, emerged as an effective protective mediator against TAC-mediated β-cell damage in this study. We first confirmed that physiological high dose of L-His exerted no cytotoxicity in β-cells, confirming its safety on β-cells. Moreover, co-supplementation with L-His substantially reversed the detrimental phenotypes induced by TAC. Morphological observation and flow cytometric apoptosis profiling demonstrated that L-His normalized cellular structure and reduced the fraction of Annexin V-positive apoptotic cells, whereas functional GSIS assays validated that L-His rescued insulin secretory capacity compromised by TAC exposure. These functional rescue results position L-His as a promising candidate to counteract the dual hallmarks of TAC β-cell toxicity, cell death, and secretory dysfunction. Mechanistically, our signaling pathway dissection pinpointed the PI3K/Akt/mTOR cascade^6^^,^^19^ as the core molecular axis underlying L-His’s protective action. TAC profoundly suppressed the phosphorylation of PI3K, Akt, and mTOR without altering total protein expression, which dampens this canonical pro-survival signaling cascade and tilts the cellular balance toward apoptosis and secretory failure. Co-treatment with L-His fully restored phosphorylated PI3K, Akt, and mTOR levels, reinstating pathway activity. Critically, pharmacological blockade of PI3K via wortmannin completely abolished L-His’s beneficial effects on cell viability and insulin secretion, alongside ablating L-His-triggered phosphorylation of PI3K/Akt/mTOR. This loss-of-function evidence definitively establishes that L-His exerts its anti-apoptotic and function-preserving effects in TAC-damaged β-cells strictly through activation of PI3K/Akt/mTOR signaling. The PI3K/Akt/mTOR axis is well established to sustain β-cell survival, proliferation, and insulin granule exocytosis. TAC-mediated inhibition of this pathway therefore represents a central driver of its diabetogenicity, while L-His acts to reverse this inhibitory signal.

The systemic metabolic, gut microbiome, and pancreatic cellular data presented across all figures converge to build two cascades driving TAC-induced PTDM. The first one consists of extra-pancreatic gut microbial dysfunction triggered by TAC. Immunosuppressant exposure reduces gut microbial alpha diversity, shifts Firmicutes/Bacteroidetes balance, depletes SCFA-producing commensals, enriches pro-inflammatory histidine-metabolizing taxa, and elevates luminal imidazole propionate. The second one comprises direct intrinsic pancreatic β-cell toxicity of TAC. Intracellular TAC accumulation suppresses PI3K/Akt/mTOR phosphorylation, activates mitochondrial apoptotic pathways, downregulates PDX-1/NeuroD1/MafA(24, 30) transcription factor networks to drive β-cell dedifferentiation, and abolishes glucose-dependent insulin granule exocytosis. When combined, peripheral insulin resistance and impaired endogenous insulin secretion create an irreversible glycemic defect that manifests clinically as PTDM. Our *in vitro* and *in vivo* pancreatic validation experiments confirmed the cytotoxicity of TAC on β-cells, whereas the gut microbiome data contextualized these pancreatic defects within a systemic metabolic framework. TAC monotherapy reduced β-cell viability, elevated Annexin V/PI apoptotic populations, impaired glucose tolerance, suppressed nuclear transcription and translation of PDX-1, NeuroD1, MafA, and insulin protein expression,^21^^,^^22^^,^^25^^,^^26^^,^^27^^,^^28^ and induced islet architectural damage and TUNEL-positive β-cell in murine pancreatic tissue. Mechanistic western blot and pharmacological inhibition assays established that TAC suppresses the PI3K/Akt/mTOR pro-survival cascade, the core signaling node linking both gut-derived peripheral insulin resistance and pancreatic β-cell survival. The PI3K/Akt pathway serves as a shared therapeutic target for L-His. In peripheral tissues, L-His counteracts imidazole propionate-mediated Akt inhibition to restore insulin sensitivity; in pancreatic β-cells, L-His reverses TAC-driven PI3K/Akt/mTOR suppression to block apoptosis and preserve insulin secretory function. Pharmacological blockade of PI3K with wortmannin completely ablated all L-His-mediated protective phenotypes, including improved cell viability, rescued GSIS, restored β-cell transcription factor expression, and normalized systemic glucose tolerance,^18^ confirming PI3K/Akt/mTOR as the mandatory downstream effector of L-His’s dual gut-pancreas protective activity. Taken together, our findings suggest that L-His improves TAC-induced islet dysfunction through coordinated regulation of upstream signaling pathways and downstream transcriptional programs.

Although our study demonstrates that gut microbiota-derived L-histidine protects pancreatic β-cell function against tacrolimus-induced injury through the gut-pancreas axis, the complexity of PTDM in clinical transplantation should be further considered. The current model was established using healthy rodents treated with tacrolimus rather than a transplantation model, and therefore primarily reflects the direct metabolic toxicity of tacrolimus on pancreatic islets. In clinical settings, however, PTDM develops within a complex immunometabolic environment involving both graft-related immune responses and multiple immunosuppressive therapies.

Following transplantation, alloimmune activation induced by the graft may promote systemic inflammatory responses through cytokine and chemokine release, which can influence intestinal barrier function, microbial community composition, and microbial metabolite production. These immune-mediated alterations may subsequently modify the availability and biological activity of gut-derived metabolites, thereby affecting pancreatic β-cell stress responses and insulin secretion. In addition, transplant recipients usually receive combination immunosuppressive regimens, including corticosteroids, mycophenolate mofetil, and mTOR inhibitors, which may independently reshape the gut microbiota, alter intestinal metabolic pathways, and contribute to glucose dysregulation. Therefore, the L-histidine-mediated gut-pancreas axis identified in our study should be considered as one component of the broader regulatory network underlying PTDM, in which graft-induced immune responses and concomitant medications may serve as important modulators. Future studies using clinically relevant transplantation models with standard immunosuppressive combinations will be required to determine how these factors interact with microbiota-derived metabolites and influence β-cell function after transplantation.

Furthermore, in this study, the increased abundance of L-histidine after tacrolimus exposure suggests a potential endogenous adaptive response to TAC-induced metabolic stress. However, we acknowledge that L-histidine levels were assessed only at the end of the 30-day experimental period, which limits our ability to determine the precise temporal relationship between L-histidine elevation and the development of hyperglycemia or β-cell dysfunction. Therefore, although our findings support the possibility that L-histidine represents a compensatory response involved in the gut-pancreas axis, the dynamic changes of L-histidine during the progression of TAC-induced metabolic impairment remain to be determined. Future longitudinal studies with serial measurements of circulating and intestinal L-histidine levels, together with sequential assessment of glucose metabolism and β-cell function, will be necessary to establish whether L-histidine elevation precedes, parallels, or follows TAC-induced metabolic injury and to further clarify its role as an adaptive metabolic response.

In addition, only 6-week-old male Sprague-Dawley rats were included, which may limit the generalizability of our findings given that sex differences can markedly influence gut microbiota composition and metabolic profiles. Further studies should explore the potential impact of sex on disease progression and therapeutic responses. Male recipients are tending to have PTDM with TAC treatment.^29^ Studies have found a higher proportion of new-onset diabetes cases among male transplant recipients; tacrolimus dosage is an independent risk factor for the condition in men, whereas preoperative hypertension and the cumulative postoperative dose of glucocorticoids are key triggers for the disease in women.^30^ Significant gender-based differences exist in tacrolimus use among kidney transplant recipients. Women require higher dosages due to greater CYP3A4 activity and faster drug clearance, yet face a higher risk of rejection and adverse neurological or gastrointestinal effects. In contrast men are more prone to drug accumulation and increased pancreatic β-cell toxicity, leading to significantly elevated risks of PTDM, long-term cardiovascular complications, and graft injury.^31^ Therefore, in this study, we use male rodents for *in vivo* studies.

However, sex is an important biological factor that may influence gut microbiota composition and host-microbiome interactions. In the present study, male rodents were selected to establish the tacrolimus-induced diabetes model, which is clinically relevant because male sex has been identified as an important risk factor associated with the development of PTDM. Therefore, our findings may better reflect the microbiota and metabolic alterations occurring in the predominant high-risk male PTDM population.

Nevertheless, PTDM also occurs in female transplant recipients, and sex-dependent regulation of gut microbiota should be considered when interpreting the translational relevance of our findings. Previous studies have demonstrated that sex hormones, including estrogen and androgen, can modulate gut microbial composition and microbial-derived metabolic pathways in a sex-specific manner.^32^ Notably, Lactobacillus species, one of the bacterial genera altered in our study, play important roles in female health, particularly in maintaining vaginal microbiota homeostasis and reproductive health.^33^^,^^34^^,^^35^^,^^36^ These studies have highlighted that hormonal and anatomical differences may influence Lactobacillus colonization and functional activity.^33^^,^^34^^,^^35^^,^^36^ Similarly, Collinsella, another genus enriched after tacrolimus exposure in our study. It has been associated with host metabolic status and inflammatory regulation, and its abundance may also be affected by sex-related physiological factors.^23^^,^^37^

Therefore, although the Lactobacillus- and Collinsella-associated microbial alterations identified in this study provide important insights into the gut-pancreas axis in TAC-induced metabolic dysfunction, their magnitude and functional consequences may differ between male and female transplant recipients. Future studies incorporating female transplantation models and sex-stratified clinical cohorts will be necessary to determine whether the L-histidine-mediated microbiota-metabolite pathway exhibits sex-dependent characteristics.

Another thing needs to address is the potential influence of ImP, a microbial secondary metabolite of L-His. Since ImP has been reported to impair insulin signaling and glucose metabolism,^38^ we further performed three studies to observe whether the deleterious function of ImP could be counteracted by L-His. By three independent experiments (cell viability, GSIS, and immunoblotting of insulin related regulators, Figures S1–S3), we found that the function of L-His could override that of the ImP. One discrepancy of our study with previous publication is that they found that ImP impair insulin signaling by the activation of mTOR in liver. However, maybe due to organ differences and different cell function between hepatocyte and islet β-cell, in our study, we found that mTOR is inhibited by ImP, and activation of mTOR by L-His could stimulate the insulin secretion.

Moreover, the 35 metabolites increased in mice were unchanged in our previous human study, maybe due to cross-species conservation. The metabolism of ImP, an L-Histidine-derived metabolite, is regulated in completely differently in human and mice.^39^ They are even in opposite ways by the gut microbiota of these two species, further validate the species-specific metabolic regulation.^39^ But this is a potential limitation of L-Histidine clinical translation.

In summary, this study indicates that L-His, a nutrient derived from food, can ameliorate TAC-induced islet dysfunction and glucose metabolic disorder. Its protective effects are associated with modulation of PI3K/Akt/mTOR signaling and restoration of β-cell functional transcription factors (PDX-1/NeuroD1/MafA). These findings provide the potential value of L-His as an important therapeutic strategy for transplant recipient receiving TAC treatment.

### Limitations of the study

This study has several limitations. First, the *in vivo* model used healthy rodents treated with tacrolimus rather than a transplantation model. Clinical PTDM develops in the context of graft-related immune responses and combination immunosuppressive regimens, including corticosteroids, mycophenolate mofetil, and mTOR inhibitors, which may independently affect the gut microbiota, metabolism, and β-cell function. Studies using clinically relevant transplantation models will be needed to determine how these factors interact with the L-His-associated response.

Second, although longitudinal measurements were performed, the temporal and causal relationship between L-His elevation and TAC-associated hyperglycemia remains incompletely resolved. Defined microbial consortia, isotope tracing, and more frequent serial sampling would help establish whether altered microbial histidine synthesis precedes, parallels, or follows β-cell dysfunction. ImP, a microbial metabolite of histidine, may also have context-dependent effects that require further investigation.

Third, the study primarily used 6-week-old male Sprague-Dawley rats. Female animals were not included in those experiments, limiting assessment of sex-dependent differences in gut microbial composition, histidine metabolism, TAC pharmacology, and β-cell responses. Sex-stratified animal studies and clinical cohorts will be needed to evaluate the generalizability of the findings.

Finally, the manuscript notes differences between the rodent metabolomic findings and a previous human study, as well as species-specific regulation of ImP metabolism. These cross-species differences limit direct extrapolation to transplant recipients and support the need for validation in human samples and prospective clinical studies.

## Resource availability

### Lead contact

Requests for further information and resources should be directed to and will be fulfilled by the lead contact, Liang Wei (weiliang05@tkhealthcare.com).

### Materials availability

This study did not generate new unique plasmids, mouse lines, or patented reagents beyond commercially available chemicals (tacrolimus, L-histidine, antibiotics, and standard assay kits). Unique/stable reagents used in this study are available from the lead contact with a completed materials transfer agreement if there is potential for commercial application.

### Data and code availability

• 16S rRNA sequencing data have been deposited at NCBI SRA as PRJNA1507484 and are publicly available as of the date of publication.
• Metabolomics data have been deposited at MetaboLights with accession MTBLS15601 and are publicly available as of the date of publication.
• This paper does not report original code.
• Any additional information required to reanalyze the data reported in this paper is available from the lead contact upon request.

## Acknowledgments

This study was sponsored by the 10.13039/501100001809National Science Foundation of China (82570932 and 81802504), National Science and Technology Major Project-Noncommunicable Chronic Diseases (2025ZD0552100 and 2025ZD0552104), National Science and Technology-Key Research and Development Program (2026YFF1500400), Sichuan Science and Technology Program (2024NSFSC0589), and 10.13039/501100019014Chengdu Science and Technology Program (2026-YF05-00930-SN).

## Author contributions

Conceptualization, Y.W. and L.W.; methodology, Z.G., Q.L., and D.W.; investigation, H.Y.; formal analysis, H.W. and J.B.; writing – original draft, Z.G.; writing – review and editing, Y.W. and L.W.; funding acquisition, Y.W. and L.W.; resources, Z.G. and J.B.; supervision, Y.W. and L.W.

## Declaration of interests

The authors declare no competing interests.

## Declaration of generative AI and AI-assisted technologies in the manuscript preparation process

During the preparation of this work, the author(s) used ChatGPT in order to reduce the abstract to 150 words. After using this tool, the author(s) reviewed and edited the content as needed and take(s) full responsibility for the content of the publication.

## STAR★Methods

### Key resources table

**Table:** 

REAGENT or RESOURCE	SOURCE	IDENTIFIER
**Antibodies**		

p-PI3K antibody	MCE	RRID：HY-P81146
PI3K antibody	HUABIO	RRID：HA722474
*p*-Akt antibody	HUABIO	RRID：ET1607-73
Akt antibody	HUABIO	RRID：ET1609-51
*p*-mTOR antibody	HUABIO	RRID：HA600094
mTOR antibody	HUABIO	RRID：ET1608-5
PDX-1 antibody	HUABIO	RRID：ET1705-99
NeuroD1 antibody	HUABIO	RRID：ET1703-73
p70S6K antibody	HUABIO	RRID：HA722520
eIF4EBP1 antibody	HUABIO	RRID：ET1701-83
β-actin antibody	HUABIO	RRID：EM21002
p-p70S6K T389 antibody	Abcam	Cat# ab2571; RRID:AB_303169
*p*-eIF4EBP1 T37/46 antibody	Abcam	Cat# ab278686; RRID:AB_3741564
MafA antibody	Abcam	RRID：ab324869
Insulin antibody	HUABIO	RRID：ET1601-12
Histone H3	HUABIO	RRID：ET-1701–64
MAFA antibody for immunofluorescence	Thermo Fisher	Catr# IHC-00352; RRID:AB_1279486
PDX-1 antibody for immunofluorescence	HUABIO	RRID：ET1705-99
HRP-conjugated goat anti-rabbit IgG antibody	HUABIO	RRID：HA1001
Fluorophore-conjugated secondary antibody	HUABIO	RRID：HA1004, HA1102

**Chemicals, peptides, and recombinant proteins**		

Tacrolimus TAC FK506	Shanghai Yuanye Bio-Technology Co., Ltd.	S31618
Olive oil	Shanghai Macklin Biochemical Technology Co., Ltd.	767419
L-Histidine	Sigma-Aldrich	H6034
D-glucose	Solarbio	G8150
D-mannitol	Beyotime	ST2367
Imidazole propionate ImP	Sigma-Aldrich	900575
Wortmannin	MCE	SL-2052
Histidine decarboxylase HDC	LMAI Bio	IC8Q476Ra
Ampicillin	Beyotime	ST007
Neomycin	Beyotime	ST2533
Metronidazole	Beyotime	Y170126
Vancomycin	Beyotime	ST2807

**Critical commercial assays**		

QIAamp DNA Stool Mini Kit	QIAGEN	51504
Rat insulin assay kit	Sigma-Aldrich	RAB0904
FK506 tacrolimus assay kit	Guangzhou Aoruida Biotech	ARD11300
Glycated serum albumin assay kit	Krishgen Biosystems	KLM1353
Mouse insulin ELISA kit	Saipei Ltd.	SP14098
TUNEL staining kit	Beyotime	C1089
Reverse transcription kit	Vazyme	R302-01
Annexin V-FITC PI apoptosis assay reagents	Yeasen	40302ES60

**Deposited data**		

16S rRNA sequencing data	NCBI SRA	PRJNA1507484
Metabolomics data	MetaboLights	REQ20260807222465
Unedited uncropped western-blot source images with molecular weight standards	supplemental source-data file	N/A
Unedited microscopy source images	supplemental source-data file	N/A

**Experimental models Cell lines**		

MIN-6 mouse pancreatic β-cell line	Merck	SCC623

**Experimental models Organisms strains**		

Sprague-Dawley rats male 6 weeks 200–220 g	Chengdu Dossy Experimental Animals Co., Ltd.	N/A
C57BL/6J mice male 8 weeks 20–25 g	Chengdu Dossy Experimental Animals Co., Ltd.	N/A

**Oligonucleotides**		

Streptococcus bovis qPCR primers	This paper	F TTCCTAGAGATAGGAAGTTTCTTCGG; R ATGATGGCAACTAACAATAGGGGT
Selenomonas ruminantium qPCR primers	This paper	F CAATAAGCATTCCGCCTGGG; R TTCACTCAATGTCAAGCCCTGG
hisG qPCR primers	This paper	F ATATTCTGAAATGAGCTGTTGACAA; R TACTGCCGCCAGGCCAAATTC
hisD qPCR primers	This paper	F GTGATCCATATGAGCTTTAACACAA; R AGAATTCACGGTGCTCATGCTTGC
PDX-1 qPCR primers	This paper	F AGCAGTACTACGCGGCCACA; R GCACTTCGTATGGGGAGATG
NeuroD1 qPCR primers	This paper	F CCTCAAGAAAGCGACGAAGAA; R CTTGTTTCCATCATGCTCTCG
MafA qPCR primers	This paper	F GCTCTGGAGTTGGCACTTCT; R CTTCAGCAAGGAGGAGGTCA
18S qPCR primers	This paper	F GTAACCCGTTGAACCCCATT; R CCATCCAATCGGTAGTAGCG
16S V4 primer pair 515F 806R	N/A	F GTGCCAGCMGCCGCGGTAA; R GGACTACHVGGGTWTCTAAT

**Software and algorithms**		

fastp	fastp	v0.23.1
QIIME2	QIIME2	v1.9.1
Compound Discoverer	Thermo Scientific	v3.0
R	R Foundation	v4.1.3
ggplot2	R package	V3.5.2
LEfSe	LEfSe	v1.0
GraphPad Prism	GraphPad Software	v9.0
SPSS	IBM	v25.0

**Other**		

Yuwell 660 glucometer	Yuwell	660
NovaSeq 6000 sequencing platform	Illumina	NovaSeq 6000
DNBSEQ-G99 sequencing platform	MGI	DNBSEQ-G99
Nexera X2 LC-30AD UHPLC	Shimadzu	LC-30AD
5500 QTRAP mass spectrometer	AB SCIEX	5500 QTRAP
UPLC BEH Amide column 1.7 μm 2.1 × 100 mm	Waters	186004801
Flow cytometer	BD	Aria II
Light fluorescence microscope	Nikon	Eclipse 80i

### Experimental model and study participant details

#### Animals

Sprague-Dawley rats: Six-week-old male Sprague-Dawley rats (200–220 g) were purchased from Chengdu Dossy Experimental Animals Co., Ltd. (Chengdu, Sichuan, China). The strain background was reported as Sprague-Dawley; no additional engineered genotype was reported. Following a 1-week acclimatization period, rats were randomly assigned to the experimental groups described under “Animal models and sample collection” (*n* = 6 per group).

C57BL/6J mice: Eight-week-old male C57BL/6J mice (20–25 g) were purchased from Chengdu Dossy Experimental Animals Co., Ltd. (Chengdu, Sichuan, China). The strain background was reported as C57BL/6J; no additional engineered genotype was reported. Mice were used as recipients in fecal microbiota transplantation experiments and for pancreatic/islet analyses described in the Results and Method Details. For the mouse experiments, *n* = 6 animals per group, where n represents individual mice.

Animal husbandry and regulatory oversight: Rats and mice were housed under specific pathogen-free conditions with a 12 h light/12 h dark cycle, controlled temperature of 20–22 °C, relative humidity of 40%–70%, and free access to food and water. The animal study was approved by the Ethics Committee of Sichuan Academy of Medical Sciences and Sichuan Provincial People’s Hospital (Ethical Approval No. 2021–314), and all animal experiments conformed to applicable institutional and national regulatory standards or guidelines.

#### Cell line

MIN-6 cells (mouse pancreatic beta-cell line) were purchased from Merck (cat. no. SCC623). Species: mouse. Genotype/derivation: MIN-6 cells were derived from an insulinoma tissue that developed in a transgenic mouse in which expression of Simian Virus 40 (SV40) Large T antigen was driven by the human insulin promoter. The age/developmental stage and sex of the donor mouse are not reported by the supplier. Cells were cultured in Dulbecco’s Modified Eagle Medium (DMEM) supplemented with 15% fetal bovine serum, 1% penicillin-streptomycin, and 50 μM beta-mercaptoethanol at 37 °C with 5% CO2. The cell line was authenticated by the supplier: mouse origin and the absence of interspecies contamination from human, rat, Chinese hamster, Golden Syrian hamster, and non-human primate cells were verified using the Contamination Clear panel performed by Charles River Animal Diagnostic Services. The supplier also reports negative testing using the Mouse Essential CLEAR panel and negative testing for mycoplasma. Cells were additionally confirmed to be mycoplasma-negative in our laboratory before use. No independent post-receipt authentication or additional genotyping was performed in our laboratory. For MIN-6 experiments, *n* = 3 independent experiments per group/condition, where n represents independent experimental replicates.

#### Primary pancreatic islets

Pancreatic islets isolated from the male C57BL/6J mice described above were used for *ex vivo* molecular analyses, including immunoblotting of islets from control, TAC, and TAC+L-His groups. Source-animal age, sex, housing conditions, and regulatory oversight are as stated above. The available manuscript does not describe a separate prolonged primary-islet culture or an independent primary-cell authentication procedure.

#### Microbial material

The study analyzed complex fecal microbial communities from rodent samples by 16S rRNA sequencing and used fecal microbial suspensions for fecal microbiota transplantation (FMT). These were community preparations rather than isolated, banked microbial strains. Streptococcus bovis and Selenomonas ruminantium were quantified in fecal DNA by species-specific quantitative PCR, but no isolated strains of these organisms were cultured as standalone experimental models. FMT suspensions were processed under anaerobic or oxygen-limited conditions in sterile pre-reduced phosphate-buffered saline with 0.05% cysteine. For inactivated FMT, suspensions were heated at 80 °C for 30 min, and complete inactivation was verified by absence of visible colony growth after 48 h at 37 °C on aerobic and anaerobic blood agar. Strain genotype, age/developmental stage, and sex are therefore not applicable to these community samples.

#### Human participants and samples

No human participants were enrolled and no human-derived samples were collected for the present study. Therefore, human sample size and assignment to experimental groups, sex and gender, ancestry, race, ethnicity, participant-care conditions, and human-subject institutional approvals are not applicable. No plant models were used.

#### Sex and gender considerations

Only male rats and mice were included in the *in vivo* experiments. Sex-dependent effects were not directly tested. This limits generalizability because sex may influence gut microbial composition, histidine metabolism, tacrolimus pharmacology, and pancreatic beta-cell responses. The magnitude and functional consequences of the observed microbiota-metabolite changes may differ in female animals, and future sex-stratified studies are warranted. Gender is not applicable to the animal experiments, and no human participants were included for assessment of gender-related effects.

### Method details

#### Reagents

Tacrolimus (TAC; S31618) was purchased from Shanghai Yuanye Bio-Technology Co., Ltd. (Shanghai, China) and dissolved in olive oil (767419, Shanghai Macklin Biochemical Technology Co., Ltd., China). L-Histidine was purchased from Sigma-Aldrich.

#### Animal models and sample collection

To establish a TAC-induced islet dysfunction model, healthy rats were used for *in vivo* experiments. After one week of acclimatization, rats were randomly assigned to the following groups (*n*=6 per group): control group, TAC group, TAC+L-His group, ABX group, ABX+TAC group, ABX+TAC+L-His group, TAC+FMT group, TAC+FMT-inactivated group, TAC+D-Mannitol group, TAC+ImP group, TAC+L-His+ImP group. Rats in the TAC group received daily intraperitoneal injections of TAC at 2 mg/kg/day for 30 consecutive days, whereas control rats received vehicle alone (olive oil, 0.3 mL, intraperitoneally) for the same duration. For mannitol treatment, 200 mg/kg were delivered orally, then after 4 h, the blood was collected. For ImP treatment, 100 mg/kg/day dose was delivered intraperitoneally for 7 days. At the end of the study, animals were anesthetized by intraperitoneal injection of pentobarbital sodium (75 mg/kg) and euthanized by cervical dislocation. Blood samples were collected from the abdominal aorta, and fecal samples were collected immediately after defecation for subsequent analyses.

#### Physiological and biochemical measurements

Body weight and blood glucose were monitored during the experimental period. Blood glucose levels were measured using a Yuwell 660 glucometer (Shanghai, China). After 4 weeks of treatment, intraperitoneal glucose tolerance tests (IPGTTs) were performed. Briefly, D-glucose (G8150, Solarbio, Beijing, China) was administered at 2 g/kg body weight via tail vein injection, and blood glucose levels were measured at 0, 15, 30, 60, and 120 min. Intraperitoneal insulin tolerance tests (IPITTs) was performed with 1 IU/kg body weight to evaluate insulin responsiveness.

Serum insulin levels were measured using a commercial rat insulin assay kit (RAB0904, Sigma-Aldrich, USA). Blood tacrolimus concentrations were determined using a commercial FK506 (TAC) assay kit (Guangzhou Aoruida Biotech, Guangzhou, China). Glycated serum albumin levels were measured using a commercial kit (Krishgen Biosystems, Wuhan, China).

#### Bacterial genomic DNA extraction and 16S rRNA illumina sequencing

##### DNA extraction and 16S rRNA amplification

To maintain the metabolite integrity and microbial composition, the fecal samples were collected immediately after defecation by a stress-induced method. The DNA was extracted using a QIAGEN QIAamp DNA Stool Mini Kit (QIAGEN, Germany) following the protocol provided by the kit. 16S rRNA/18SrRNA/ITS genes of distinct regions (16SV4/16SV3/16SV3-V4/16SV4- V5, 18SV4/18SV9, ITS1/ITS2, ArcV4) were amplified used specific primer (16SV4: 515F- 806R, 18SV4: 528F-706R, 18SV9: 1380F- 1510R, et al.^38^^,^^40^) with the barcode. All PCR reactions were carried out with 15 μL of Phusion High-Fidelity PCR Master Mix; 0.2 μM of forward and reverse primers, and about 10 ng template DNA. Thermal cycling consisted of initial denaturation at 98 °C for 1 min, followed by 30 cycles of denaturation at 98 °C for 10 s, annealing at 50°C for 30 s, and elongation at 72°C for 30 s and 72°C for 5 min.

##### Purification of PCR product

The PCR products were purified using magnetic bead purification. Samples were mixed in equal density ratios based on the concentration of PCR products. After thorough mixing, the PCR products were detected, and the target bands were recovered. For the PCR products generated by amplification, PCR product detection will be performed.

##### Library preparation and sequencing

Sequencing libraries were generated, and indexes were added. The library was checked with Qubit and real-time PCR for quantification and a bioanalyzer for size distribution detection. Quantified libraries were pooled and sequenced on NovaSeq6000 or DNBSEQ-G99 platforms, according to effective library concentration.

After the stool samples were tested on the machine, the barcode sequence and primer sequence were used to split the test data. The fastp software (V 0.23.1) was used to splice, quality control, and remove chimeras, and finally obtain valid data on the intestinal microbiota. The DADA2 module in the QIIME2 (V 1.9.1) software was used to remove the barcode sequence and primers to perform noise reduction on the raw data. The final amplicon sequence variants (ASVs) and feature table were obtained, and the species comparison of the database was performed.

#### Untargeted metabolomics analysis

##### Sample preparation

For fecal metabolomics, 100 mg of fecal sample was ground in liquid nitrogen and extracted in 500 μL of 80% methanol. For serum metabolomics, 100 μL of serum was mixed with 400 μL of 80% methanol. Samples were vortexed and centrifuged at 15,000 × g at 4 °C for 20 min. The supernatants were further diluted with 53% methanol and centrifuged again at 15,000 × g at 4 °C for 20 min. The final supernatants were used for liquid chromatography–mass spectrometry (LC-MS) analysis.

##### LC-MS conditions

Metabolite profiling was performed using a Shimadzu Nexera X2 LC-30AD ultra-high-performance liquid chromatography system coupled to a 5500 QTRAP mass spectrometer (AB SCIEX). Chromatographic separation was achieved using a Waters UPLC BEH Amide column (1.7 μm, 2.1 × 100 mm). The mobile phases consisted of solvent A (0.1% formic acid and 10 mM ammonium formate in water) and solvent B (0.1% formic acid in acetonitrile). Samples were reconstituted in 100 μL of 50% methanol and maintained at 4 °C prior to injection. The column temperature was set to 40 °C, the flow rate was 300 μL/min, and the injection volume was 2 μL.

Mass spectrometry was conducted in positive electrospray ionization mode. Source parameters were set as follows: source temperature, 550 °C; ion source gas 1, 40; ion source gas 2, 50; curtain gas, 35; and ion spray voltage, 5,500 V. Data were acquired in multiple reaction monitoring (MRM) mode.

##### Metabolomics data processing

Raw metabolomics data were processed using Compound Discoverer 3.0 (Thermo Scientific). Peaks were aligned based on retention time and mass-to-charge ratio, followed by peak extraction, signal correction, and integration. Metabolite annotation was performed by comparison with the mzCloud database. Background ions were removed using blank samples, and normalized quantitative values were used for downstream analyses.

#### Antibiotic-mediated gut microbiota depletion

Gut microbiota depletion was performed using a broad-spectrum antibiotic cocktail (ampicillin, 1g/L; neomycin, 1g/L; metronidazole, 1g/L and vancomycin, 0.5g/L) was administered by oral gavage for 3 weeks and was discontinued thereafter according to the study design. Depletion efficacy was verified using quantitative PCR of the universal bacterial 16S rRNA gene.

#### Fecal microbiota transplantation (FMT) and inactivation

Fresh fecal pellets from healthy donors were collected into sterile tubes, processed immediately under anaerobic or oxygen-limited conditions and suspended in sterile pre-reduced phosphate-buffered saline at approximately 100 mg feces/mL. 0.05% cysteine was added as a reducing agent. Suspensions were homogenized and centrifuged. Recipient mice received 200 μL of freshly prepared supernatant by oral gavage.

For inactivation, the supernatant was subjected to heat treatment in a water bath at 80 °C for 30 minutes to eliminate all viable microorganisms. To verify complete inactivation, an aliquot of the treated supernatant was inoculated onto both aerobic and anaerobic blood agar plates and incubated at 37 °C for 48 hours. Only batches showing no visible colony growth were considered successfully inactivated.

#### Quantification of histidine-producing bacteria, microbial histidine-biosynthesis genes, and histidine metabolites

Fecal microbial DNA was extracted for quantitative PCR analysis. Streptococcus bovis and Selenomonas ruminantium were quantified using the following primers. For Streptococcus bovis, the forward primer is 5′-TTCCTAGAGATAGGAAGTTTCTTCGG-3′ and reverse primer is 5′-ATGATGGCAACTAACAATAGGGGT-3’. For Selenomonas ruminantium, the forward primer is 5′-CAATAAGCATTCCGCCTGGG-3′ and the reverse primer is 5′-TTCACTCAATGTCAAGCCCTGG-3’. The microbial histidine-biosynthesis genes hisG and hisD were quantified using gene-specific primers (HisG: forward: 5′-ATATTCTGAAATGAGCTGTTGACAA-3’; reverse: 5′-TACTGCCGCCAGGCCAAATTC-3’; HisD: 5′-GTGATCCATATGAGCTTTAACACAA-3’; reverse: 5′-AGAATTCACGGTGCTCATGCTTGC-3’. Quantitative PCR was performed with SYBR Green MasterMix (Tiangen, Beijing, China), and absolute copy numbers were calculated from standard curves generated using serial dilutions of target-containing standards. Gene copy numbers were normalized to the amount of fecal DNA used in each reaction. Fecal and serum L-Histidine levels and imidazole propionate (ImP) abundance were determined from LC-MS.

#### Bioinformatic analysis

The following downstream data were processed using R software (version 4.1.3). Analysis of variance (ANOVA)^41^ was used to evaluate the alpha diversity (Shannon index, Simpson index) between different groups in the 16S sequencing data. PERMANOVA (Adonis)^42^ was used to test the clustering of microbial communities (PCoA). To create a species abundance information table, the microbiome’s ASV abundance data and species annotation were combined. To determine the Unifrac distance, which measures the variations in microbial communities among groups, the beta diversity was primarily computed using the phylogenetic relationship of ASVs. The variations in microbiota between the groups were assessed using linear discriminant analysis (LDA) results. The Kruskal-Wallis rank-sum test (*p* < 0.05) and LDA score ≥ 2 were the criteria for the Mann-Whitney U test, which was used to examine paired comparisons for linear discriminant analysis effect size (LEfSe) analysis.^43^ Additionally, the data’s dimension was reduced using R’s prcomp function, and the principal component analysis (PCA) diagram of metabolites was created using the ggplot2 package.

#### Cell viability assay

Cell viability was assessed using the MTT assay as previously described. Briefly, MIN-6 cells were seeded into culture plates, treated under the indicated conditions, and incubated with MTT reagent. Absorbance was measured to determine relative cell viability. Cell morphology was observed with a light microscope (Nikon, Japan).

#### Glucose-stimulated insulin secretion test

Cells are washed and pre-incubated for two hours in low-glucose (2.8 mM) Krebs-Ringer Bicarbonate HEPES (KRBH) buffer with 0.1% BSA after achieving 80–90% confluence. To induce insulin release, cells are then successively treated with basal glucose KRBH (2.8 mM, 30 minutes) and stimulatory glucose KRBH (16.7–25 mM, 30–60 minutes). Supernatants are gathered, and the amount of insulin was measured using a mouse insulin ELISA kit (Saipei Ltd., Wuhan, China). Total protein content (BCA assay) is used to normalize the results.

#### Quantitative real-time PCR

Total RNA was extracted from cells or tissues using TRIzol reagent according to the manufacturer’s instructions. RNA concentration and purity were determined spectrophotometrically. Complementary DNA (cDNA) was synthesized from 1 μg total RNA using a reverse transcription kit. Quantitative real-time PCR was performed using SYBR Green Master Mix on a real-time PCR detection system. Relative gene expression levels were calculated using the 2-ΔΔCt method withβ-actin as the internal control. Primer sequences: PDX-1-F: 5′-AGCAGTACTACGCGGCCACA-3’; R: 5′-GCACTTCGTATGGGGAGATG-3’; NeuroD1-F: 5′-CCTCAAGAAAGCGACGAAGAA-3’; R: 5′- CTTGTTTCCATCATGCTCTCG -3’; MafA-F: 5′-GCTCTGGAGTTGGCACTTCT-3’; R: 5′- CTTCAGCAAGGAGGAGGTCA-3’; 18 S-F: 5′- GTAACCCGTTGAACCCCATT -3’; R: 5′- CCATCCAATCGGTAGTAGCG -3′

#### Immunoblotting

Cells are lysed in RIPA buffer, and the BCA assay is used to quantify the protein. Samples were denatured for five minutes at 95°C. On an SDS-PAGE gel, load 20 μg of protein per lane, and run the protein by electricity. Then transfer proteins in cold transfer buffer to PVDF membranes at 100V for two hours. Block membrane using 5% BSA in TBST for one hour at room temperature. Incubate the membrane with the primary antibody at 4°C overnight. p-PI3K (# HA721672), PI3K (# HA722474), *p*-Akt (# ET1607-73), Akt (# ET1609-51), *p*-mTOR (# HA600094), total mTOR (# ET1608-5), PDX-1(# ER64542), NeuroD1(# ET1703-73), p70S6K (# HA722520), p-4E-BP1, eIF4EBP1 (# ET1701-83), and β-actin (# EM21002) were purchased from HUABIO. p-p70S6K (T389, ab2571), *p*-eIF4EBP1 (T37/46, ab278686) and MafA(ab324869) were purchased from Abcam. Membrane was wash with TBST for 10 minutes, three times. HRP-conjugated secondary antibody was incubated for one hour at room temperature. After washing for three times, an enhanced chemiluminescent substrate (ECL, Yeason Ltd., Shanghai, China) is used for detection, and an imaging system is used for visualization.

Molecular weight reference for the immunoblot figures: phospho-PI3K p110, approximately 110 kDa ; PI3K p110, approximately 110 kDa; phospho-Akt and total Akt, approximately 60 kDa; phospho-mTOR and total mTOR, approximately 289 kDa; phospho-p70S6K and total p70S6K, approximately 59 kDa; phospho-4E-BP1 and total 4E-BP1, approximately 13 kDa; PDX-1, approximately 40 kDa; NeuroD1, approximately 50 kDa; MafA, approximately 50 kDa; β-actin, approximately 42 kDa; and Histone H3, approximately 15 kDa. Molecular weight marker positions (kDa) should be displayed adjacent to all representative blot panels in the figure files.

#### Hematoxylin-eosin (HE) staining

Pancreatic tissues were fixed in 4% paraformaldehyde, embedded in paraffin, and sectioned at 5 μm thickness. After deparaffinization and rehydration through graded ethanol, sections were stained with hematoxylin and eosin according to standard protocols. Histological changes were observed and imaged under a light microscope.

#### Immunohistochemistry (IHC)

Paraffin-embedded pancreatic tissue sections were deparaffinized, rehydrated, and subjected to antigen retrieval in citrate buffer (pH 6.0). Endogenous peroxidase activity was blocked with 3% hydrogen peroxide, followed by blocking with normal serum at room temperature. Sections were then incubated with rabbit monoclonal insulin antibody (HUABIO, China, #ET1601-12) overnight at 4 °C, followed by incubation with HRP conjugated goat anti-rabbit IgG antibody. Immunoreactivity was visualized using 3,3′-diaminobenzidine (DAB), and nuclei were counterstained with hematoxylin. Images were captured under a light microscope.

#### Immunofluorescent staining

After fixing cells or tissue with 4% paraformaldehyde for 10–15 minutes, permeabilize it with 0.1–0.5% Triton X-100 for 5 minutes. To reduce non-specific binding, slides were incubated with blocking buffer (5% goat serum in PBS) for 60 minutes. Then the slides were incubated with primary antibody at 4 °C overnight, and subsequently fluorophore-conjugated secondary antibody for 2 hours at room temperature. Stain the nuclei with DAPI (1 μg/mL, 5 min) and wash 3 times with PBS. Visualize the cells or tissues with a fluorescent microscope (Nikon, Japan). The following antibodies were used for the primary antibodies: anti-insulin (# ET1601-12, HUABIO, China), anti-MAFA (# IHC-00352, Thermo Fisher, USA), anti-PDX-1 (# ET1705-99, HUABIO, China), and anti-NeuroD1 (#ET1703-73, HUABIO, China). Terminal deoxynucleotidyl transferase dUTP nick end labeling (TUNEL) staining was performed using a commercial kit (Beyotime, Suzhou, China).

#### Apoptosis assay

Apoptosis of MIN-6 cells was evaluated by Annexin V-FITC/propidium iodide (PI) staining followed by flow cytometry. After 24 h of treatment, both floating and adherent cells were collected. Briefly, culture supernatants containing suspended cells were carefully transferred to sterile centrifuge tubes. Adherent cells were detached using EDTA-free trypsin, gently resuspended, and combined with the corresponding supernatants. Cells were centrifuged at 1,000 rpm for 5 min at 4 °C, washed once with pre-chilled PBS, and resuspended in 1× binding buffer to a final concentration of 3 × 106 cells/mL.

For staining, 100 μL of cell suspension was incubated with 5 μL Annexin V-FITC for 5 min at room temperature in the dark, followed by the addition of 5 μL PI and 400 μL PBS. Samples were analyzed by flow cytometry (BD Aria II, USA) within 1 h. FITC fluorescence was detected in the FL1 channel, and PI fluorescence was detected in the FL2 or FL3 channel. Unstained controls and single-stained controls were prepared for voltage adjustment and compensation.

#### ELISA assays for serum insulin, tacrolimus, and glycated albumin

At the end of the animal experiment, blood samples were collected from the abdominal aorta under anesthesia and allowed to clot at room temperature. Serum was obtained by centrifugation and stored at −80 °C until analysis. Serum insulin, tacrolimus, and glycated serum albumin levels were measured using commercially available assay kits according to the manufacturers’ instructions.

Serum insulin levels were determined using a rat insulin ELISA kit (RAB0904, Sigma-Aldrich, USA). Serum tacrolimus concentrations were measured using a commercial FK-506 assay kit (Guangzhou Aoruida Biotech, Guangzhou, China). Glycated serum albumin levels were quantified using a commercial kit (Krishgen Biosystems, Wuhan, China). Absorbance values were recorded using a microplate reader, and concentrations were calculated based on the corresponding standard curves provided with each kit. All samples were assayed in accordance with the manufacturers’ protocols, and quality control standards were included where applicable.

### Quantification and statistical analysis

Unless otherwise indicated in the corresponding figure or table legend, quantitative data are presented as mean ± standard deviation (SD), with the arithmetic mean used as the measure of center and SD as the measure of dispersion. Confidence intervals are reported where shown (for example, Figure 1K). SEM is not reported. A *p* value < 0.05 was considered statistically significant. Where statistical significance is displayed as asterisks, the notation is ∗*p* < 0.05, ∗∗*p* < 0.01, and ∗∗∗*p* < 0.001; ns indicates not significant. The name of the statistical test is stated in each figure or supplemental-figure legend in which asterisks are used. Statistical details for individual experiments, including the statistical tests used, exact *n* values, definitions of n, measures of center and dispersion, and significance thresholds, are provided in the corresponding figure legends, figures, Results, tables, and Method Details.

Statistical analyses were performed using GraphPad Prism software (version 9.0) and SPSS software (version 25.0), with downstream microbiome analyses performed in R software (version 4.1.3). One-way ANOVA and the Wilcoxon rank-sum test were used, as appropriate, for group comparisons. For Figure 1, alpha-diversity comparisons (Shannon and Simpson indices; panels E-F) were evaluated by ANOVA, PCoA clustering (panel G) by PERMANOVA (Adonis), LEfSe analysis (panel J) by the Kruskal-Wallis rank-sum test with Mann-Whitney U tests for pairwise comparisons and the specified LDA-score criterion, and the day-30 genus-level comparison (panel K) by Welch’s *t*-test as described in the Results. Metabolomic datasets were analyzed using PCA and PLS-DA; differential-metabolite selection used *p* < 0.05 and VIP ≥ 1 as specified in the Figure 2 legend. The R prcomp function and ggplot2 package were used for PCA processing and visualization.

For Figure 3, Spearman’s correlation was used for panels A-B, the Wilcoxon rank-sum test for the two-group comparisons in panels E-K, and one-way ANOVA for the multi-group comparisons in panels L-R. For Figures 4, 5, and 7, quantitative comparisons denoted by asterisks were analyzed by one-way ANOVA. For Figure 6, two-way ANOVA was used for panels A, B, C, and E, whereas one-way ANOVA was used for the remaining quantitative comparisons. For supplemental analyses, one-way ANOVA was used for the asterisk-coded multi-group comparisons in Figures S1, S2, and S4, whereas the Wilcoxon rank-sum test was used for the asterisk-coded control-versus-TAC comparisons at the indicated time points in Figure S3. Associations between microbiome features and metabolic parameters were assessed using Spearman’s or Pearson’s correlation tests as described in Method Details, with Spearman correlation specifically used for the Figure 3 heatmaps.

For the *in vivo* rat and mouse experiments, *n* = 6 animals per group, where n represents individual animals. For MIN-6 cell experiments, *n* = 3 independent experiments per group/condition, where n represents independent experimental replicates. Tables 1 and 2 report *N* = 6 per group for the metabolomics analyses.

#### Ethics statement

The animal study received ethical approval from Ethics Committee of Sichuan Academy of Medical Sciences and Sichuan Provincial People’s Hospital, Ethical Approval No. 2021–314. All animal experiments conformed to the relevant institutional/national regulatory standards or guidelines.
