# Supplementary material for: L-Histidine alleviates tacrolimus-induced islet dysfunction via the gut-pancreas axis
Source: iScience. 2026 Oct 9;29(11):117802. doi: 10.1016/j.isci.2026.117802 (PMC13677189; doi:10.1016/j.isci.2026.117802)

iScience, Volume *29*

Supplemental information

L-Histidine alleviates tacrolimus-induced islet dysfunction via the gut-pancreas axis

Zhen Geng, Qiang Li, Duo Wang, Hongji Yang, Hailian Wang, Jinku Bao, Yi Wang, and Liang Wei

Supplemental Information

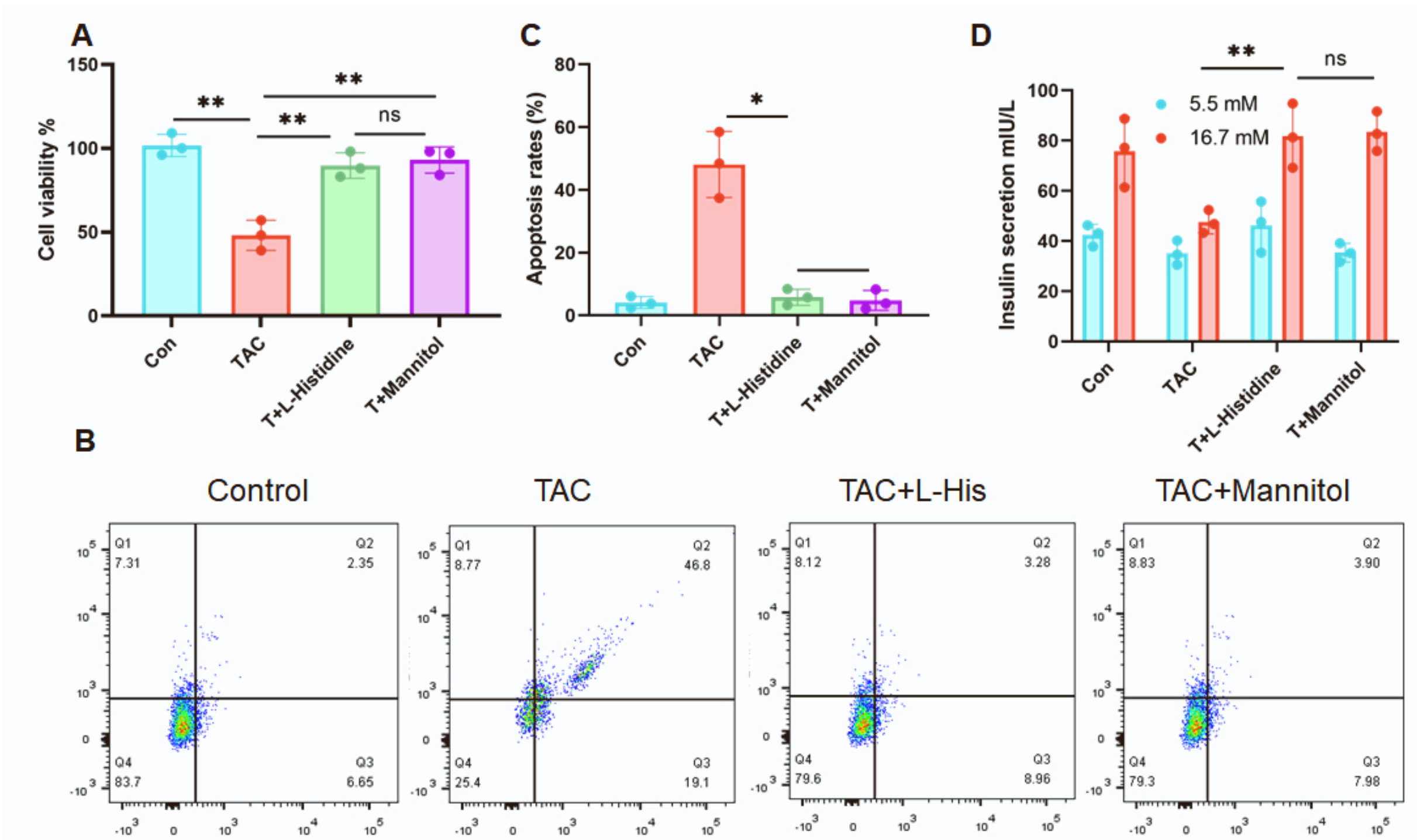

Figure S1. Distinct effects of L-His and D-(-)-mannitol on TAC-induced β-cell injury.

(A) Cell viability of control, TAC, TAC+L-His, and TAC+D-(-)-mannitol-treated MIN-6 cells. (B) Representative Annexin V-FITC/PI flow-cytometry plots for the four groups. (C) Quantification of apoptosis rates. (D) Glucose-stimulated insulin secretion. Data are presented as mean ± SD. Statistical test for quantitative comparisons denoted by asterisks: one-way ANOVA. *P < 0.05, **P < 0.01; ns, not significant. n = 3 independent experiments.

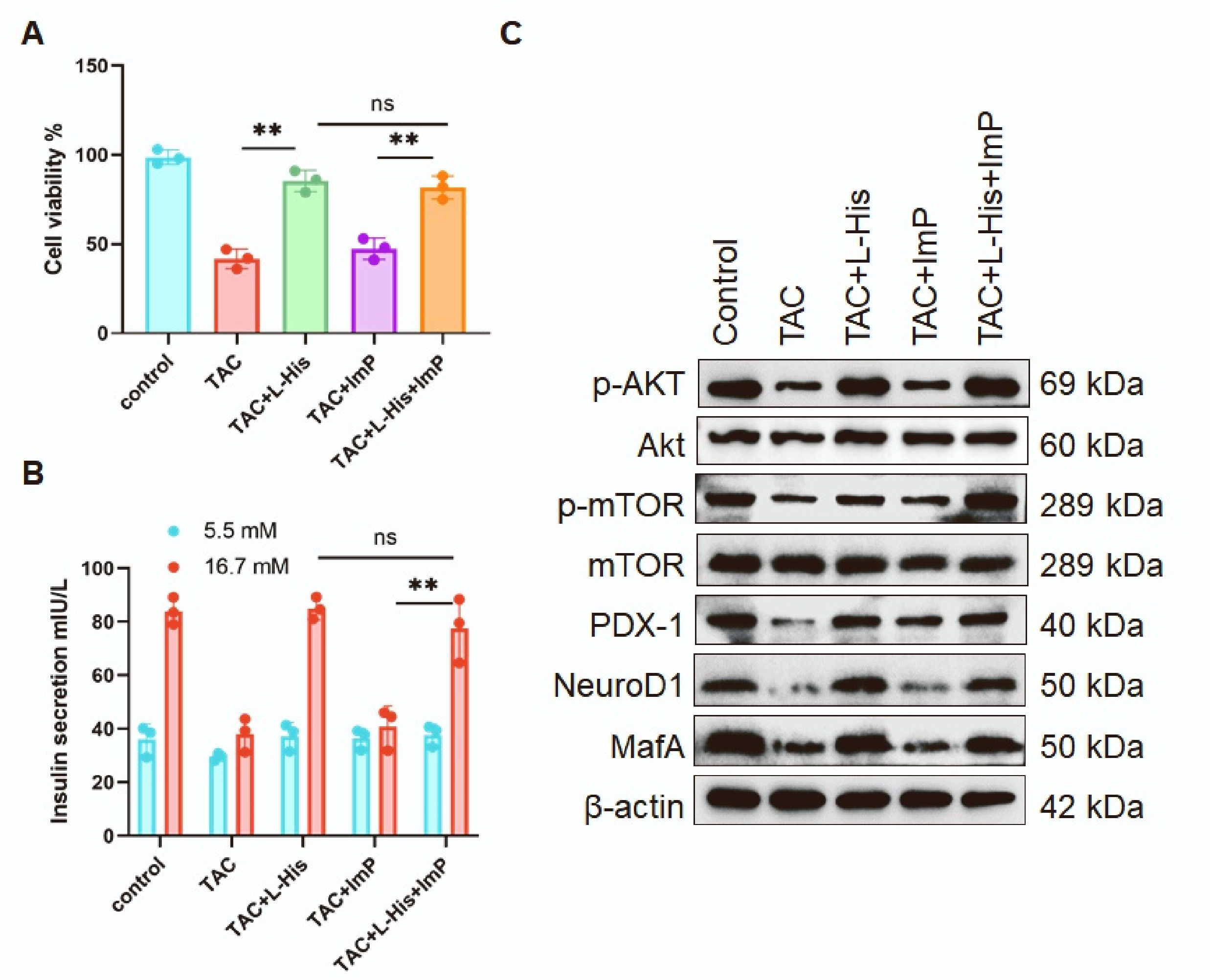

Figure S2. L-His counteracts ImP-associated impairment in TAC-treated β-cells.

(A) Cell viability in control, TAC, TAC+L-His, TAC+ImP, and TAC+L-His+ImP groups. (B) Glucose-stimulated insulin secretion. (C) Immunoblotting of p-Akt, total Akt, p-mTOR, total mTOR, PDX-1, NeuroD1, and MafA, with β-actin as the loading control. Data are presented as mean ± SD. Statistical test for quantitative comparisons denoted by asterisks: one-way ANOVA. **P < 0.01; ns, not significant. n = 3 independent experiments.

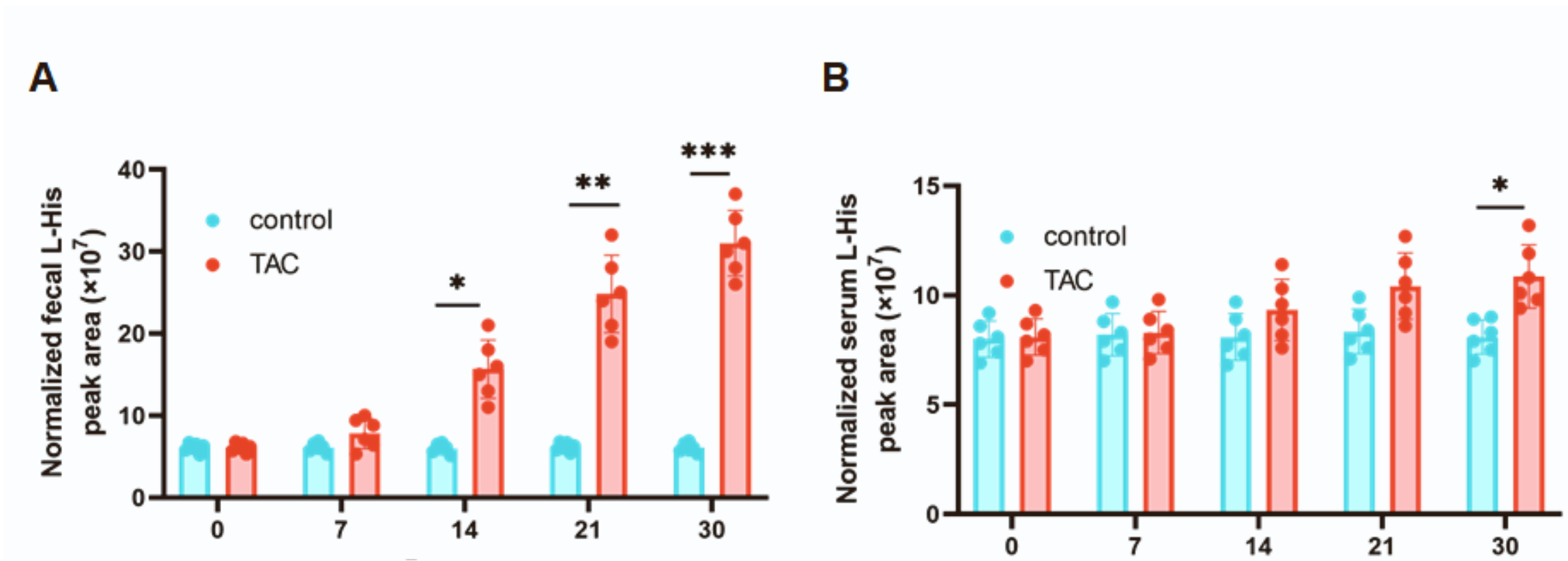

Figure S3. Longitudinal changes in fecal and serum L-His during TAC exposure.

(A) Normalized fecal L-His peak area in control and TAC-treated animals at days 0, 7, 14, 21, and 30. (B) Normalized serum L-His peak area data are presented as mean ± SD. Statistical test for asterisk-coded control-versus-TAC comparisons at the indicated time points: Wilcoxon rank-sum test. *P < 0.05, **P < 0.01, ***P < 0.001. n = 6 animals per group, where n represents individual animals.

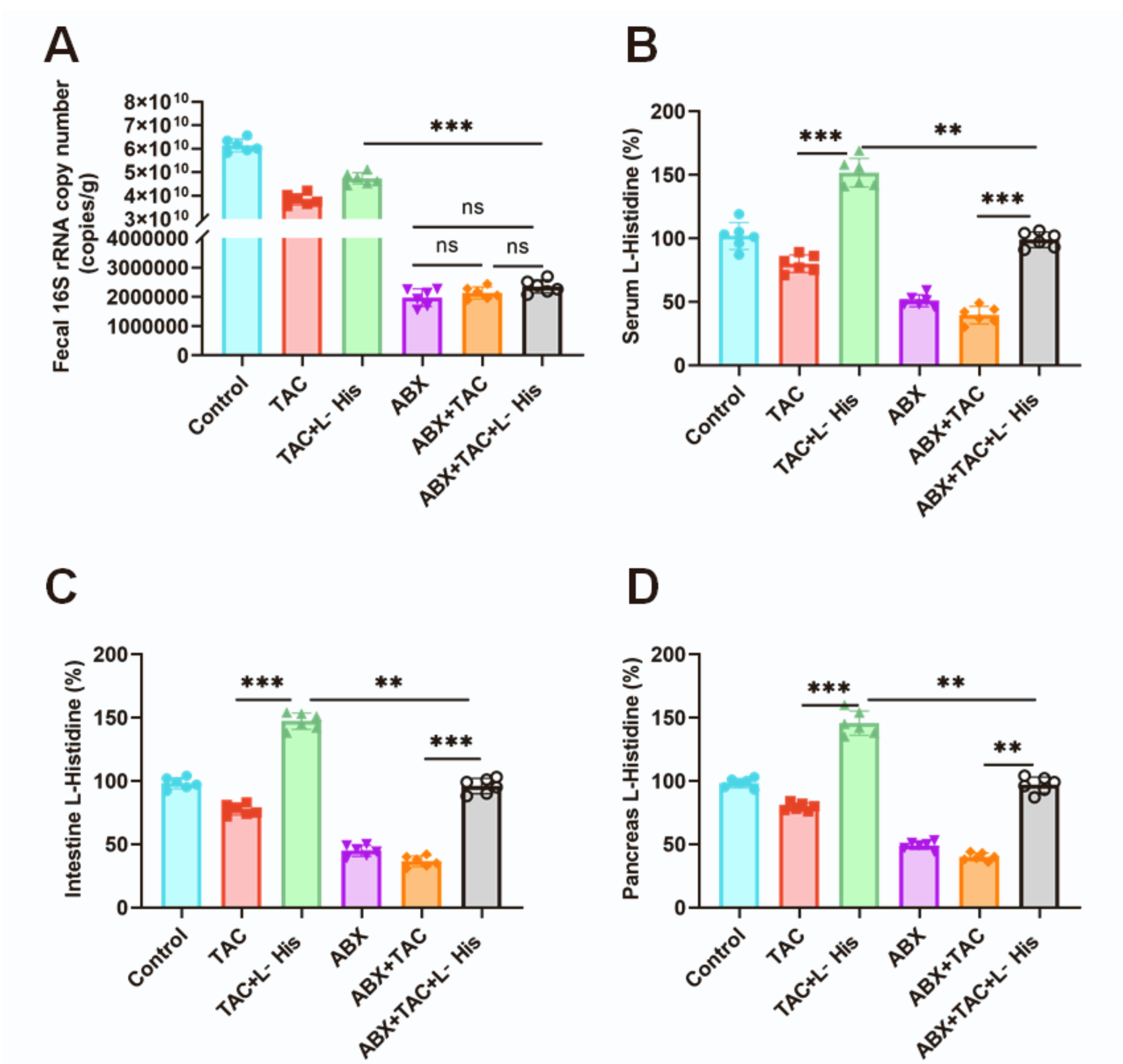

Figure S4. Antibiotic-mediated microbiota depletion.

(A) Fecal 16S rRNA copy number in control, TAC, TAC+L-His, ABX, ABX+TAC, and ABX+TAC+L-His groups, confirming marked bacterial depletion after antibiotic treatment. Relative L-Histidine abundance in (B) serum, (C) intestinal tissue, and (D) pancreatic tissue. Exogenous L-His restored L-His abundance in ABX+TAC animals despite persistent microbiota depletion. Data are presented as mean ± SD. Statistical test for quantitative comparisons denoted by asterisks: one-way ANOVA. **$P < 0.01$, ***$P < 0.001$; ns, not significant. n = 6 mice per group, where n represents individual mice.

Data S1: Uncropped western blot images corresponding to Figures 4–7 and Figure S2.

FIGURE 4K

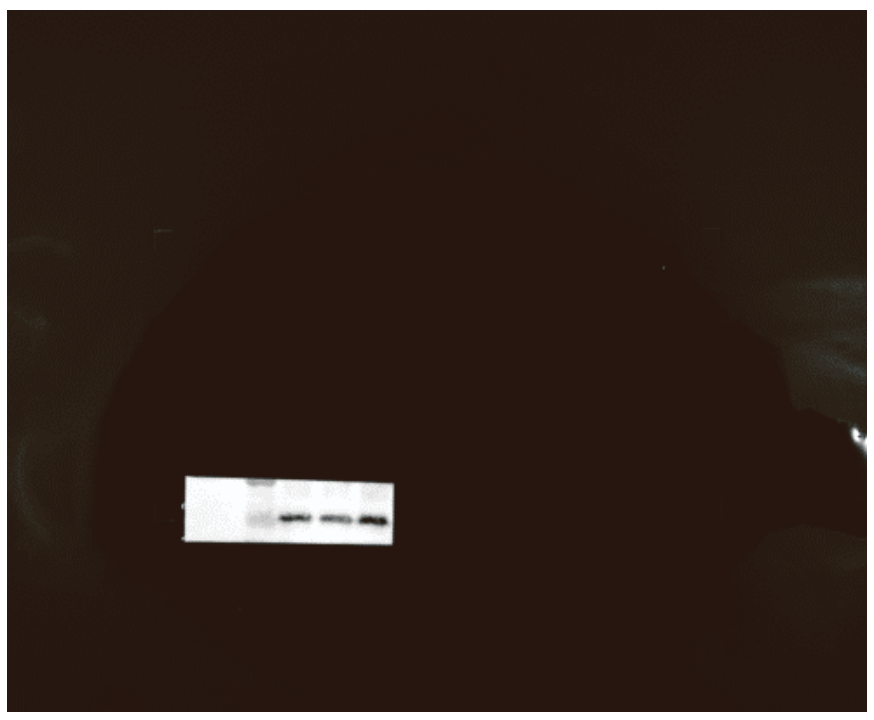

Figure 4K p-PI3K （110 kDa）

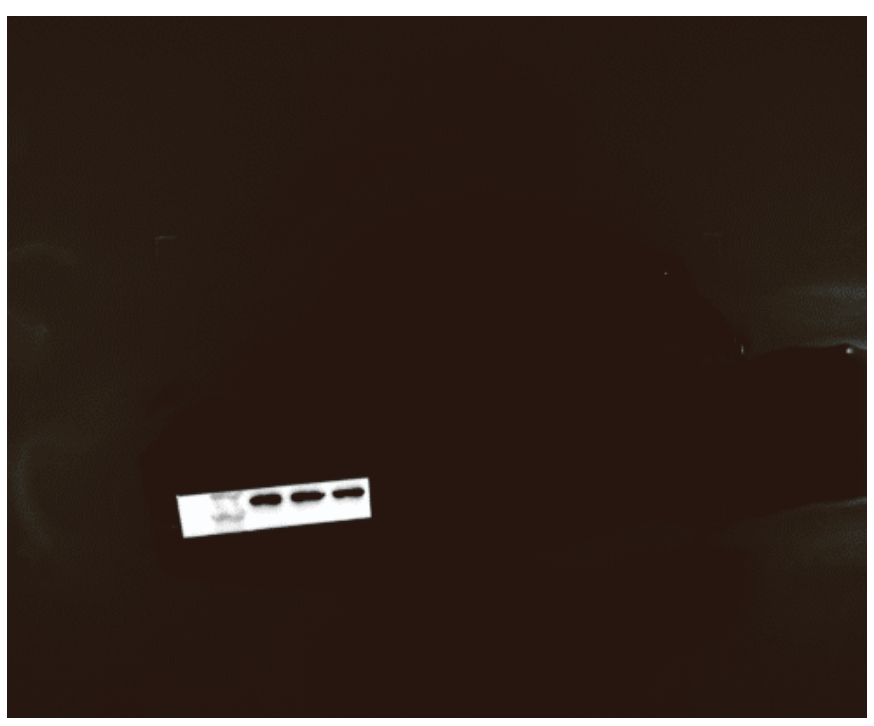

Figure 4K PI3K （110 kDa）

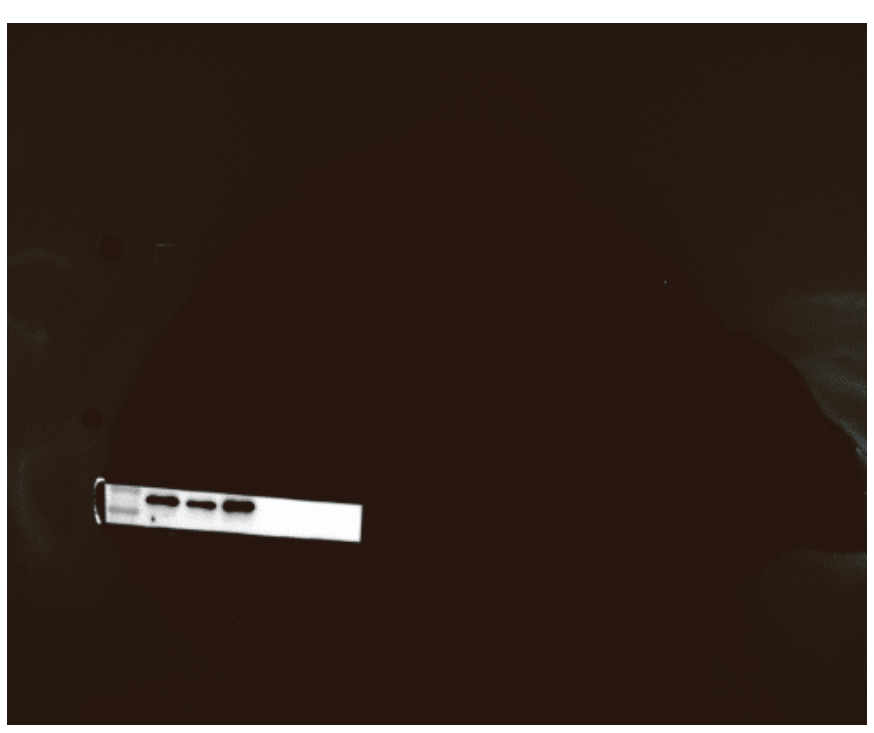

Figure 4K p-Akt (60 kDa)

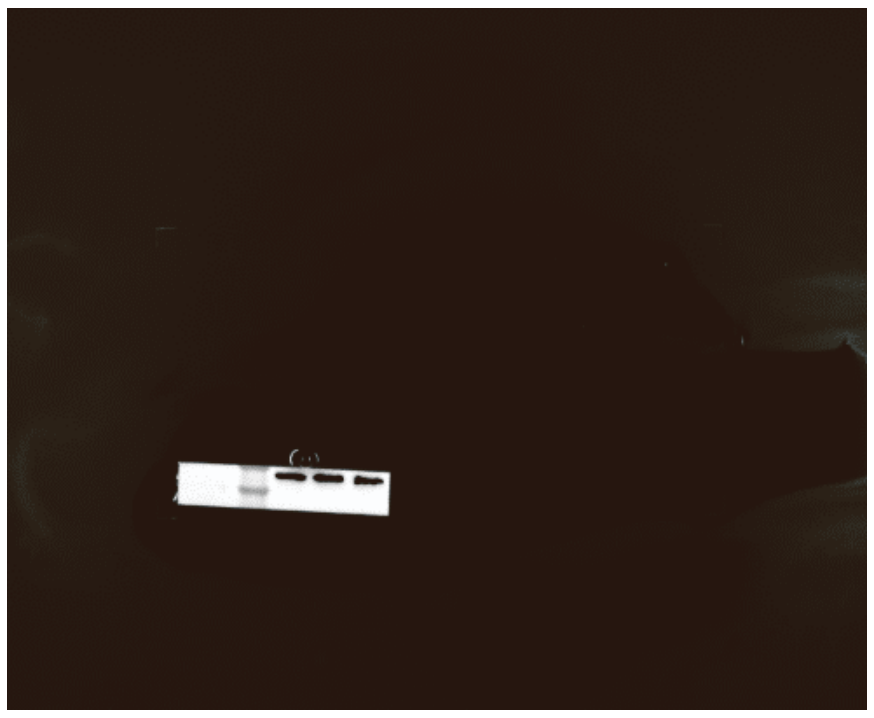
Figure 4K Akt (60 kDa)

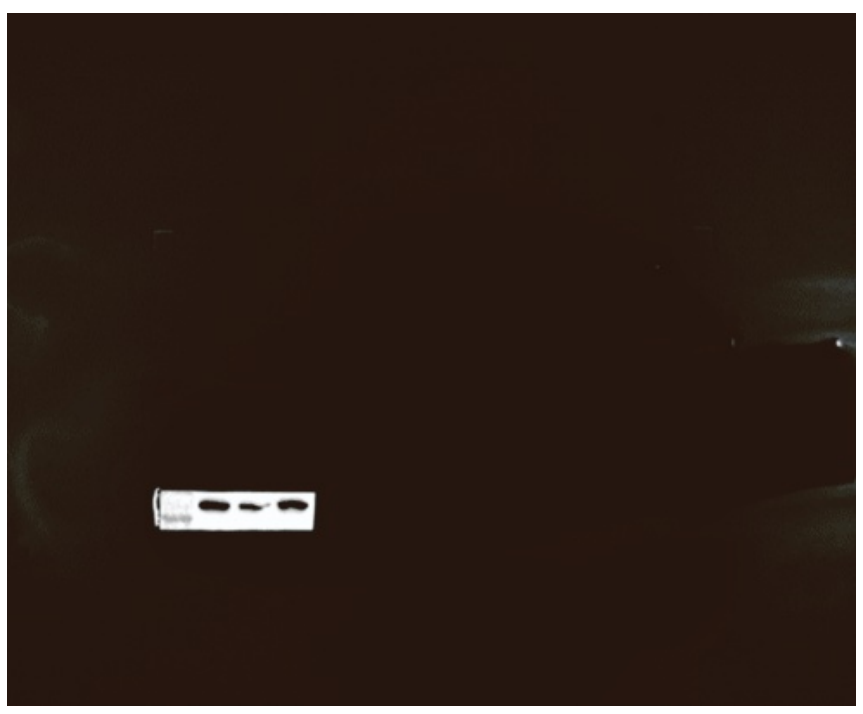
Figure 4K p-mTOR (~289 kDa)

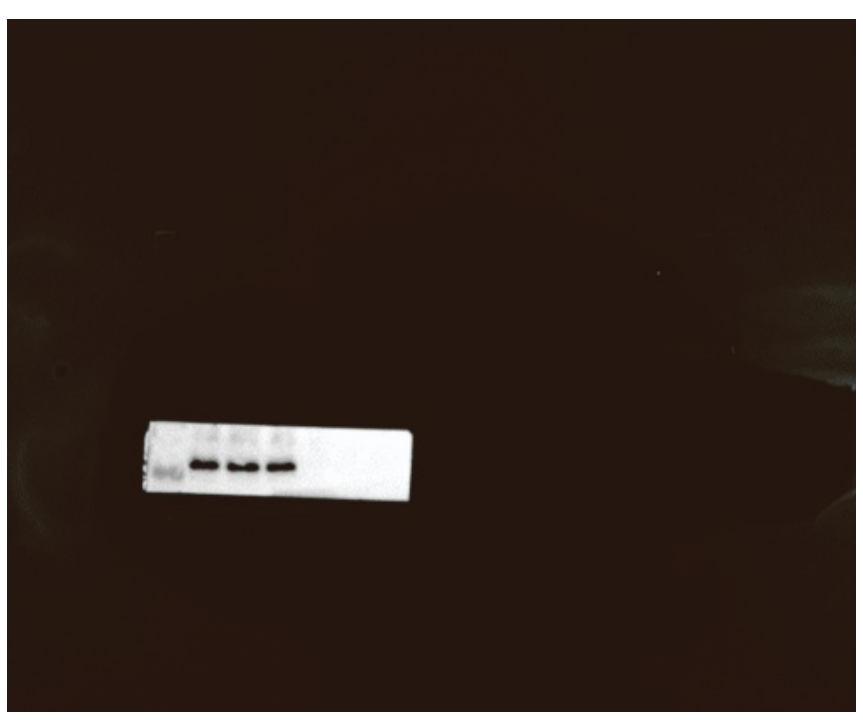
Figure 4K mTOR (~289 kDa)

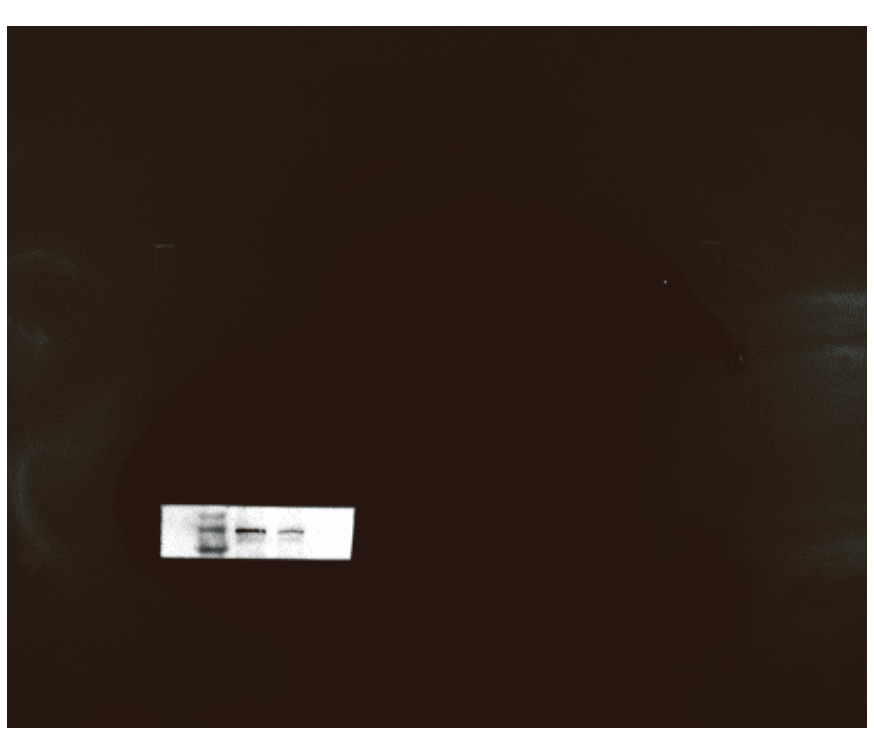
Figure 4O p-PI3K （110 kDa）

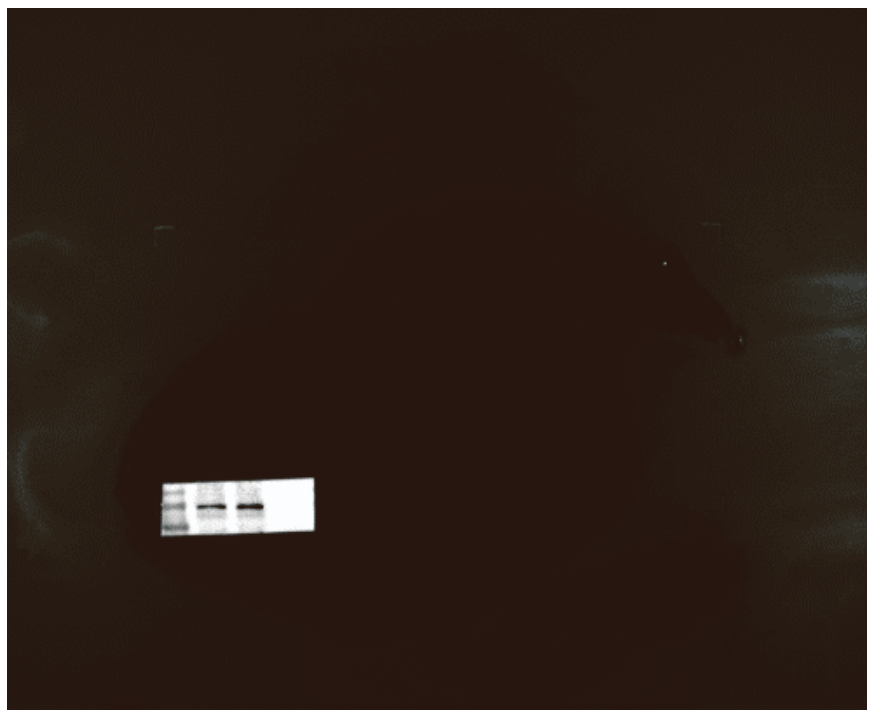
Figure 4O PI3K （110 kDa）

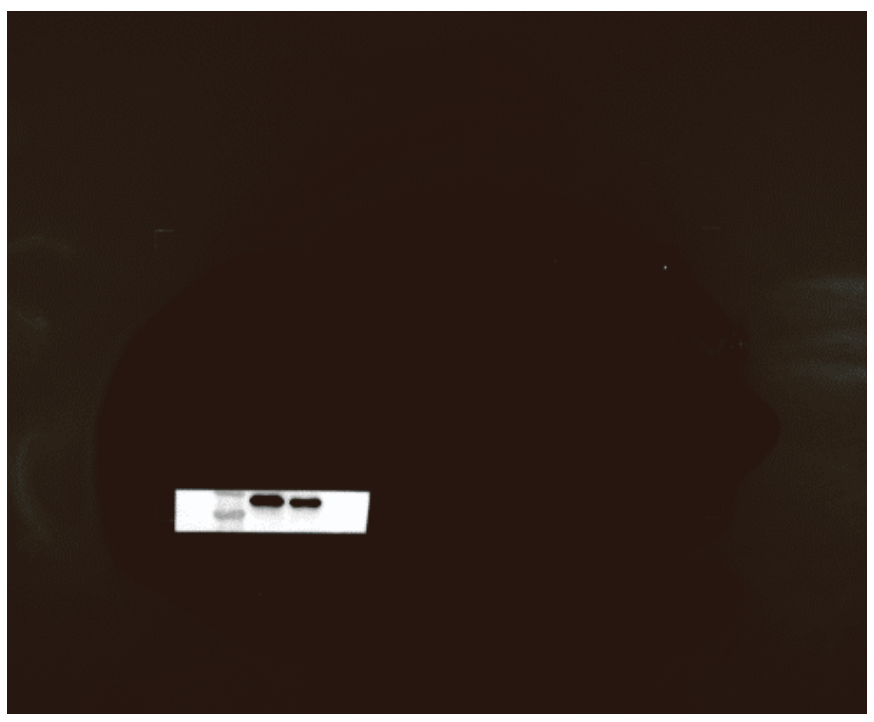
Figure 4O p-Akt (60 kDa)

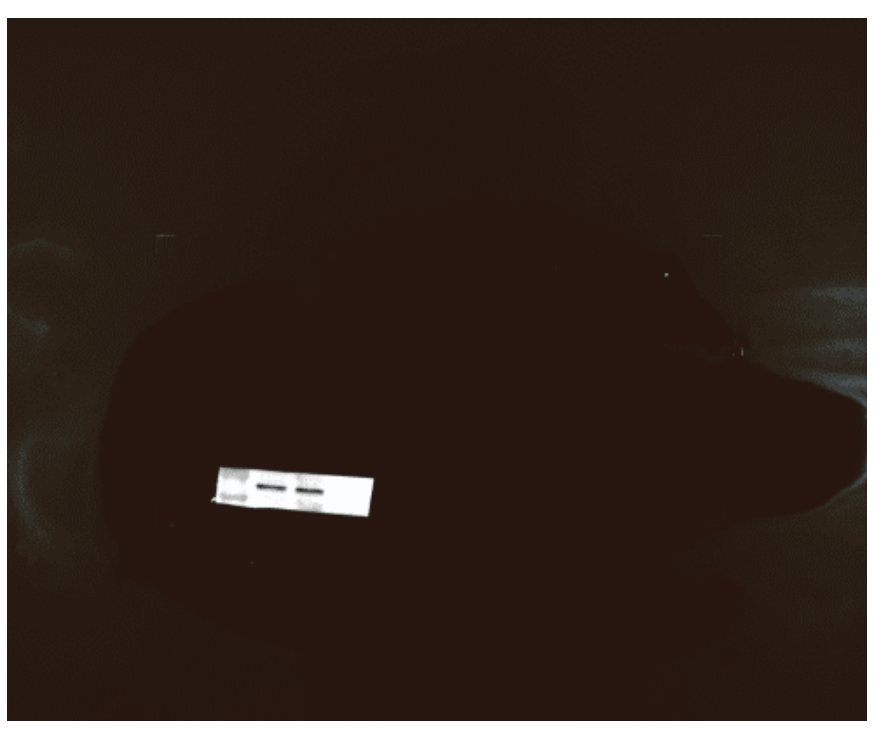
Figure 4O Akt (60 kDa)

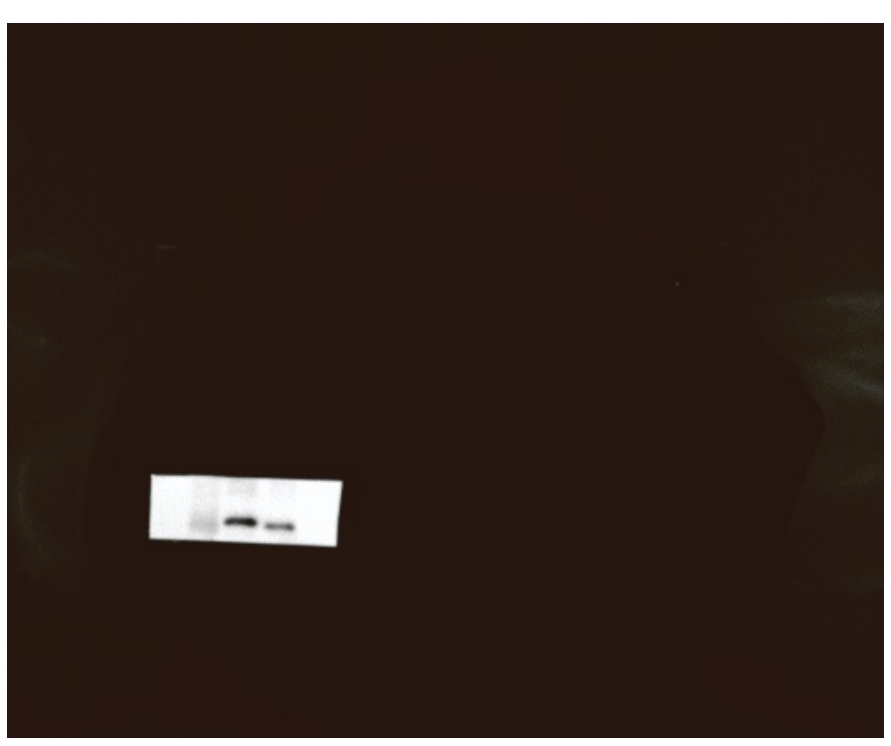
Figure 4O p-mTOR (~289 kDa)

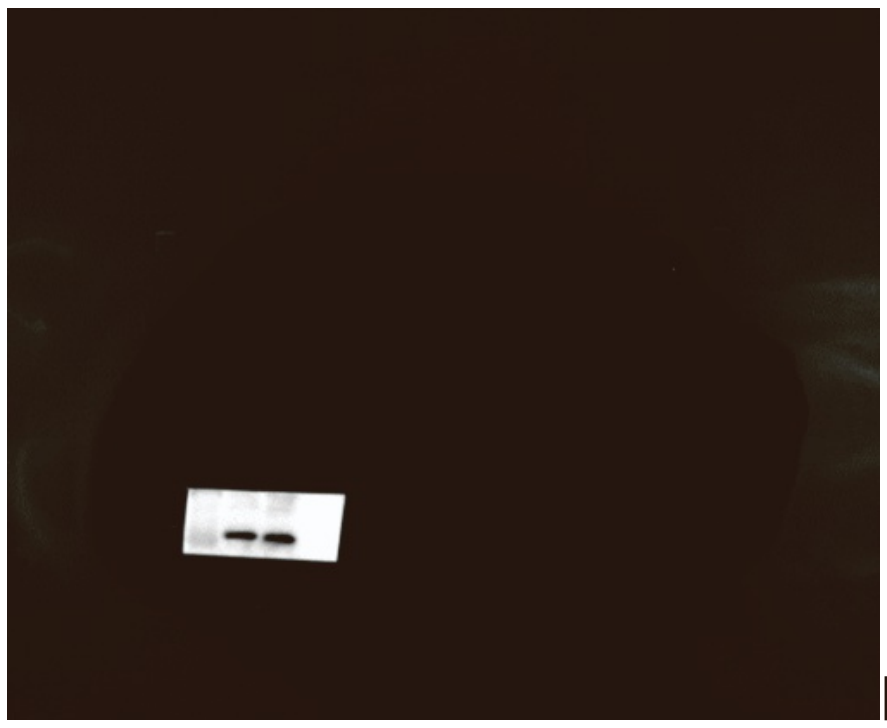
Figure 4O mTOR (~289 kDa)

FIGURE 5K

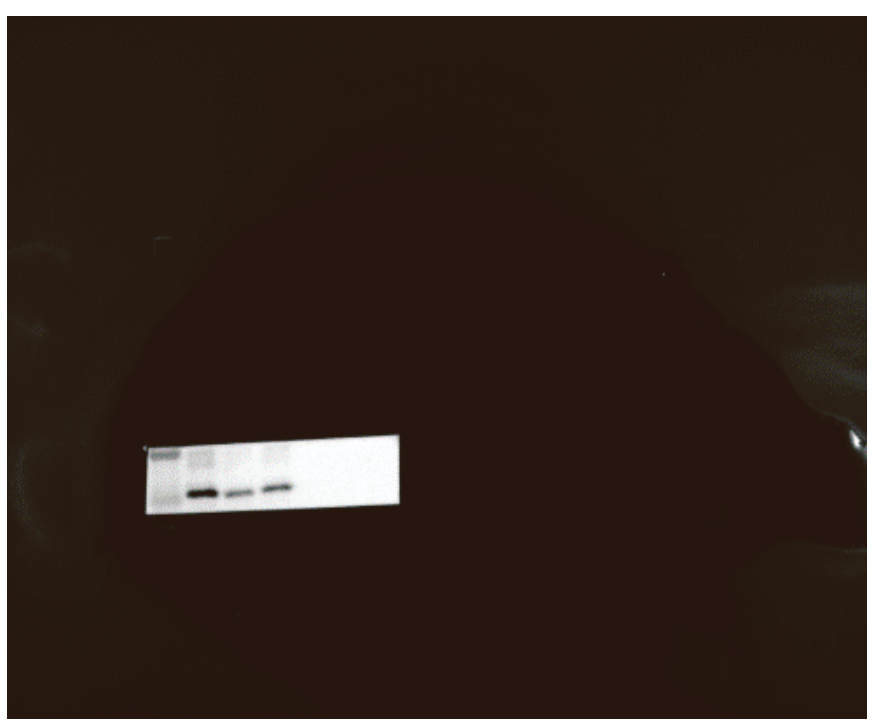
Figure 5D PDX-1 (40 kDa)

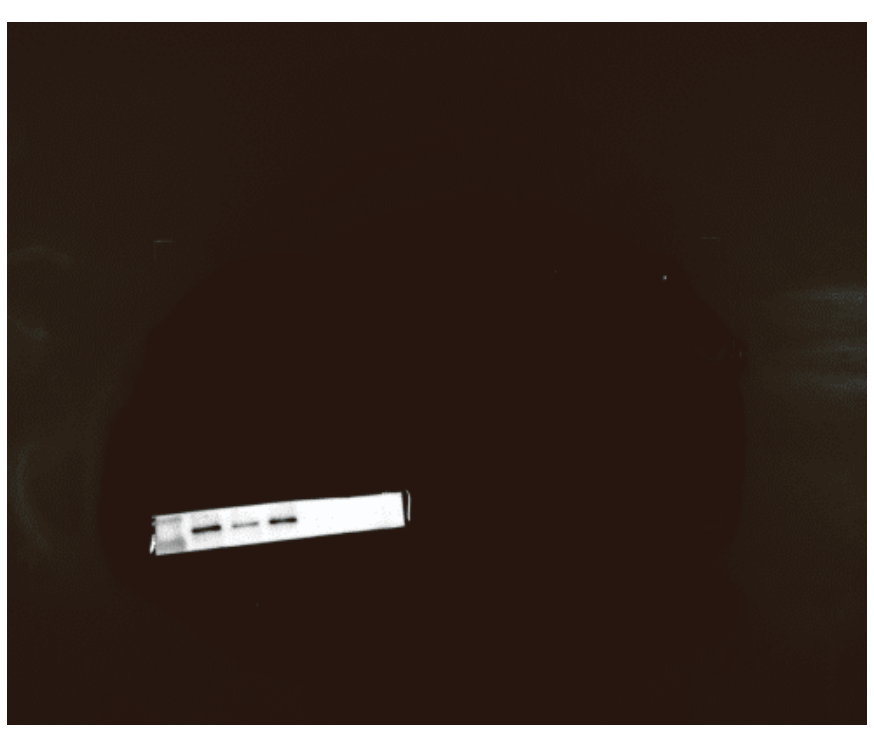
Figure 5D NeuroD1 (50 kDa)

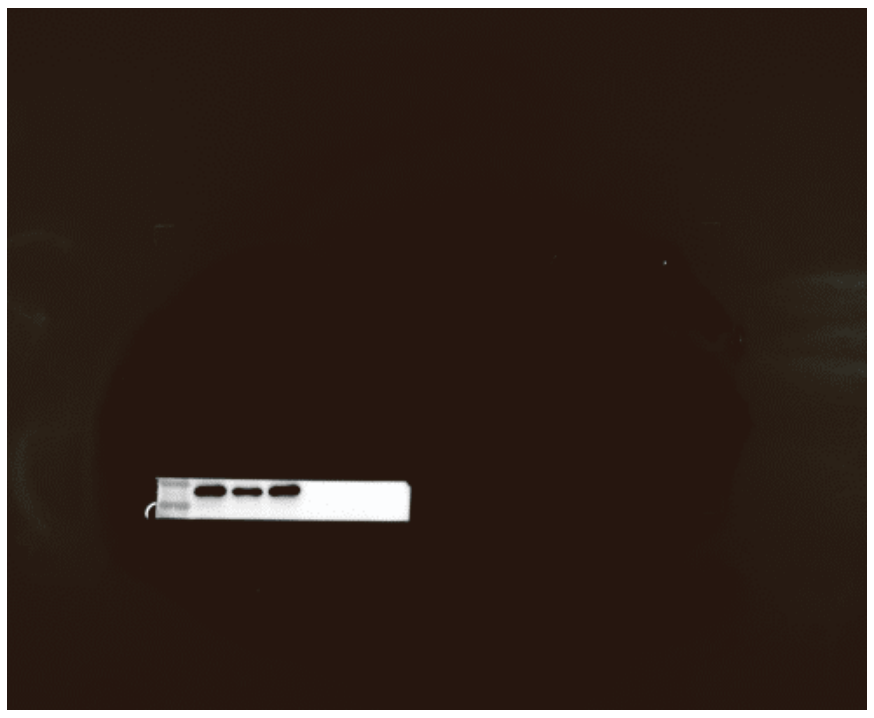
Figure 5D MafA (50 kDa)

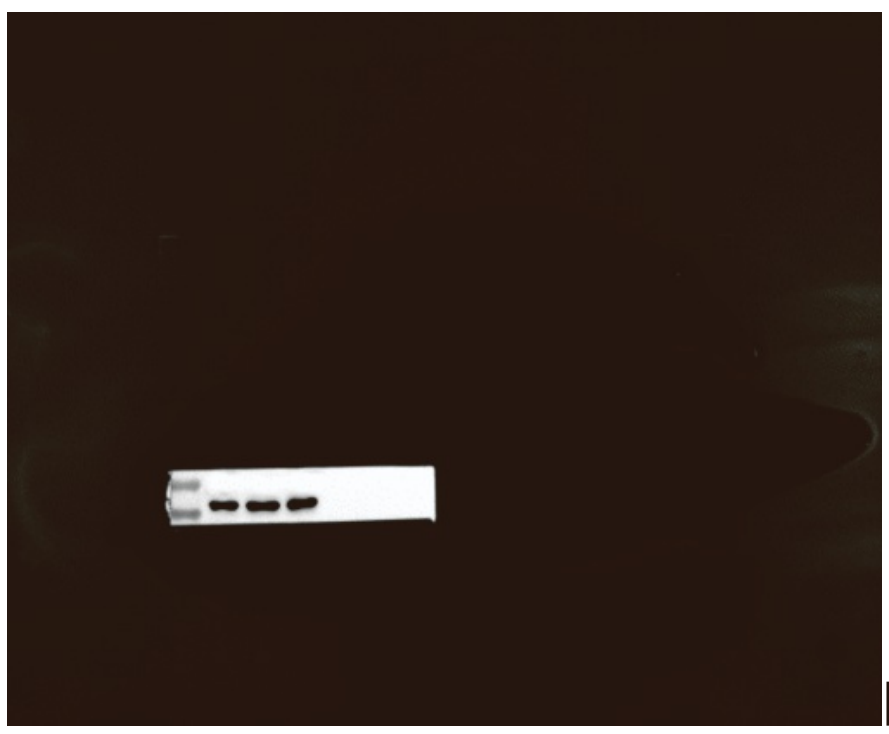
Figure 5D Histone H3 (15 kDa)

FIGURE 6N

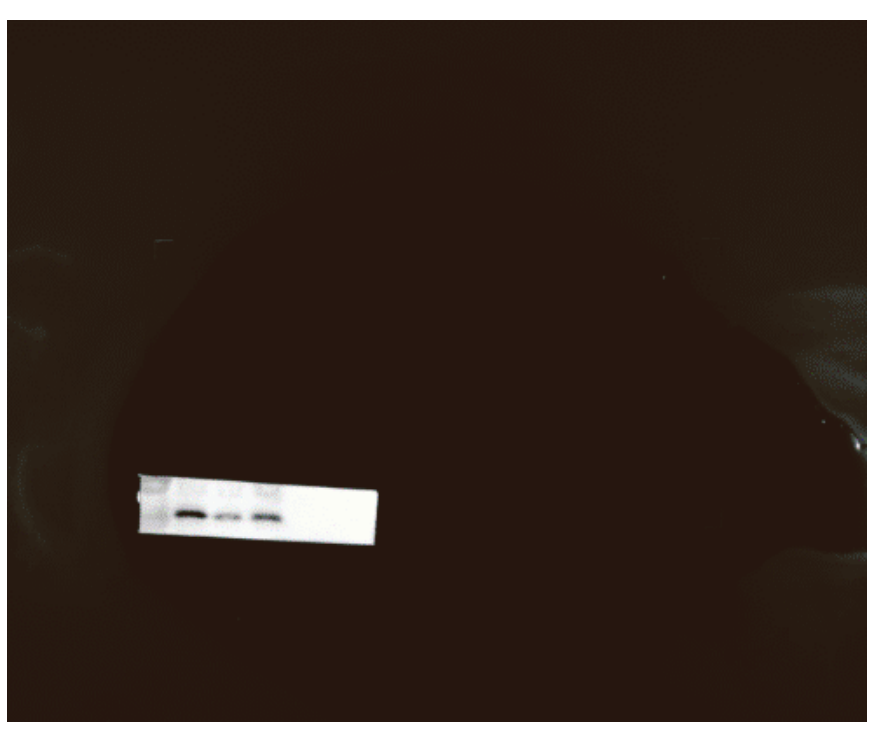
Figure 6N p-PI3K （110 kDa）

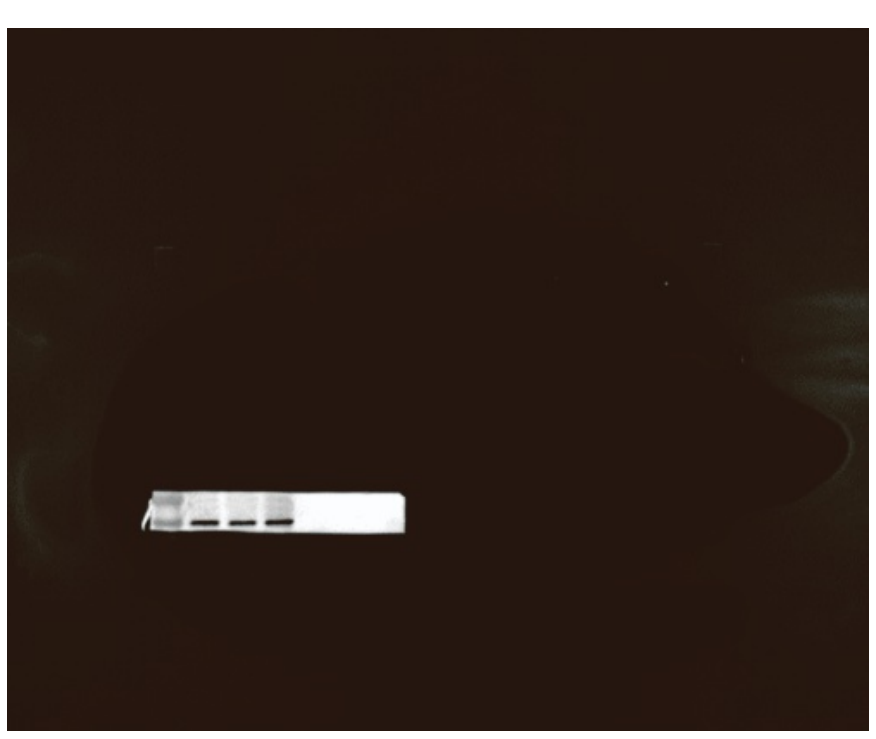
Figure 6N PI3K (110 kDa）

63 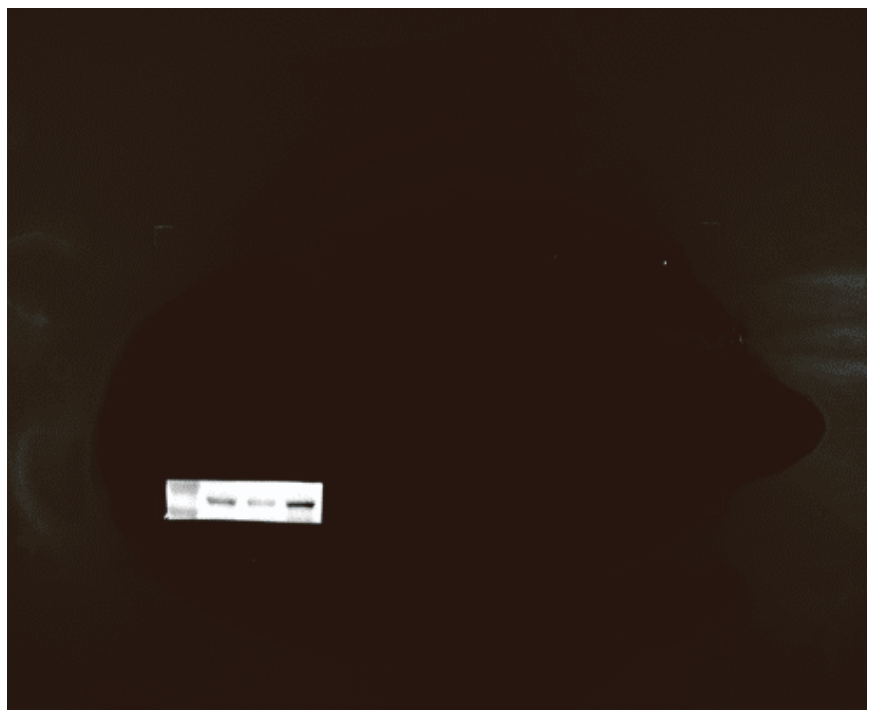 Figure 6N p-Akt (60 kDa)

64 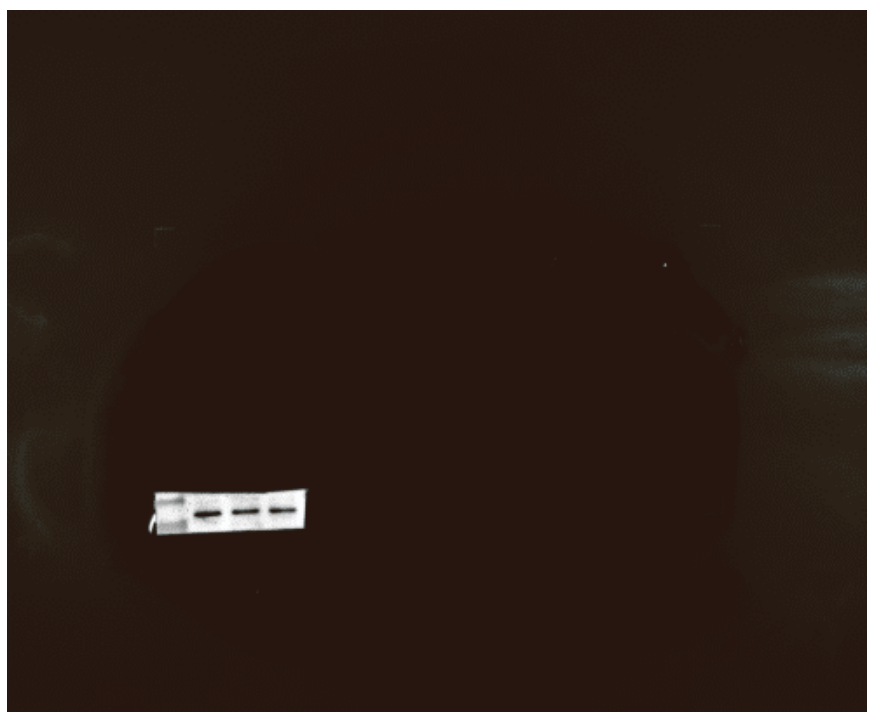 Figure 6N Akt (60 kDa)

65 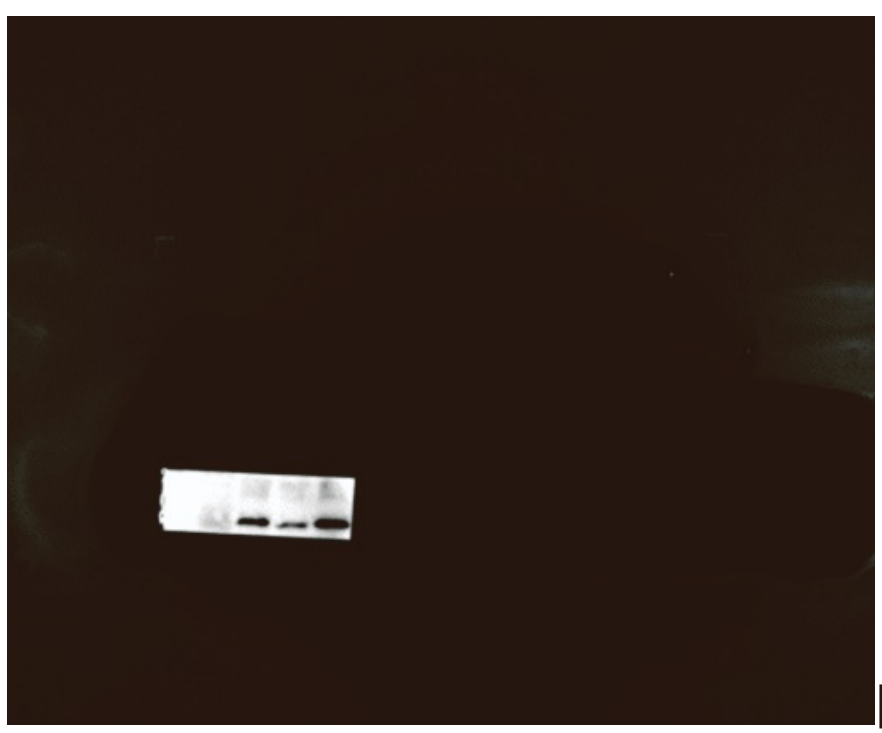 Figure 6N p-mTOR (~289 kDa)

66 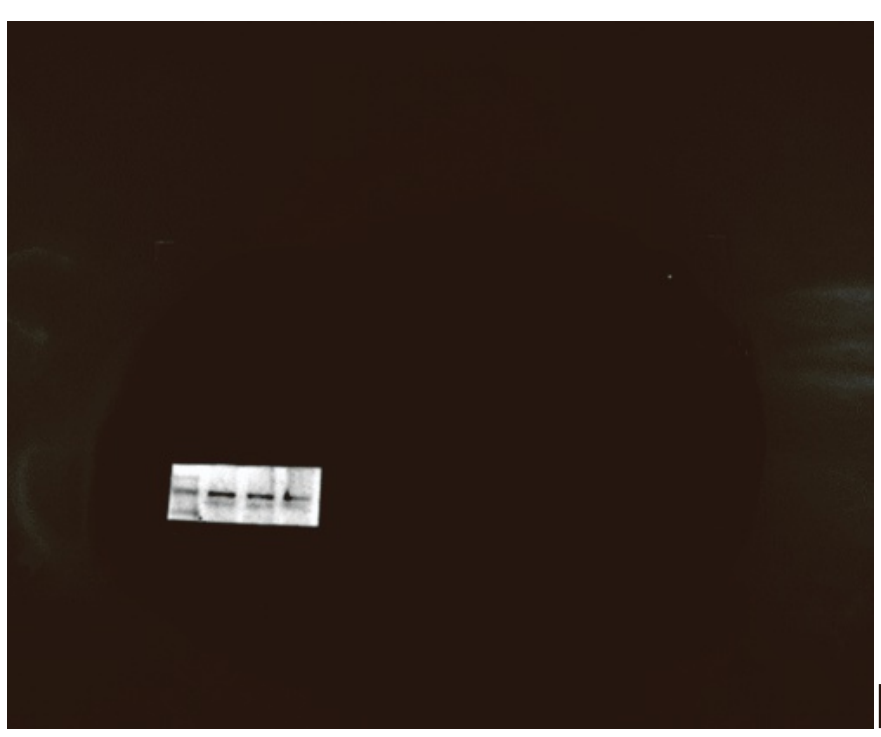 Figure 6N mTOR(~289 kDa)

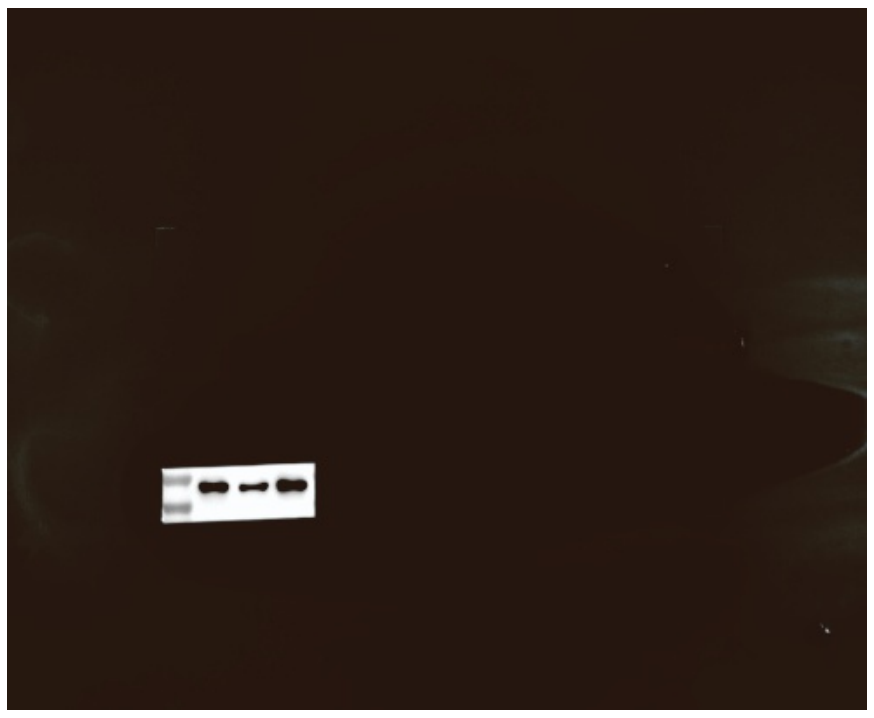
Figure 6N p-p70S6K (59 kDa)

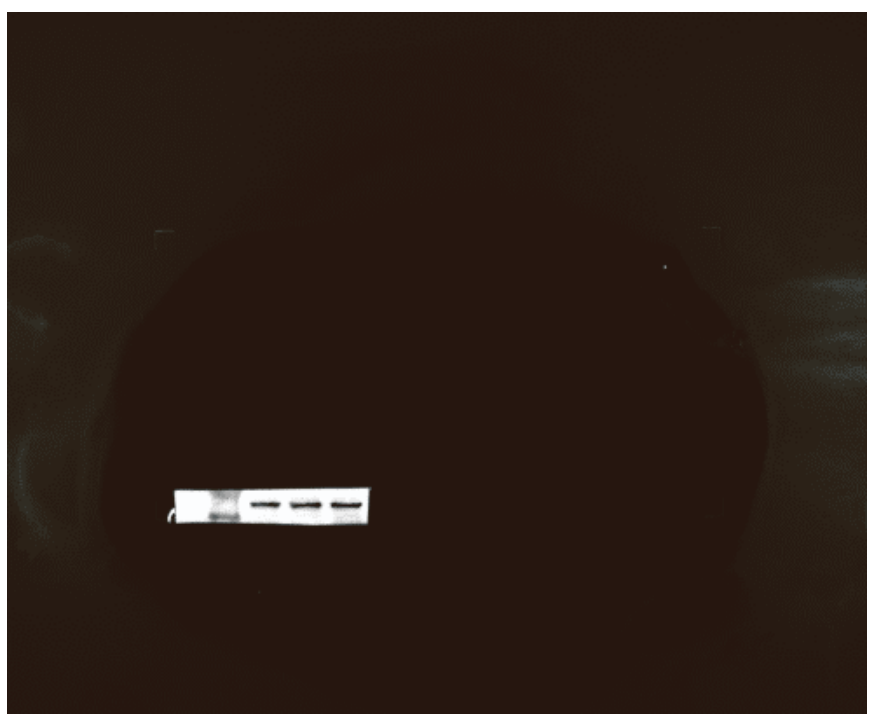
Figure 6N p70S6K (59 kDa)

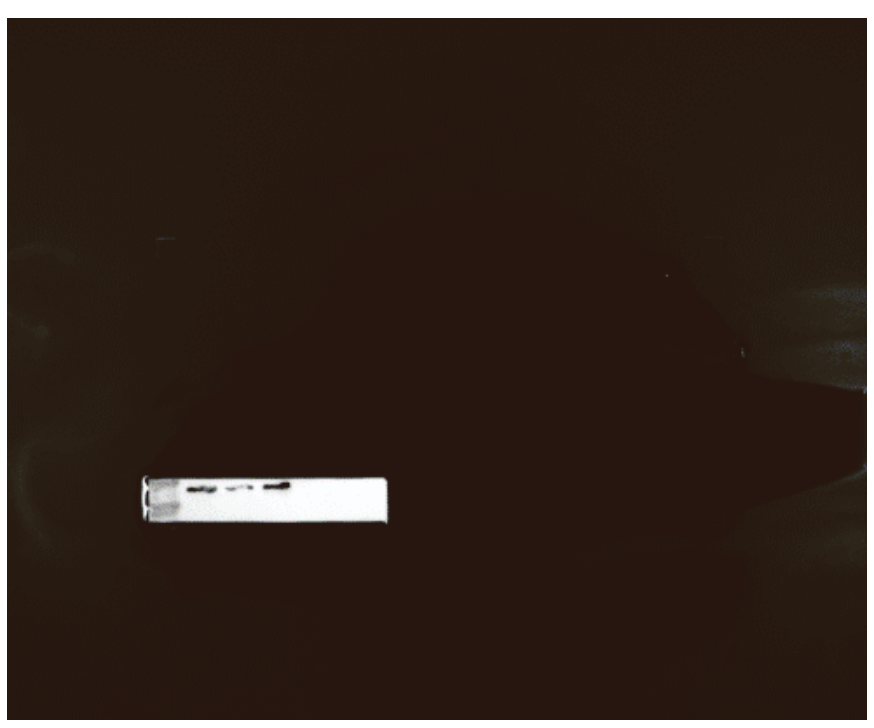
Figure 6N p4E-BP1 (13 kDa)

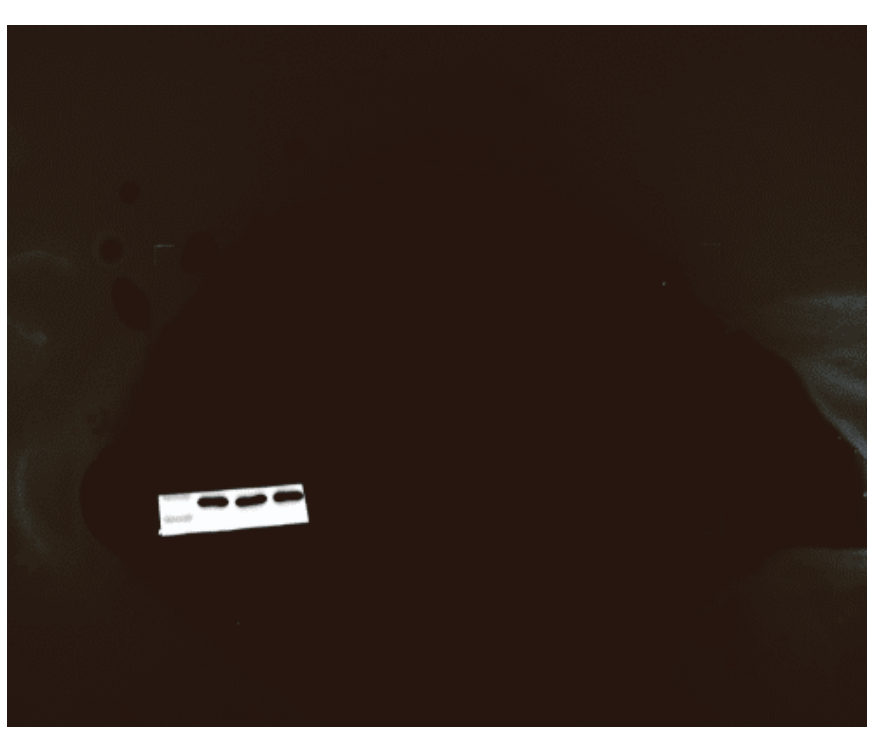
Figure 6N 4E-BP1 (13 kDa)

FIGURE 7D

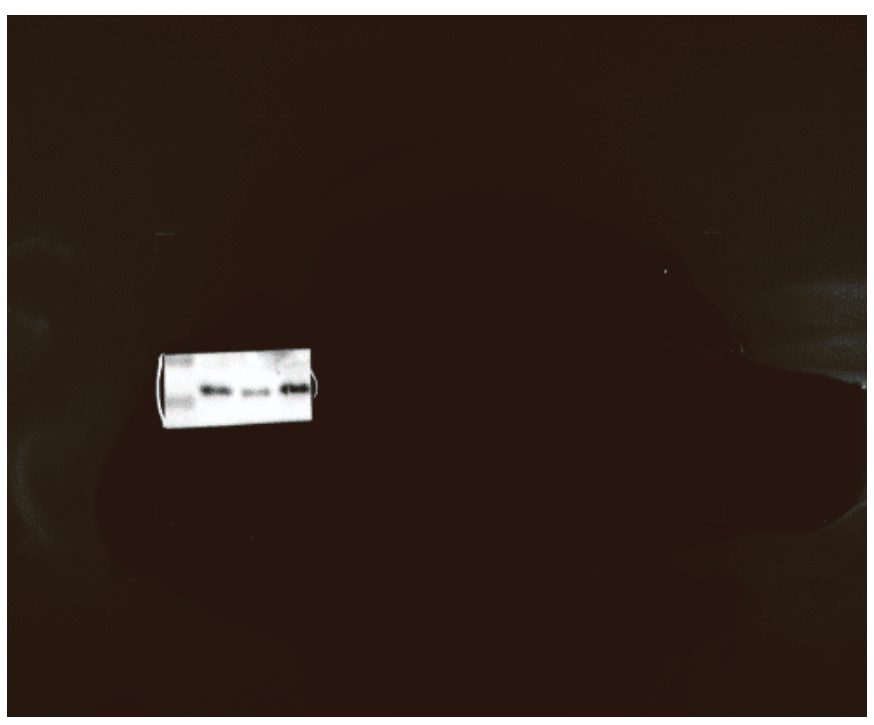

Figure 7D PDX1 (40 kDa)

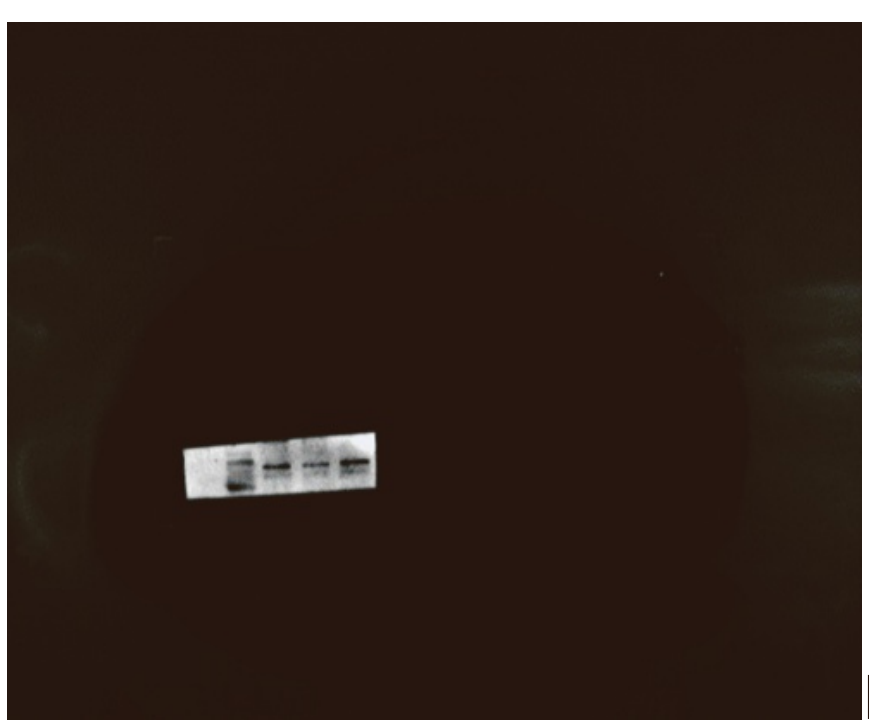

Figure 7D NeuroD1 (50 kDa)

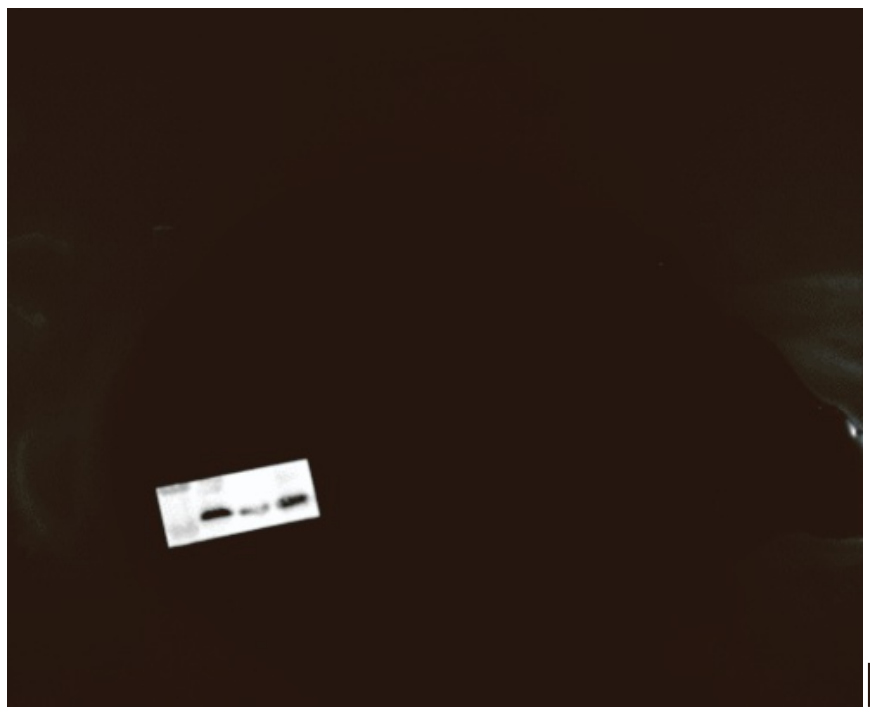
Figure 7D MafA (50 kDa)

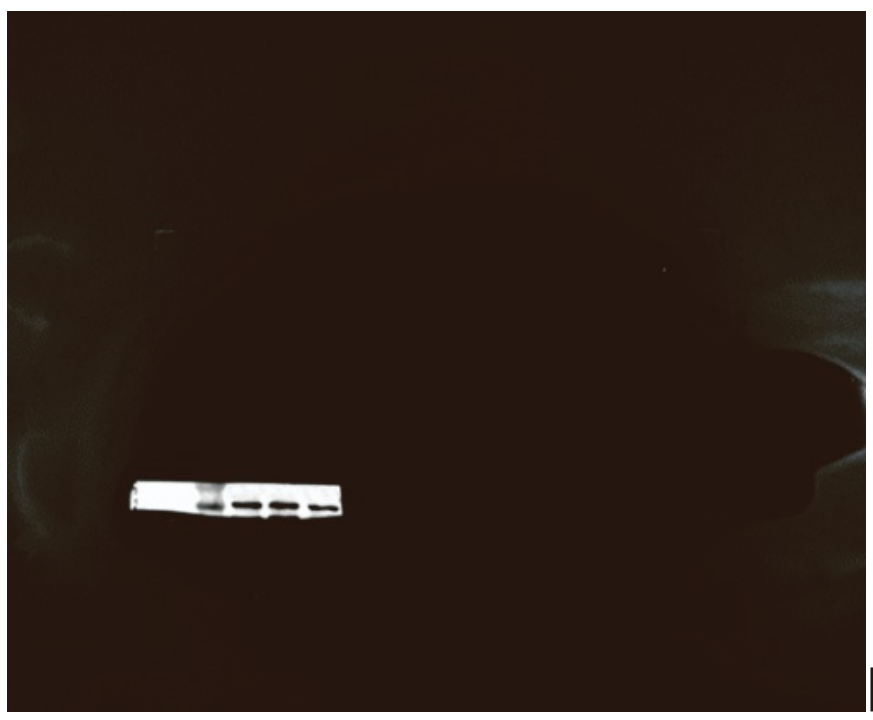
Figure 7D Histone H3 (15 kDa)

SUPPLEMENT FIGURE 2C

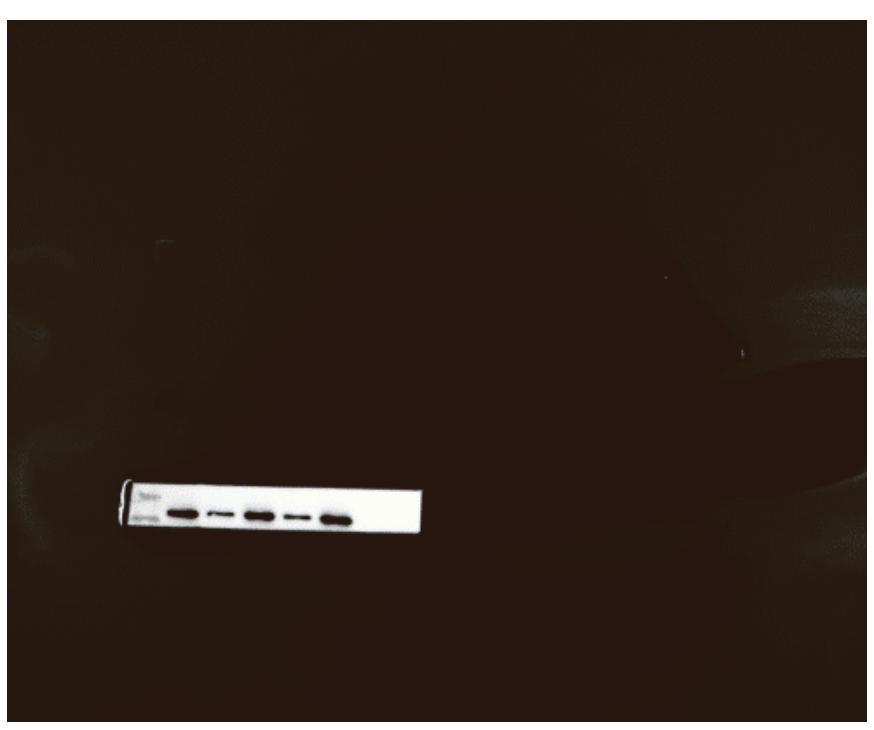
Supplement Figure 2C p-Akt (60 kDa)

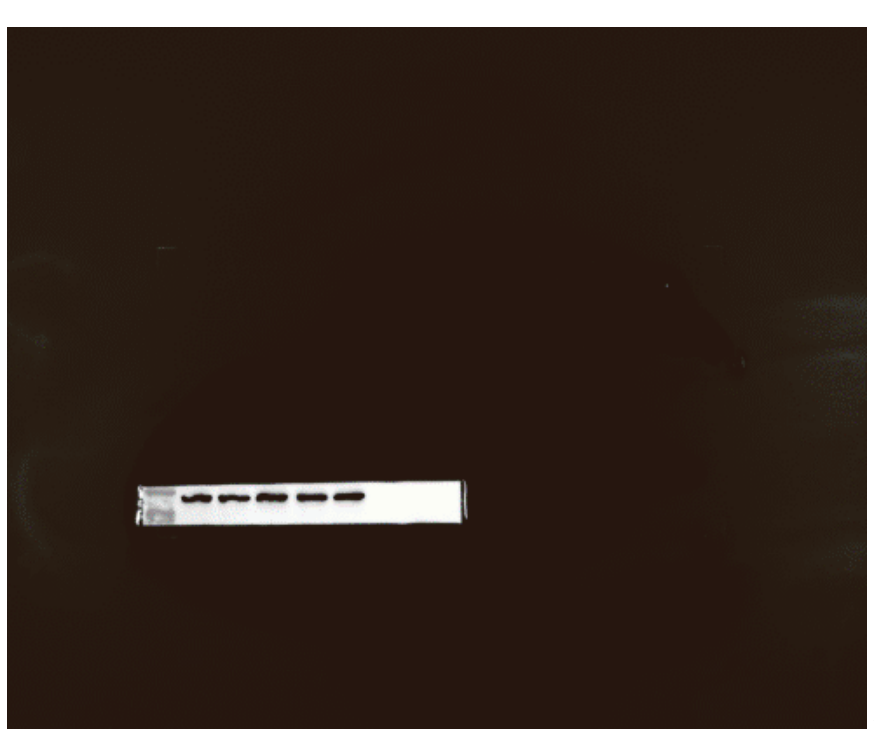
Supplement Figure 2C Akt (60 kDa)

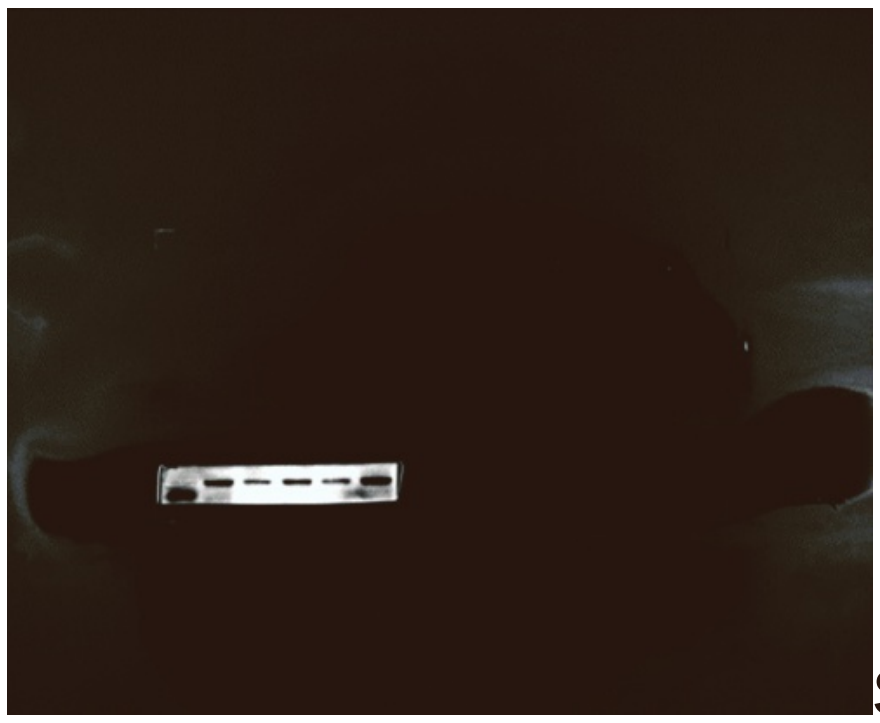

Supplement Figure 2C p-mTOR (~286 kDa)

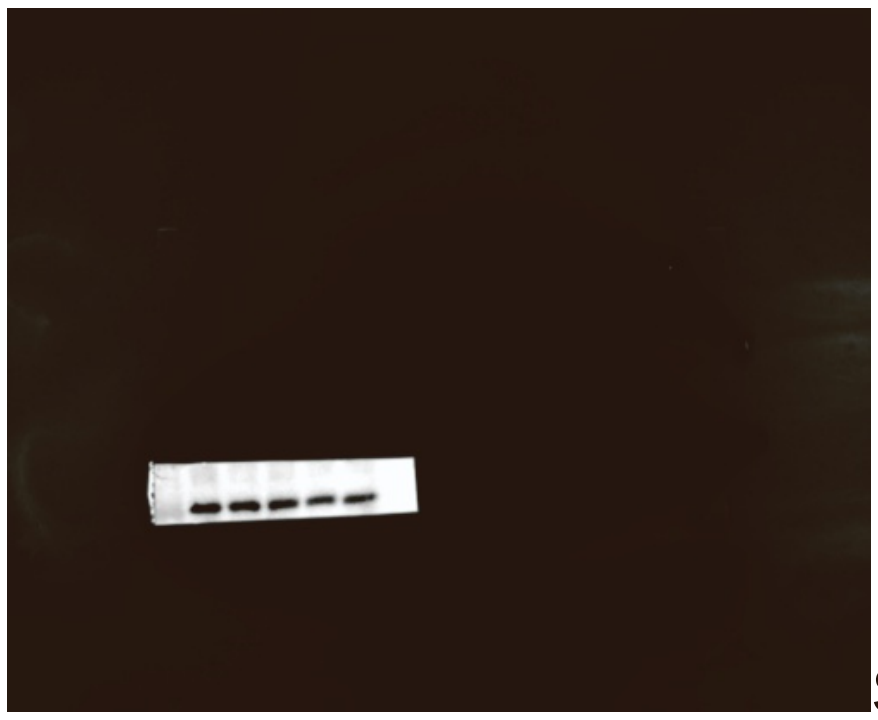

Supplement Figure 2C mTOR (~286 kDa)

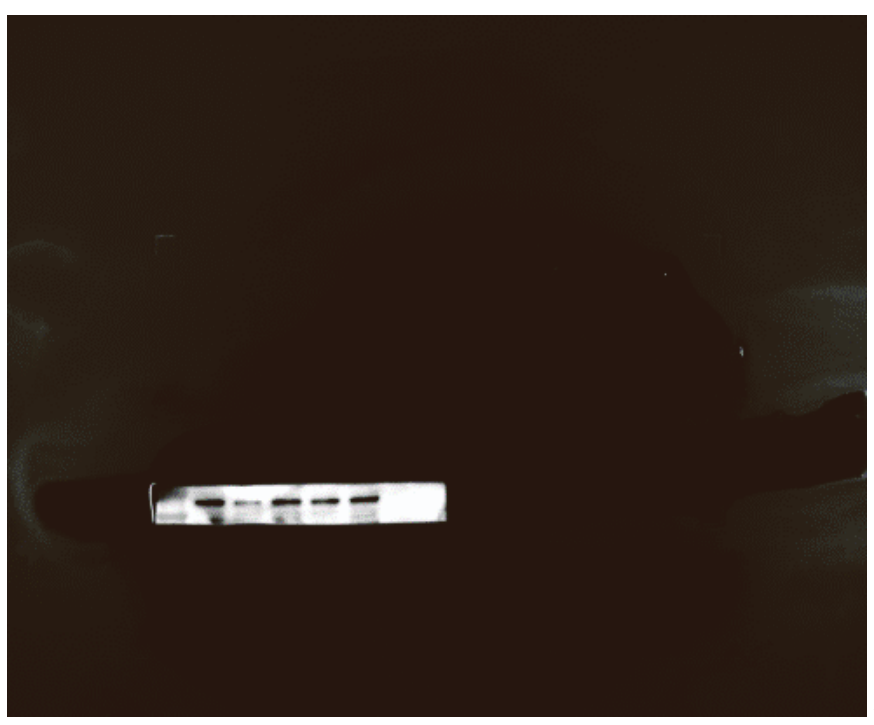

Supplement Figure 2C PDX-1 (40 kDa)

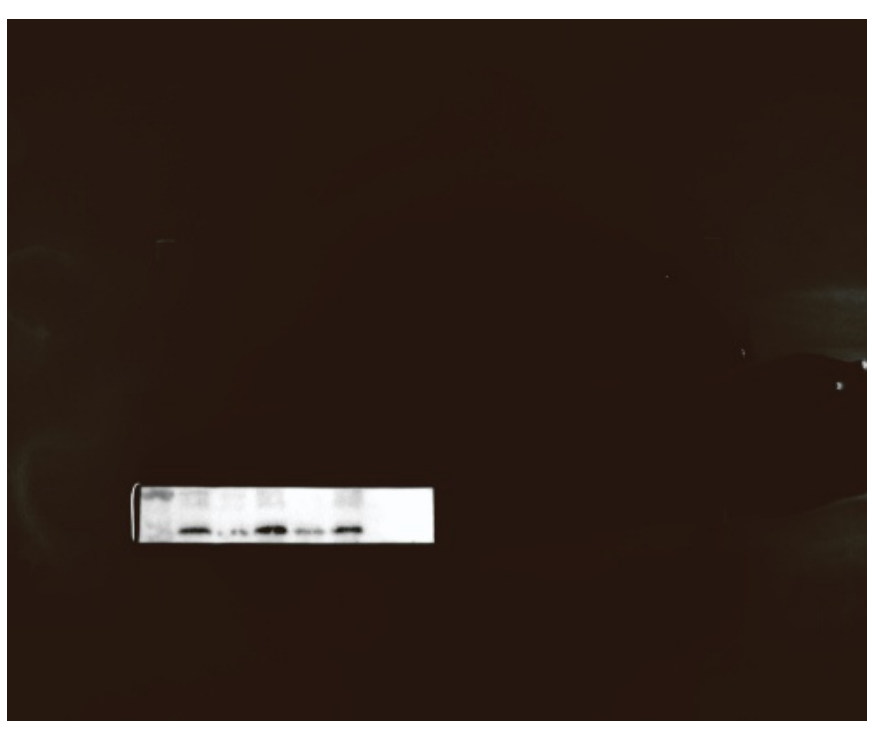

Supplement Figure 2C NeuroD1 (50 kDa)

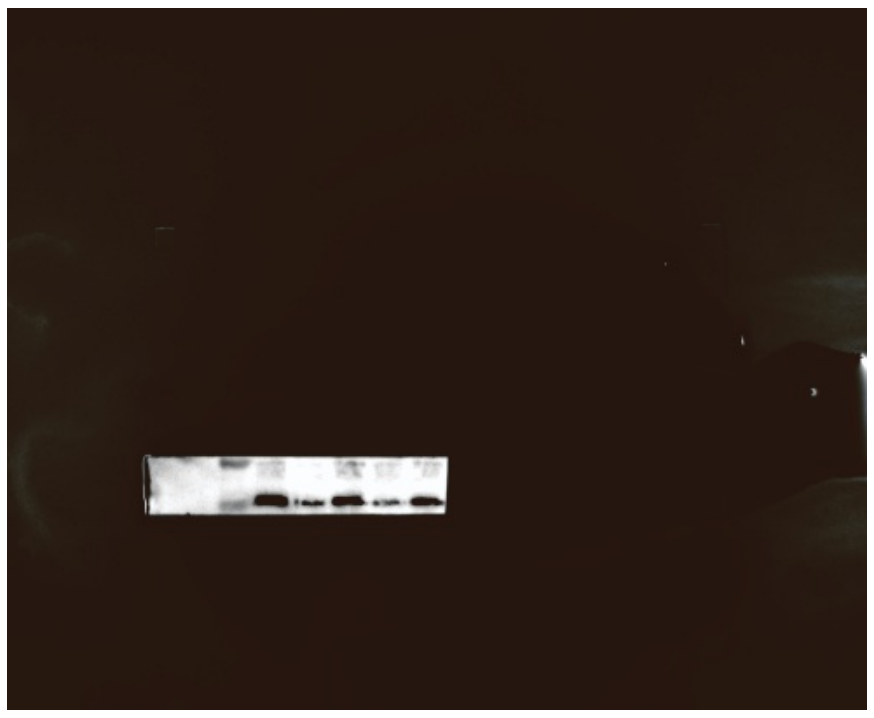

Supplement Figure 2C MafA (50 kDa)

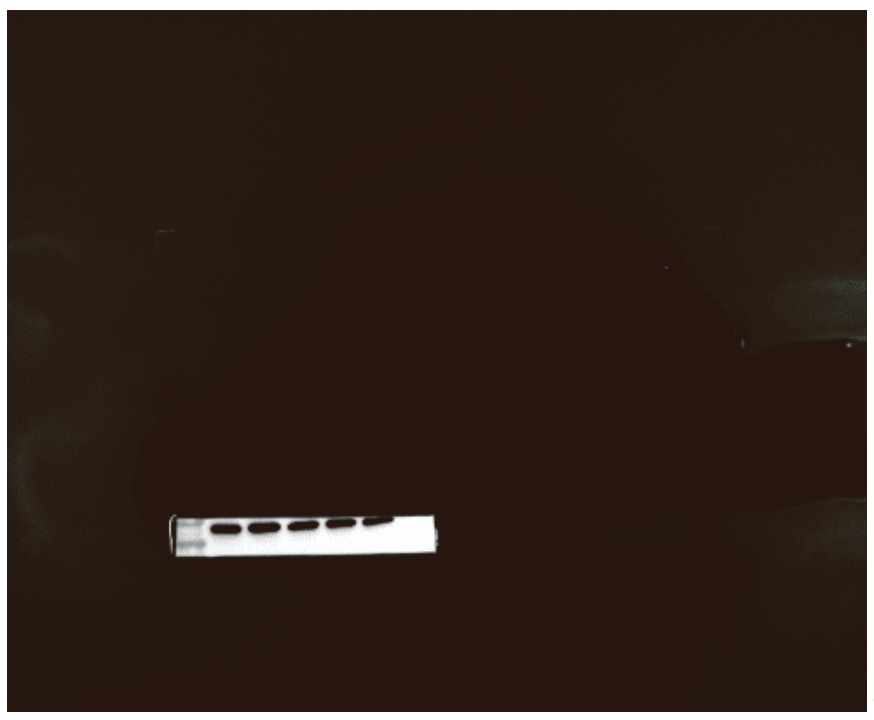

Supplement Figure 2C β-actin (42 kDa)

Data S2: Original microscopy images corresponding to the representative microscopy panels in Figures 4–7.

FIGURE 4A

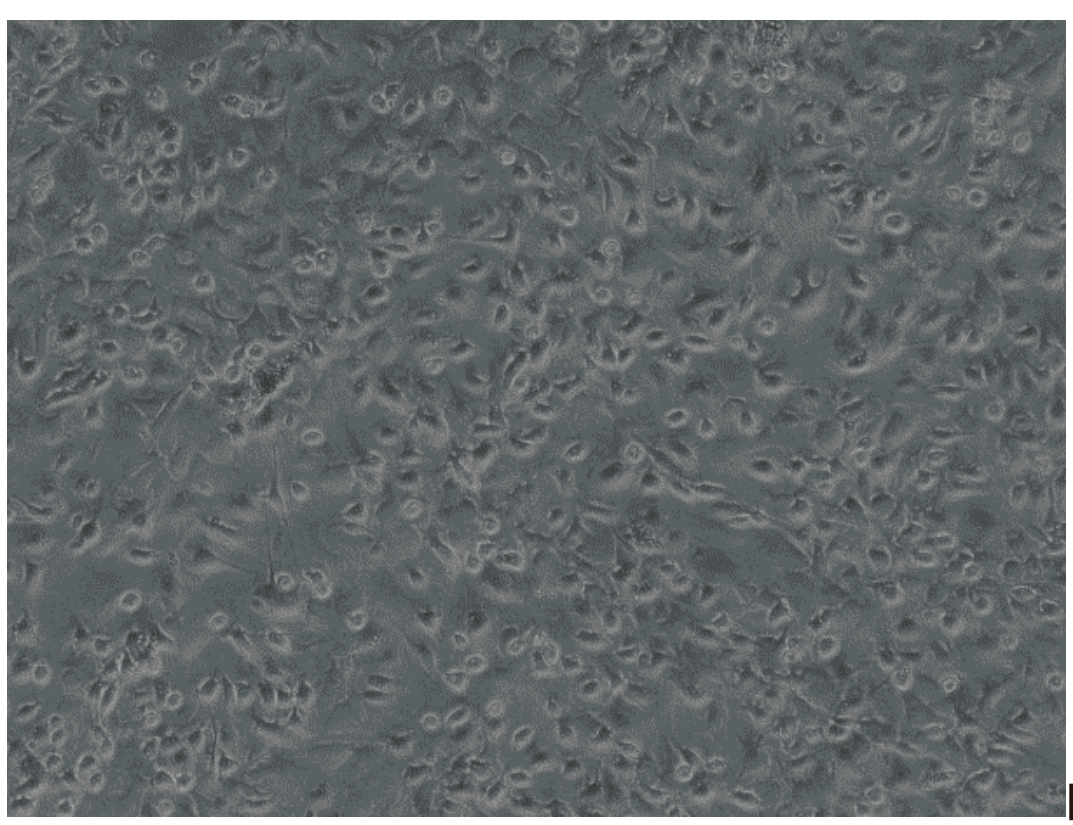

Figure 4A 0 nM

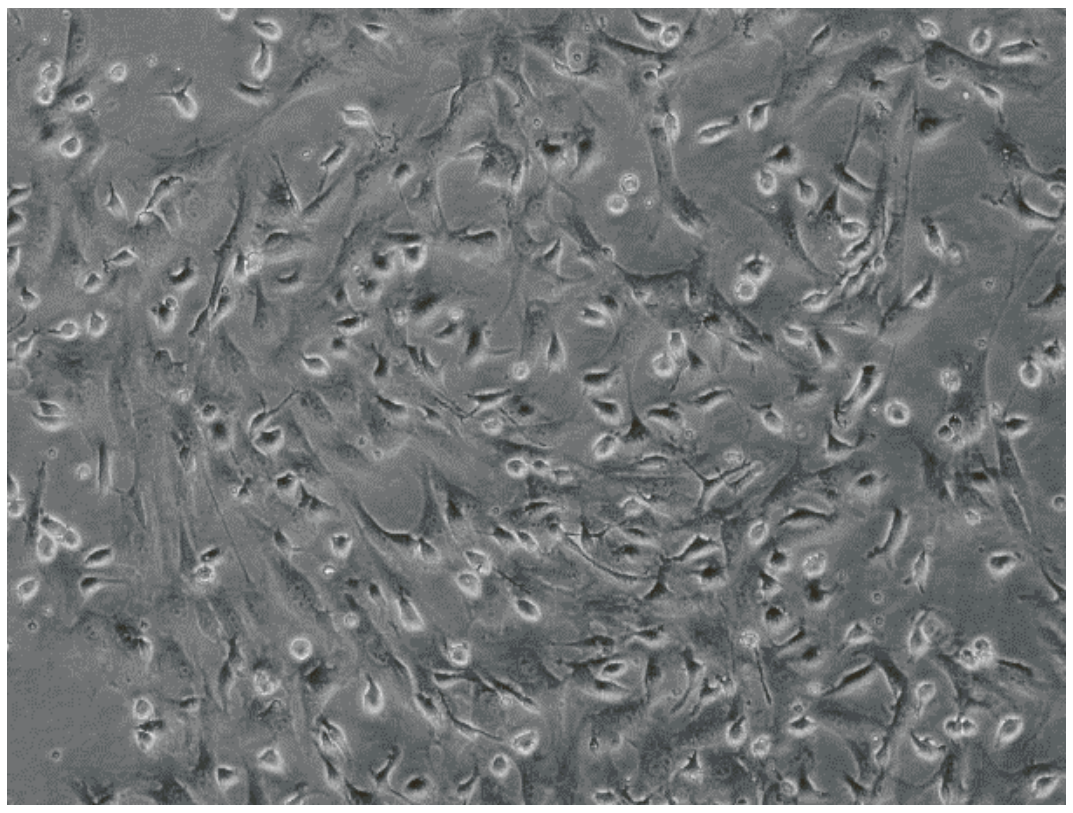

Figure 4A 0.1 nM

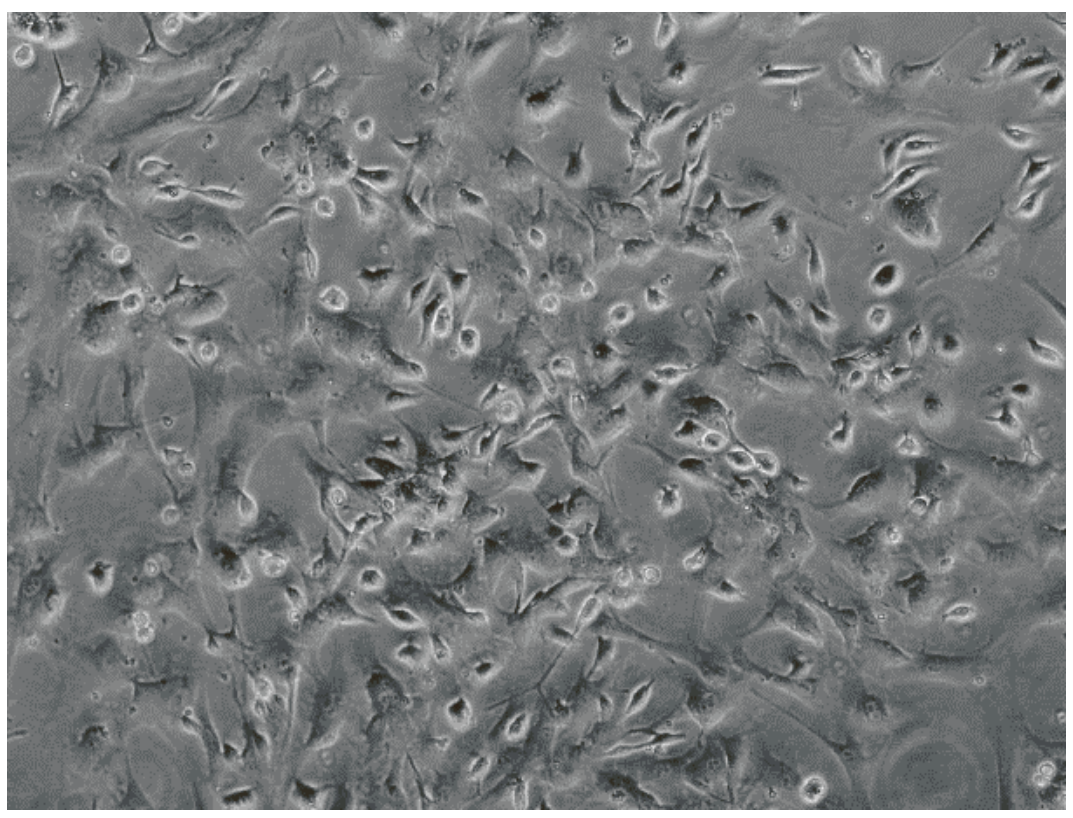

Figure 4A 1 nM

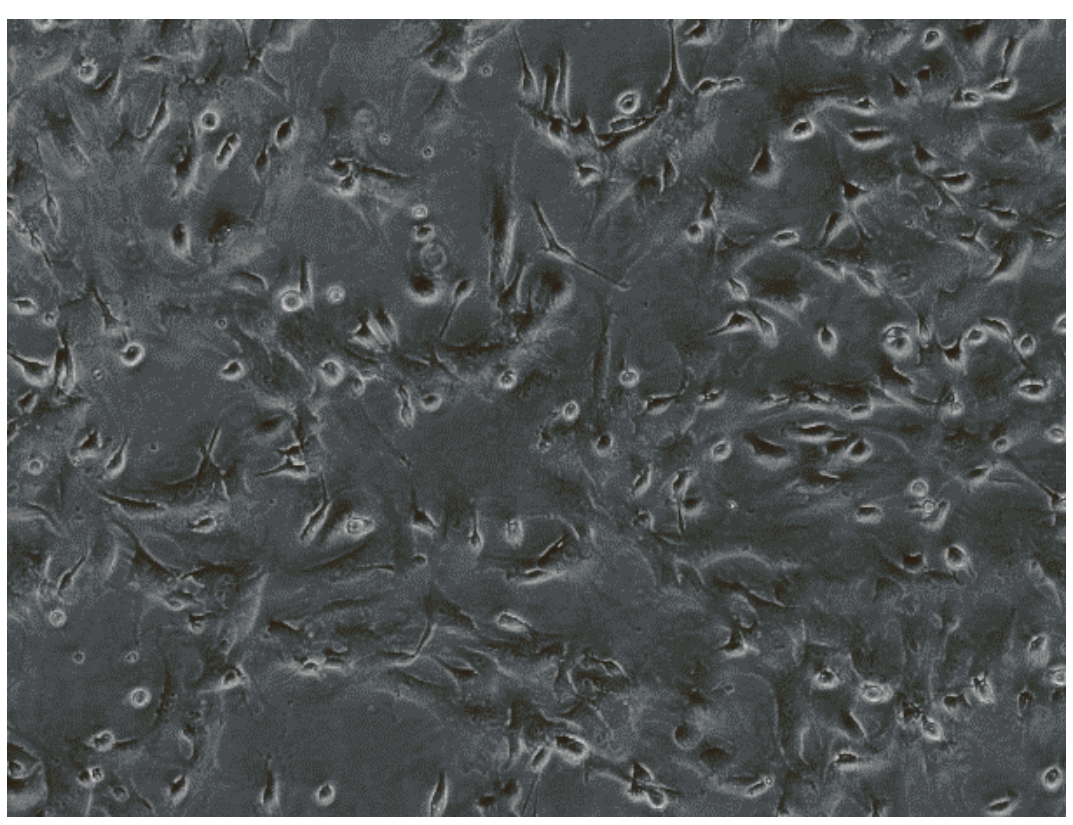

Figure 4A 10 nM

FIGURE 4D

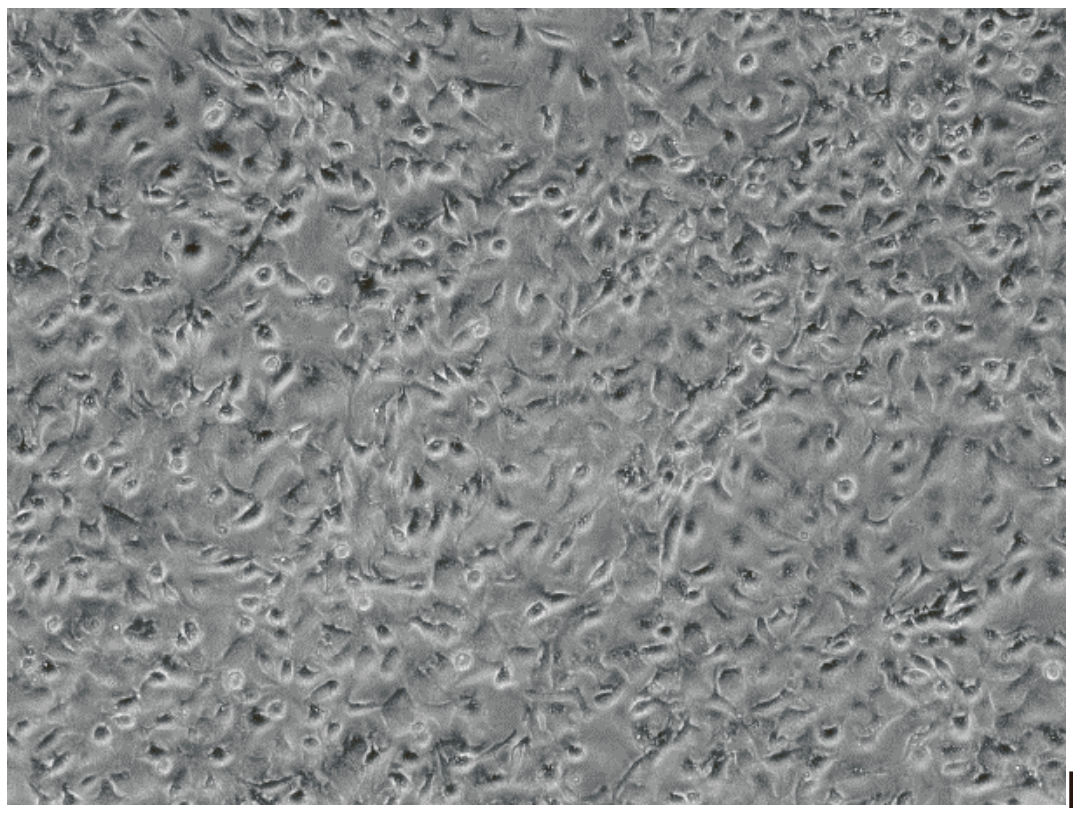
Figure 4D control

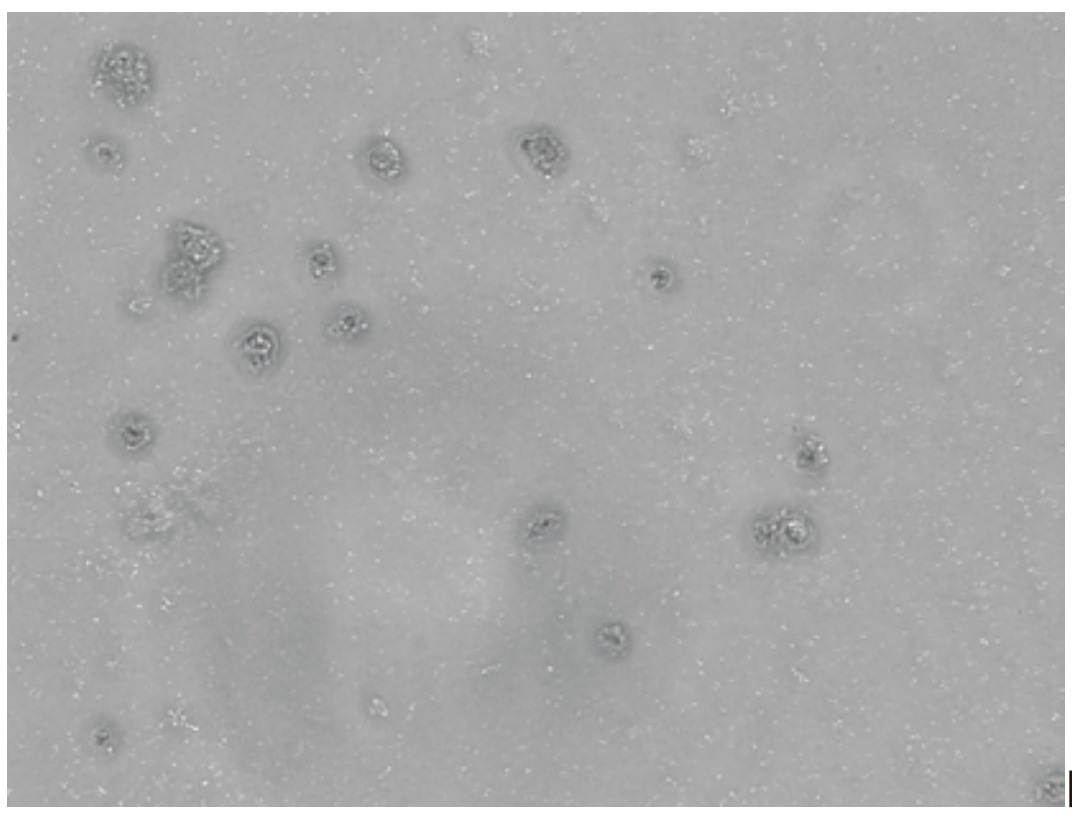
Figure 4D TAC

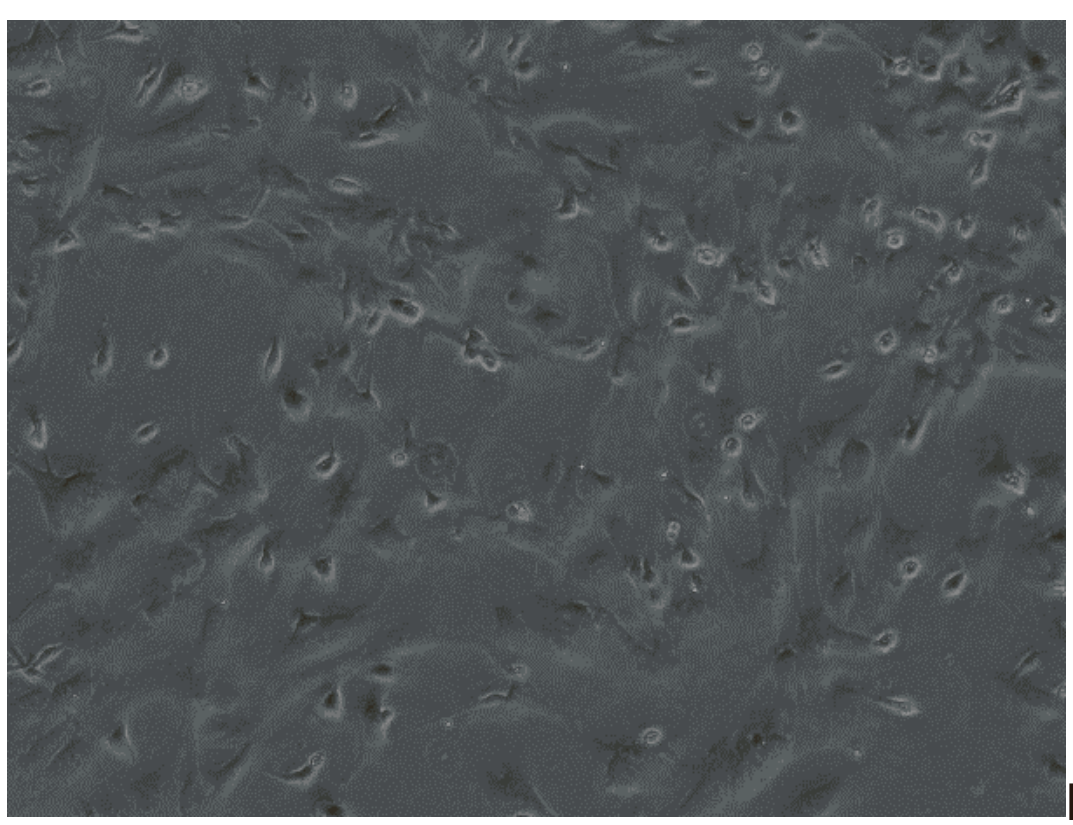
Figure 4D TAC+L-His

FIGURE 5E

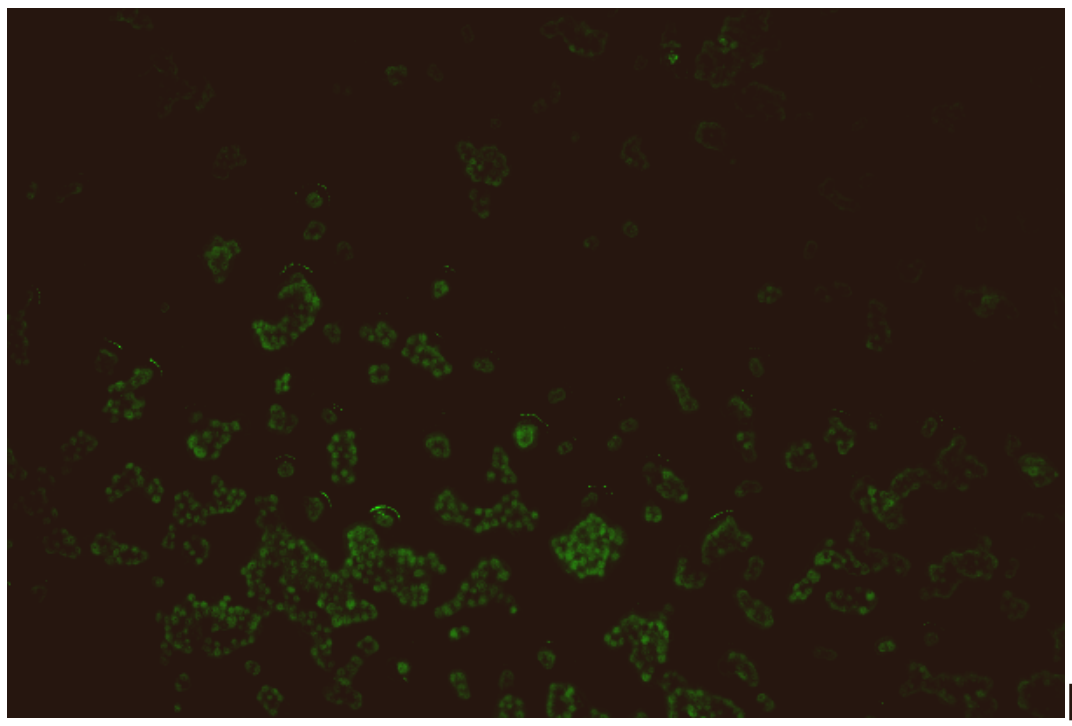
Figure 5E PDX-1 (FITC), control

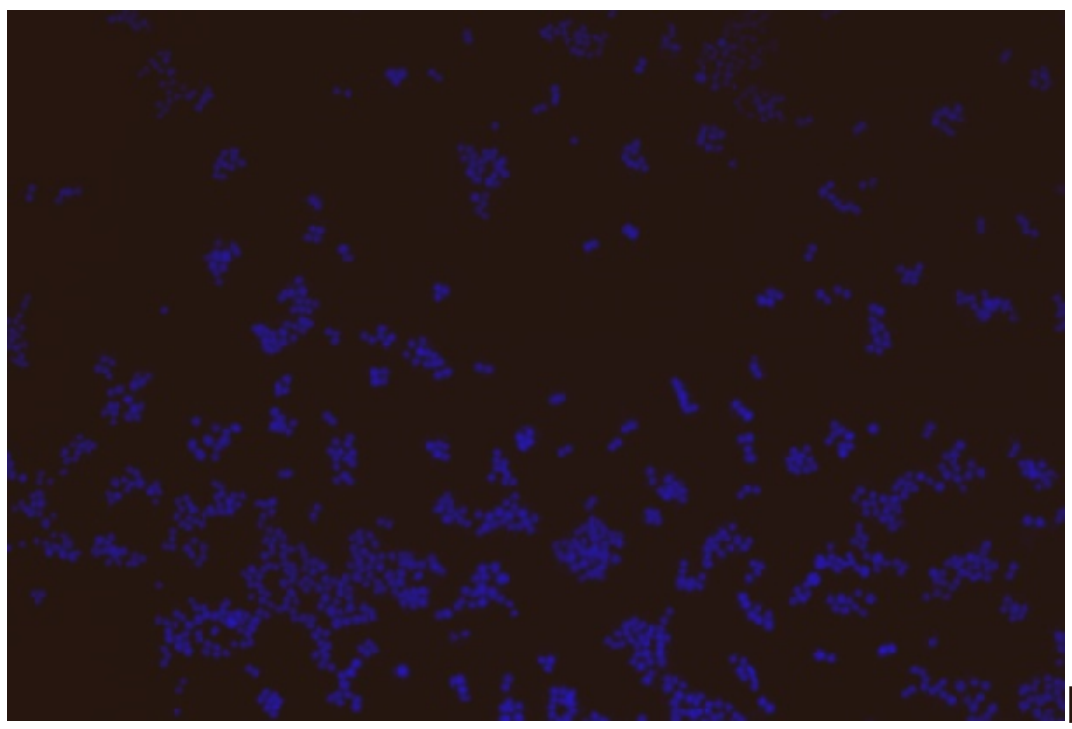
Figure 5E DAPI, control

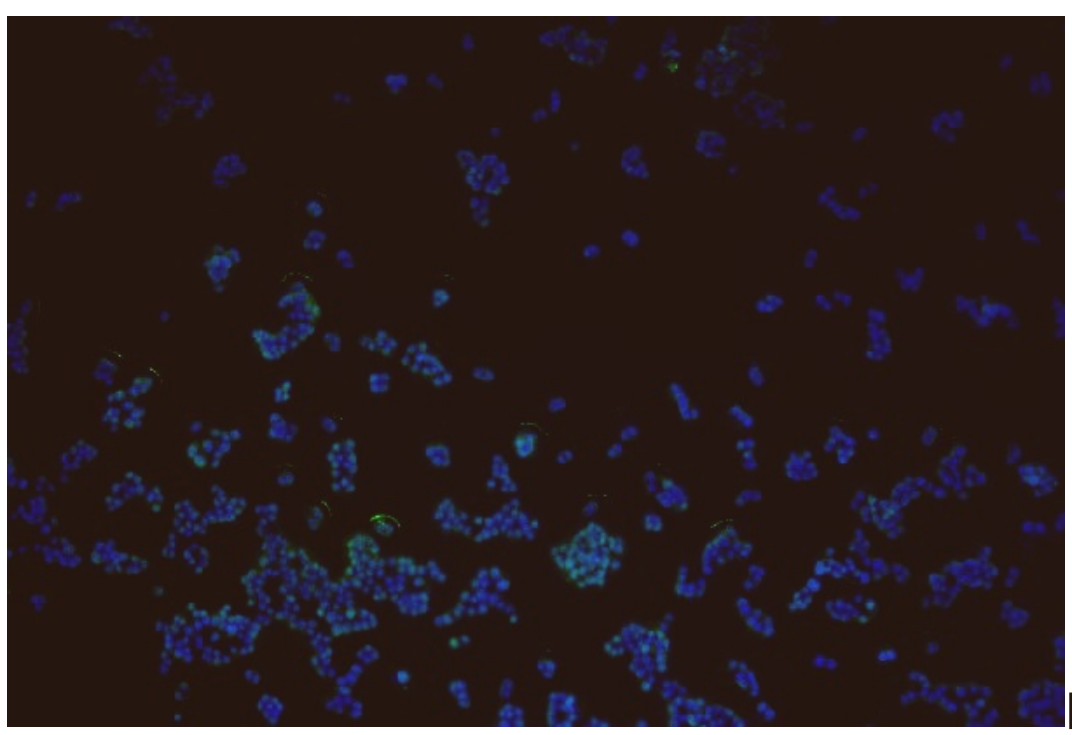
Figure 5E Merge, control

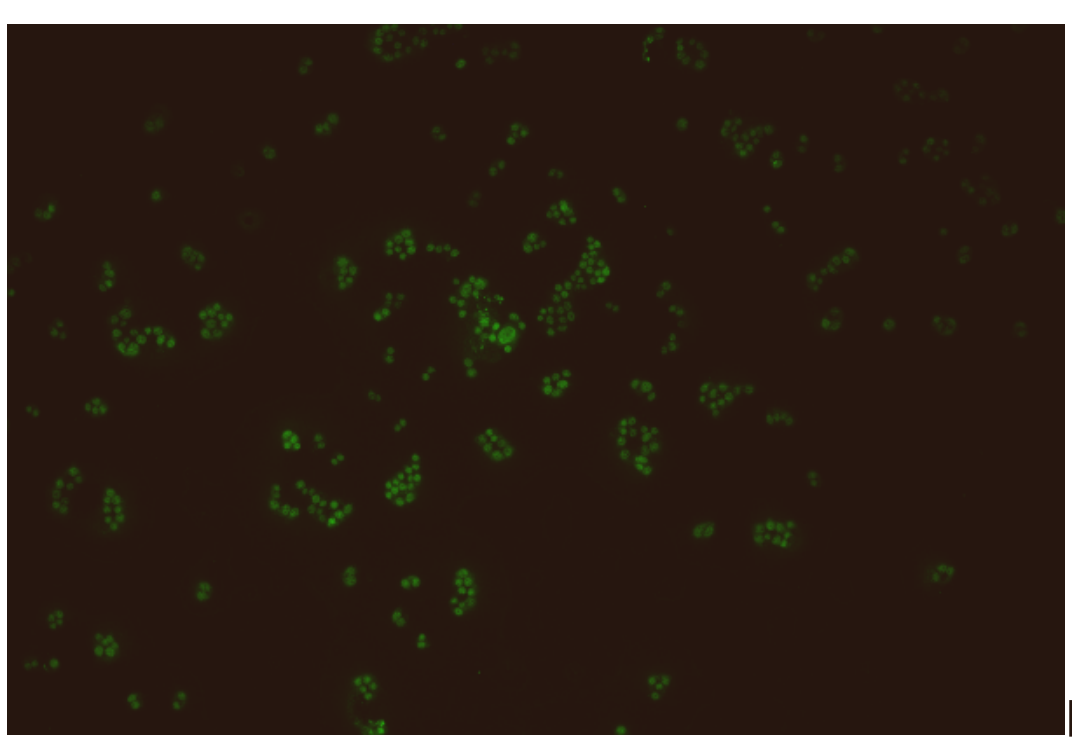
Figure 5E PDX-1 (FITC), TAC

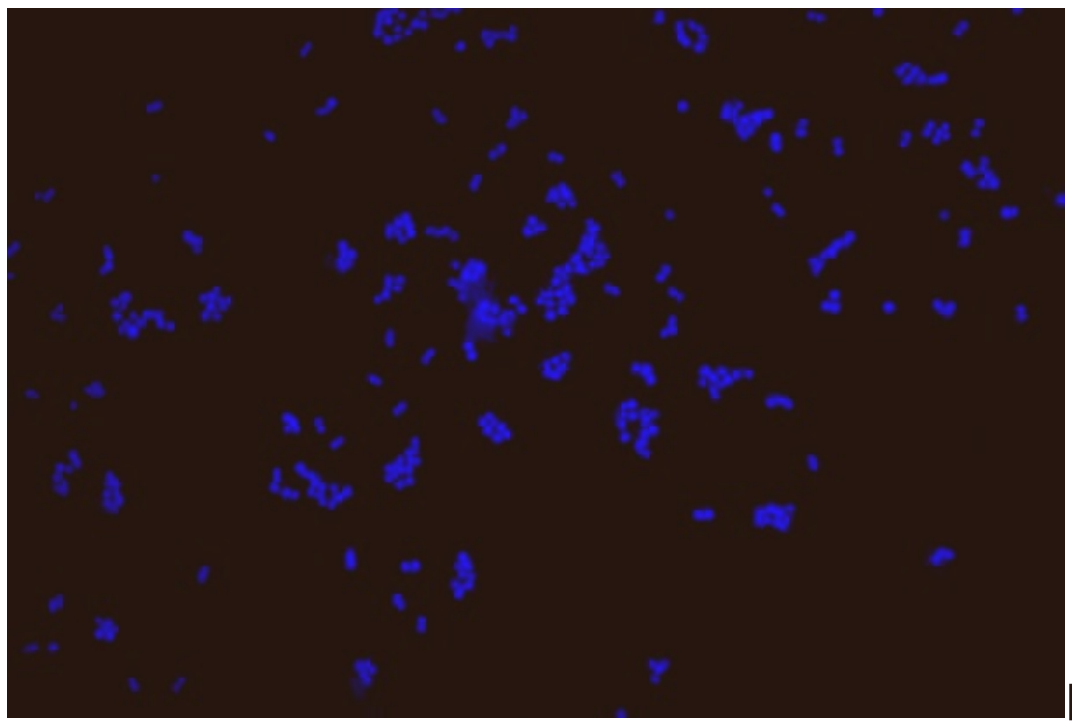
Figure 5E DAPI, TAC

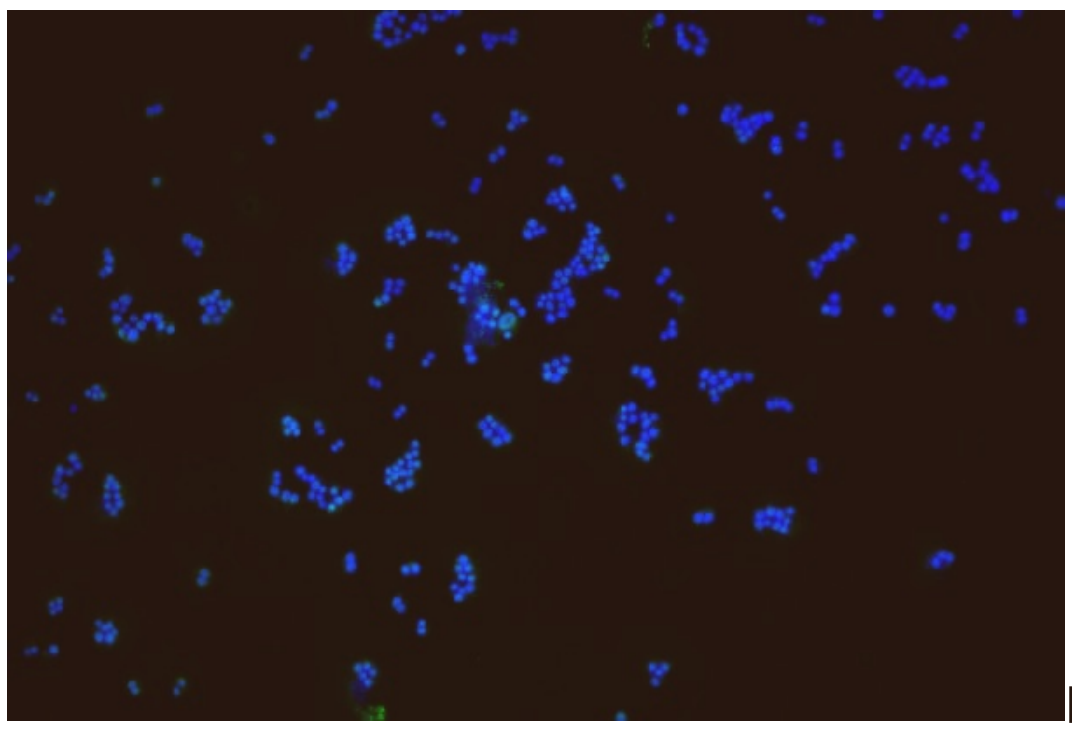
Figure 5E Merge, Merge

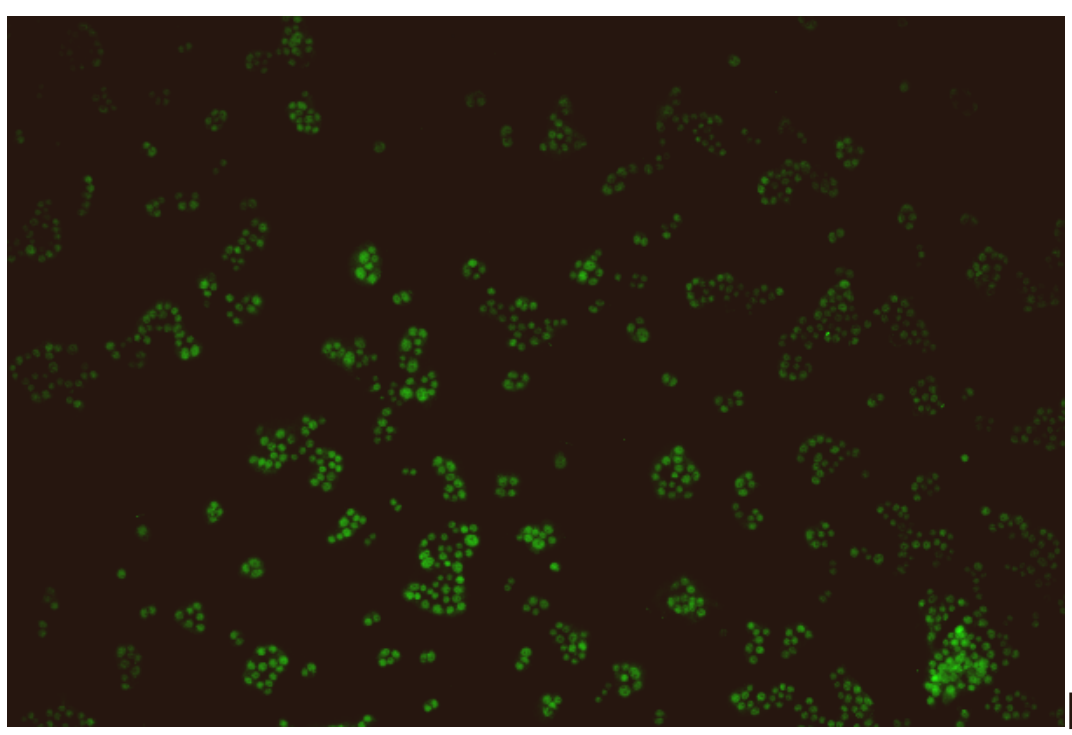
Figure 5E PDX-1 (FITC), TAC+L-His

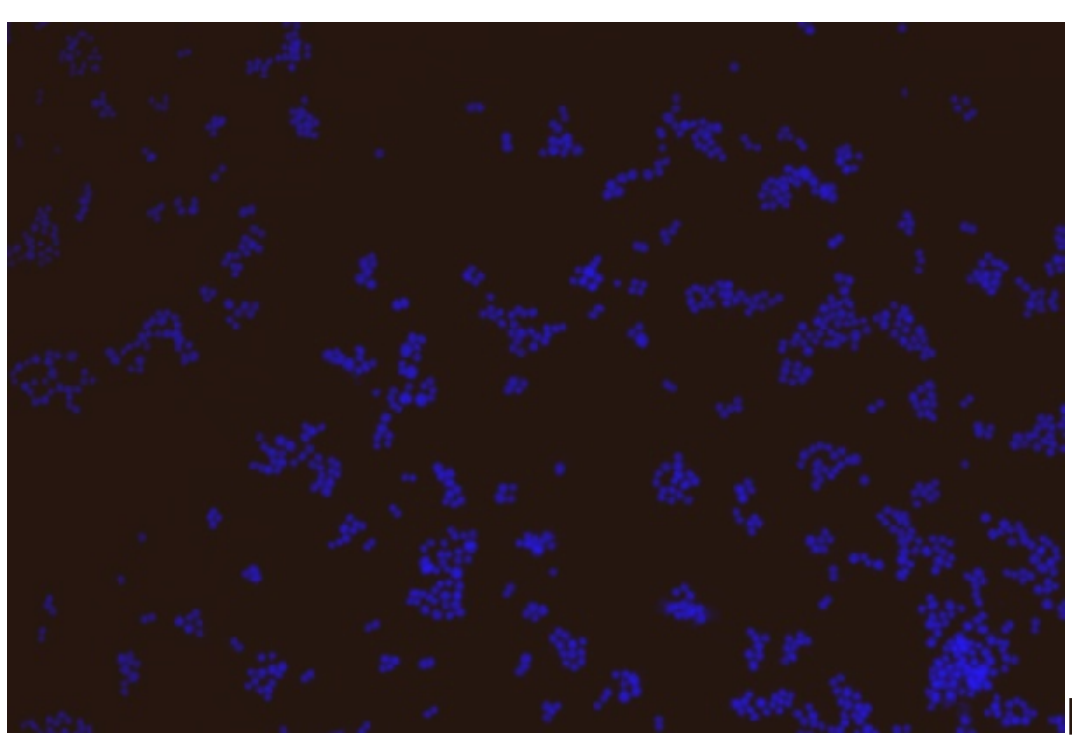
Figure 5E DAPI, TAC+L-His

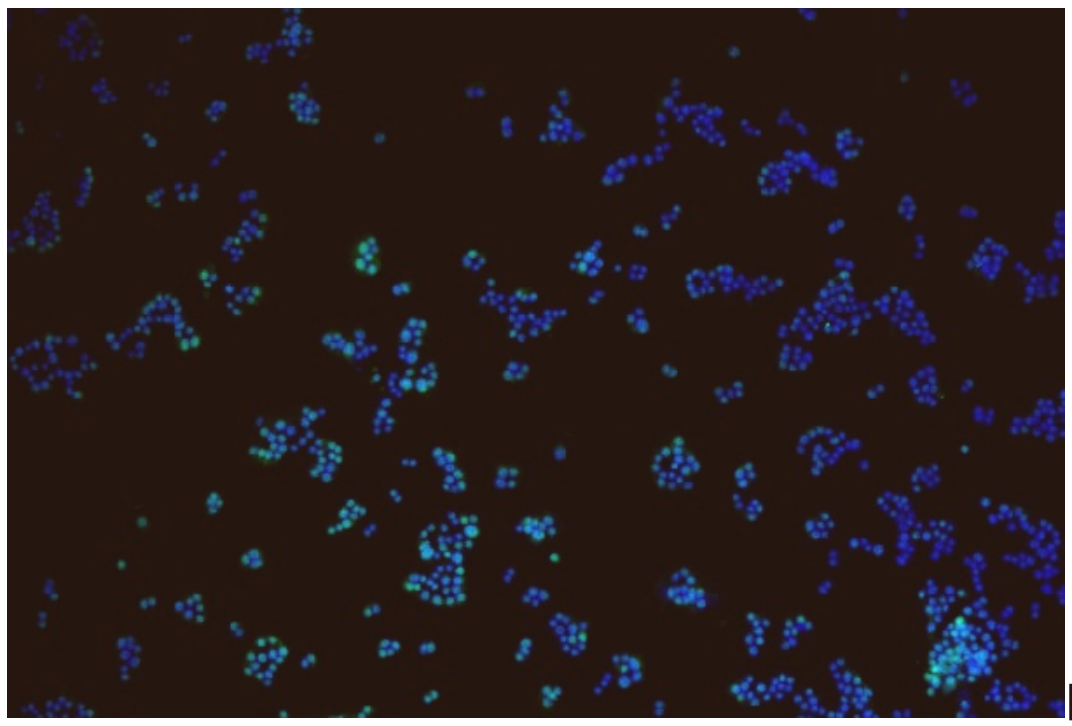
Figure 5E Merge, TAC+L-His

FIGURE 5G

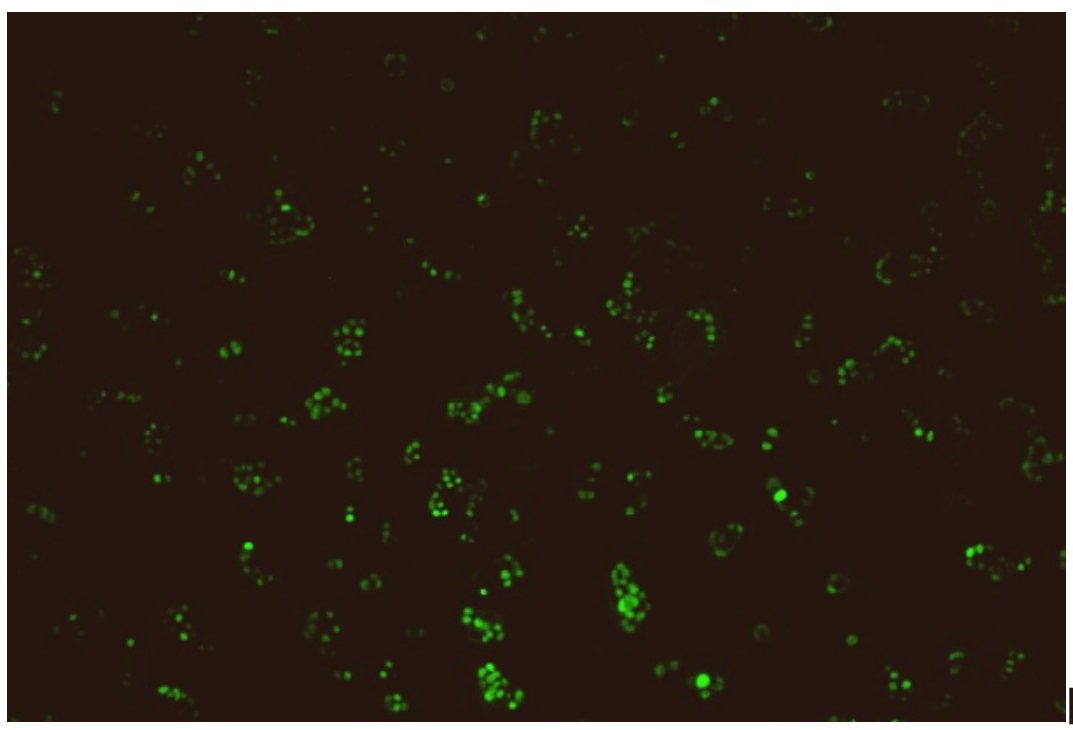
Figure 5G NeuroD1 (FITC), control

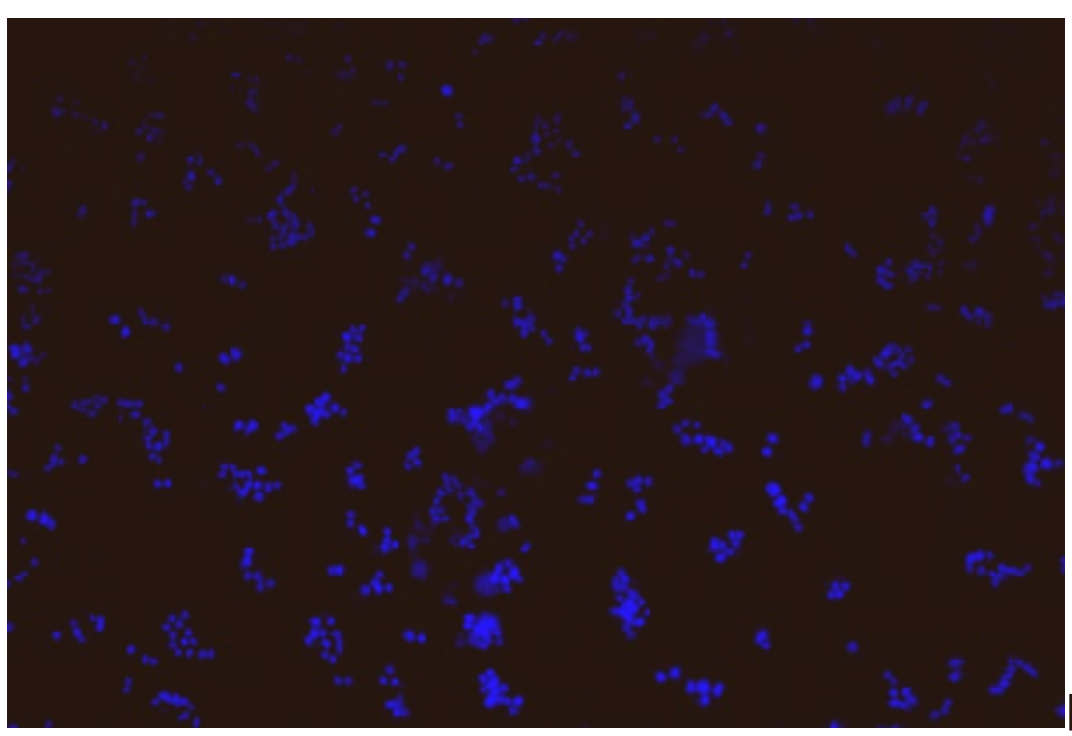
Figure 5G DAPI, control

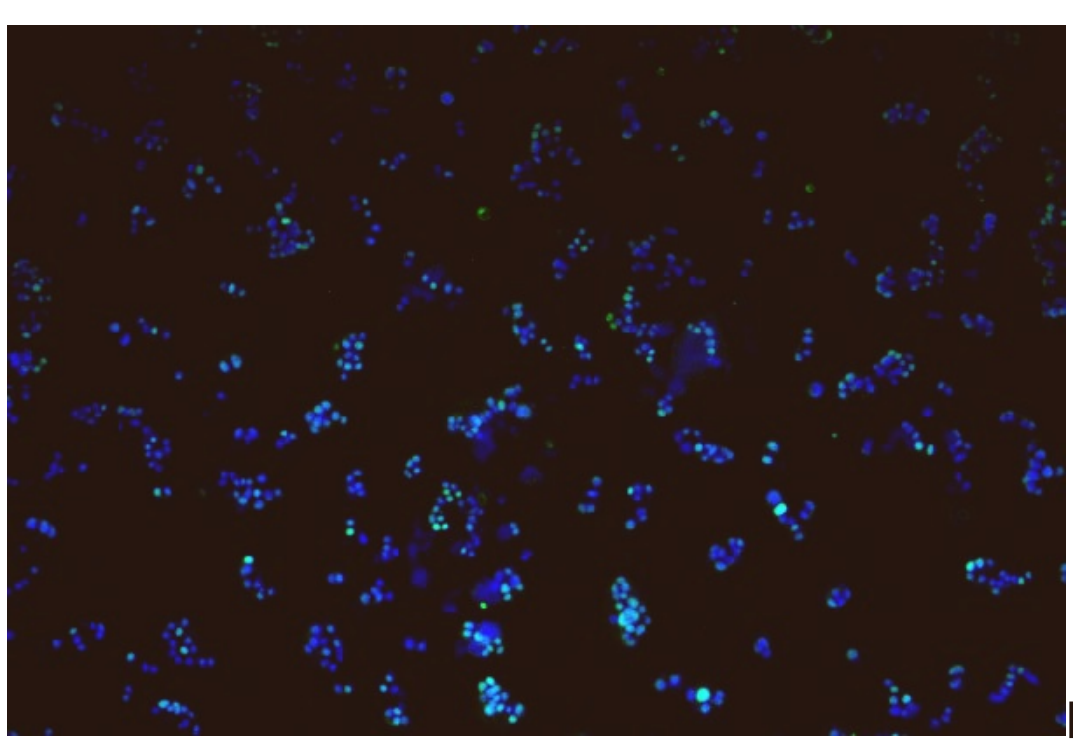
Figure 5G Merge, control

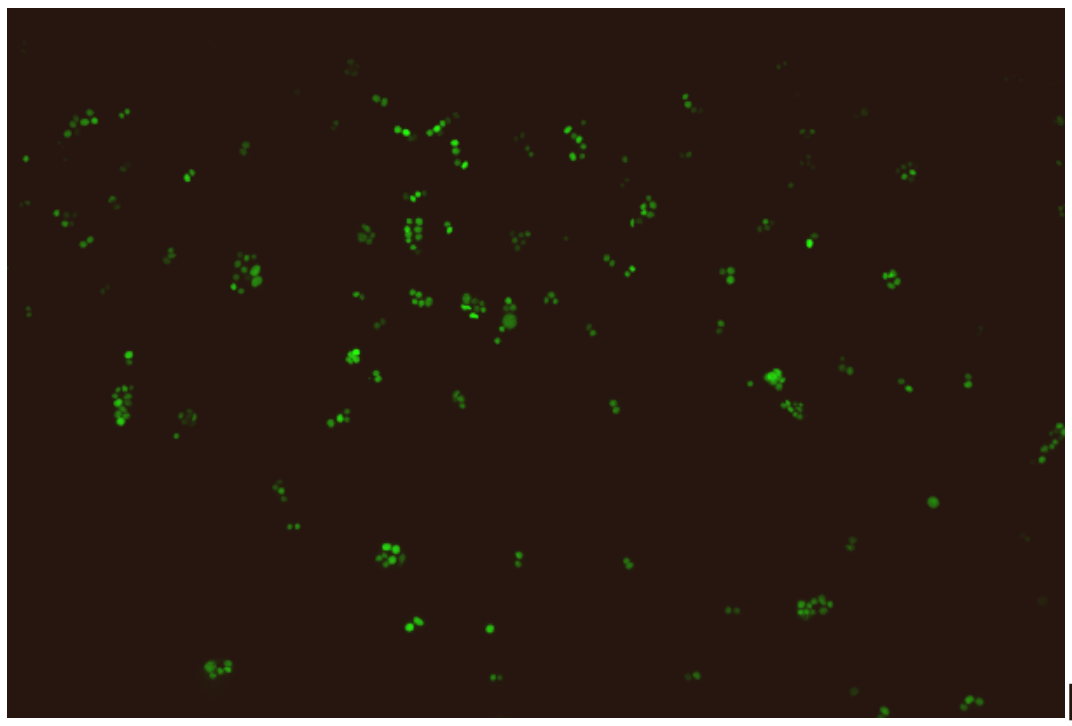
127 Figure 5G NeuroD1 (FITC), TAC

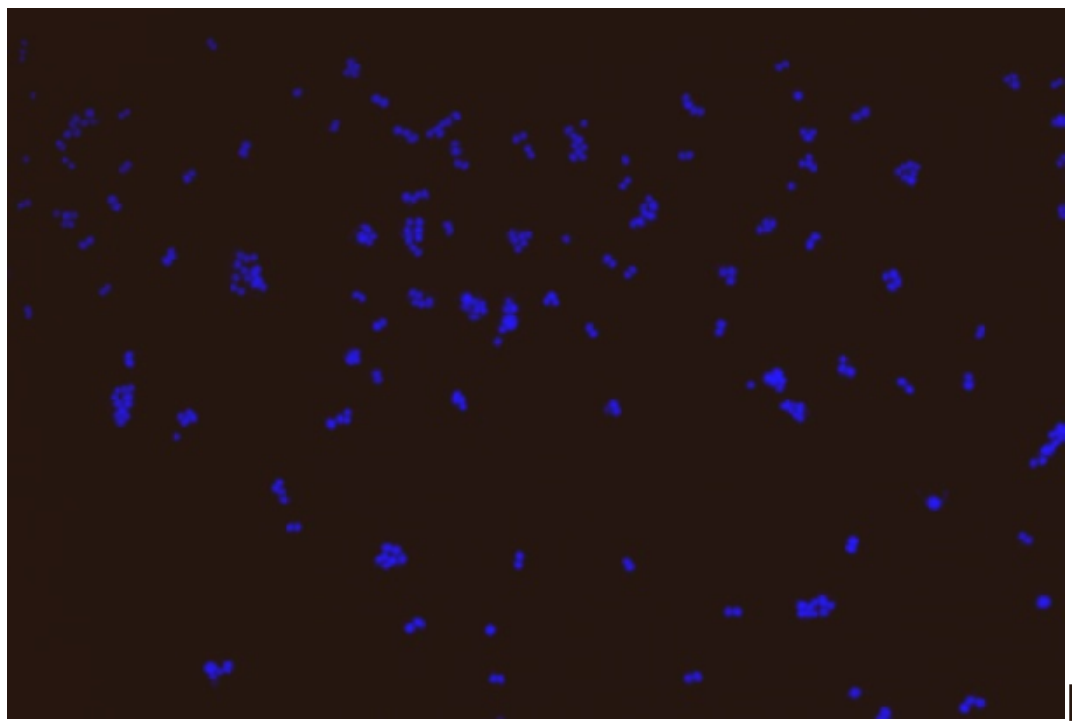
128 Figure 5G DAPI, TAC

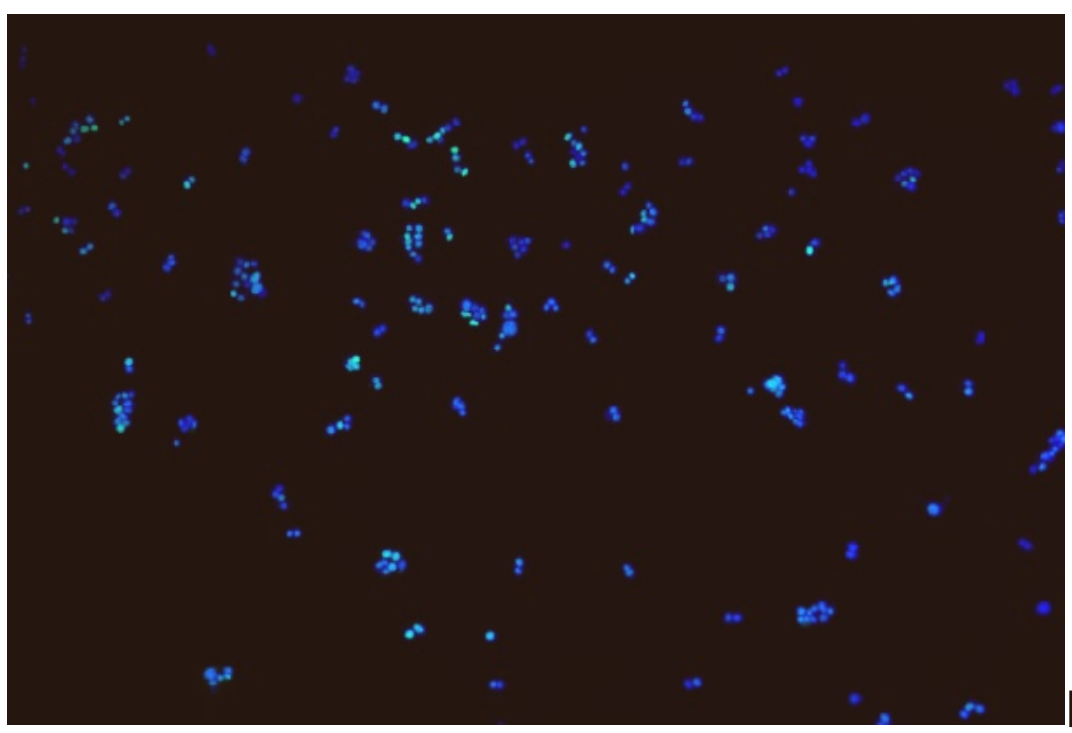
129 Figure 5G Merge, TAC

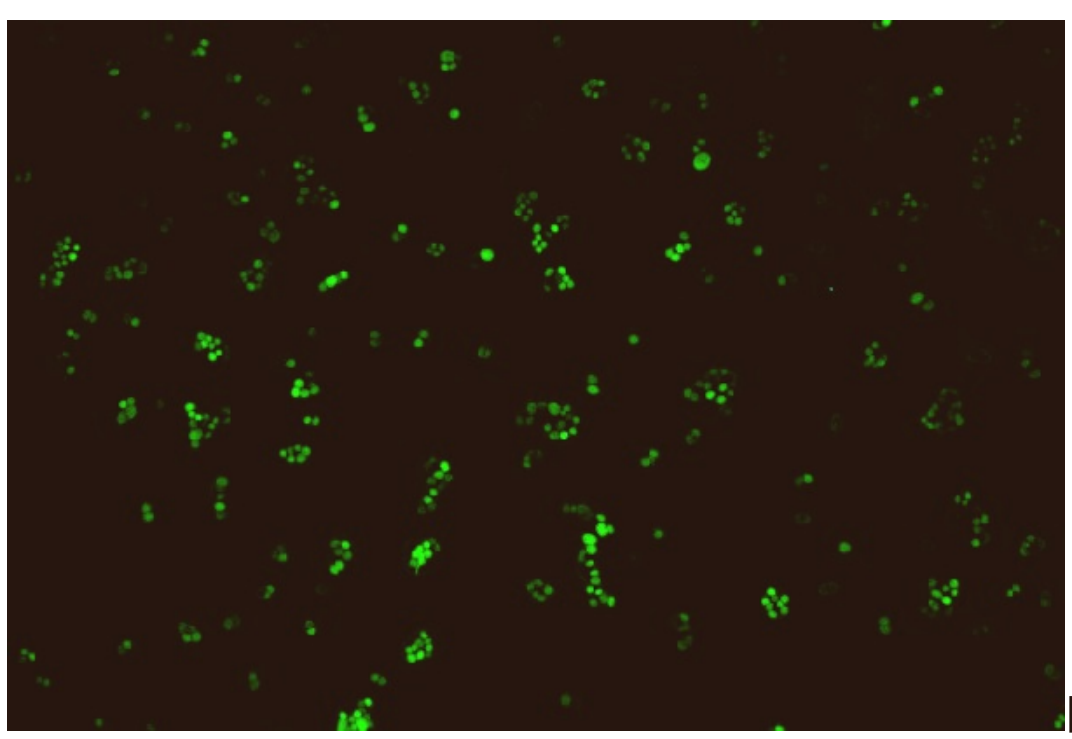
130 Figure 5G NeuroD1 (FITC), TAC+L-His

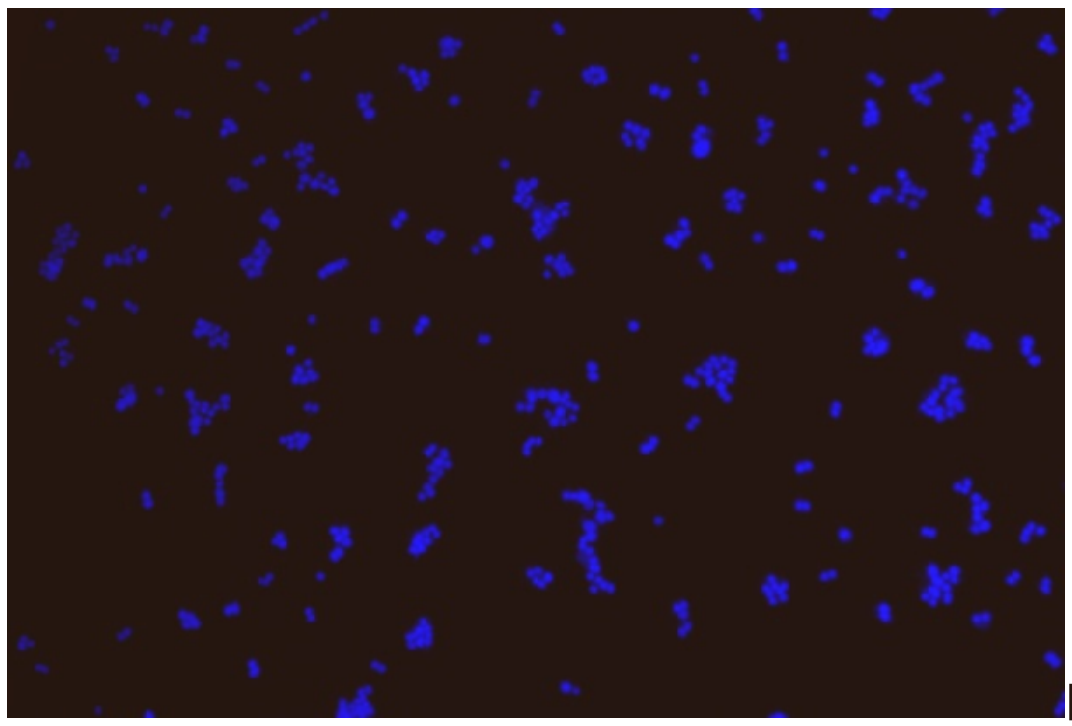
Figure 5G DAPI, TAC+L-His

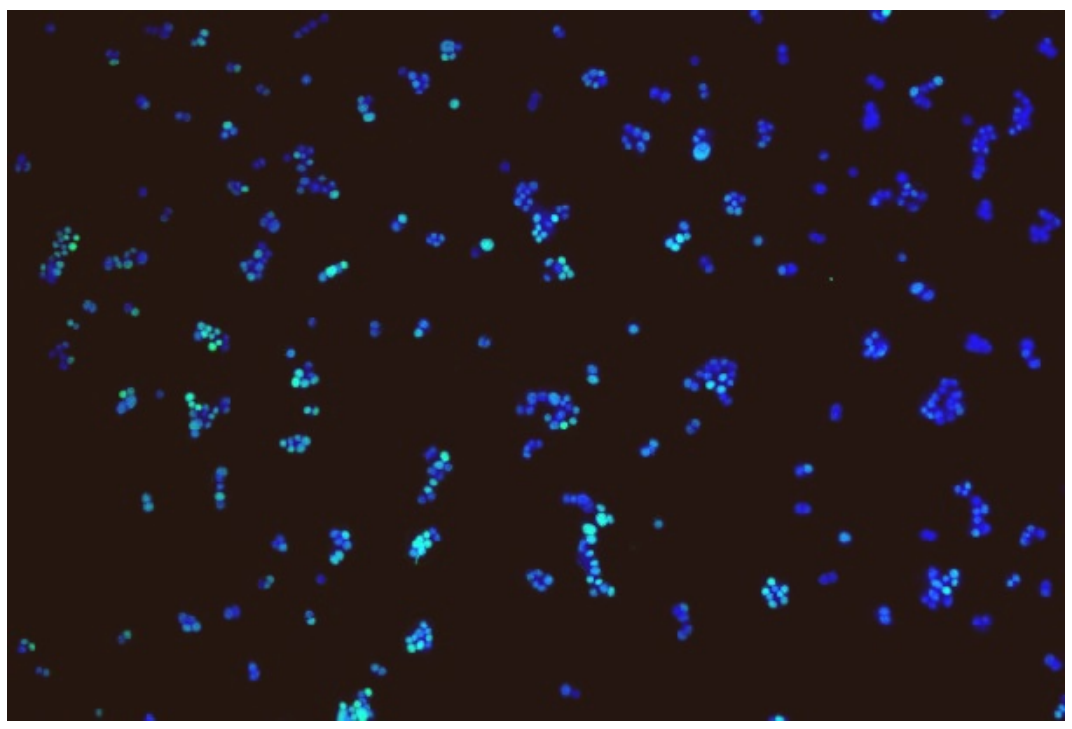
Figure 5G Merge, TAC+L-His

FIGURE 5I

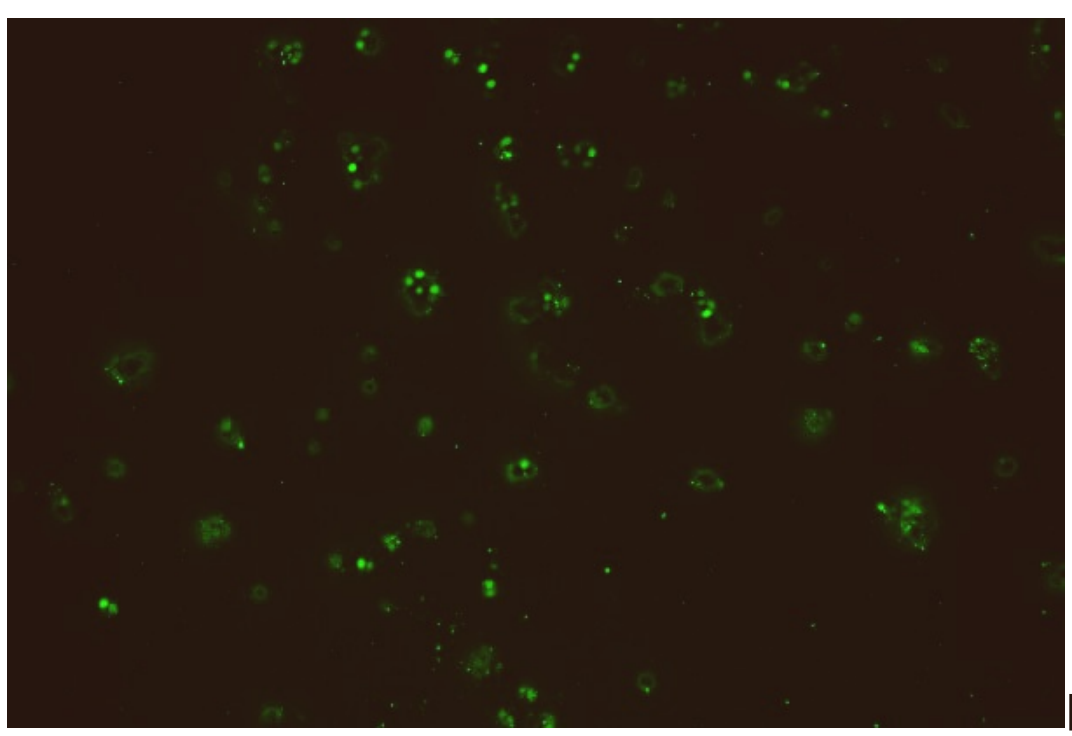
Figure 5I MafA (FITC), control

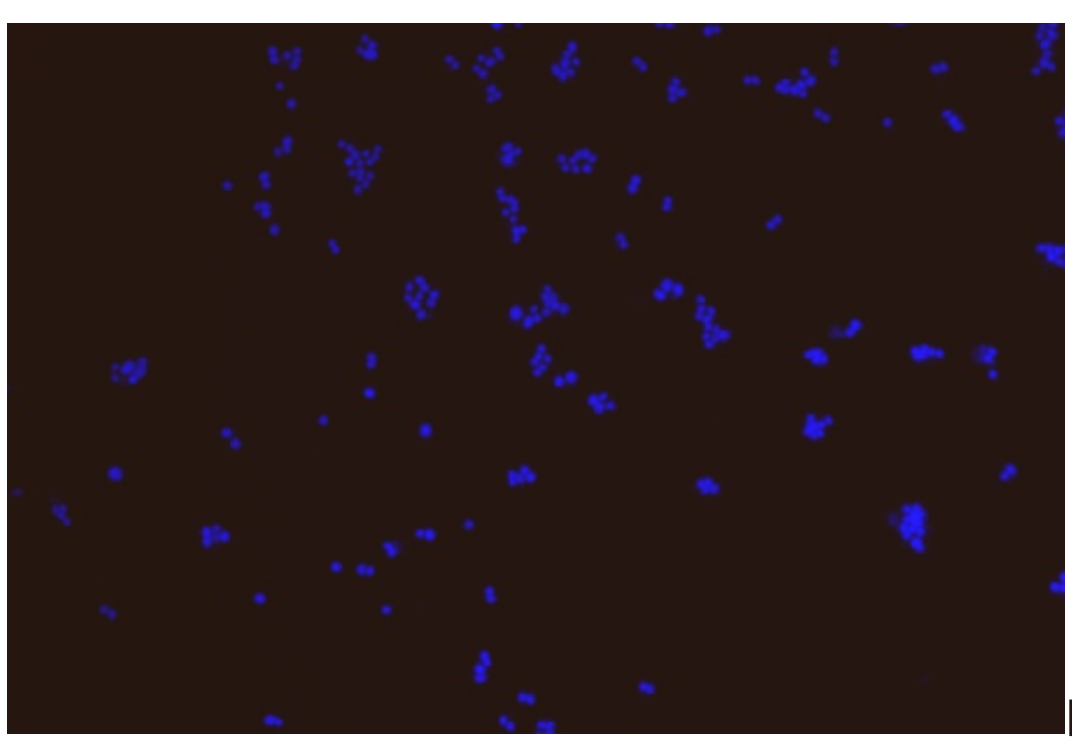
Figure 5I DAPI, control

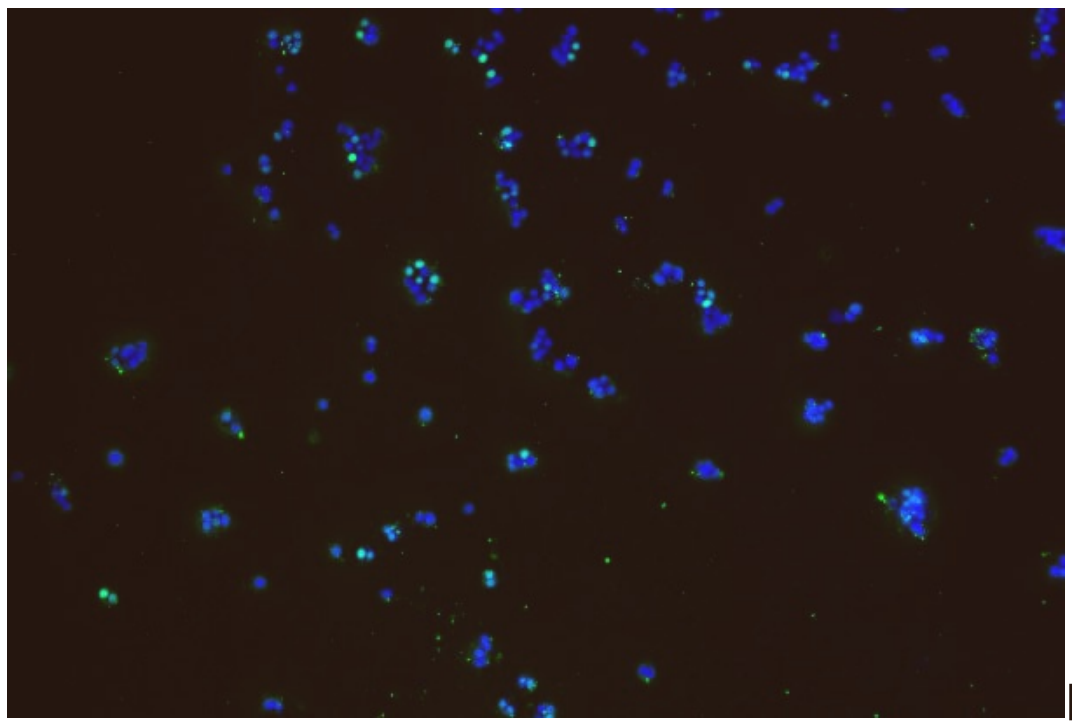

136 Figure 5I merge, control

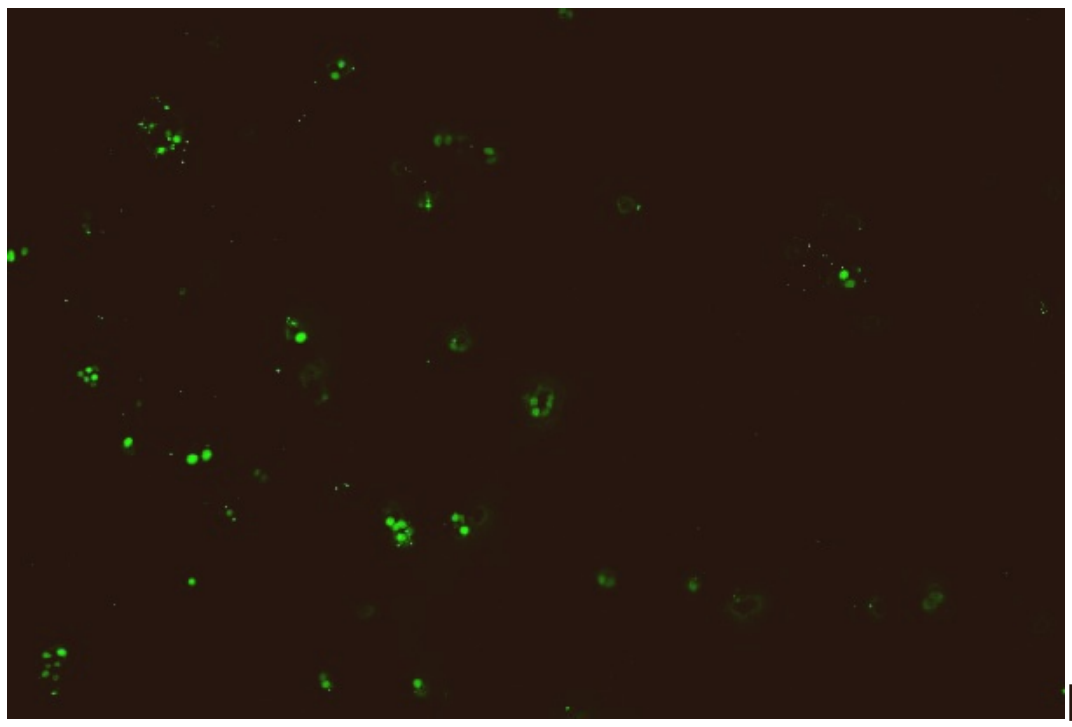

137 Figure 5I MafA (FITC), TAC

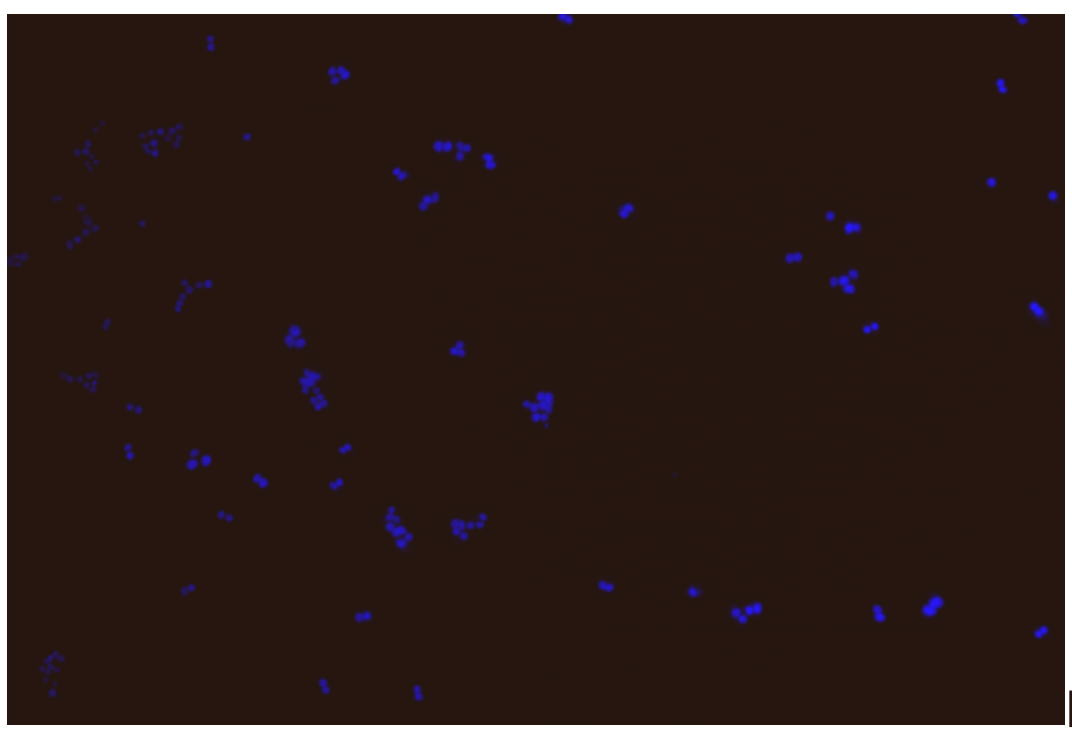

138 Figure 5I DAPI, TAC

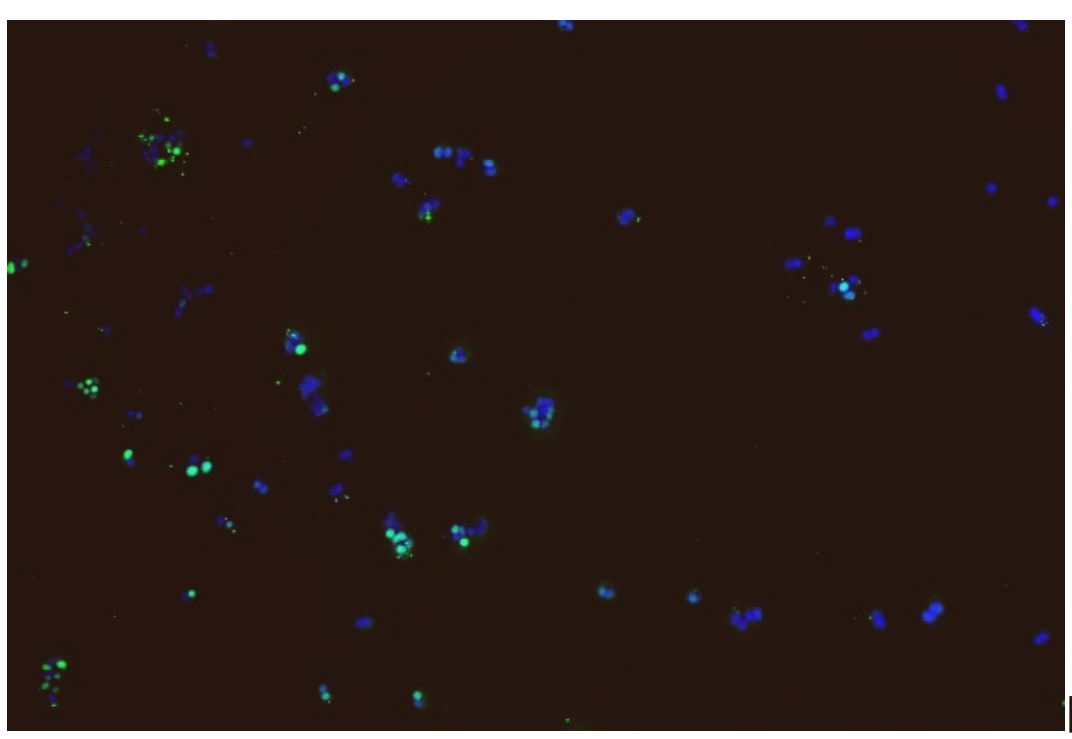

139 Figure 5I merge, TAC

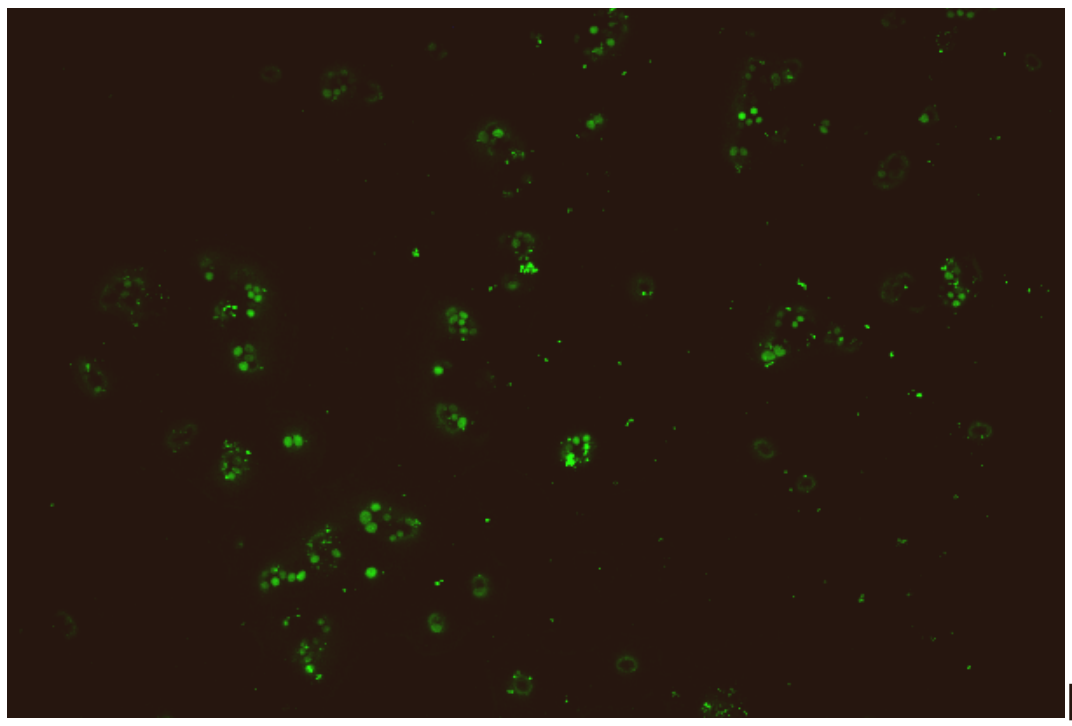
Figure 5I MafA (FITC), TAC+L-His

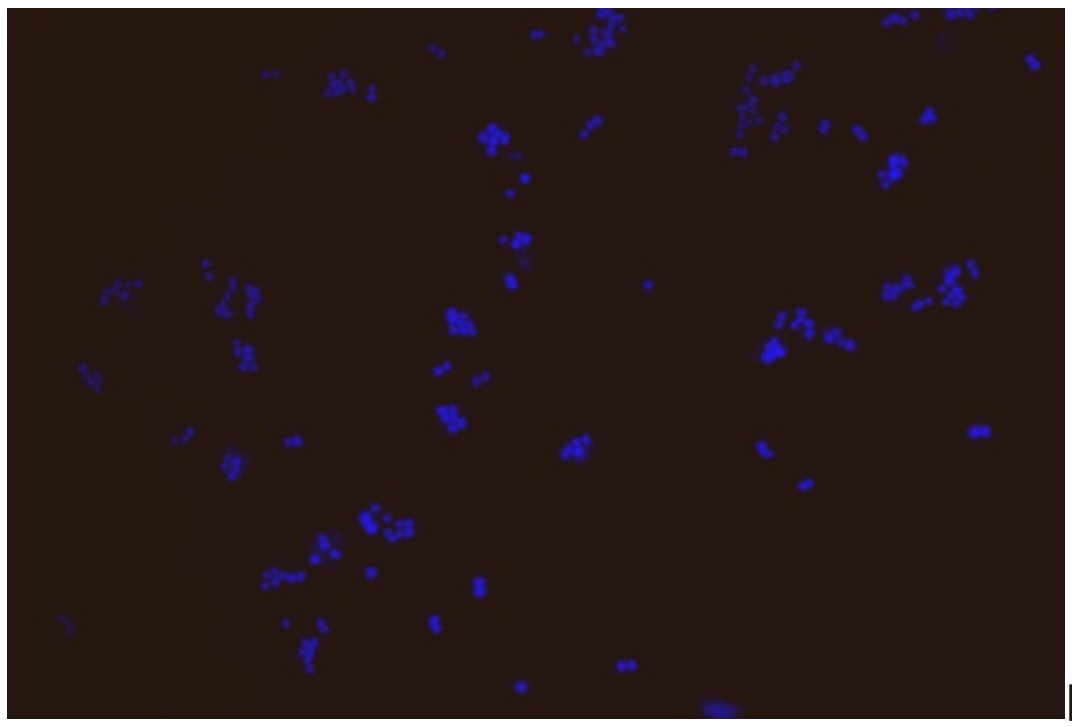
Figure 5I DAPI, TAC+L-His

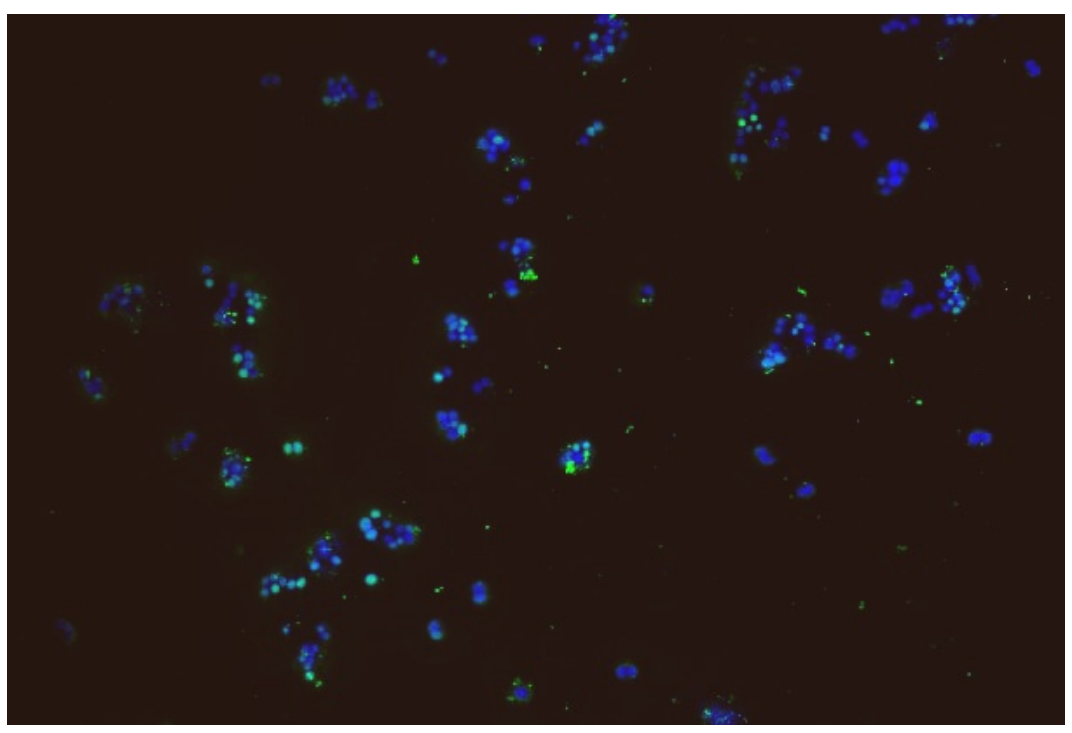
Figure 5I merge, TAC+L-His

FIGURE 5K

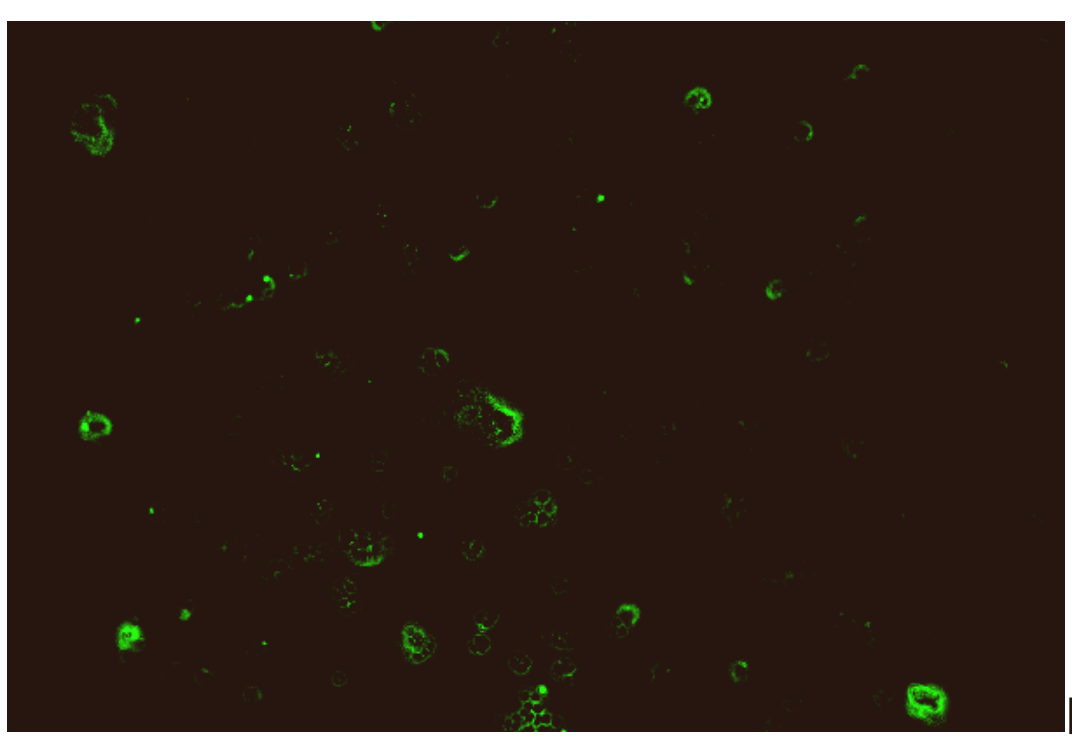
Figure 5K Insulin (FITC), control

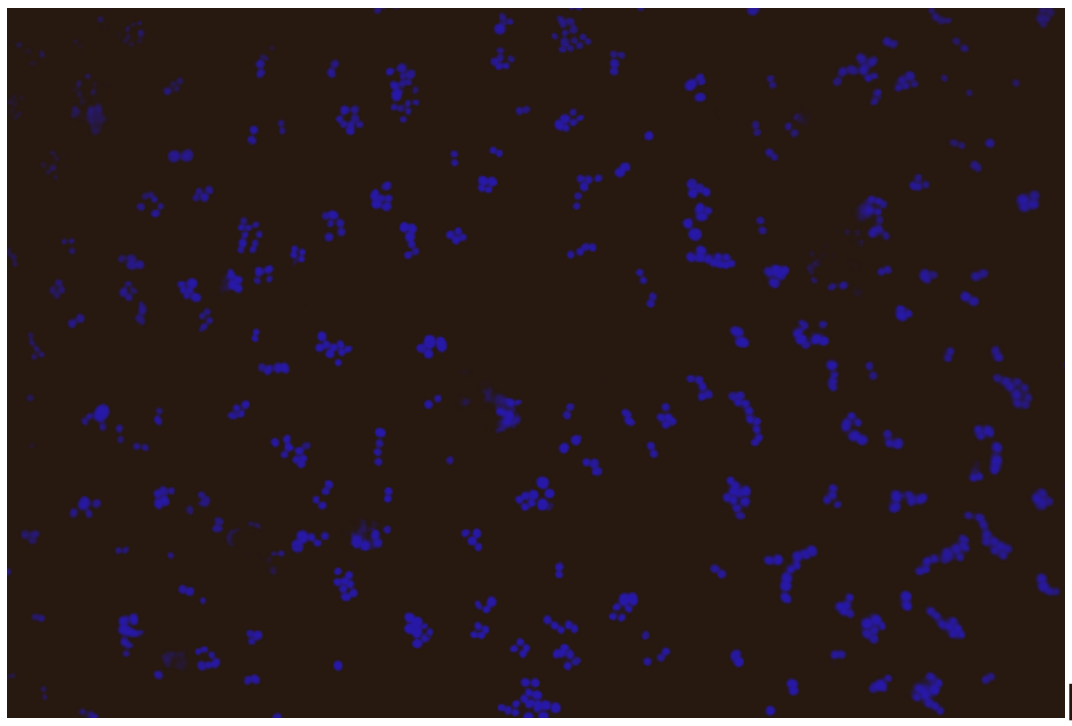
Figure 5K DAPI, control

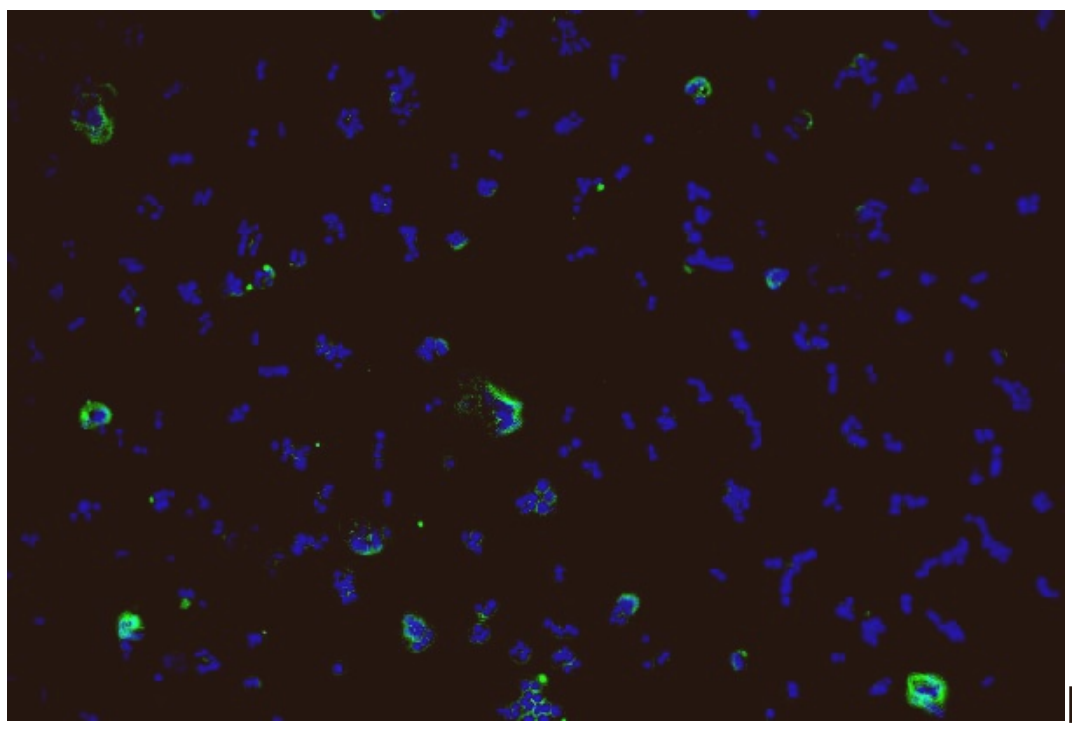
Figure 5K merge, control

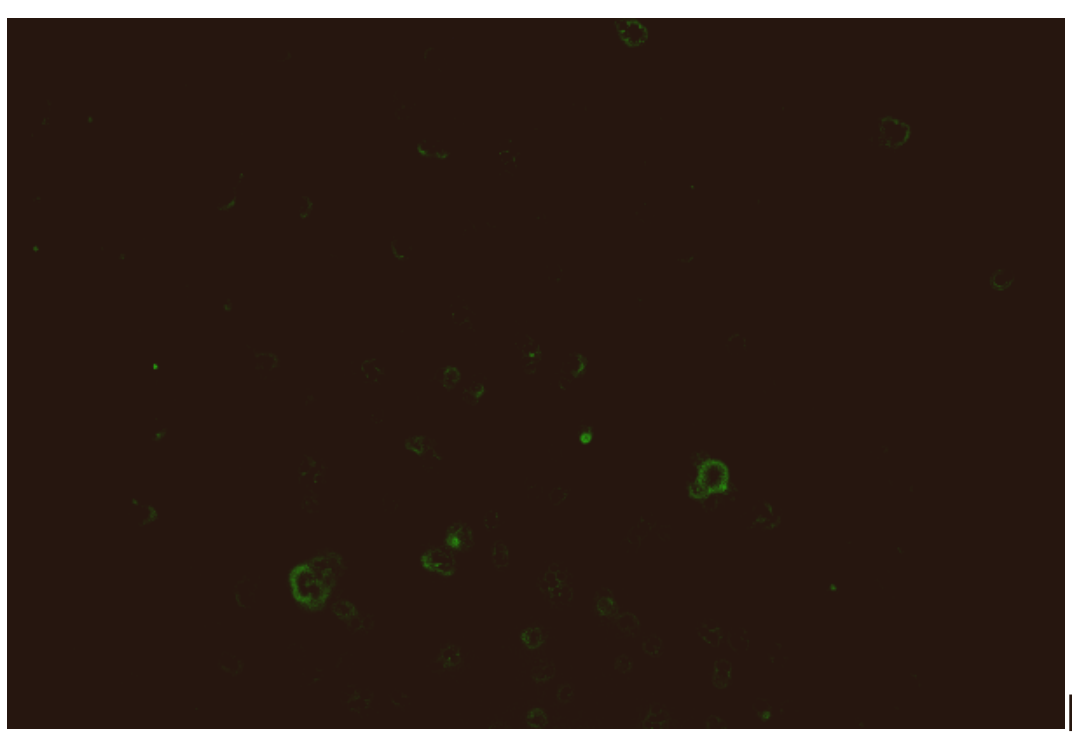
Figure 5K Insulin (FITC), TAC

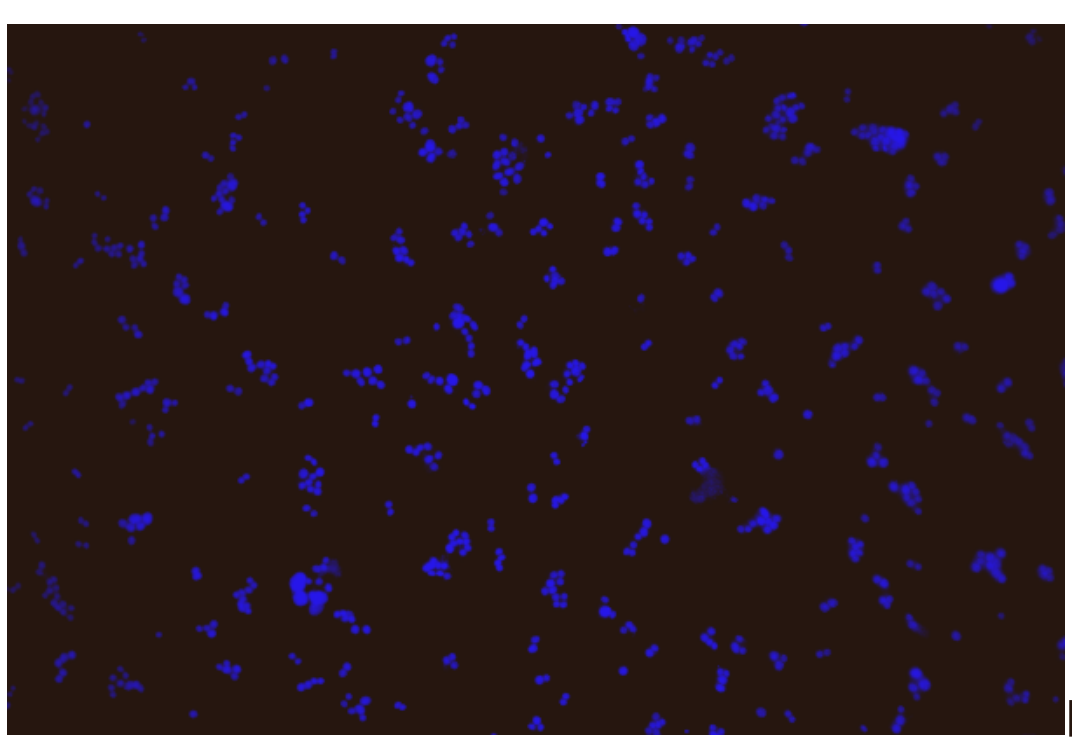
Figure 5K DAPI, TAC

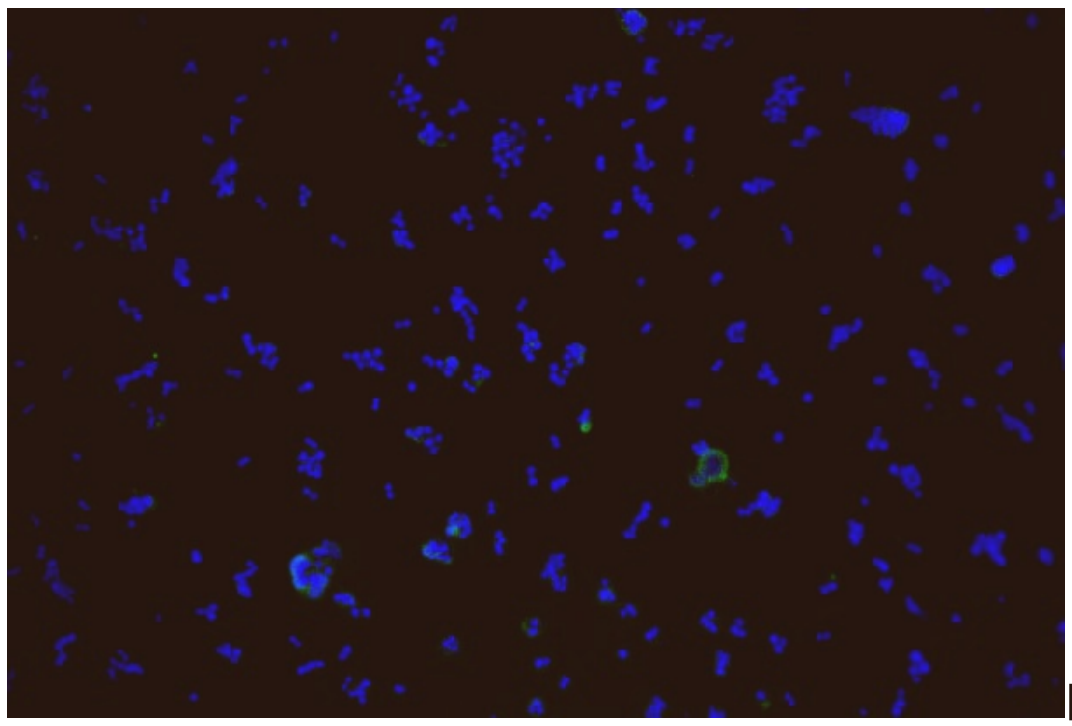
Figure 5K merge, TAC

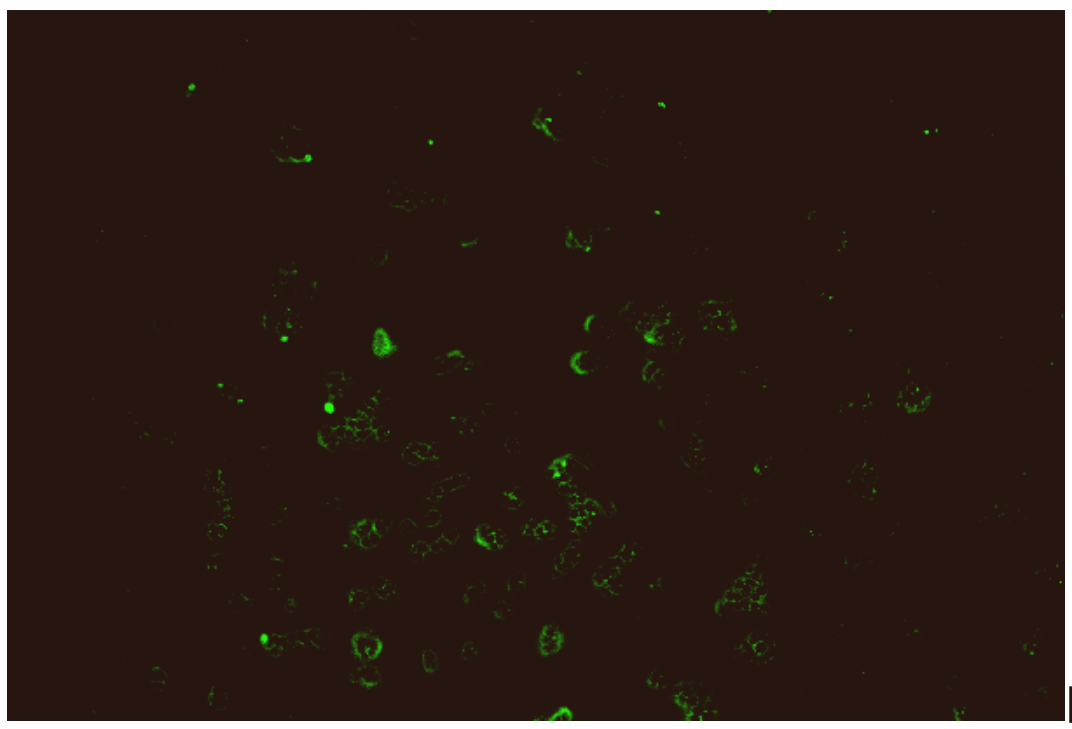
Figure 5K Insulin (FITC), TAC+L-His

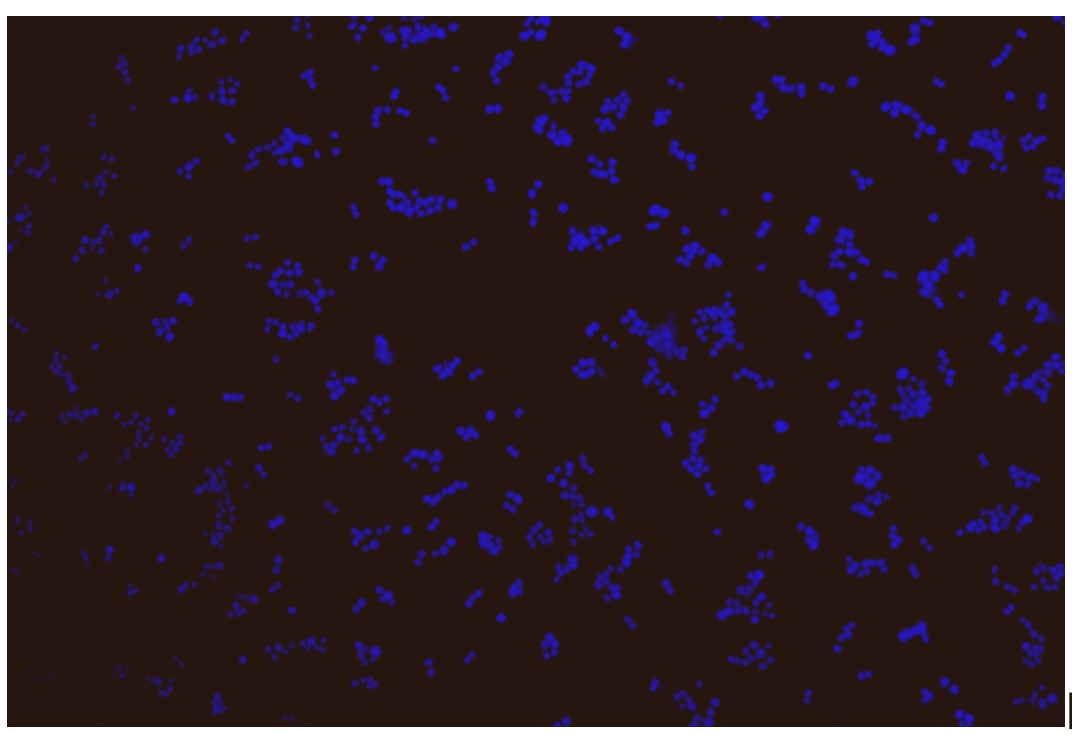
Figure 5K DAPI, TAC+L-His

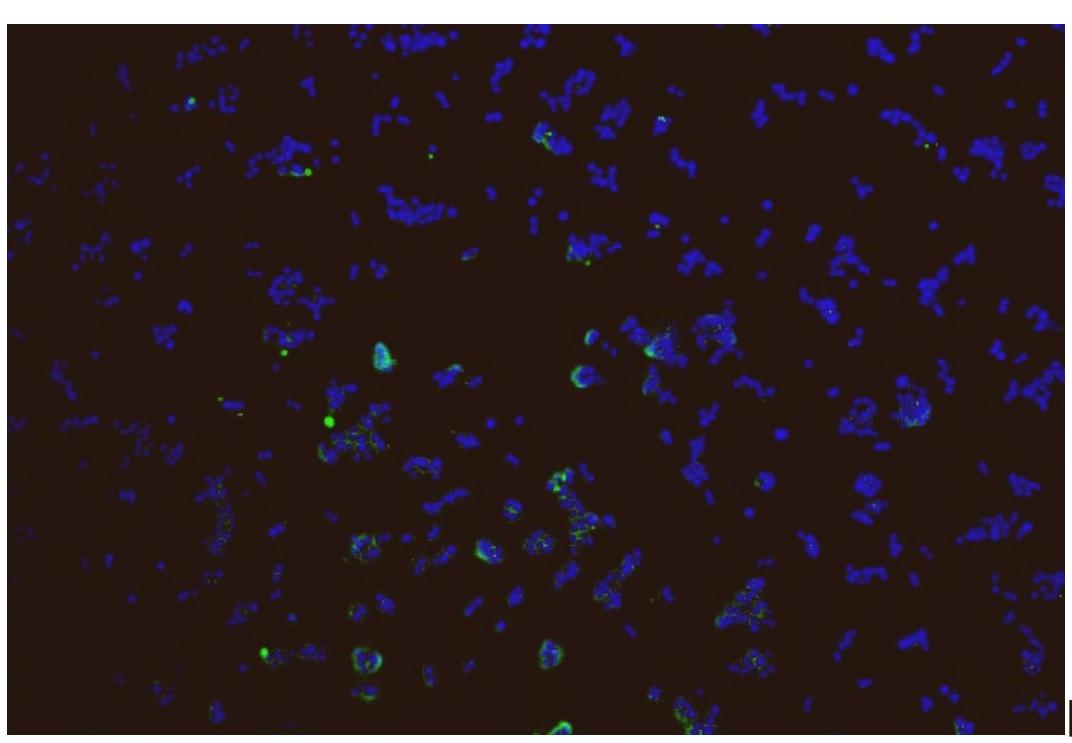
Figure 5K merge, TAC+L-His

FIGURE 6J

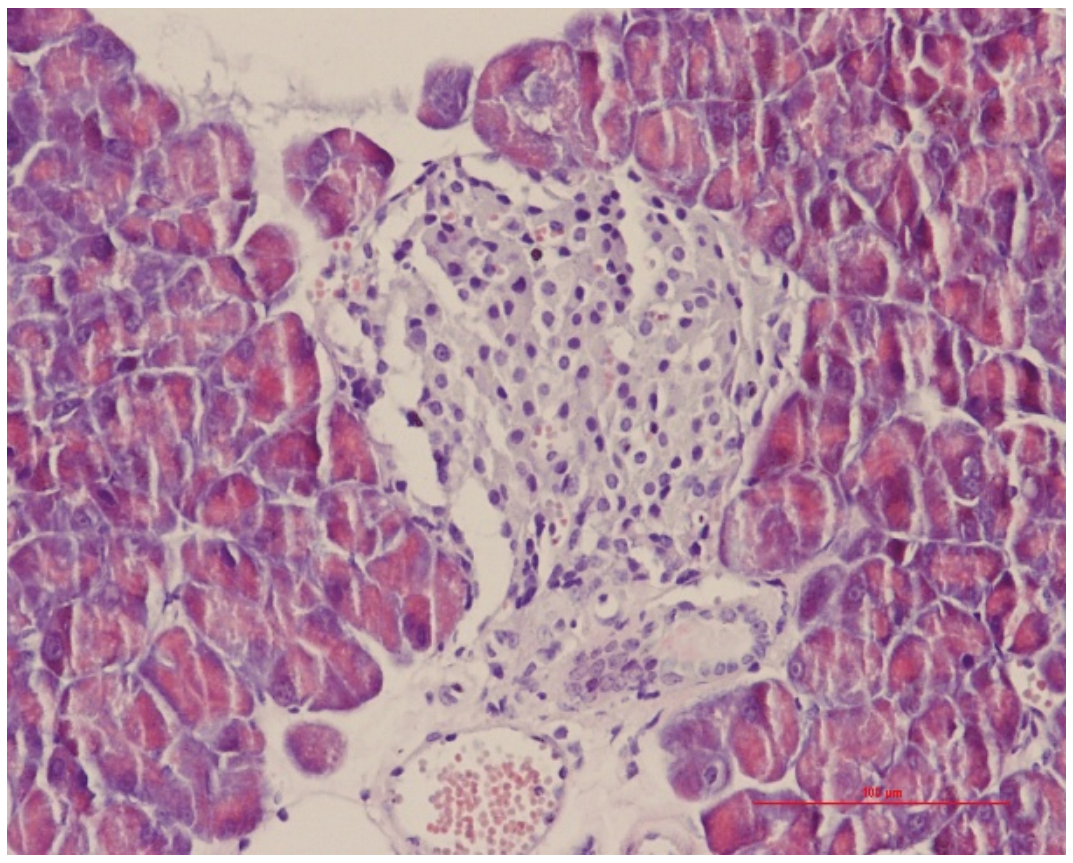

control

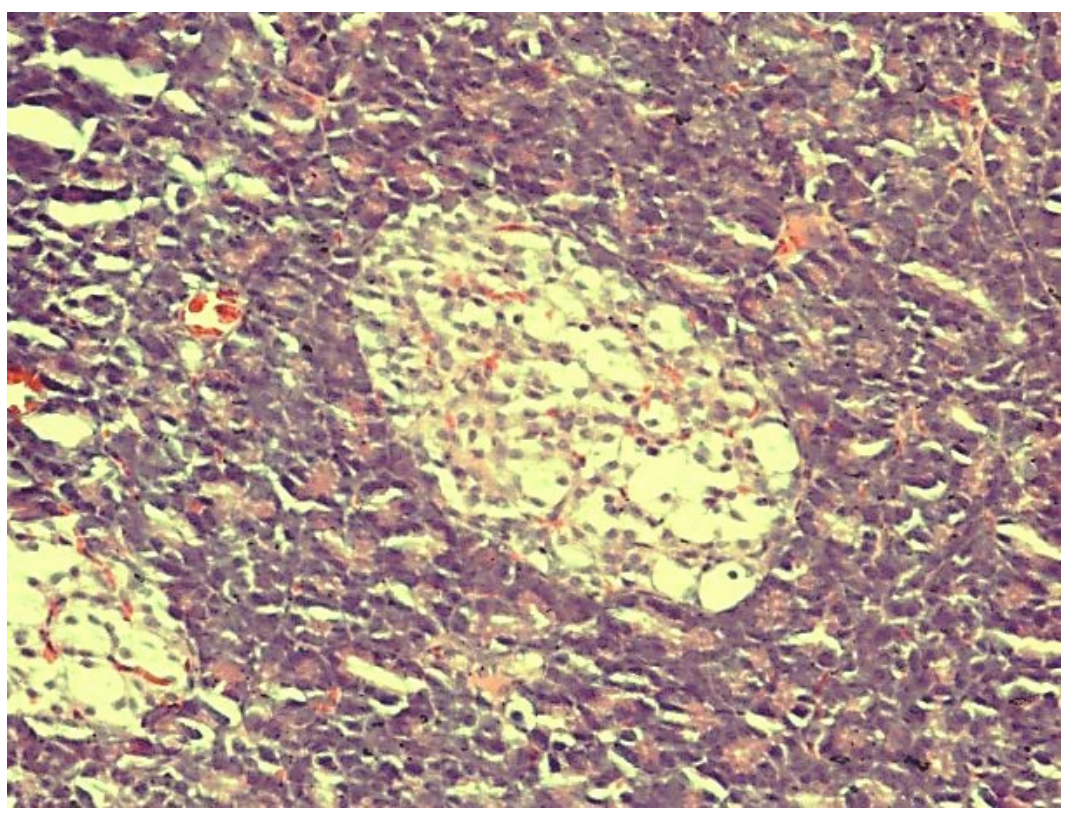

TAC

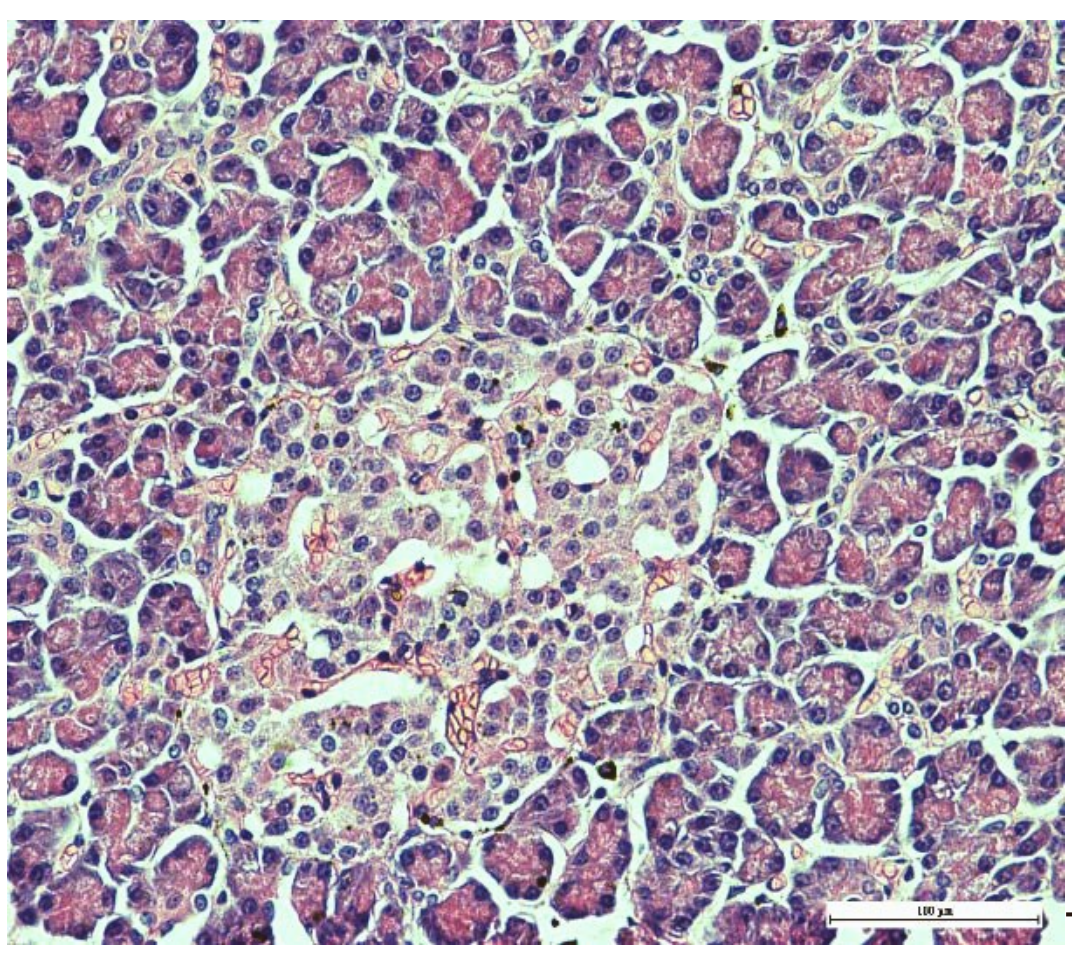

TAC+L-His

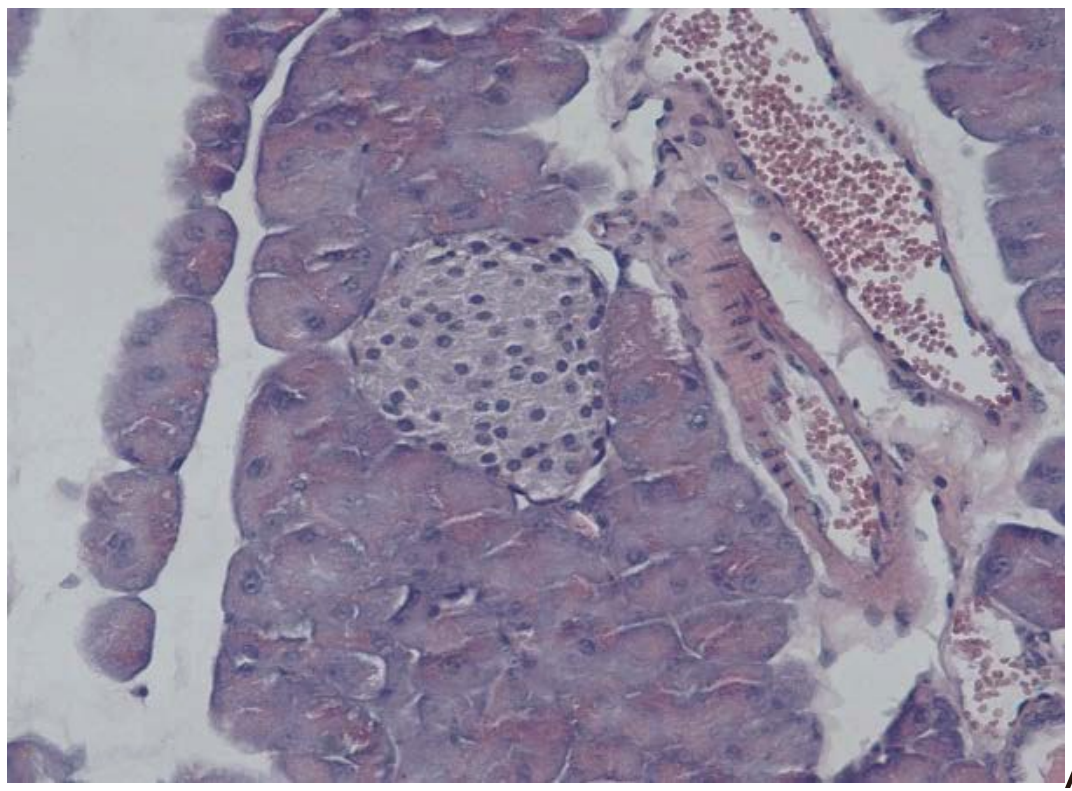
ABX

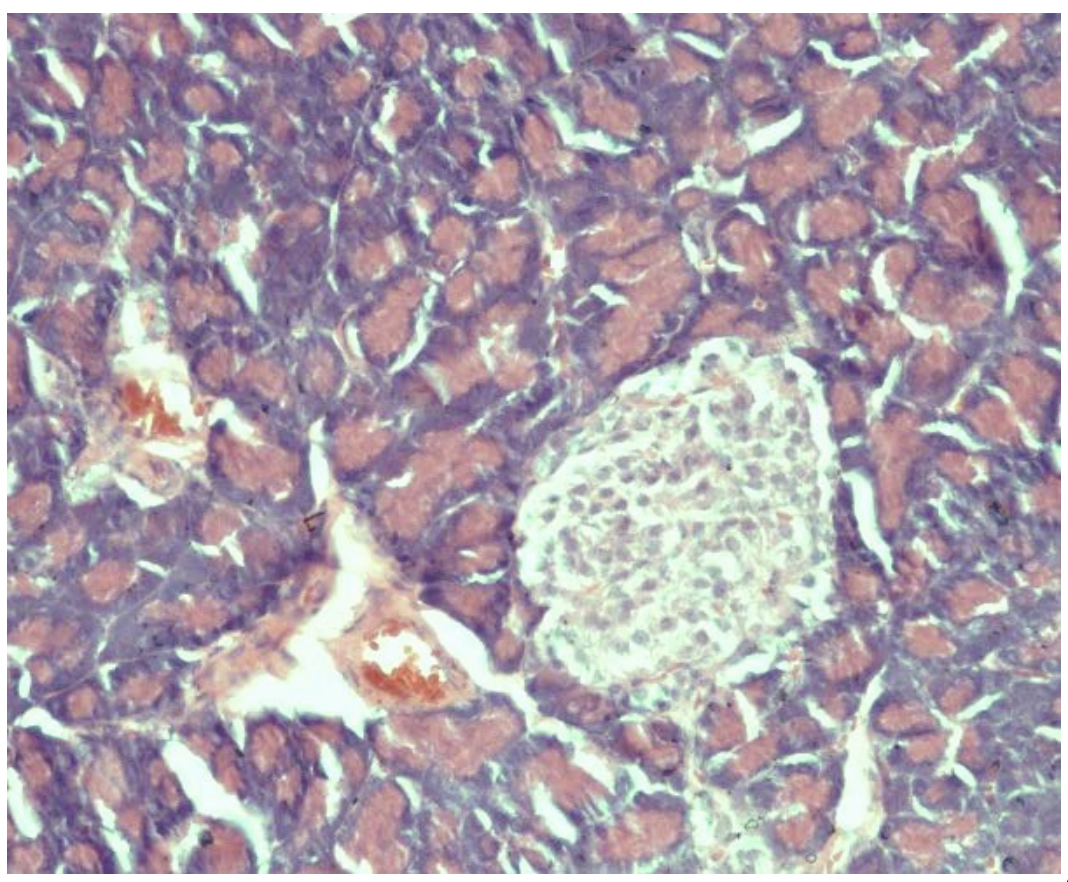
ABX+TAC

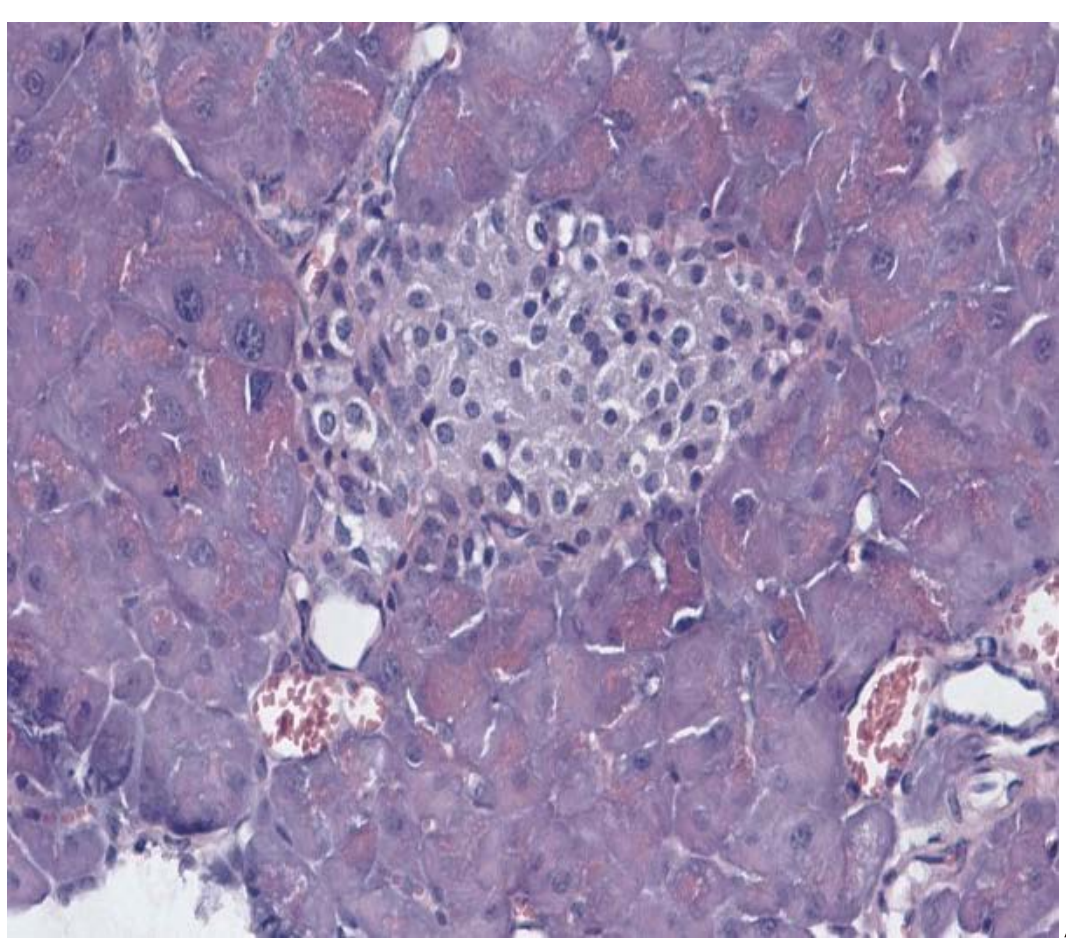
ABX+TAC+L-His

FIGURE 6K

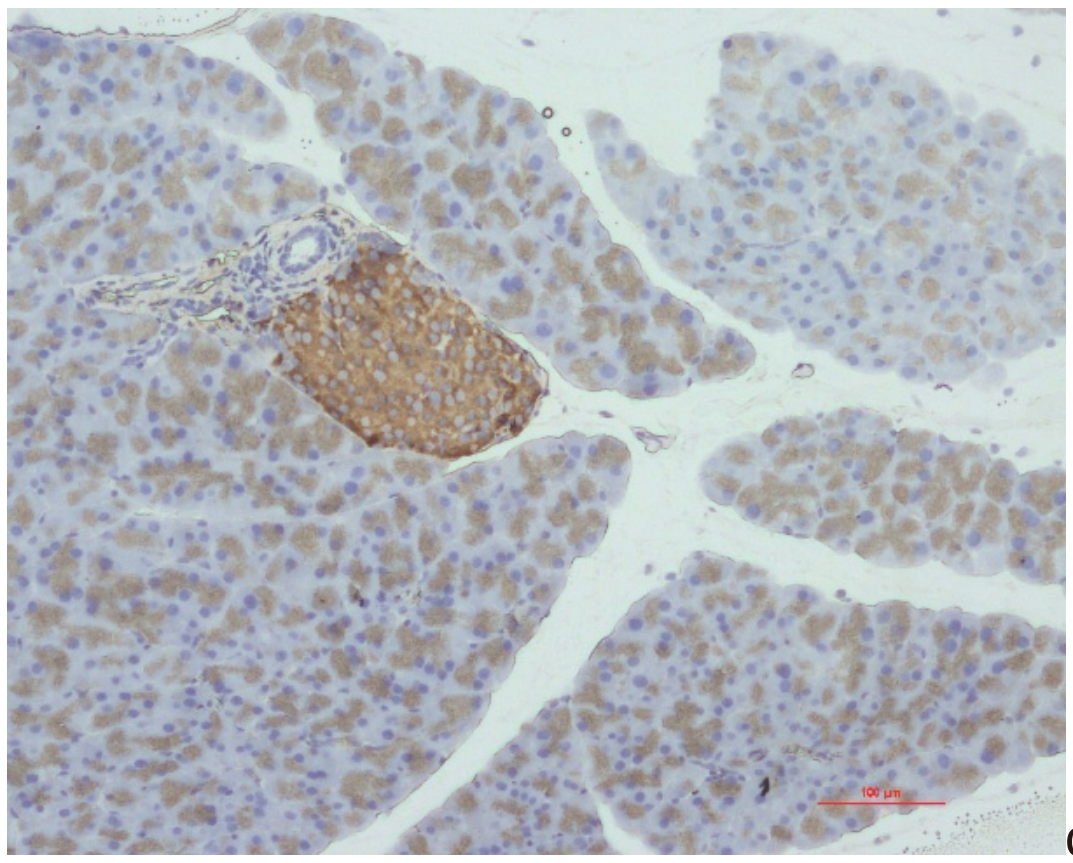

control

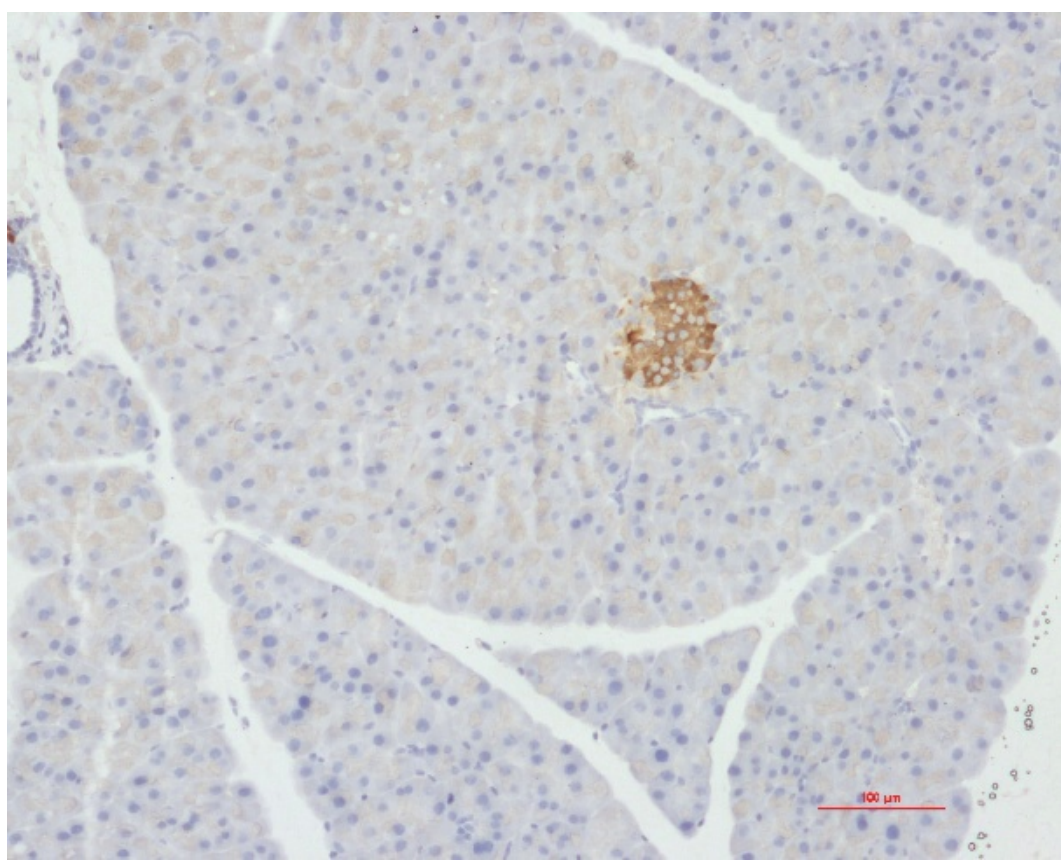

TAC

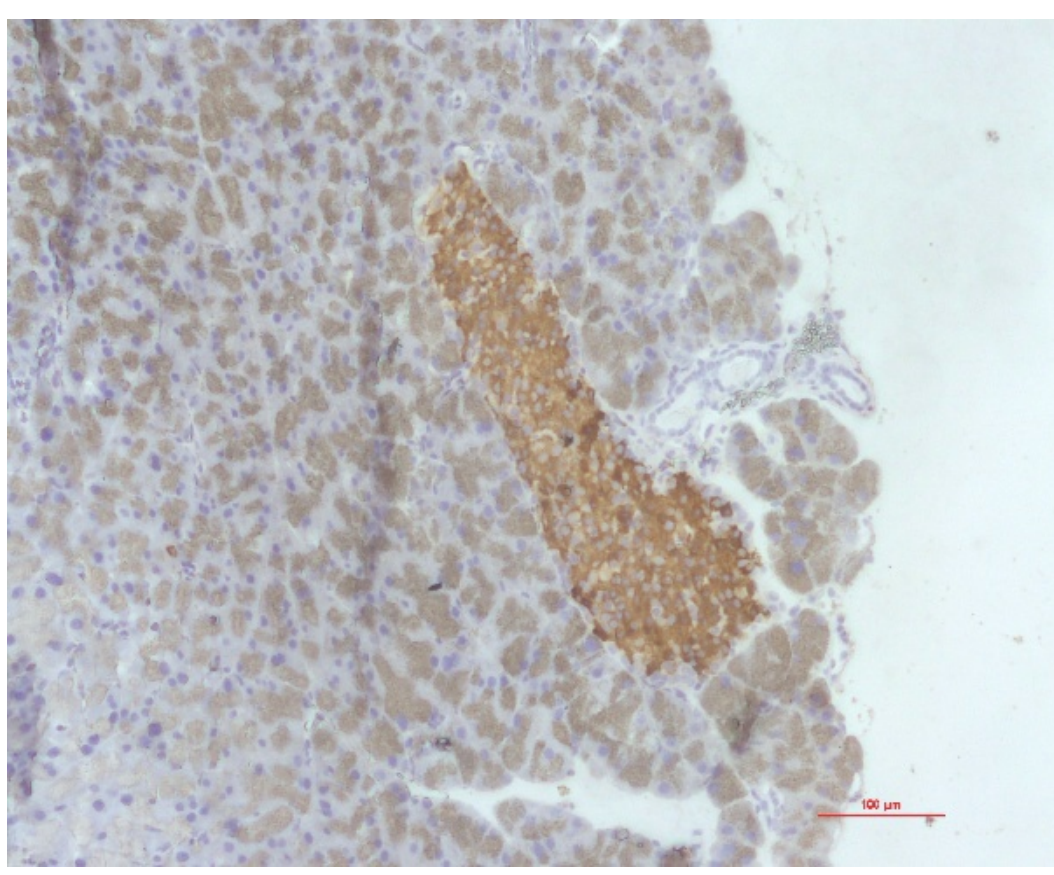

TAC+L-His

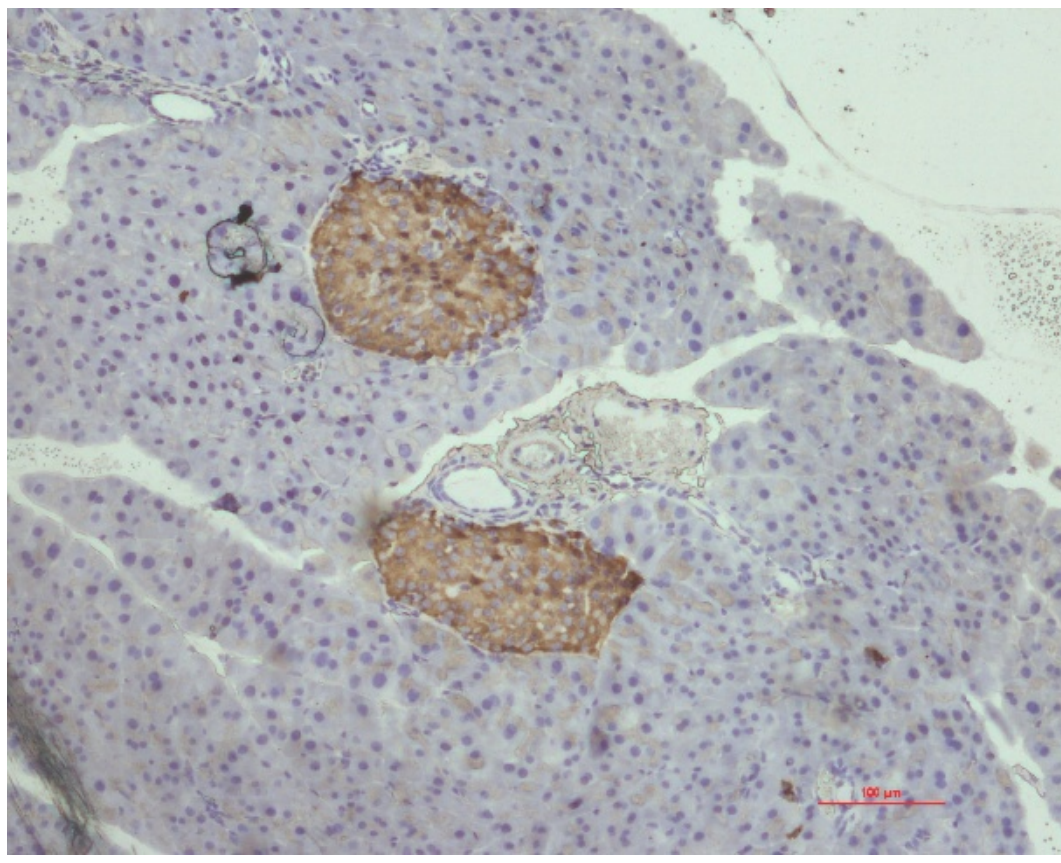
ABX

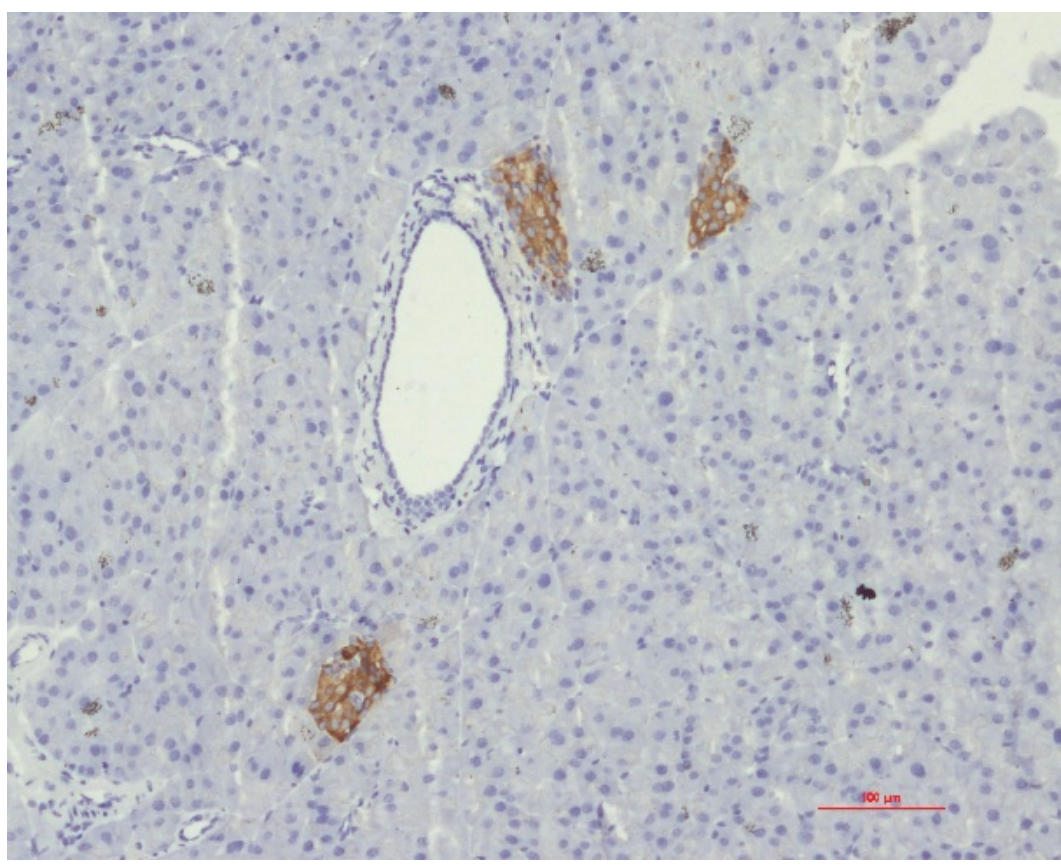
ABX+TAC

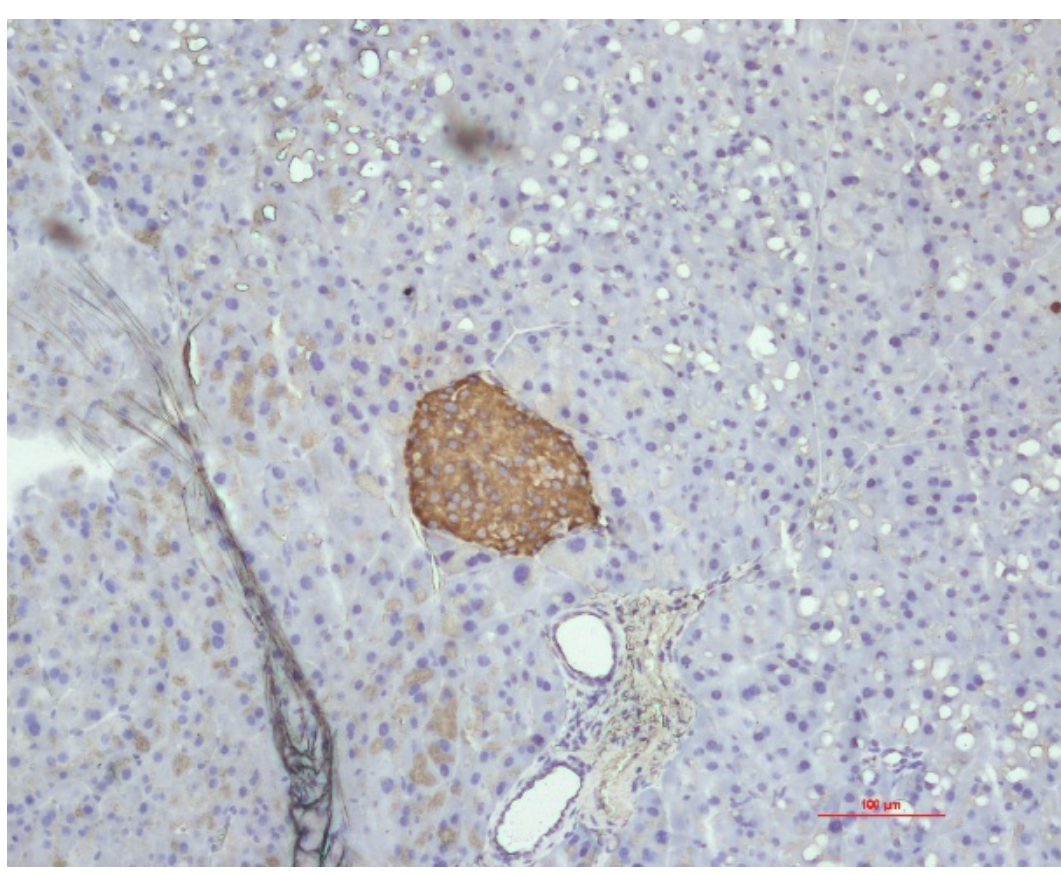
ABX+TAC+L-His

FIGURE 6L

CONTROL

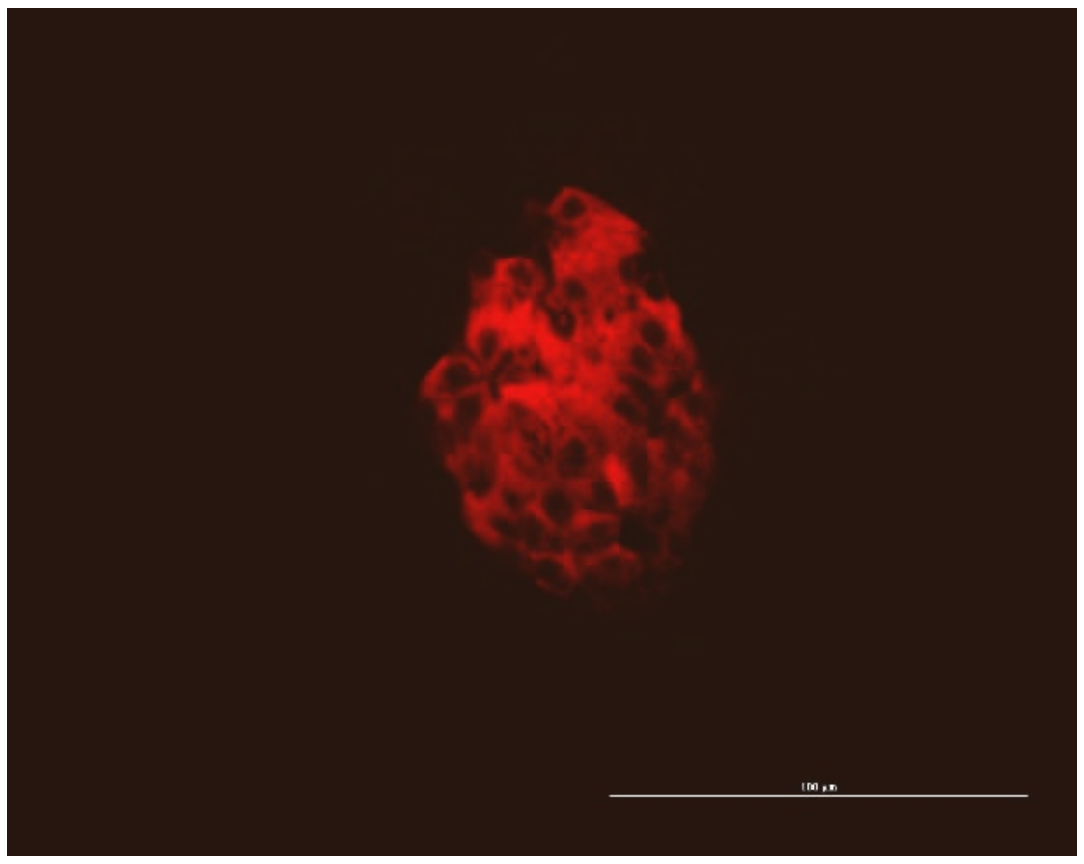

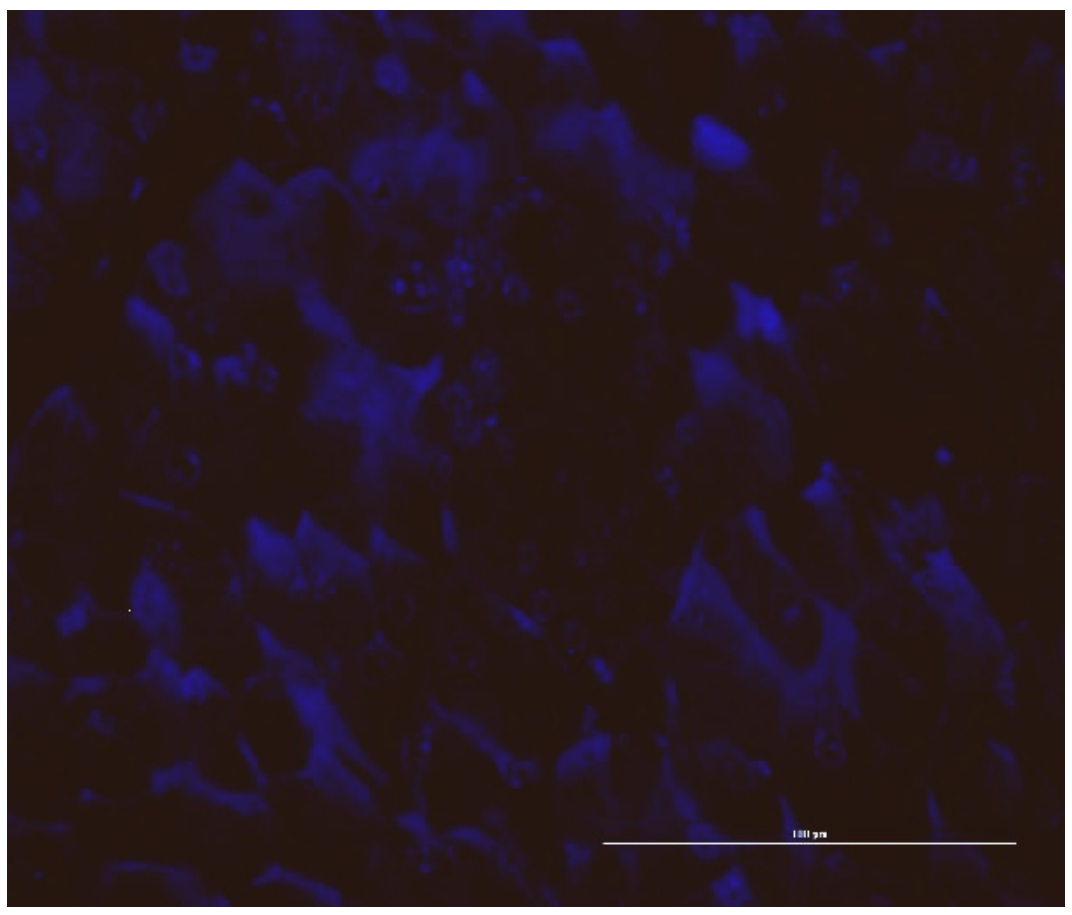

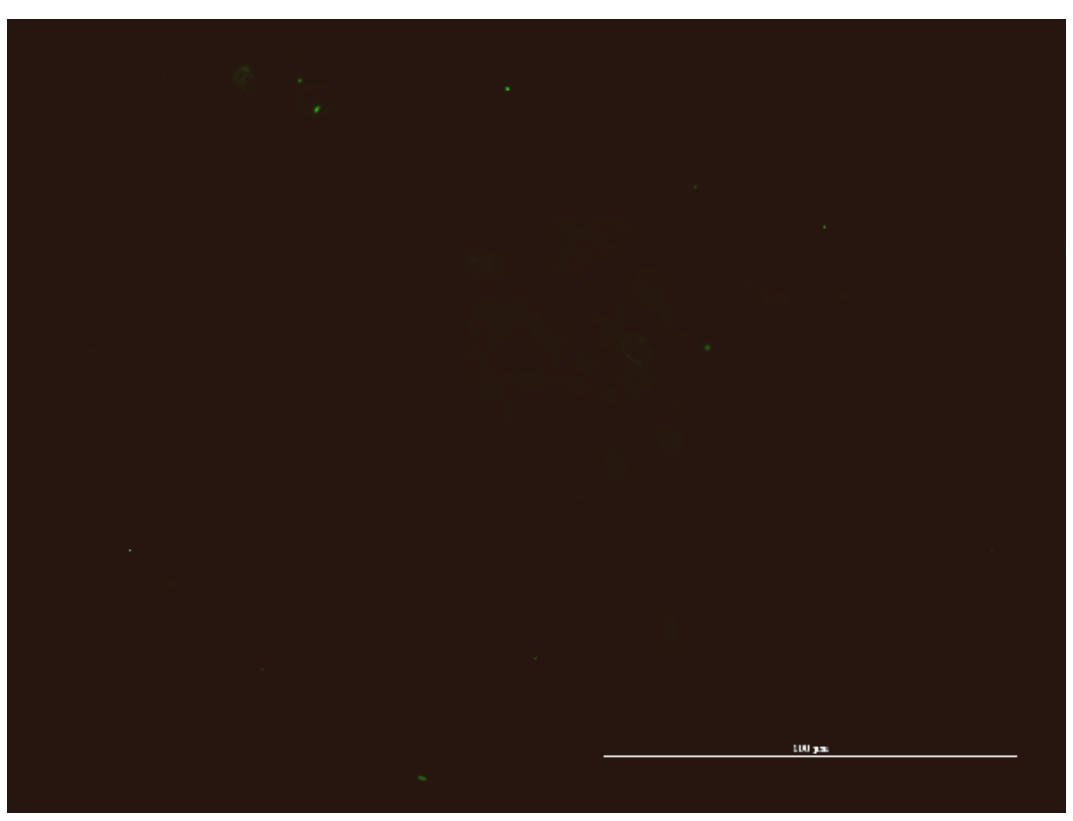

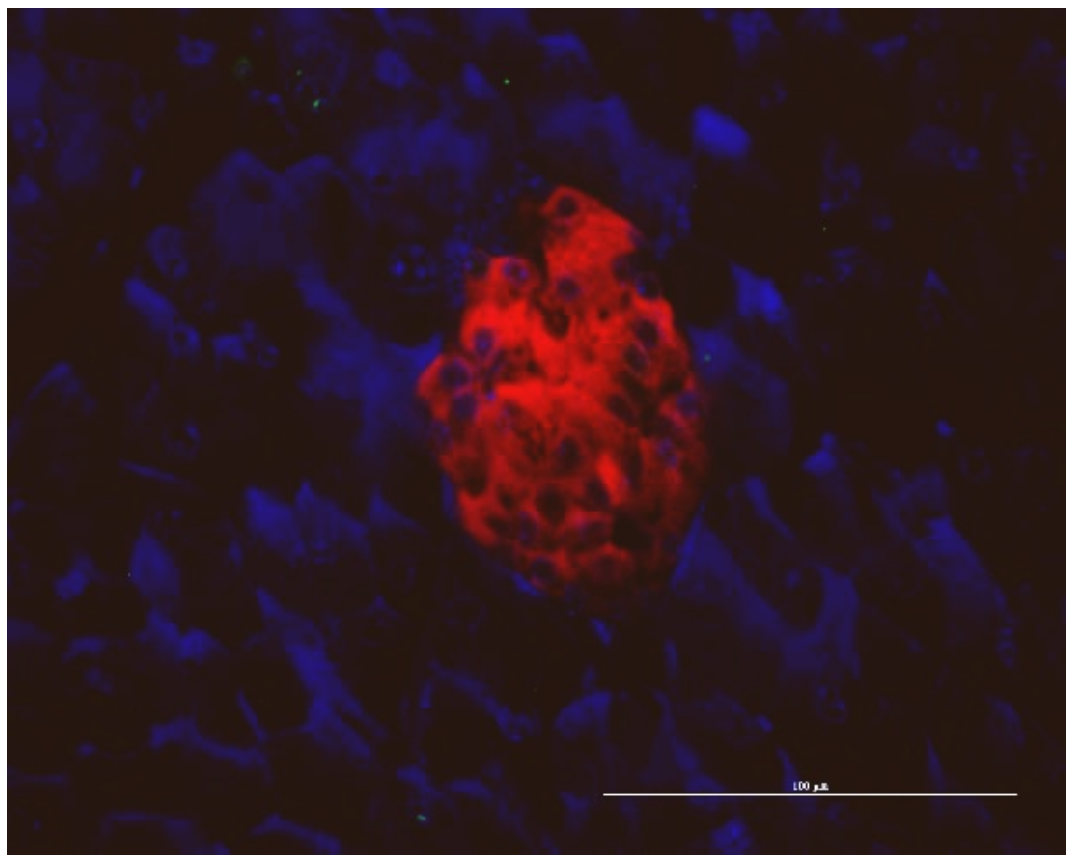

TAC

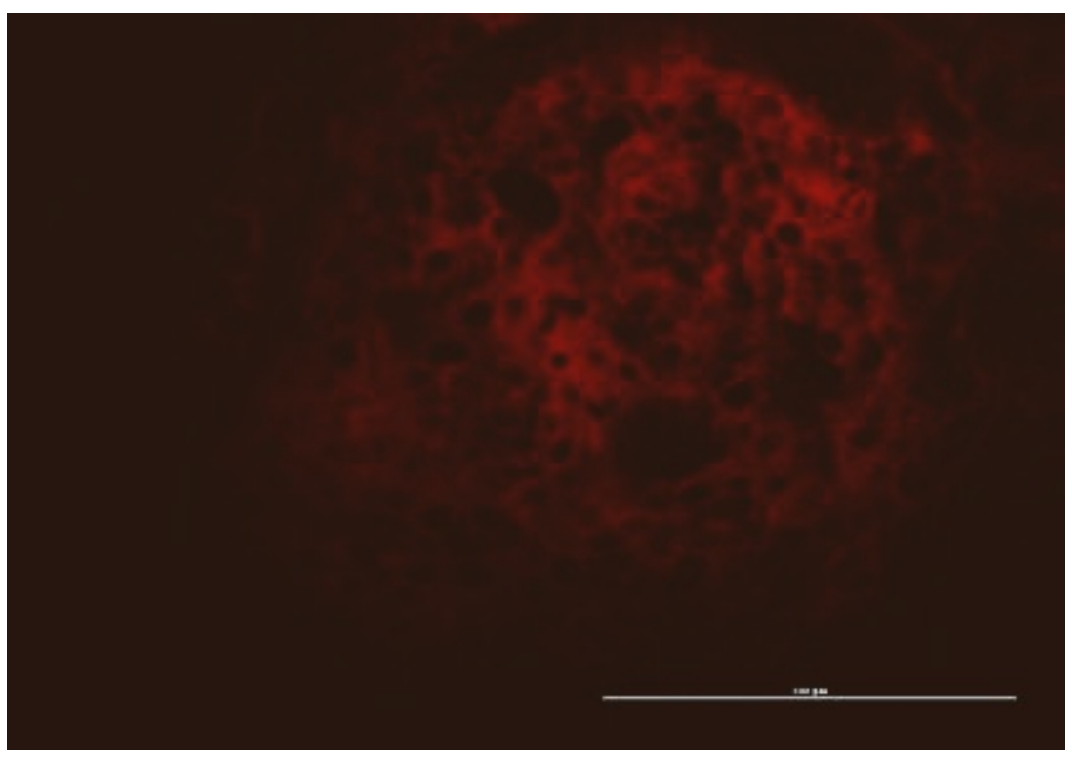

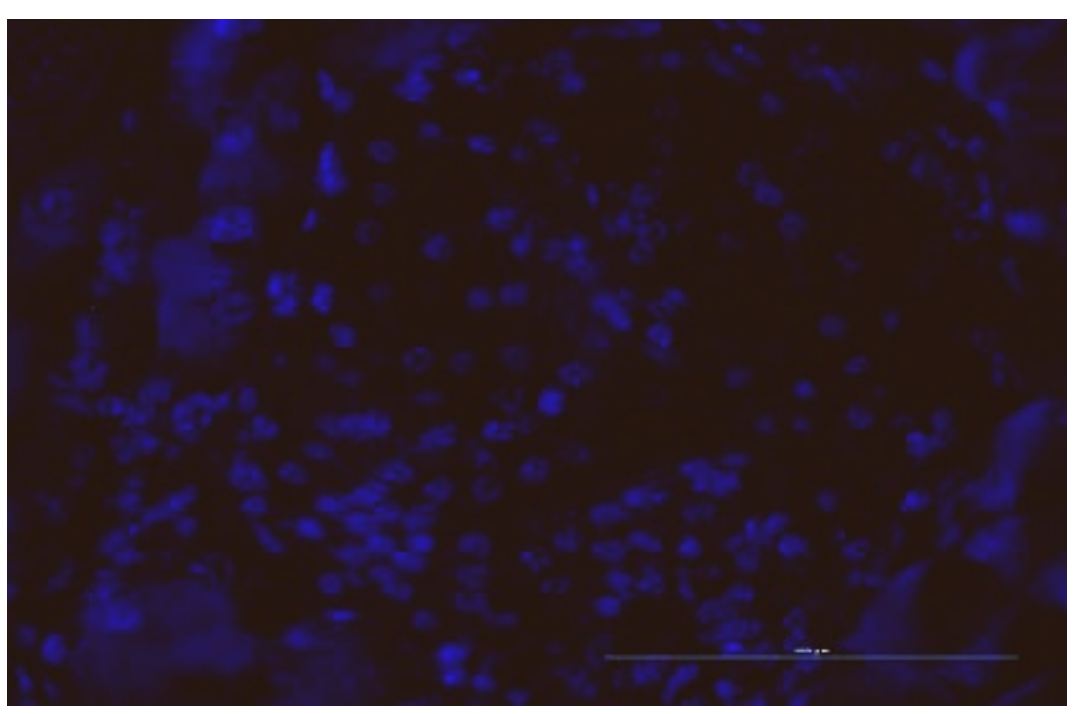

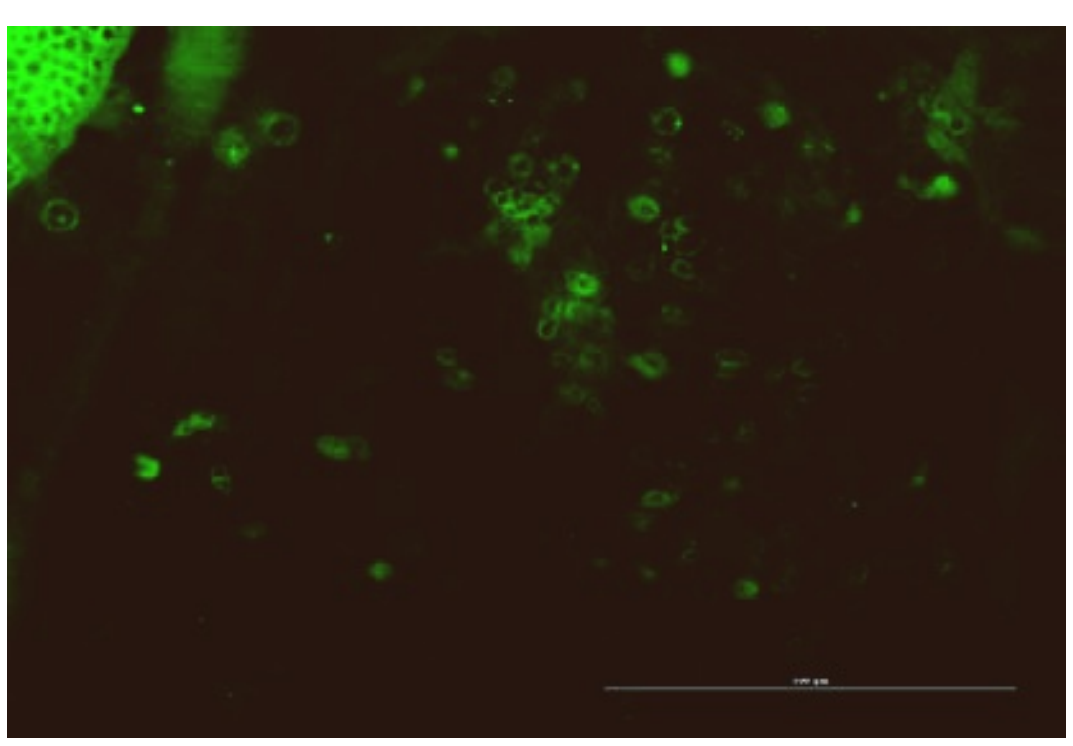

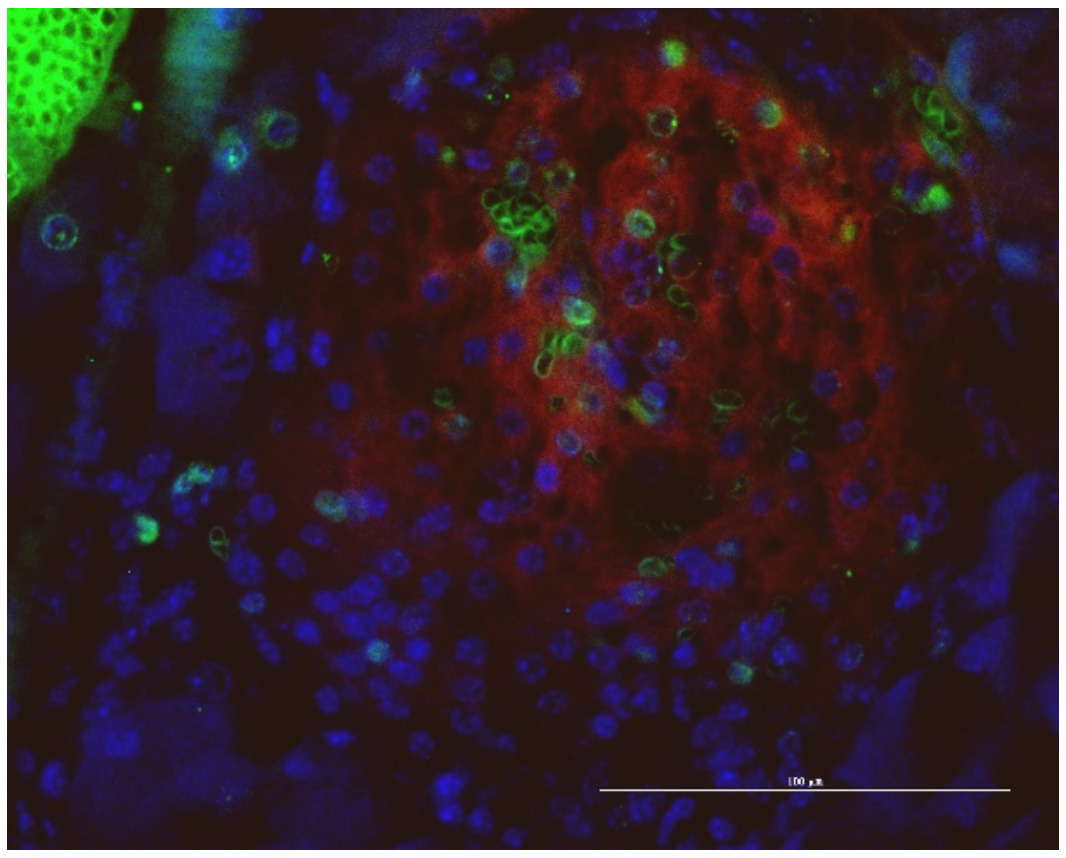

TAC+L-HIS

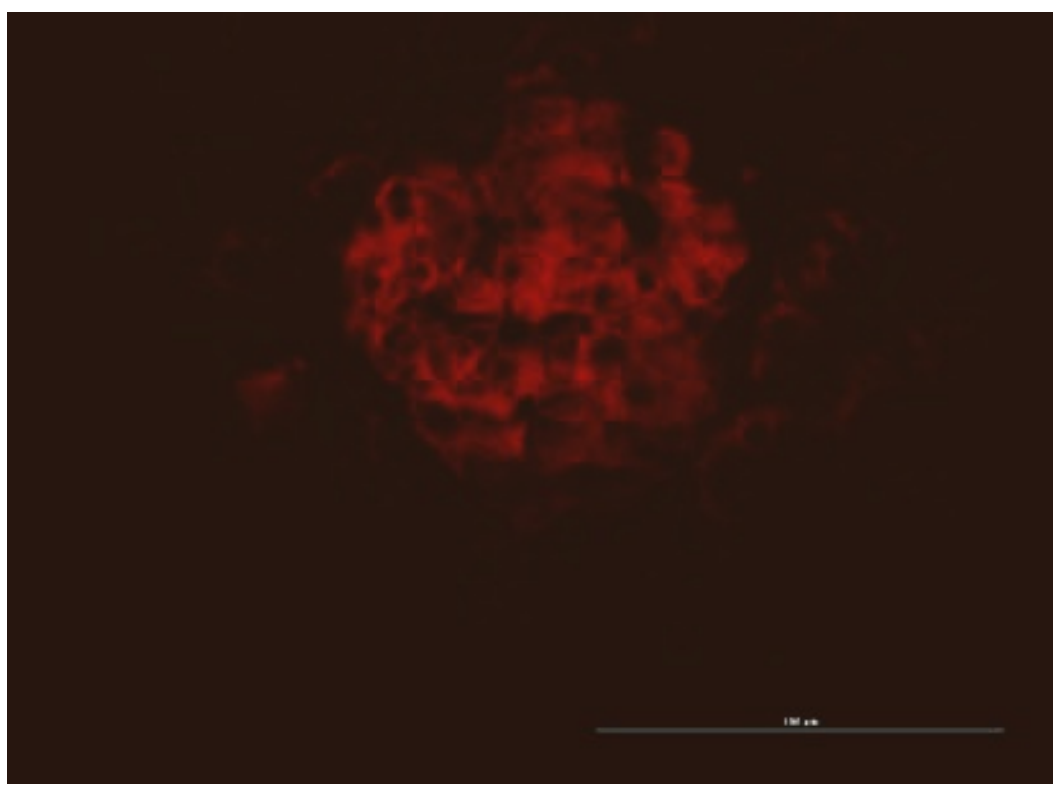

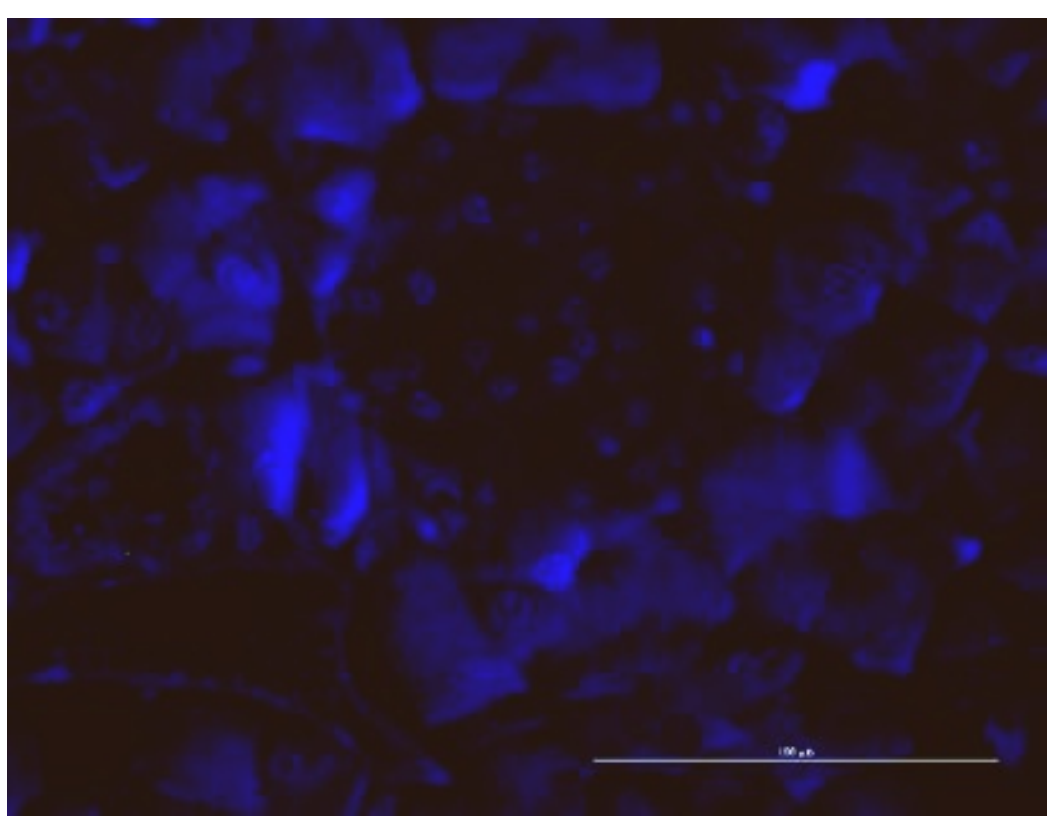

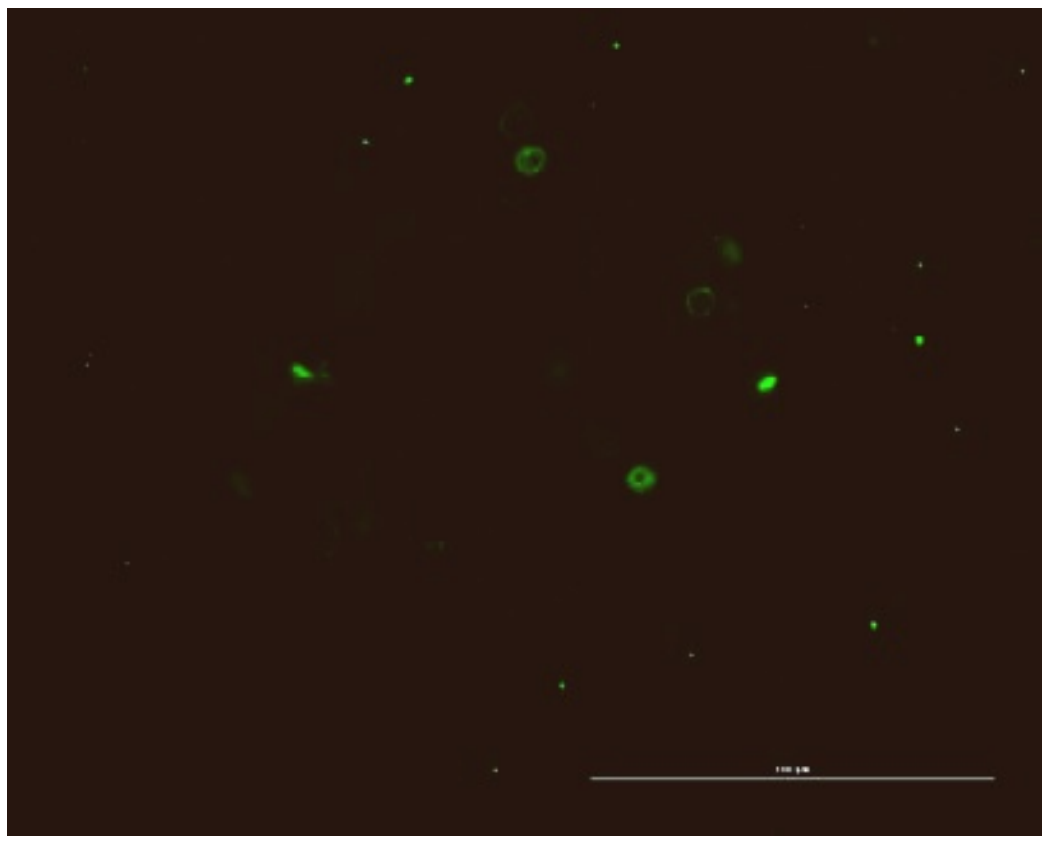

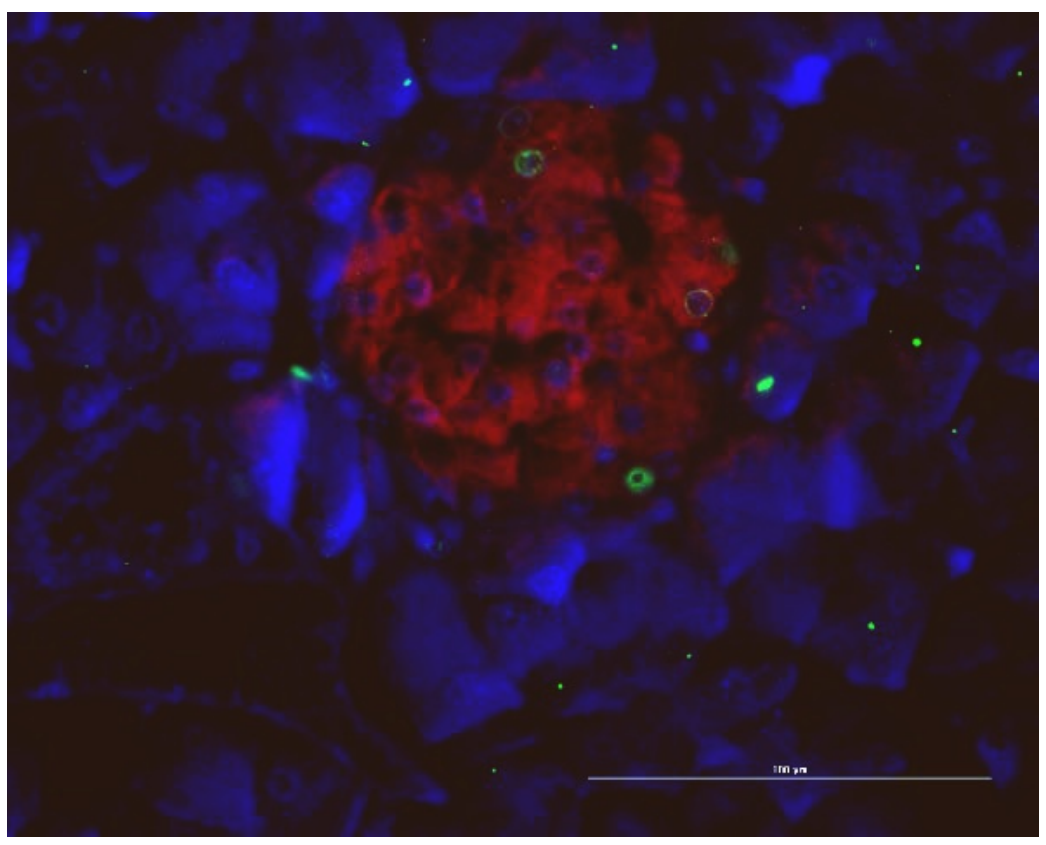

ABX

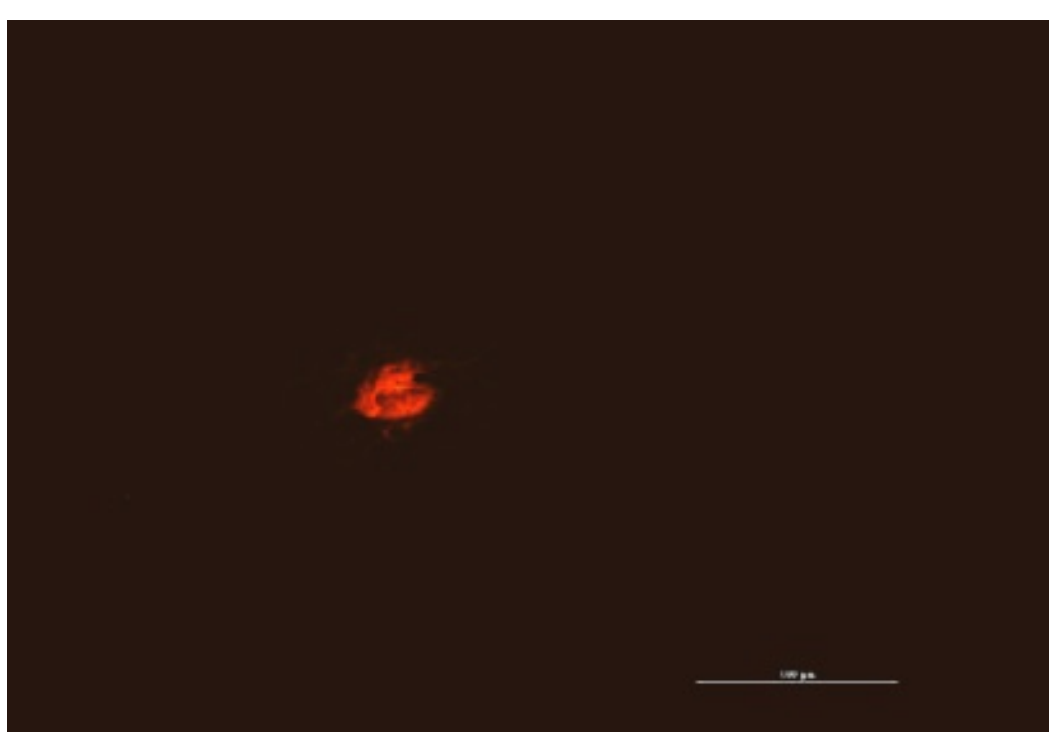

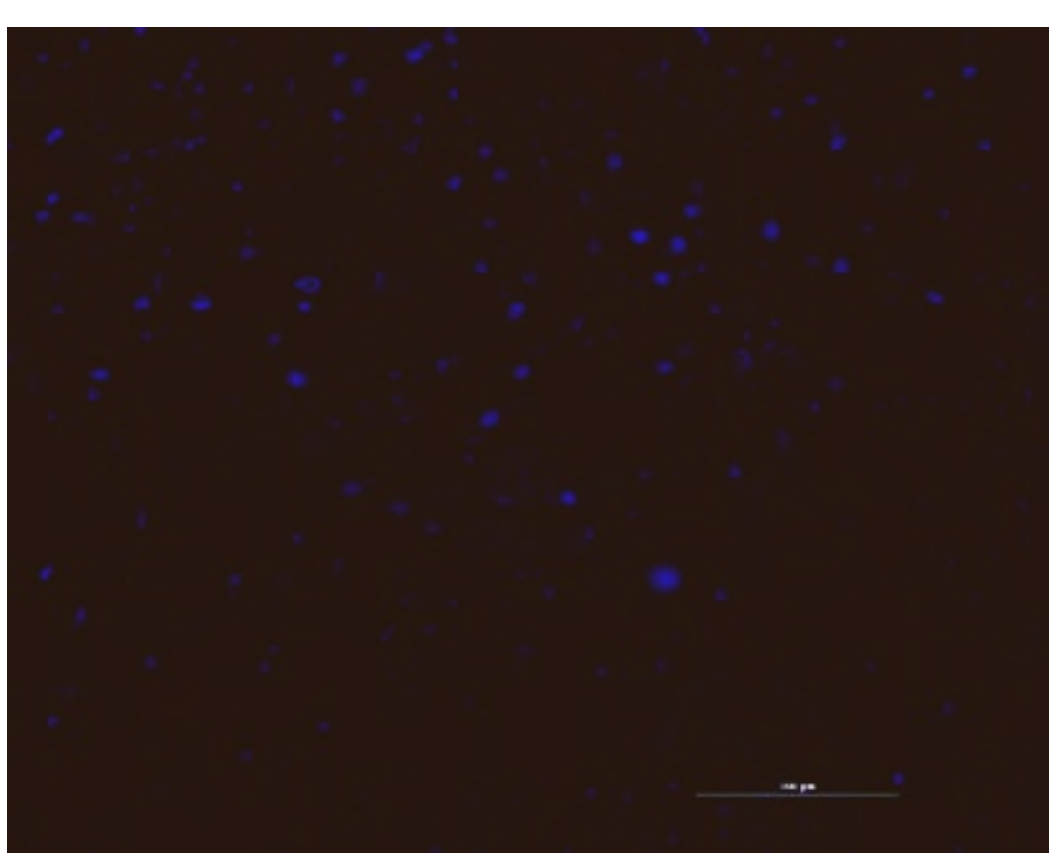

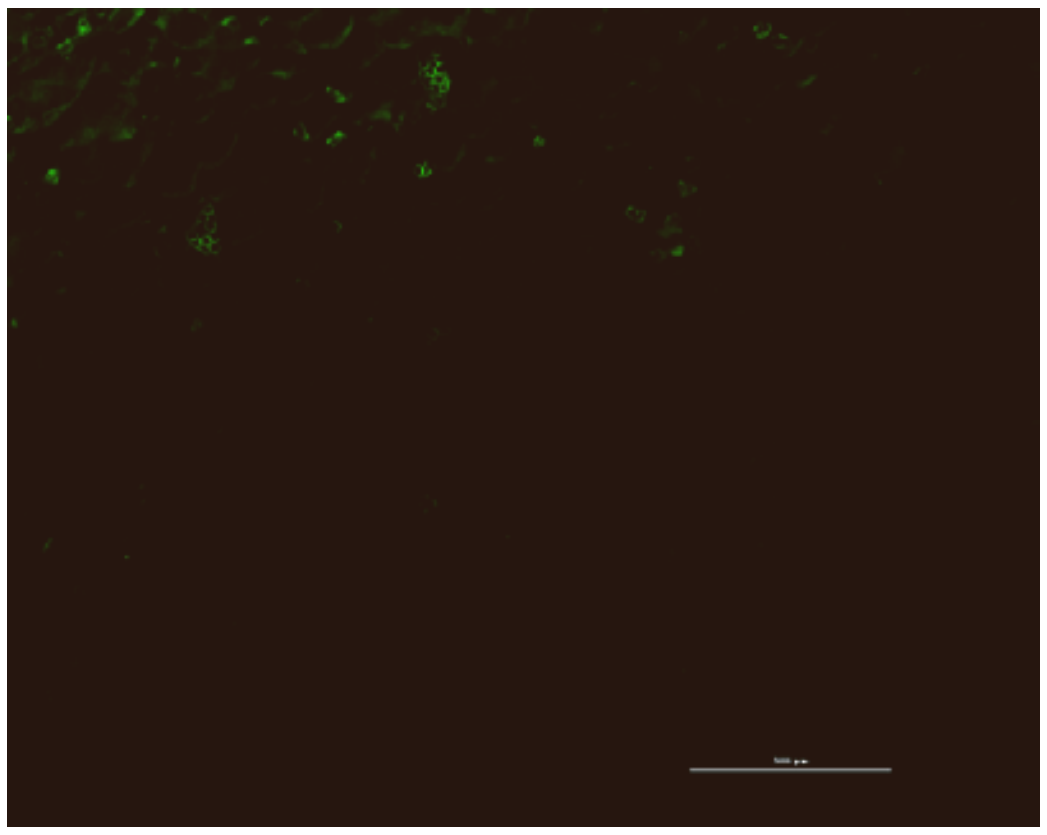

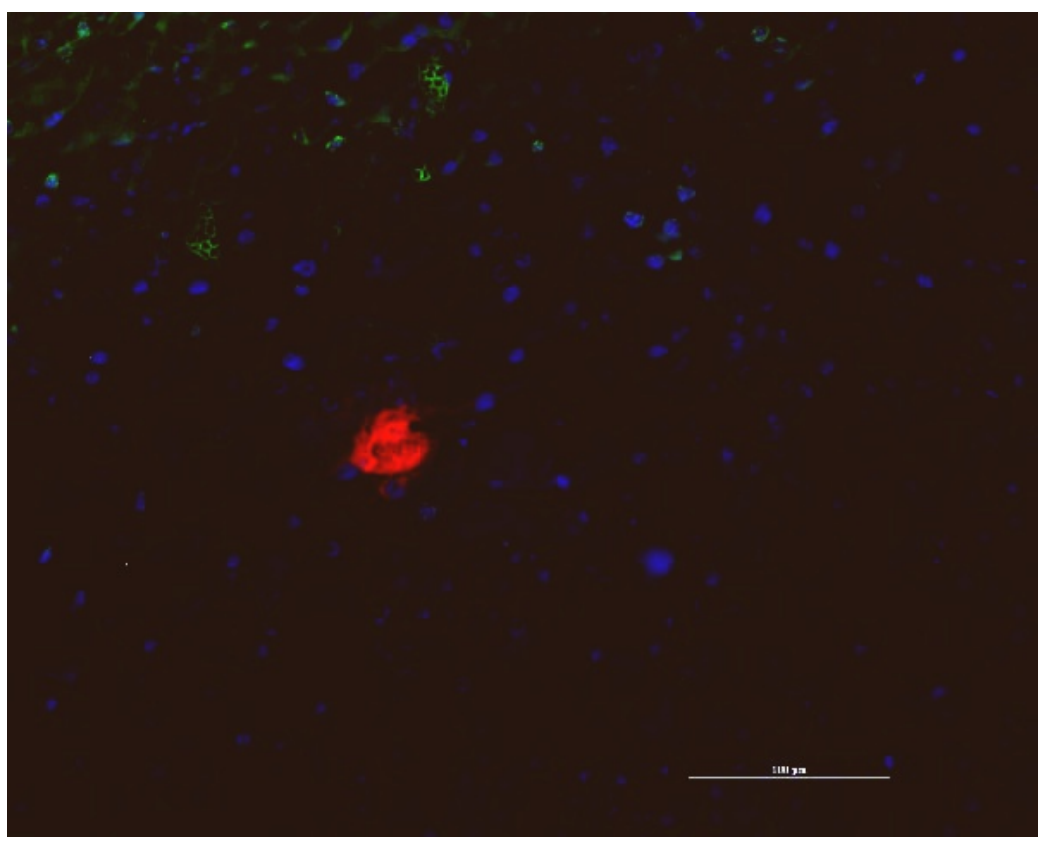

ABX+TAC

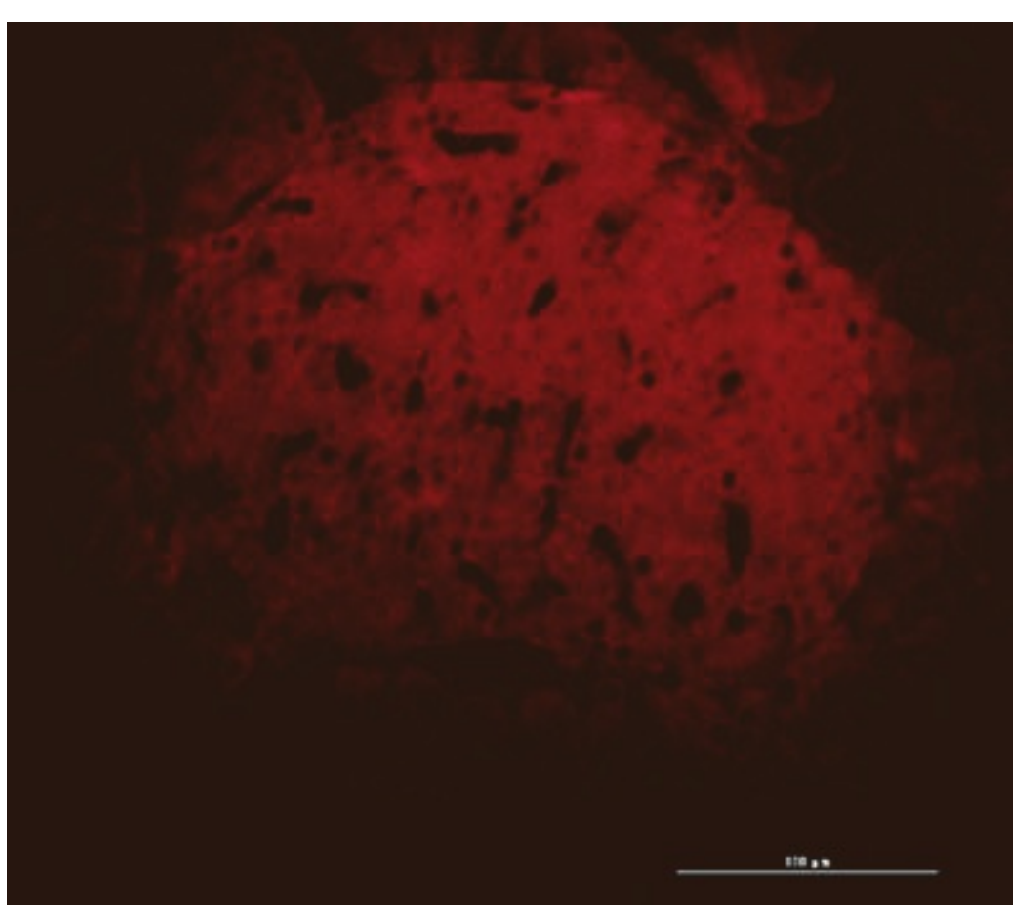

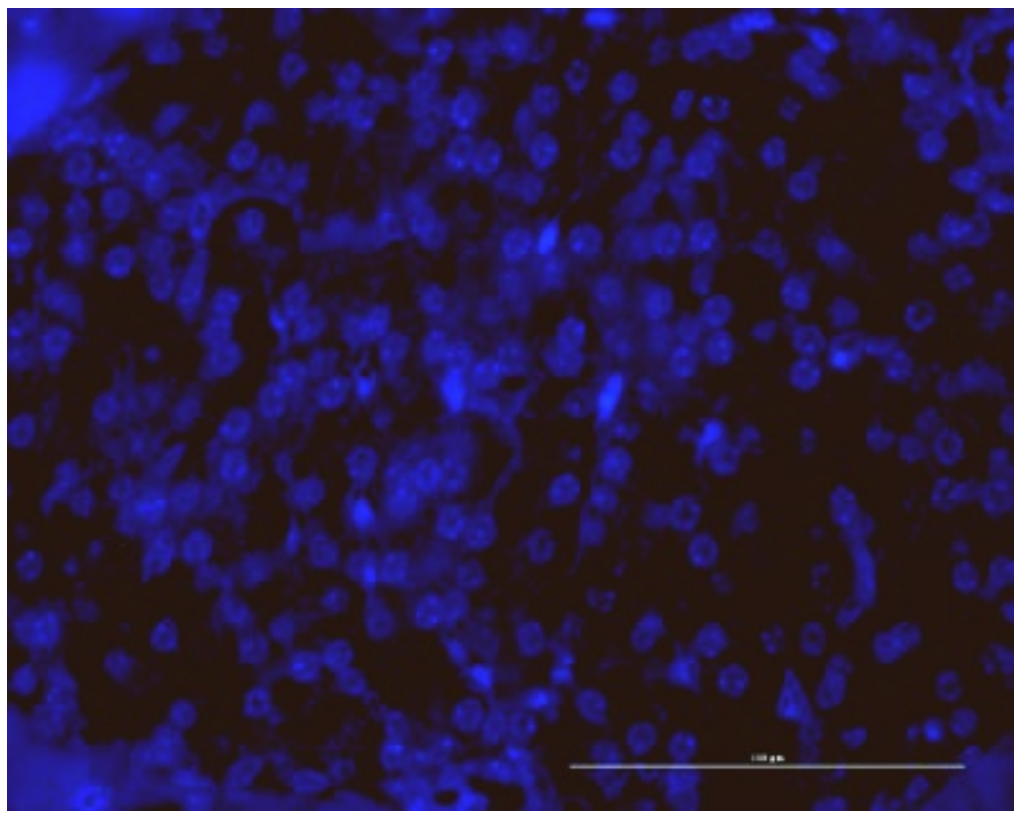

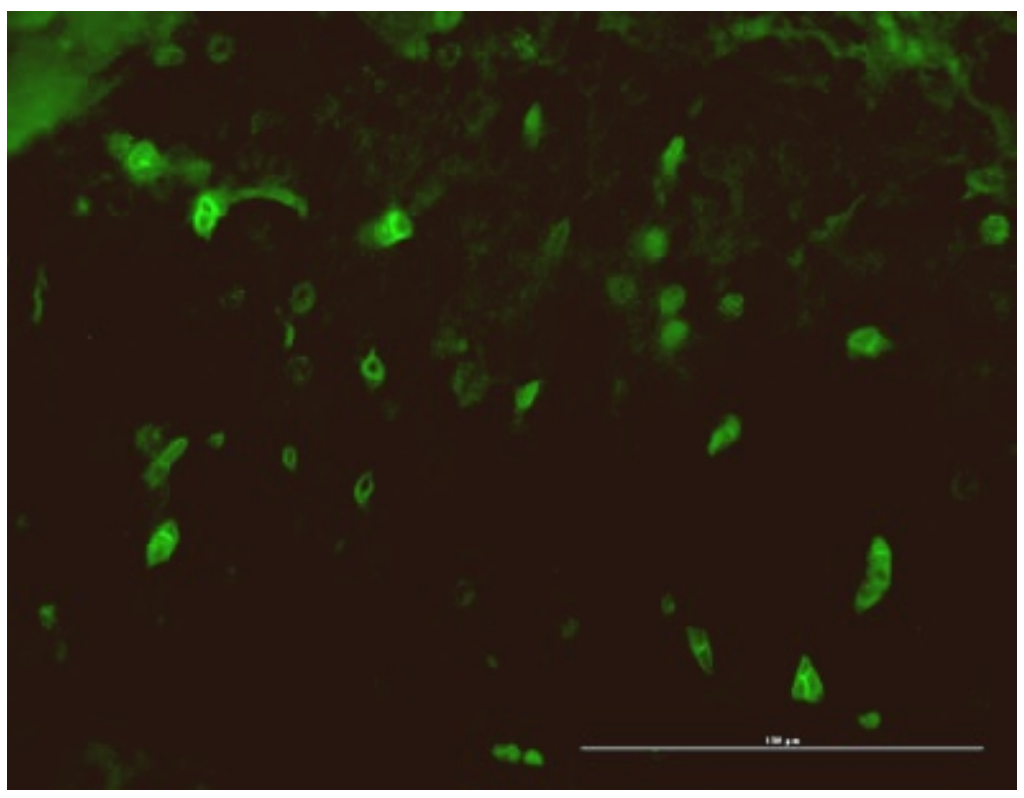

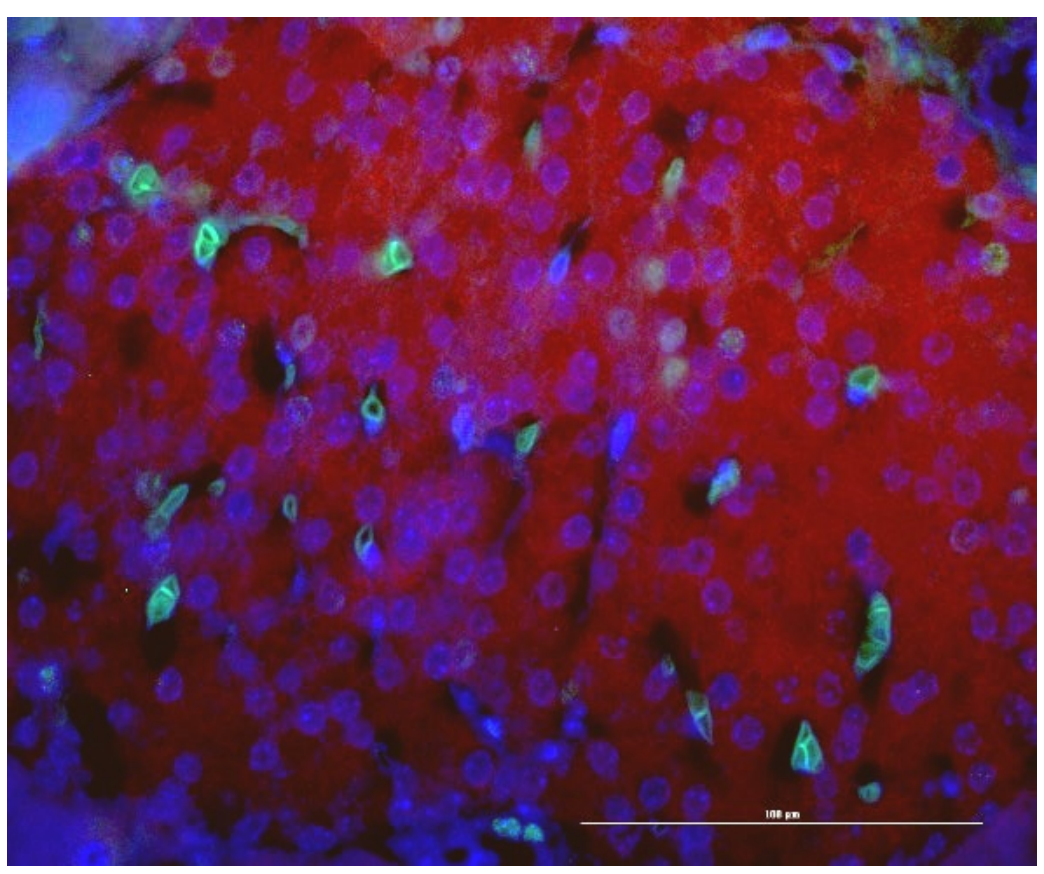

ABX+TAC+L-HIS

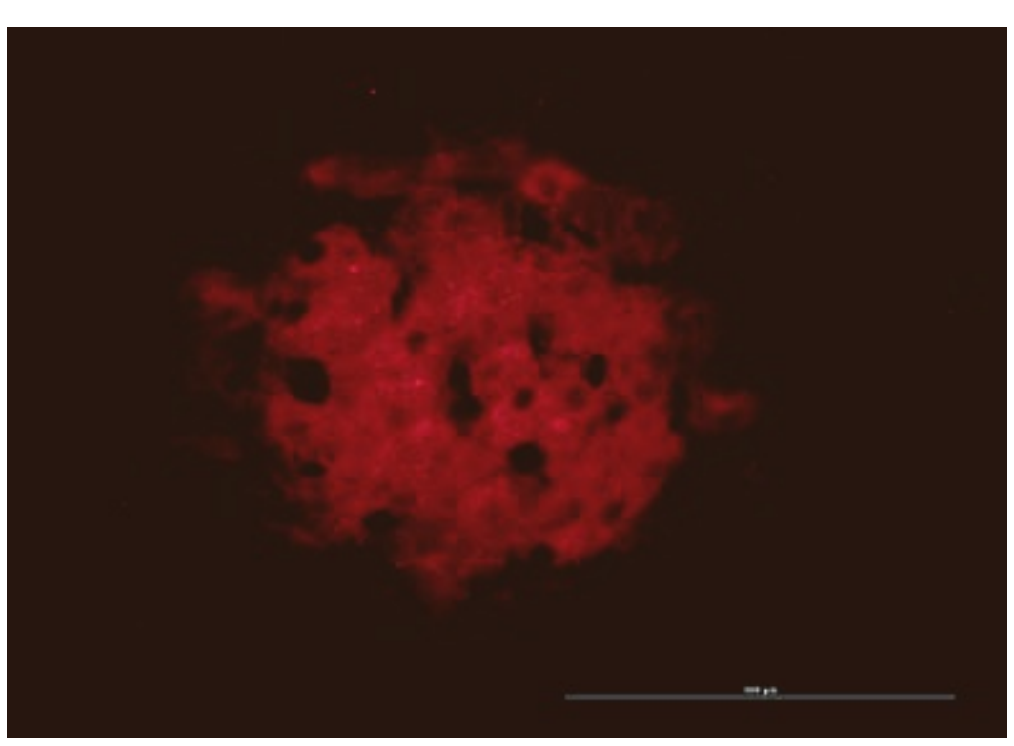

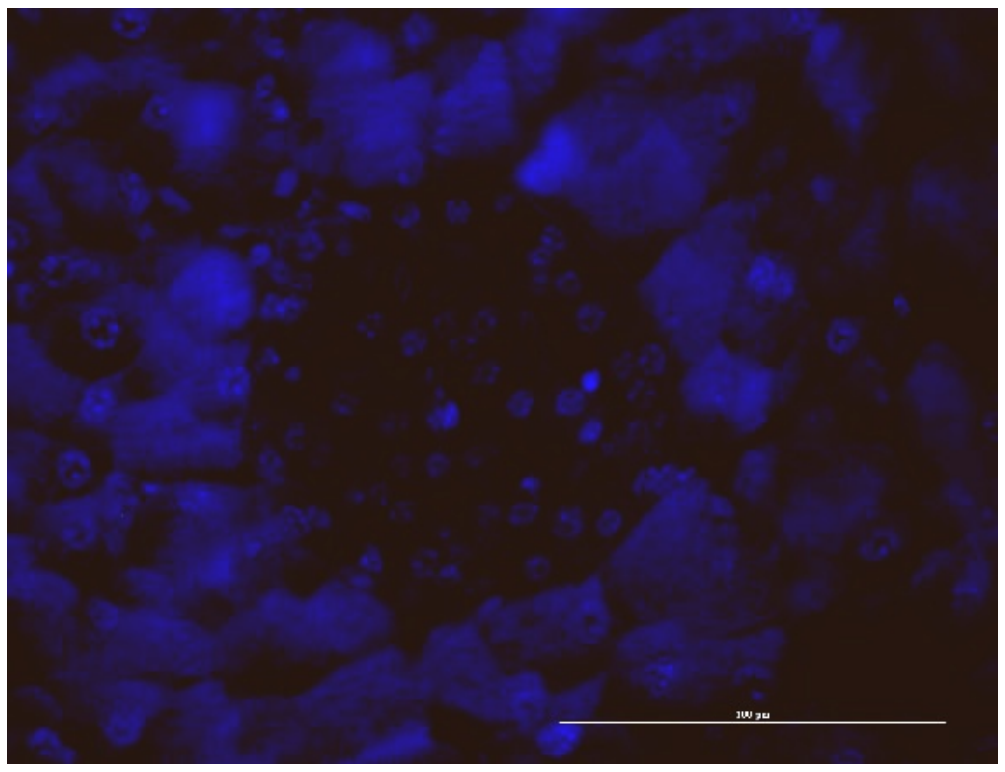

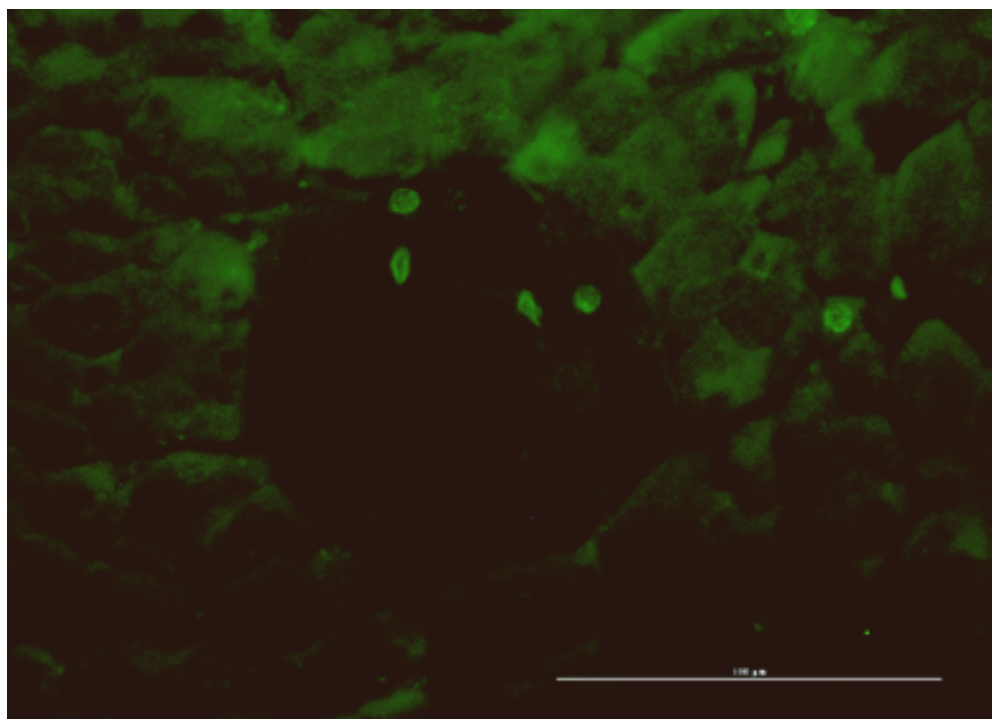

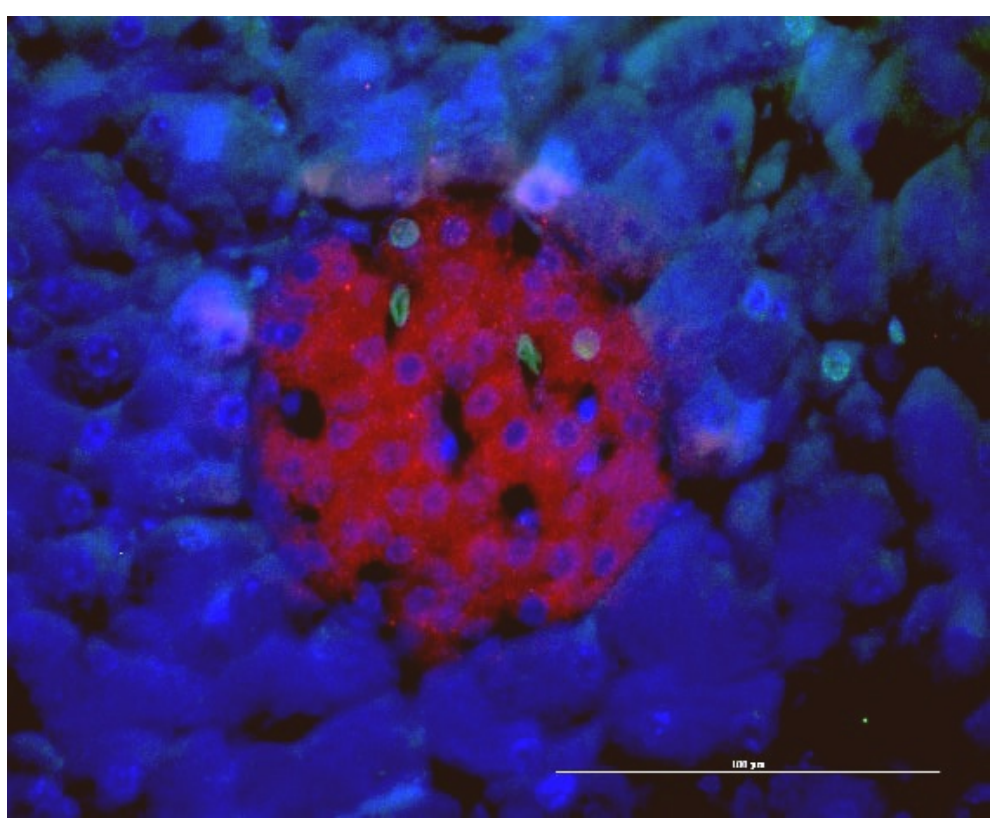

FIGURE 7E

CONTROL

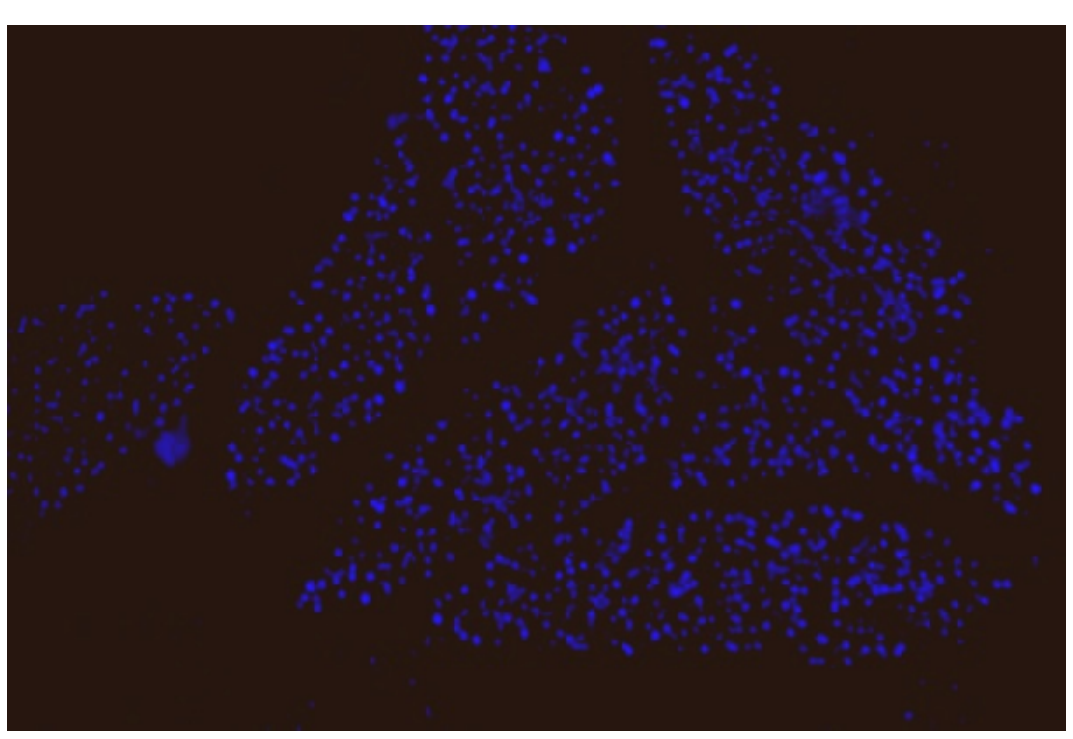

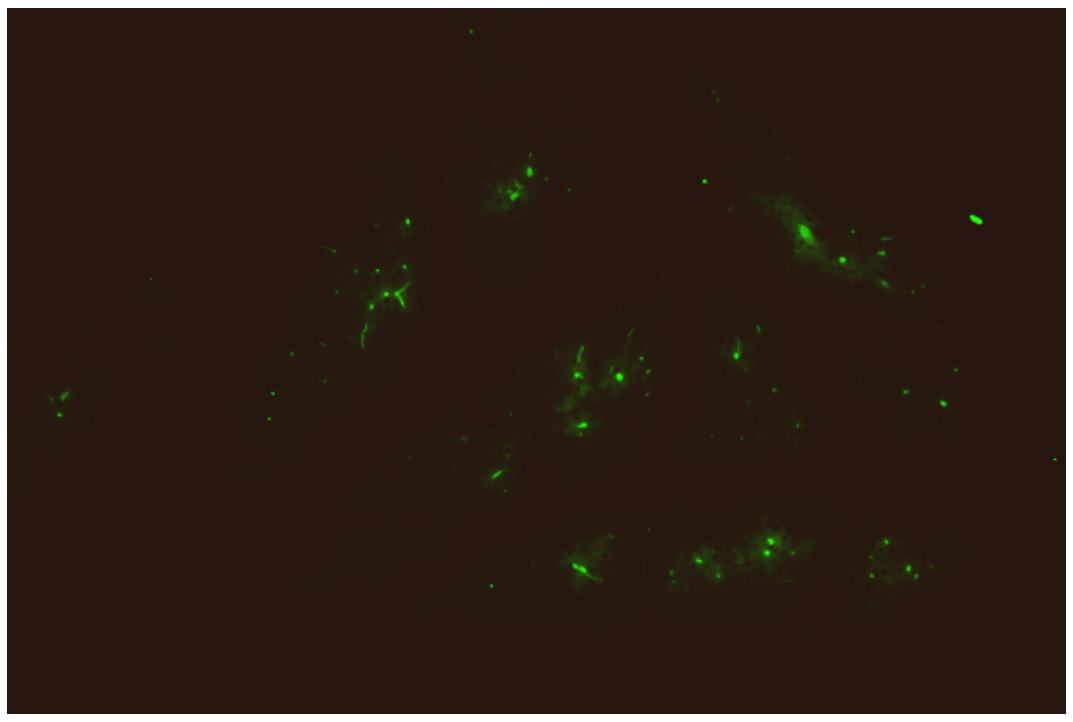

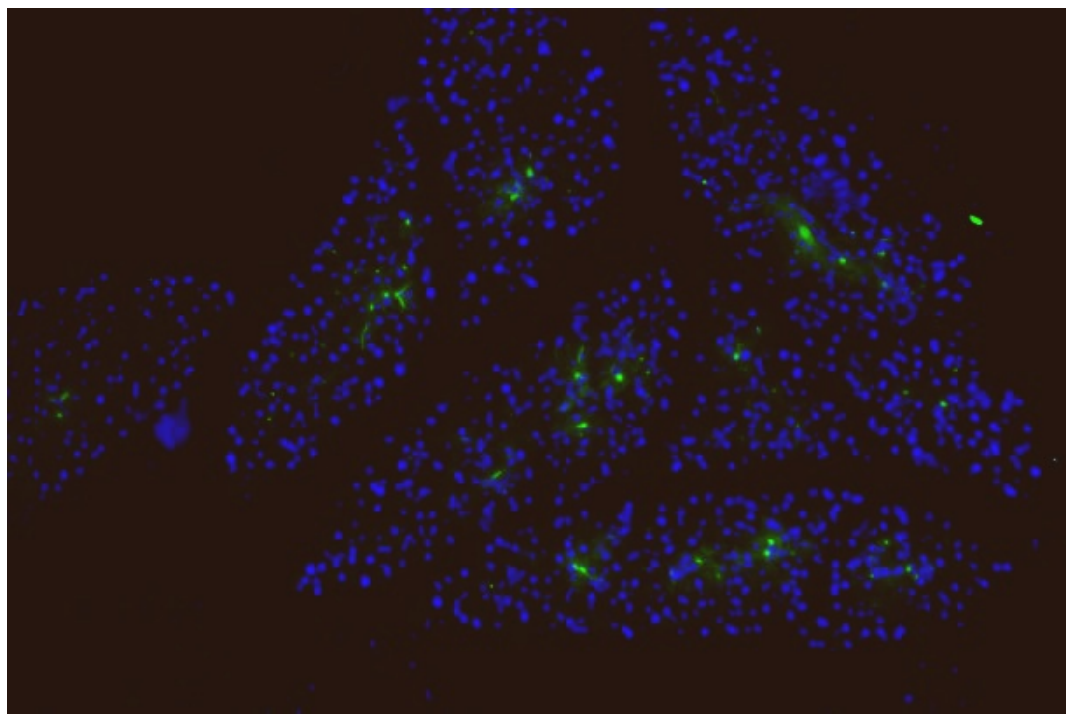

TAC

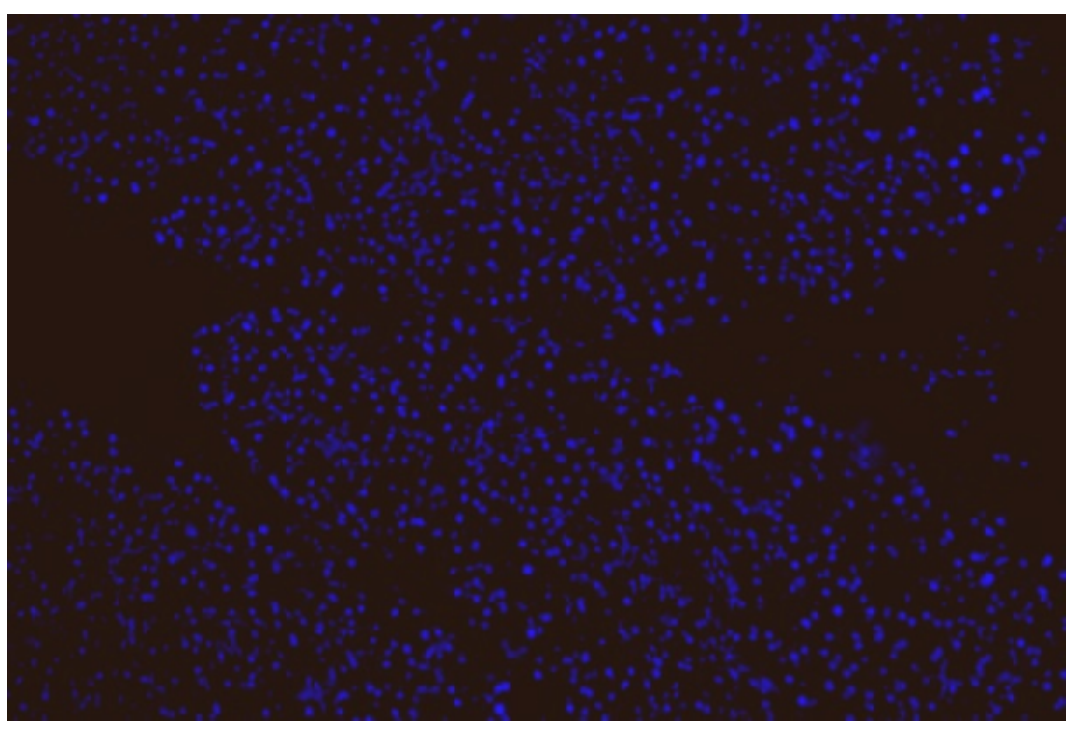

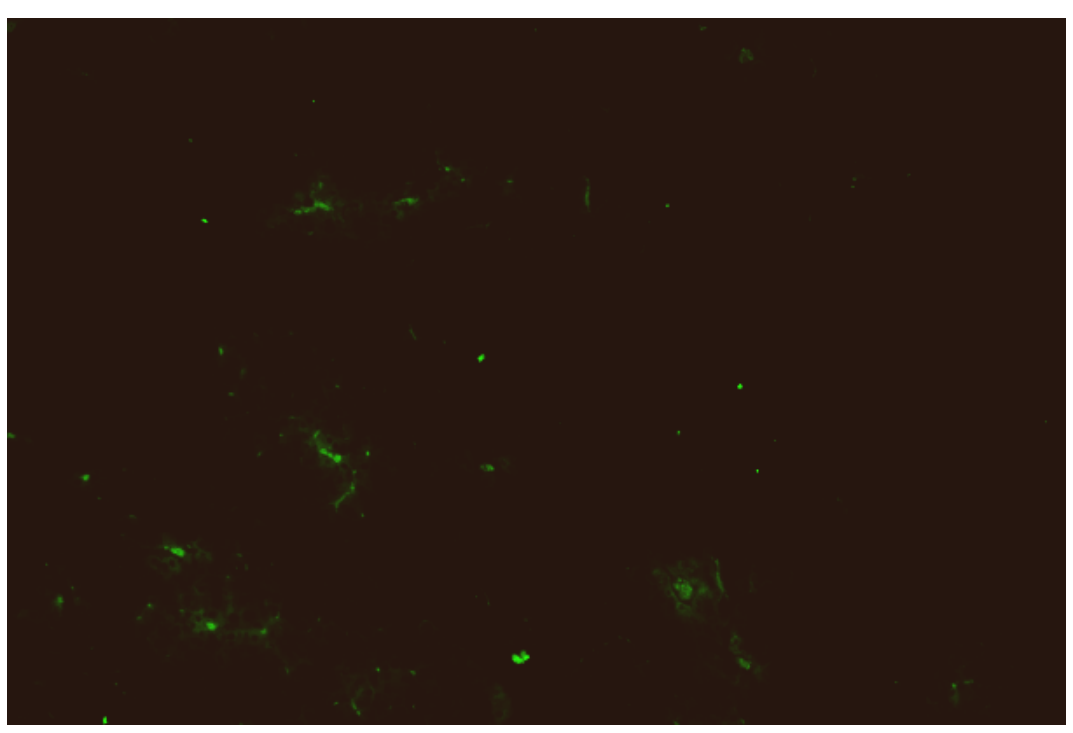

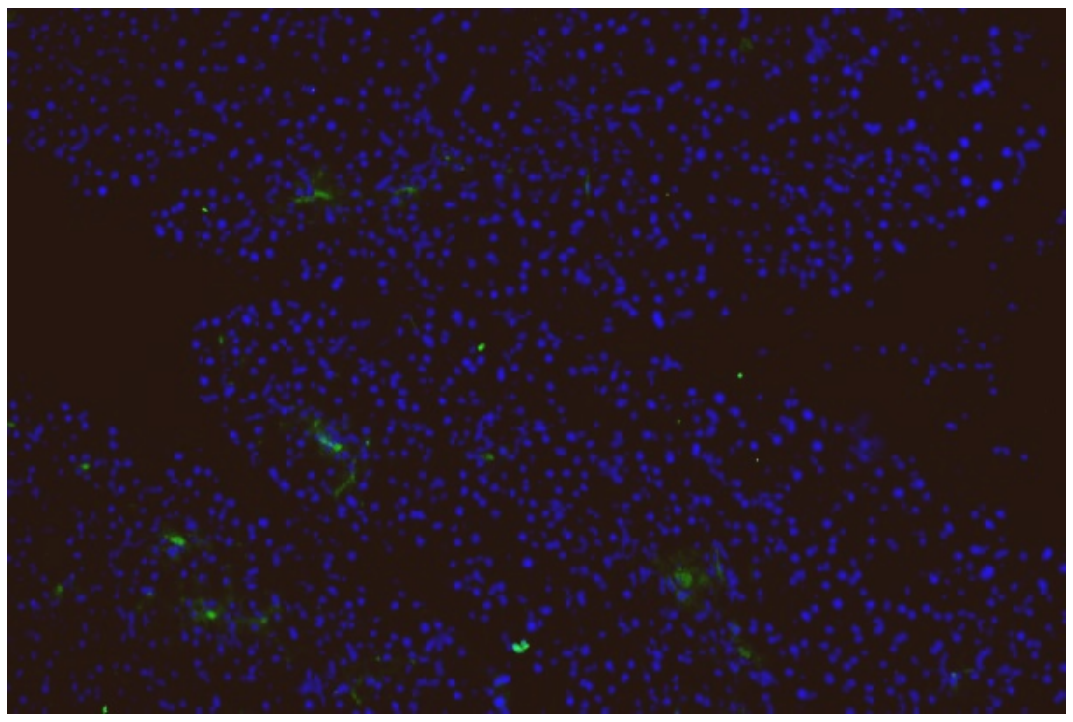

TAC+L-HIS

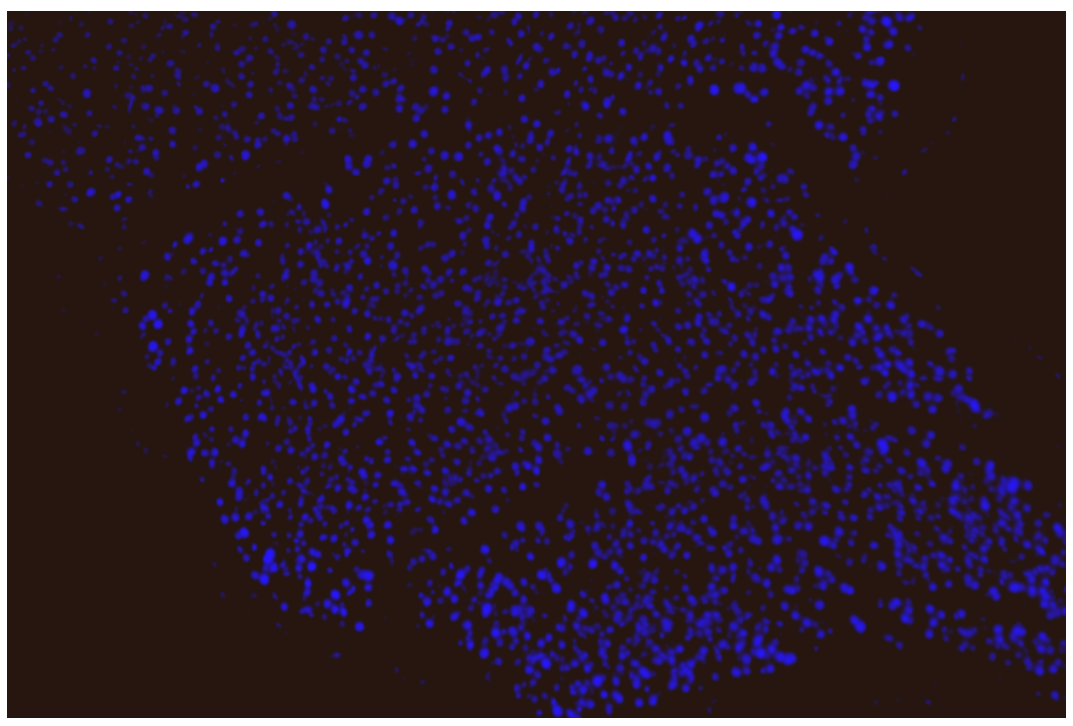

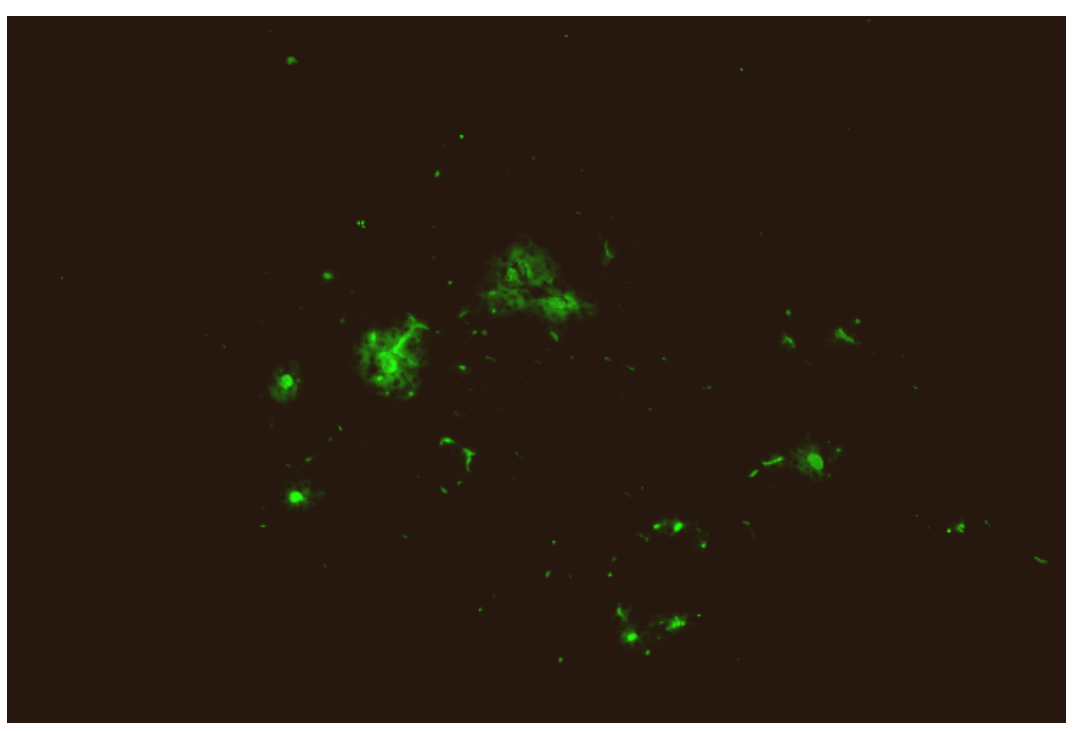

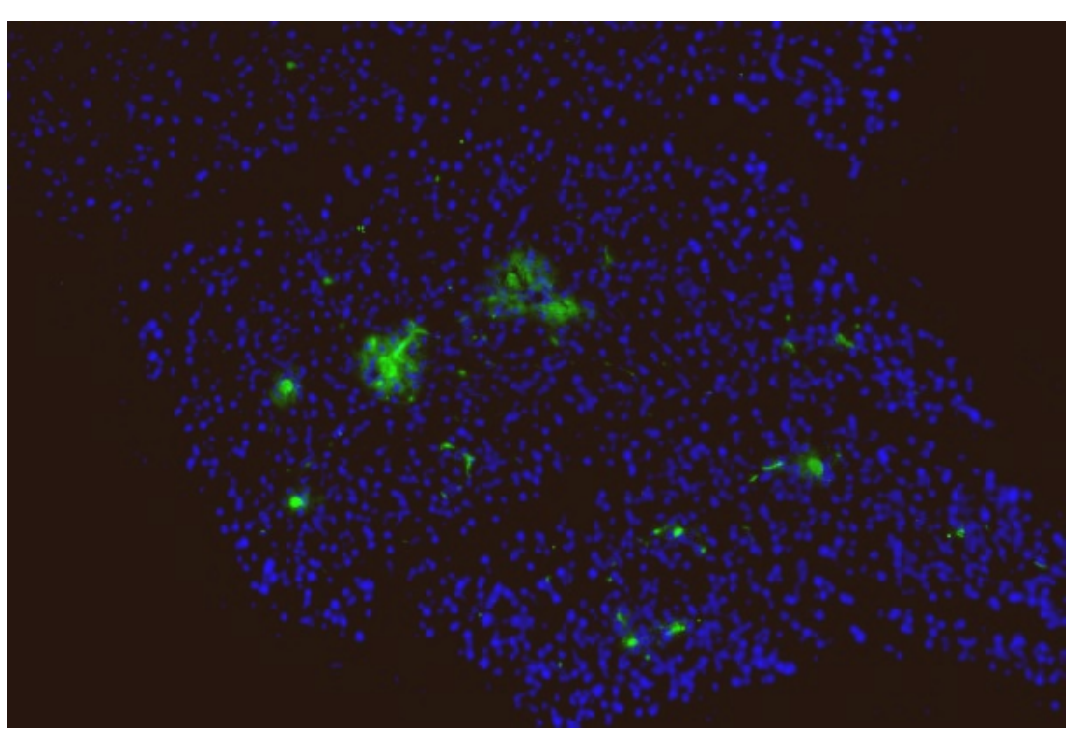

FIGURE 7G

CONTROL

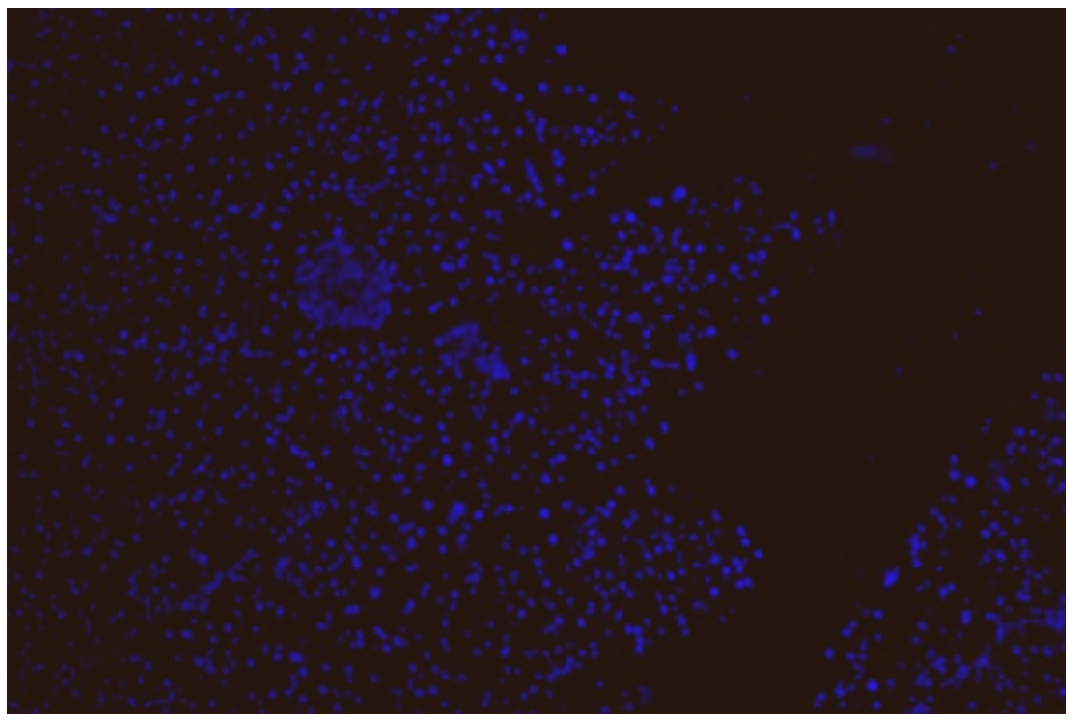

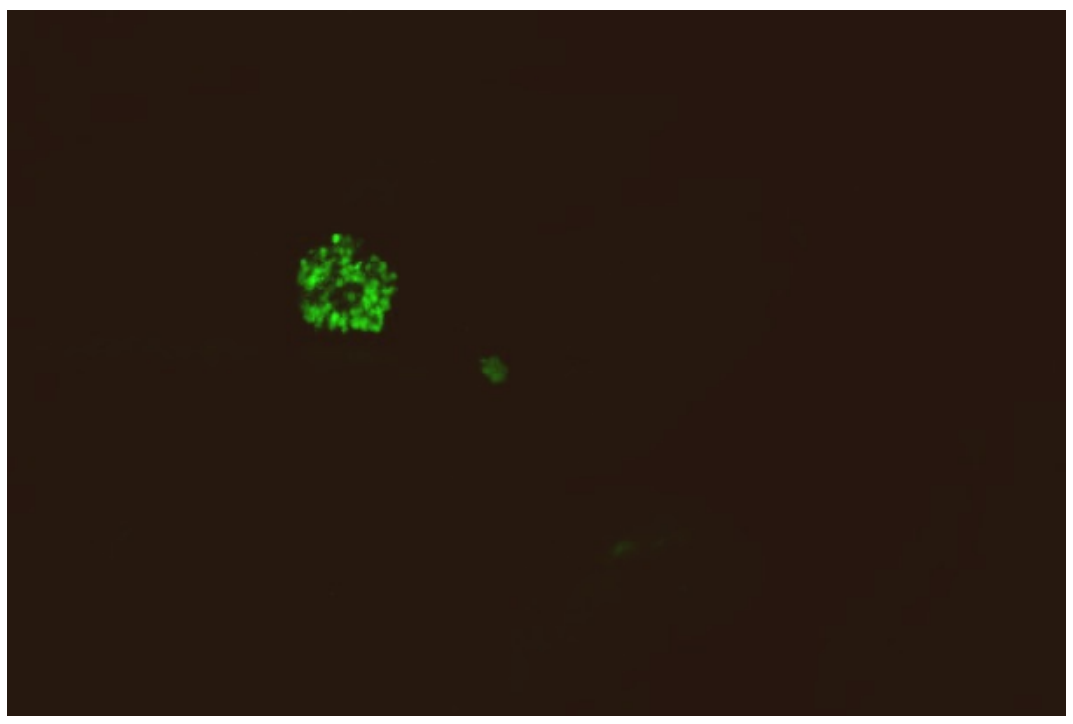

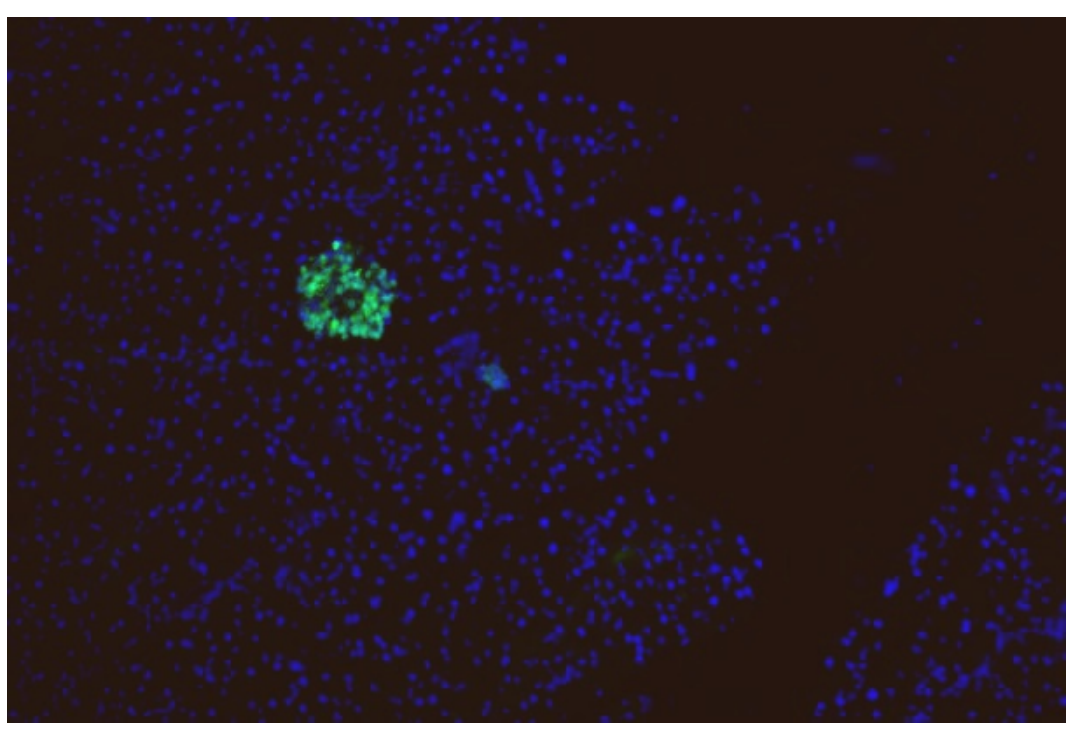

TAC

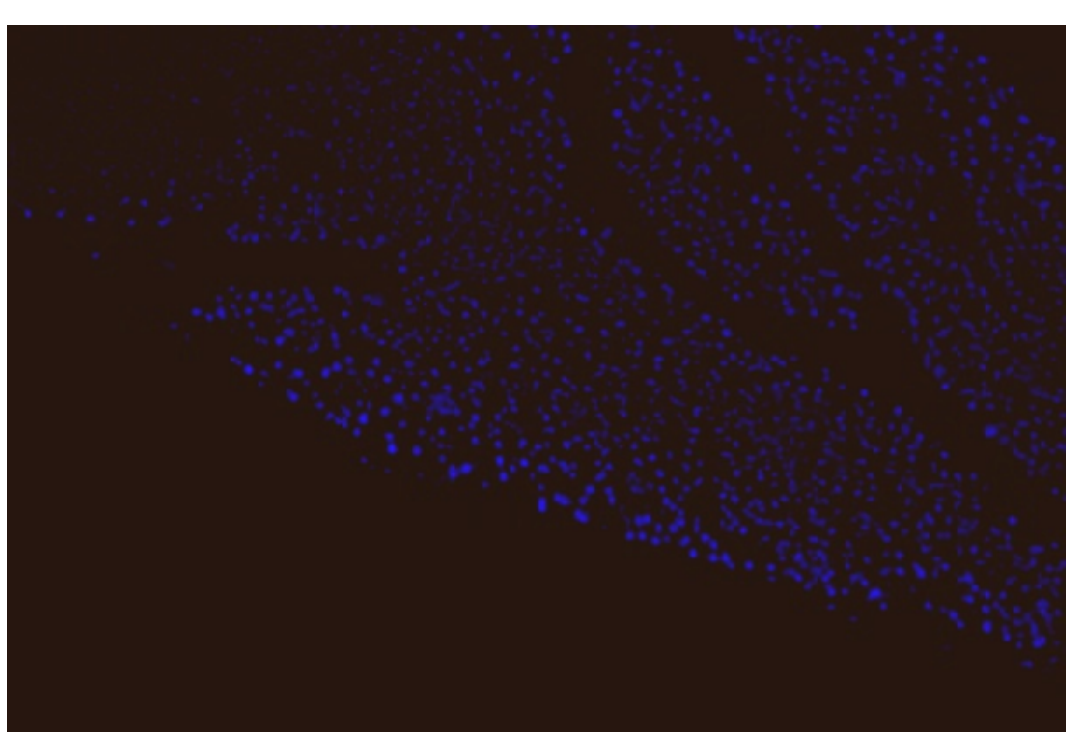

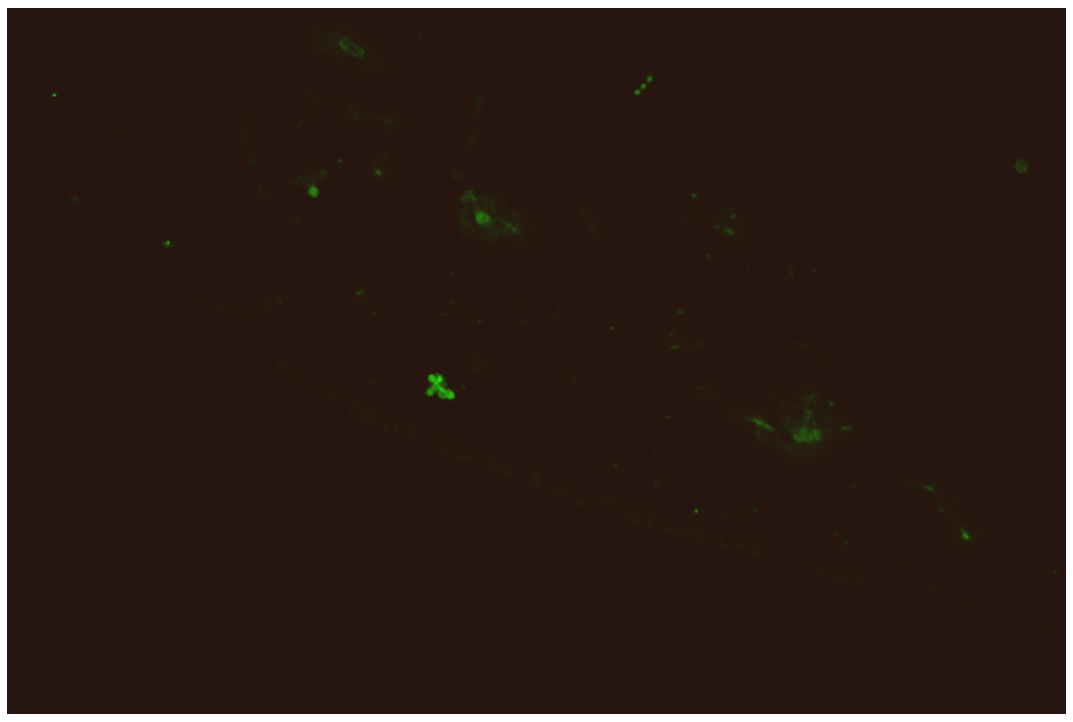

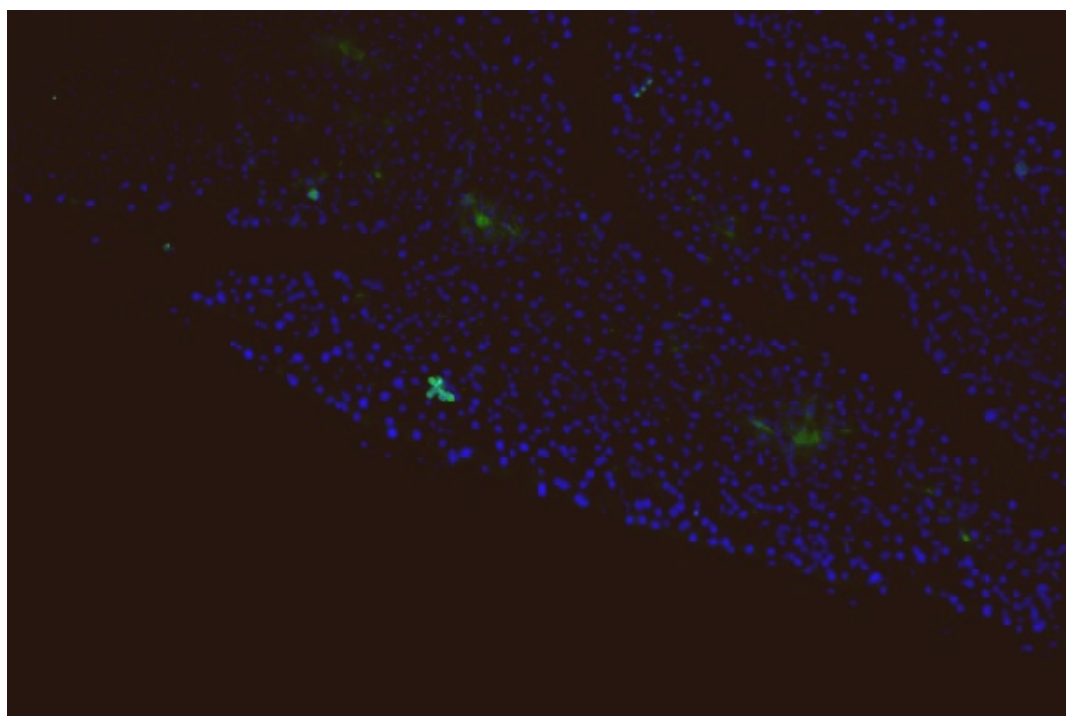

TAC+L-HIS

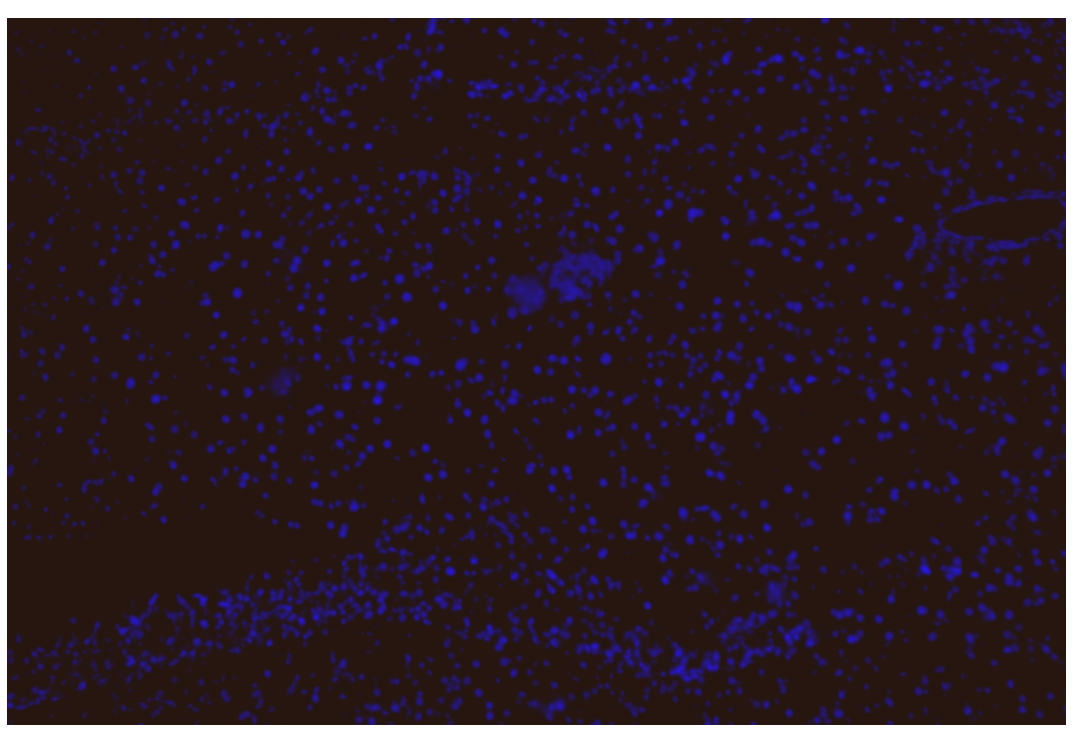

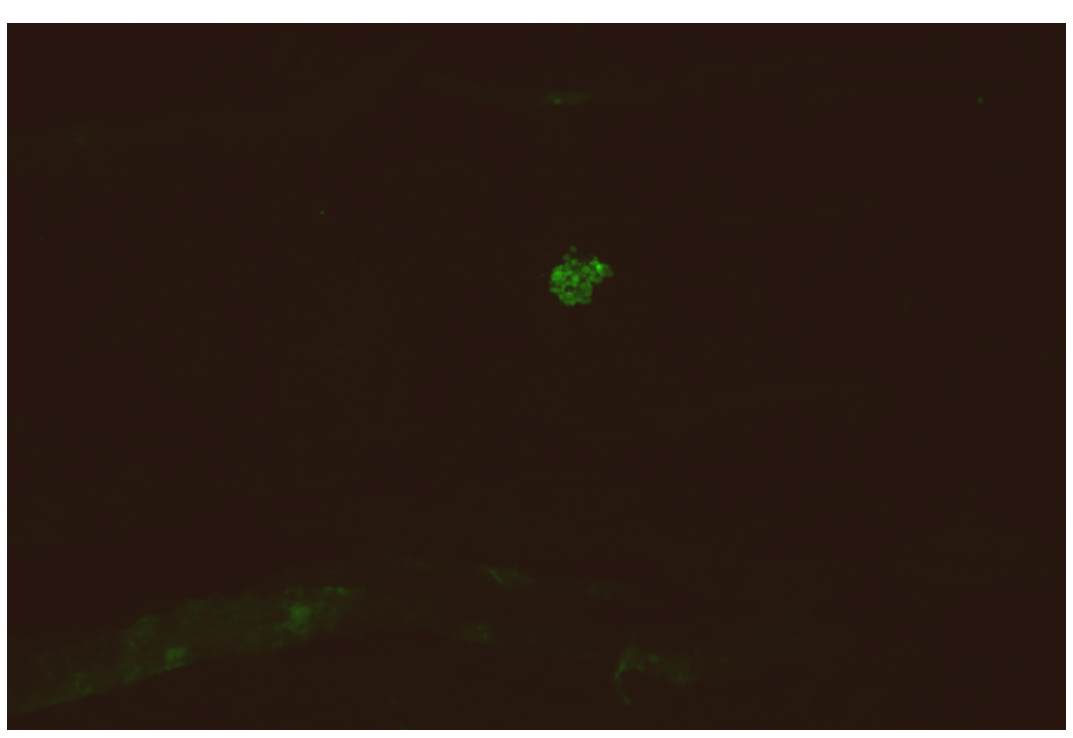

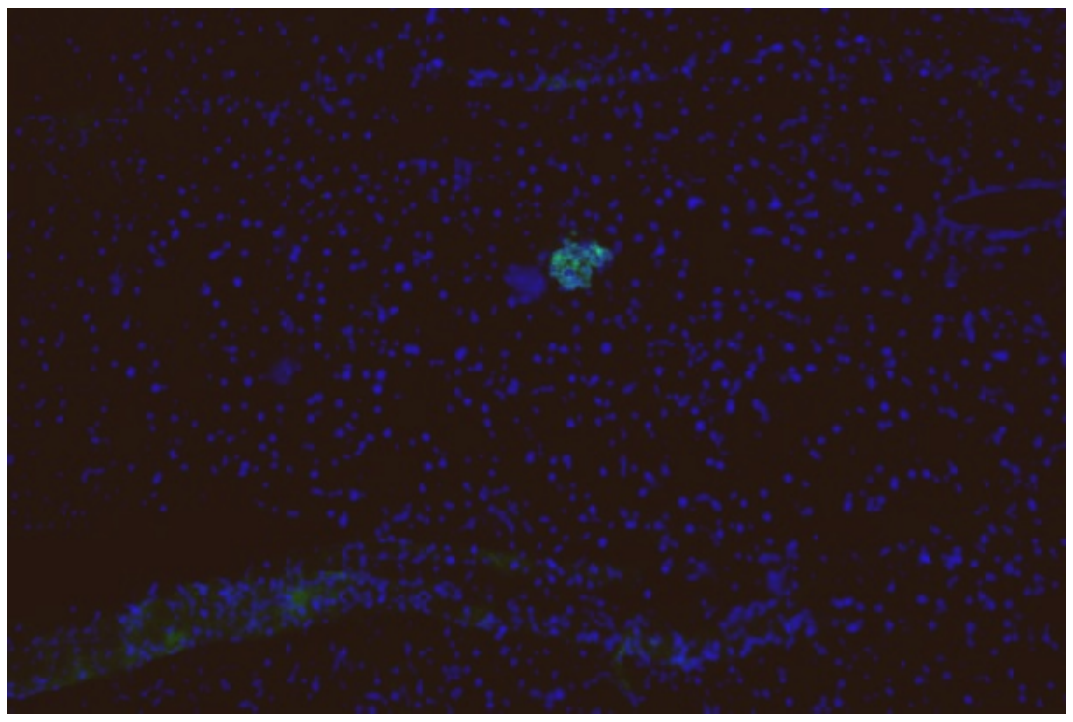

FIGURE 7I

CONTROL

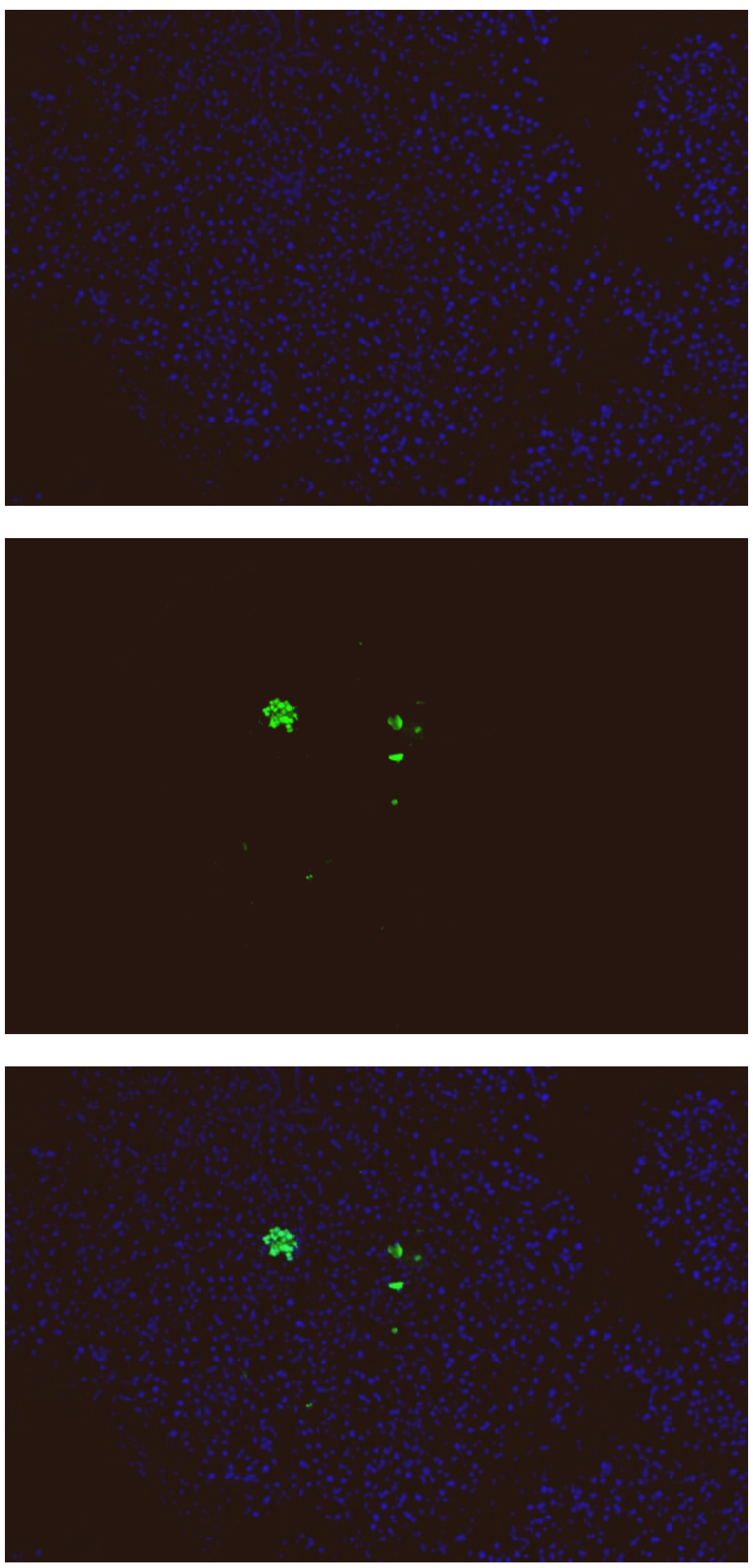

TAC

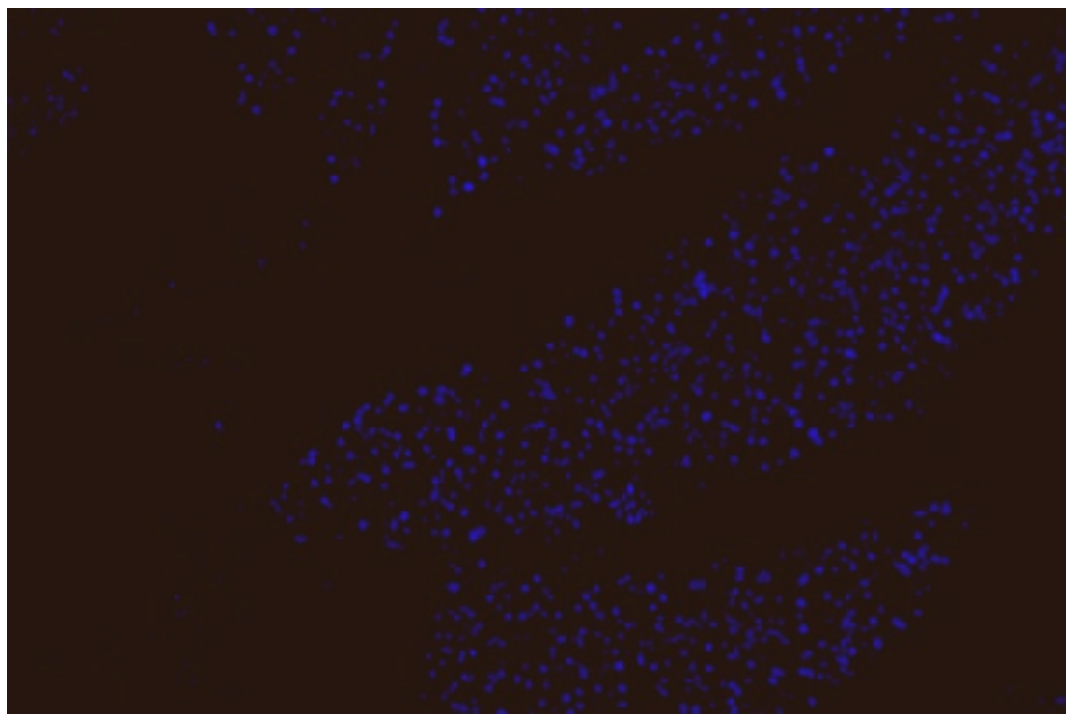

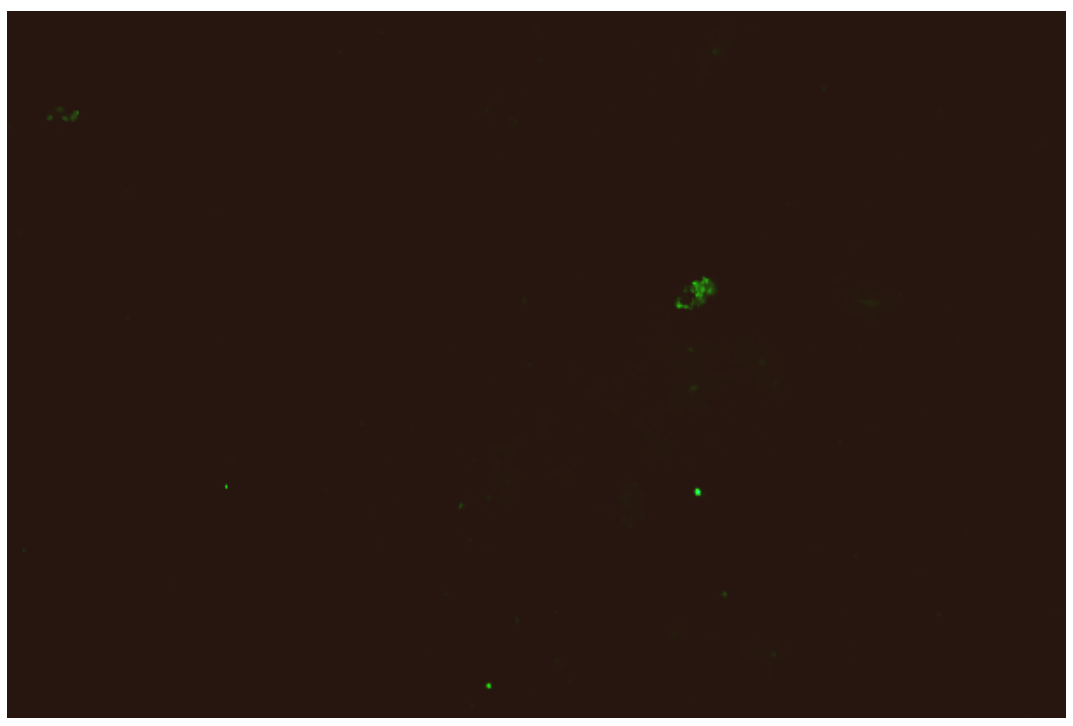

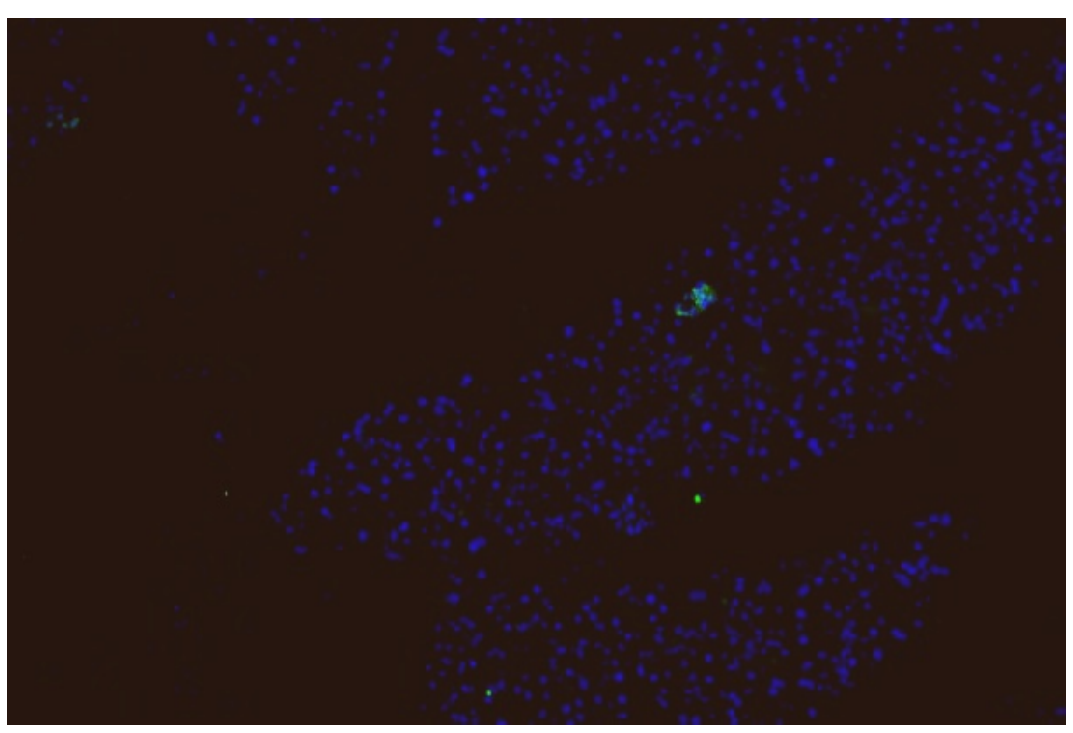

TAC+L-HIS

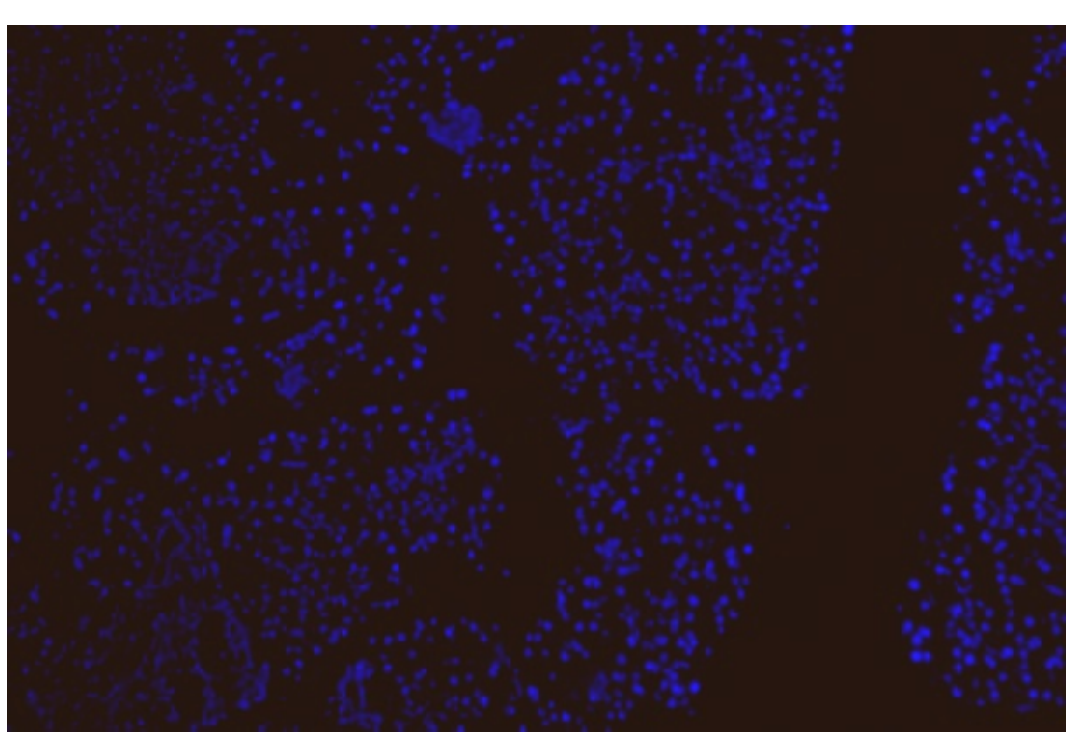

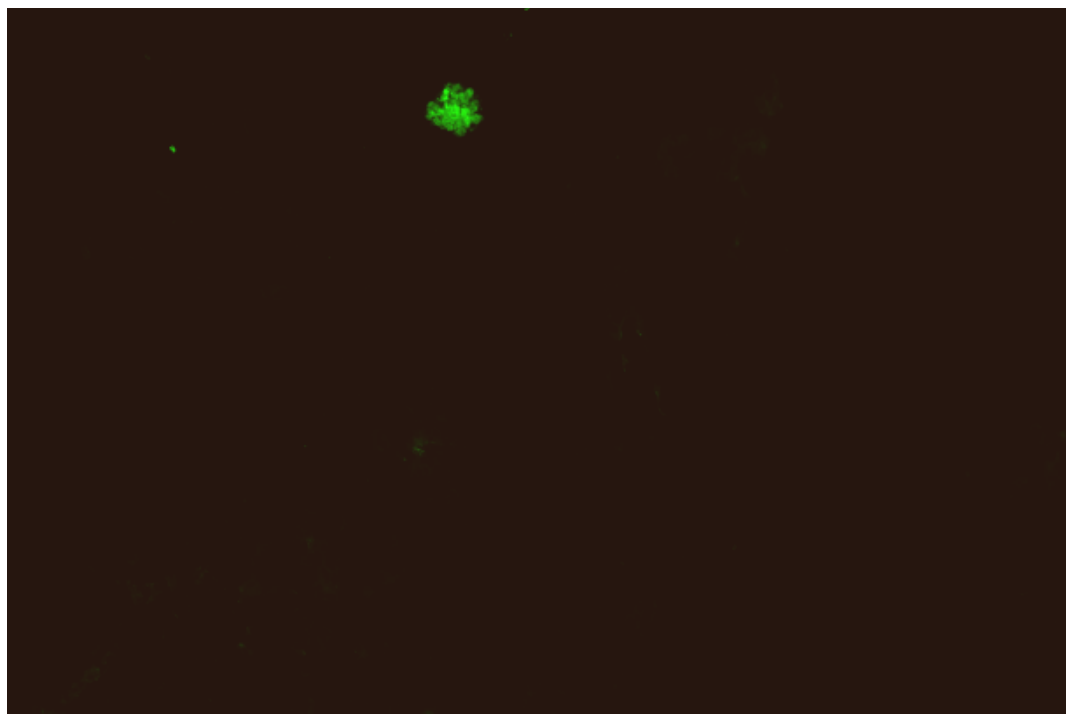

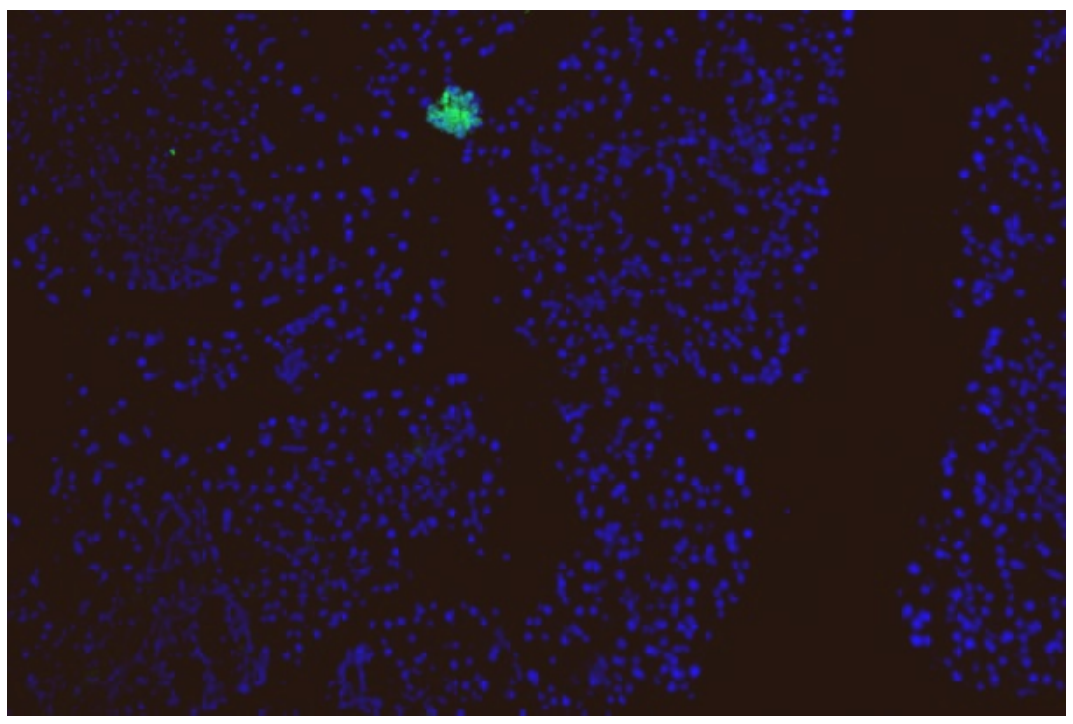

FIGURE 7K

CONTROL

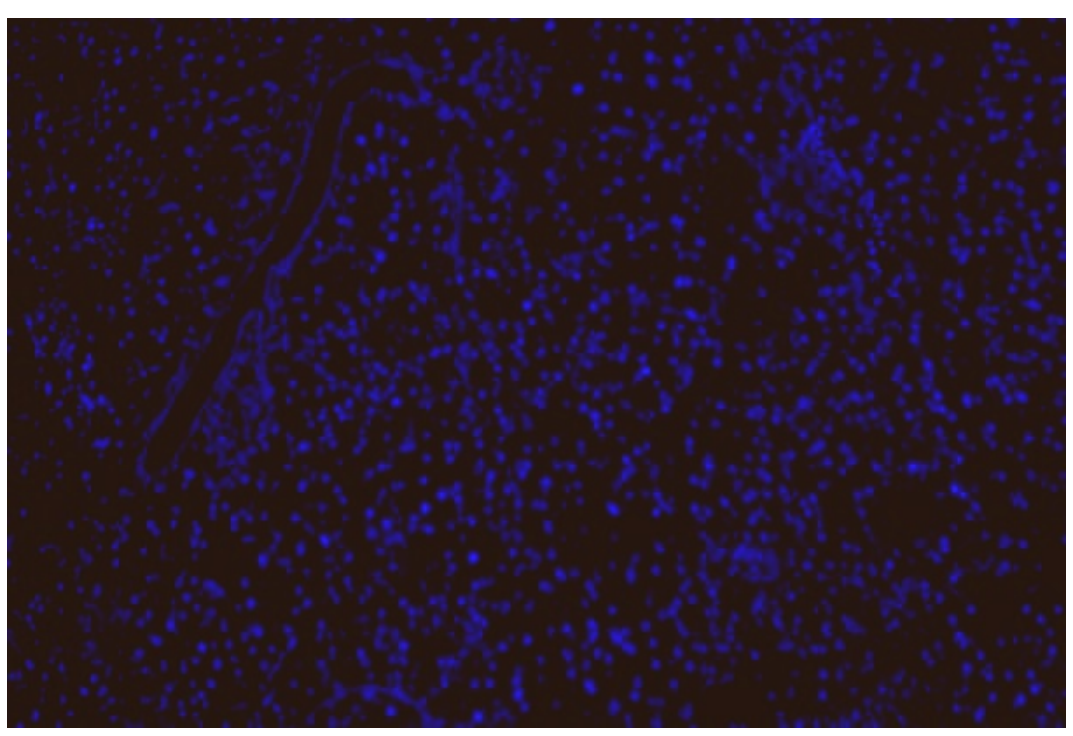

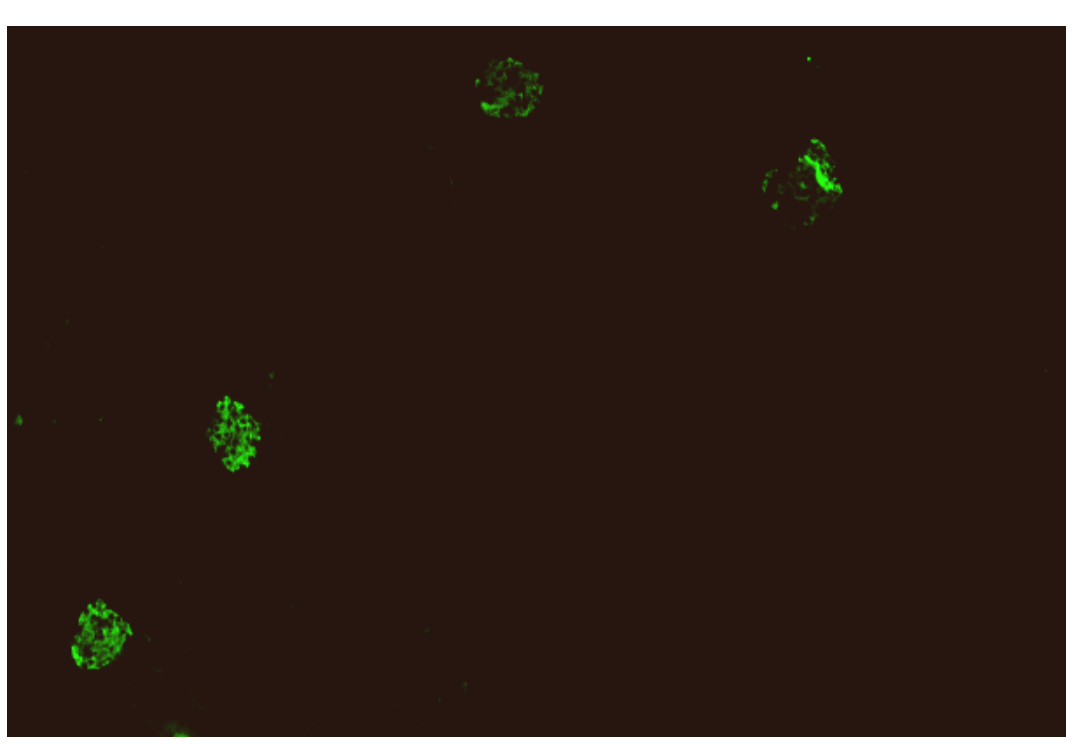

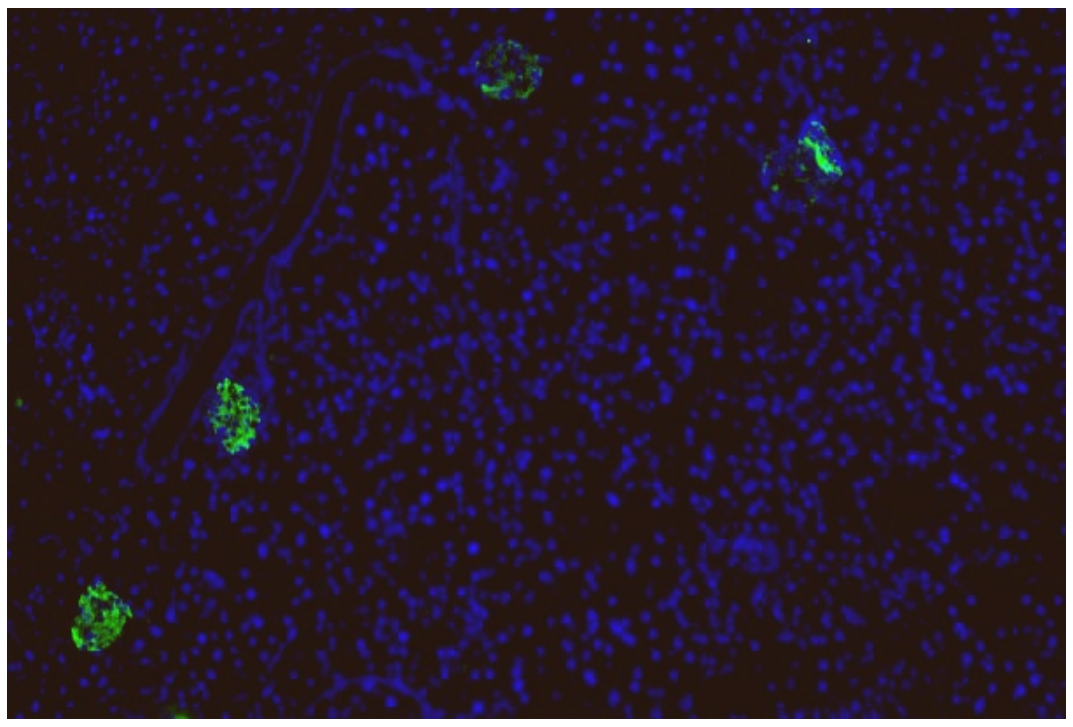

TAC

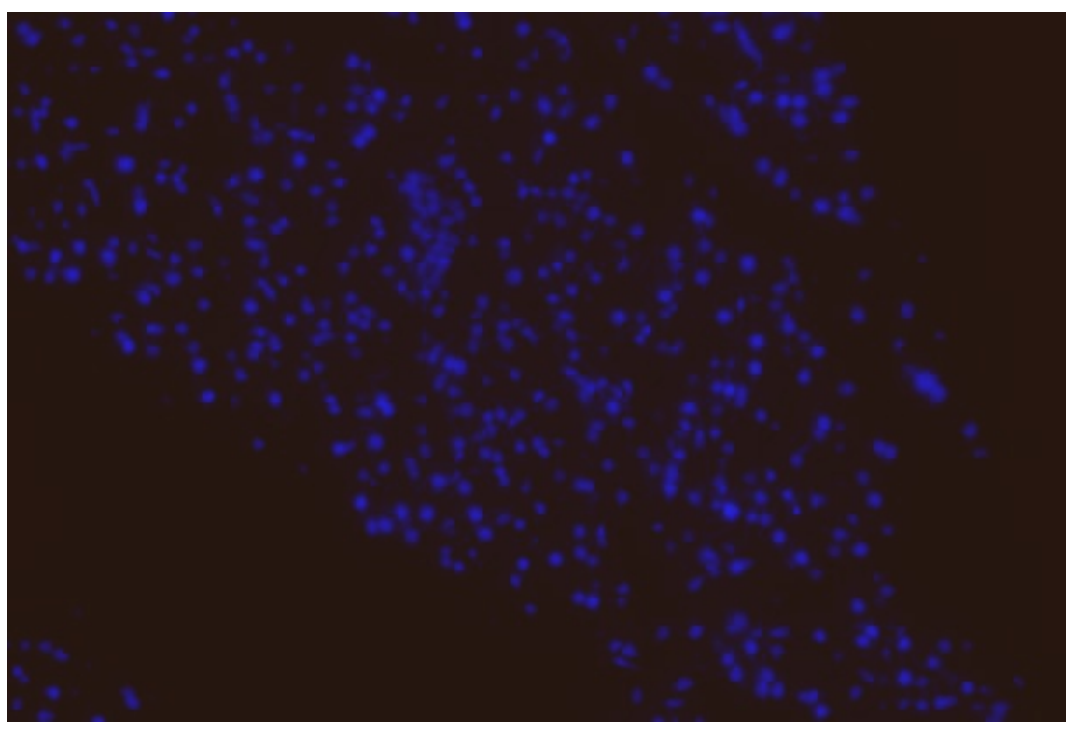

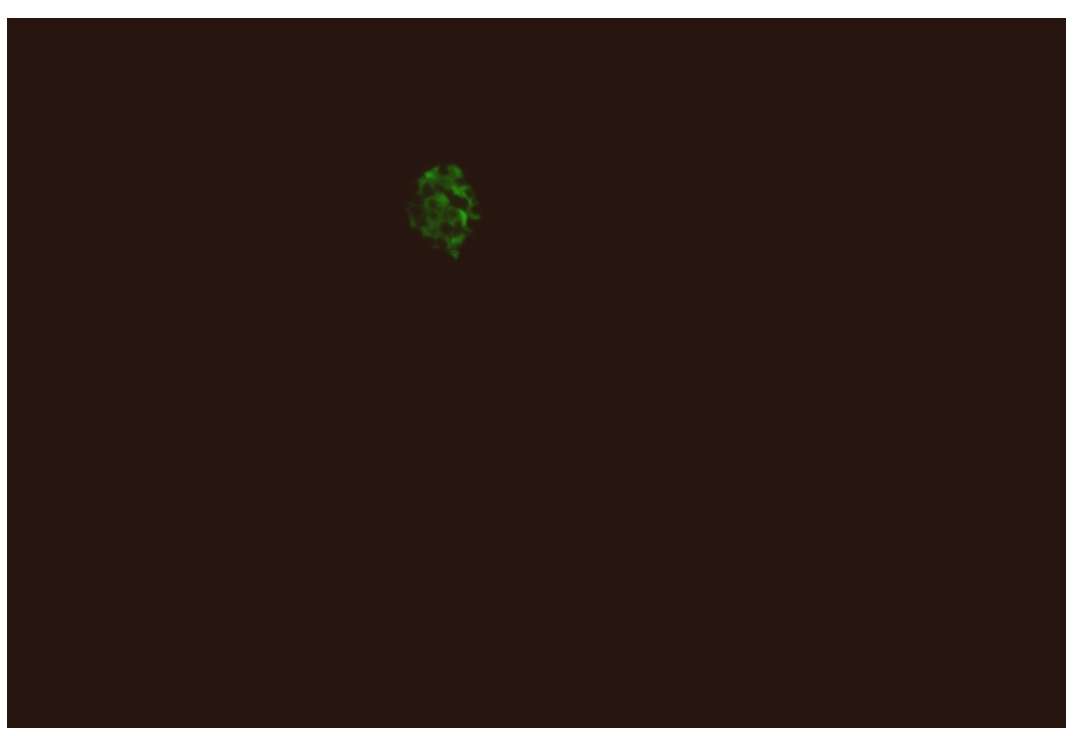

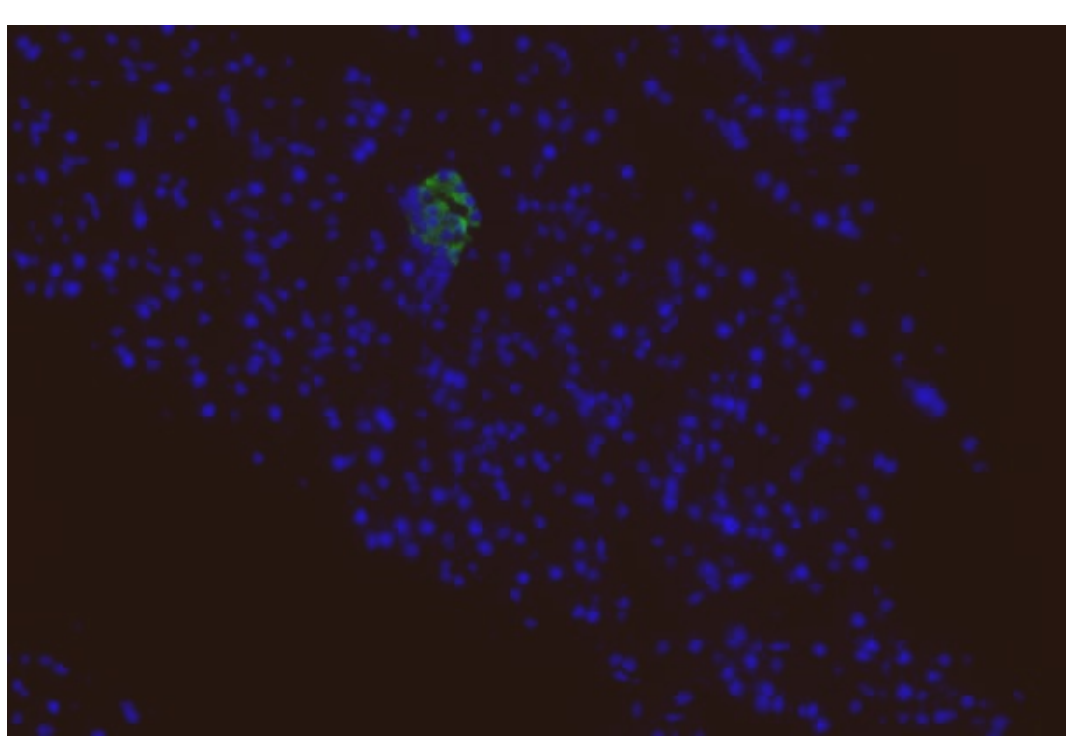

TAC+L-HIS

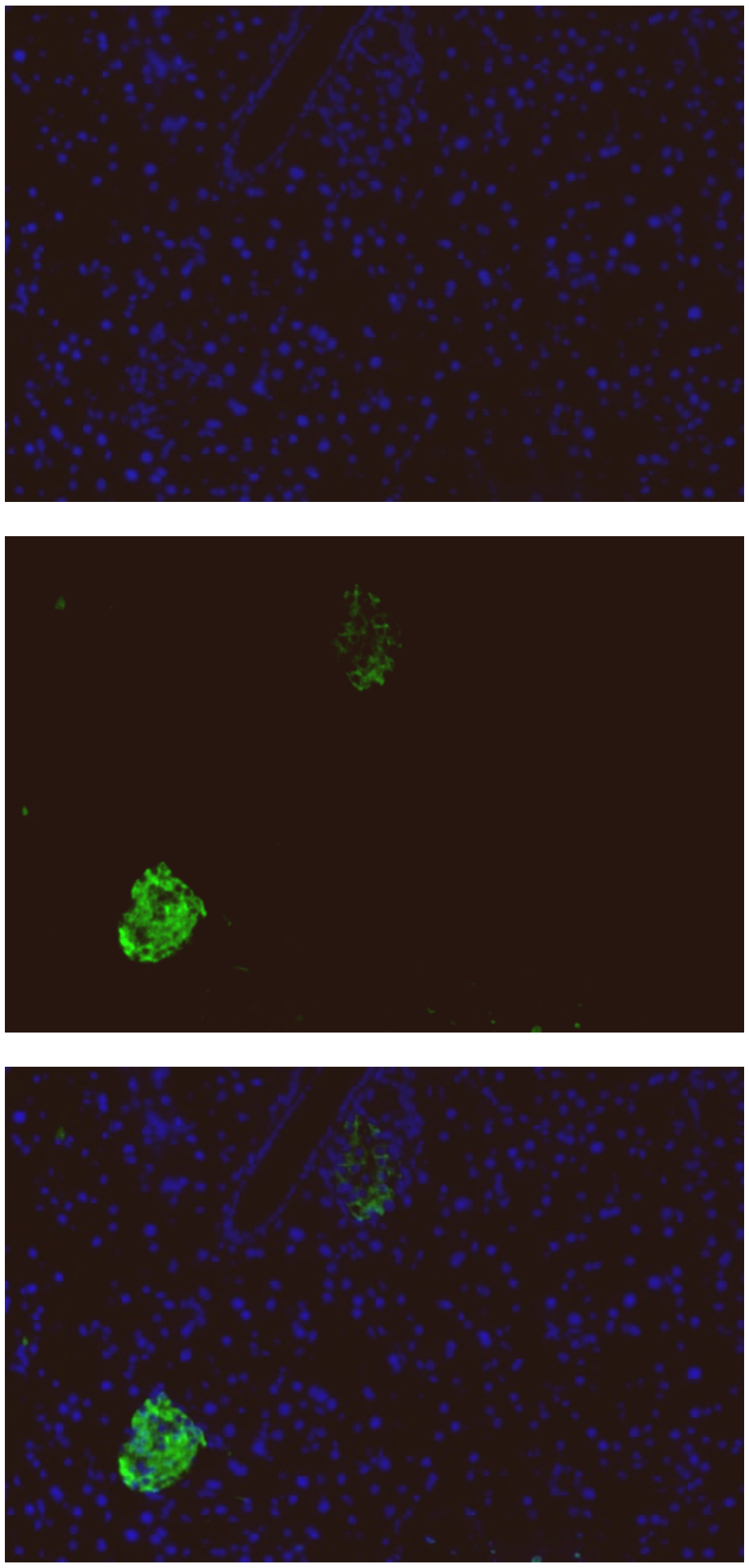

Supplement: Supplementary file 1 [file mmc1.pdf]
